# Nucleophilic arylation with tetraarylphosphonium salts

**DOI:** 10.1038/ncomms10337

**Published:** 2016-01-29

**Authors:** Zuyong Deng, Jin-Hong Lin, Ji-Chang Xiao

**Affiliations:** 1 Key Laboratory of Organofluorine Chemistry, Shanghai Institute of Organic Chemistry, Chinese Academy of Sciences, 345 Lingling Road, Shanghai 200032, China

## Abstract

Organic phosphonium salts have served as important intermediates in synthetic chemistry. But the use of a substituent on the positive phosphorus as a nucleophile to construct C–C bond remains a significant challenge. Here we report an efficient transition-metal-free protocol for the direct nucleophilic arylation of carbonyls and imines with tetraarylphosphonium salts in the presence of caesium carbonate. The aryl nucleophile generated from phosphonium salt shows low basicity and good nucleophilicity, as evidenced by the successful conversion of enolizable aldehydes and ketones. The reaction is not particularly sensitive to water, shows wide substrate scope, and is compatible with a variety of functional groups including cyano and ester groups. Compared with the arylmetallic reagents that are usually moisture sensitive, the phosphonium salts are shelf-stable and can be easily handled.

## Abstract (web summary)

Carbon based nucleophiles often are synthetically limited by the high basicity/reactivity of the starting materials. Here, the authors show that tetraarylphosphonium salts can be induced to act as C-aryl nucleophiles for the addition to carbonyls and imines.

## Graphical abstract

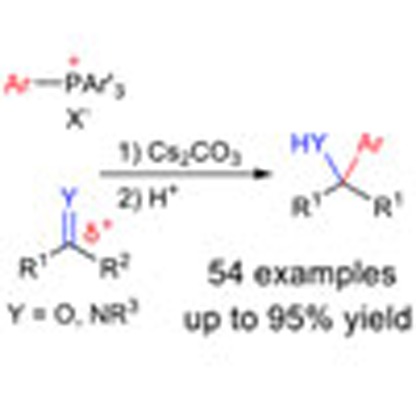

Significant advances in C-arylation have emerged in the past few decades because this reaction can effectively form C–C bonds to provide valuable molecules that are the core structures of natural products or pharmaceuticals^1^,^2^,^3^,^4^,^5^. Recently, intensive studies have been devoted to the exploration of efficient methods for arylation of carbonyl and imino compounds, allowing access to α-aryl-alcohols and α-aryl-amines, which are abundant in biologically active molecules^1^,^2^. Traditional arylmetallic reagents, which are usually generated *in situ* under Barbier-type conditions^6^,^7^,^8^ or prepared in advance like arylmagnesium reagents^9^,^10^,^11^, have long been used for nucleophilic addition to carbonyl or imino groups. However, this approach often suffers from narrow substrate scope, or the high moisture sensitivity and the strong basicity of the reagents. Transition-metal-catalysed arylation of carbonyl or imino groups with aryl halides^12^,^13^, arylboronic acid derivatives^14^,^15^,^16^,^17^,^18^,^19^,^20^ or other reactive reagents^21^,^22^,^23^,^24^,^25^ showed good functional group tolerance under mild reaction conditions. However, the high cost of noble transition metal complexes and possible contamination of the end products by trace amounts of heavy metals probably limit the wide application of this approach in industrial and pharmaceutical processes. Therefore, it is highly desirable to explore broadly applicable transition-metal-free methods for the arylation of carbonyl and imino compounds.

Our research interest in the chemistry of organic phosphonium salts^26^,^27^,^28^,^29^ prompted us to investigate their application in arylation reactions. Organic phosphonium salts have served as important intermediates in synthetic chemistry^30^,^31^,^32^,^33^,^34^,^35^. Although phosphonium salts are electrophilic species and the positive phosphorus could enhance the electrophilicity^36^ or acidity^34^,^35^,^37^ of its substituents because of the inductive effect, we speculated that the substituent on the phosphorus might be converted to a nucleophile initiated by another appropriate nucleophile, which may firstly attack the phosphorus and then result in the cleavage of a substituent with nucleophilic ability (Fig. 1).

Herein we report a nucleophilic arylation of carbonyl and imino compounds with tetraarylphosphonium salts in the presence of caesium carbonate (Fig. 1). The generated aryl nucleophiles exhibit low basicity and good nucleophilicity. The arylation protocol shows wide substrate scope and a high level of functional group tolerance. Mechanistic investigation reveals that the reaction is predominantly initiated by caesium carbonate.

## Results

### Optimization of reaction conditions

Considering the high affinity between phosphorus and oxygen, an O-nucleophile was used to initiate the arylation of 4-phenylbenzaldehyde (**1a**) with tetraphenylphosphonium iodide (**2a**) (Table 1, entries 1–4). Neither Na_2_CO_3_ nor K_2_CO_3_ could initiate the transformation (entries 1 and 2). Interestingly, Cs_2_CO_3_ was found to be a good initiator (entry 3), which is likely because of the better solubility of the caesium salt. Because the caesium salt was effective, another O-initiator with caesium as the cation (CsOAc; entry 4) was examined. To our surprise, no desired product was detected, which might be caused by the lower nucleophilicity of the acetate anion. Further screening of non-O-initiators (entries 5 and 6) showed that Cs_2_CO_3_ was the best initiator (entry 3), indicating that the high P–O affinity may have an important role in this reaction. The yield was increased to 65% with the elongation of reaction time (entry 7 versus entry 3). The effects of other solvents were also investigated (entries 8–10). The reaction in 1,4-dioxane gave the product in comparable yield (entry 8 versus entry 7), while acetonitrile was not as effective, probably because its acidic α-proton can quench the leaving phenyl group from salt **2a** (entry 10). Increasing the loading of salt **2a** and caesium carbonate (entries 11 and 12) increased the yield significantly (entry 12), while lowering the temperature led to significantly lower yield (entry 13). The reaction was not particularly sensitive to water, as evidenced by the moderate yield obtained with the addition of water into the reaction system (entry 14). Both of the chloride (Ph_4_P^+^ Cl^−^) and bromide (Ph_4_P^+^ Br^−^) instead of the iodide **2a** gave lower yields, which should be due to the lower solubility of the chloride and bromide (entries 15–16).

### Substrate scope for phenylation of aldehydes

With the optimized reaction conditions in hand (Table 1, entry 12), we then explored the substrate scope for the phenylation of aldehydes with phosphonium salt **2a** (Table 2). The transformation can be applied to a variety of aldehydes and gave the desired products in moderate to excellent yields. Increasing the scale of the reaction by 20-fold still afforded the desired product **3aa** in good yields, which is highly important in the synthetic application, albeit the longer reaction time or higher loading of Cs_2_CO_3_ was required. Investigation of the electronic effects showed that the electron-donating or -withdrawing groups on the aryl ring did not significantly influence the reaction (**3aa**–**3ra**). A broad functionality tolerance was demonstrated for aryl aldehydes bearing different substituents, including cyano (**3la**–**3ma**) and ester groups (**3na**). The conversion was not quite sensitive to steric effects, given that high yields were obtained for products **3ea**, **3ka** and **3oa**. It is noteworthy that the enolizable aldehydes were also converted into the expected products in good yields (**3ta** and **3ua**) without self-aldol condensation.

### Substrate scope for phenylation of ketones

Although ketone is less reactive than aldehyde, the phenylation of ketones with salt **2a** still proceeded smoothly to give the desired products (Table 3). However, increased loading of salt **2a** and caesium carbonate to 4 and 4.5 equivalents, respectively, was necessary to achieve good yields. Both aromatic (**5a**–**5l**) and aliphatic ketones (**5m** and **5n**) were reactive under the optimal conditions. The successful conversion of hindered ketones indicates that the reaction is moderately tolerant to steric effects (**5f**–**5h**, **5j**). In addition, no reaction at the carbonyl-activated ester group of **5k** suggests that the transformation shows high chemoselectivity.

Compared with the traditional arylation of carbonyl groups with arylmetallic reagents, which are strong nucleophiles and can lead to the arylation of cyano^38^ and ester^12^ groups, this mild reaction clearly exhibits a higher level of functional group tolerance. More importantly, the reaction is applicable to enolizable aldehydes and ketones, indicating that the *in situ*-generated phenyl-anion equivalent exhibits high nucleophilicity and low basicity.

### Substrate scope for phenylation of imines

The successful conversion of aldehydes and ketones prompted us to examine the phenylation of imines with salt **2a** (Table 4). Imines containing electron-donating groups on the phenyl ring were effectively converted into the desired products, but for imines with a strong electron-withdrawing substituent such as cyano, the reaction failed to give the desired product. It is likely that under these reaction conditions, the imine is prone to hydrolysis to give the aldehyde, which would then react with salt **2a** to afford the alcohol. In contrast to tosyl-protected imines (*N*-Ts), *p*-methoxyphenyl-protected imine (*N*-PMP) is inert under the reaction conditions.

### The investigation of the reactivity of phosphonium salts

The reactivities of different tetraarylphosphonium salts were also investigated. Compared with tetraphenylphosphonium iodide (**3aa**, Table 2), tetra-*p*-methoxyphenylphosphonium iodide (Table 5, entry 1) and tetra-*p*-methylphenylphosphonium iodide (entry 2) showed lower reactivity towards arylation and gave the corresponding arylation products in lower yields. This is probably because the *in situ*-generated aryl-anion equivalent containing an electron-donating group is less stable and would readily abstract a proton from the trace amount of water present.

To this point of our study, the four aryl groups on the phosphorus were identical. It will be interested to know the different leaving ability when one aryl group was different from the others. To address this issue, a variety of aryl triphenylphosphonium salts were prepared and tested for the arylation of aldehyde **1a** (Table 5, entries 3–9). If the aryl moiety contained a weak electron-withdrawing group, the corresponding arylation product was obtained in low yield (entry 3), with simultaneous formation of more phenylation product **3aa** (43% yield). This might be because the leaving ability between the phenyl and aryl group is similar owing to their comparable electron-withdrawing effect, and three phenyl groups compete with one aryl group, thus leading to more phenylation product **3aa**. However, if the aryl moiety was substituted with a strong electron-withdrawing group, the phenylation product **3aa** would be greatly suppressed and the arylation became the main reaction, indicating that electron-withdrawing ability has an important role in the selectivity (entries 4–7). In the case of **3ai**, the low yield was not because of the phenylation reaction to produce **3aa**, but because of a side reaction to give 4′-phenylacetophenone (27% yield based on aldehyde; entry 8). Pyridyl triphenylphosphonium iodide was also suitable for the desired pyridylation (entry 9).

In the case of the salts containing strong electron-withdrawing groups (**2e′**–**2i′**), it would be better to change the salt anion from iodide to bis(trifluoromethanesulfonyl)imide anion (Tf_2_N^−^) and the procedure for arylation should be modified accordingly. For example, the *in situ*-generated aryl-anion equivalent would readily attack the phosphonium salt to give the diaryl by-product (33% based on aldehyde) because of the increased electrophilicity of the salt. To avoid this problem, the phosphonium salts were added slowly to the reaction to keep the substrate aldehyde in constant excess. And this required the salts to be soluble in tetrahydrofuran (THF) so as to realize the addition of the salts via syringe. The phosphonium salts containing Tf_2_N^−^ were soluble in THF and the corresponding arylation proceeded smoothly by the addition of the phosphonium salt solution into the reaction (Table 5, entries 4–8).

### Mechanistic investigation

**Figure 2:**
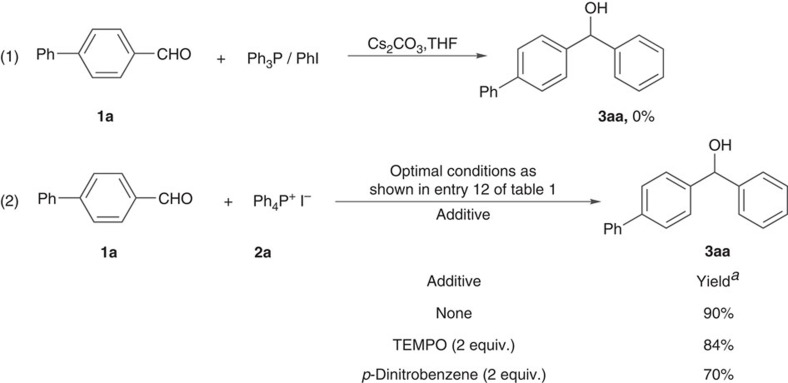
Mechanistic experiments. (1) The use of Ph_3_P/PhI system instead of salt **2a** failed to convert aldehyde **1a** into product **3aa**. (2) Neither radical scavenger TEMPO nor single-electron-transfer inhibitor *p*-dinitrobenzene can obviously suppress the phenylation reaction. ^*a*^Isolated yields.

As for the reaction mechanism, the analysis of trace transition metal in phosphonium salts and caesium carbonate by ICP spectrometry (Supplementary Table 1) revealed that this arylation is a transition-metal-free reaction. Although salt **2a** is quite stable, an equilibrium might be established between this salt and Ph_3_P/PhI under the phenylation conditions. But the use of Ph_3_P/PhI system instead of salt **2a** for the phenylation of aldehyde **1a** failed to give the desired product at all (equation 1 Fig. 2), meaning that the equilibrium cannot account for the arylation. Neither radical scavenger TEMPO [(2,2,6,6-tetramethylpiperidin-1-yl)oxyl] nor single-electron-transfer inhibitor *p*-dinitrobenzene can dramatically suppress the phenylation of aldehyde **1a** with salt **2a**, indicating that the arylation may not proceed via a single-electron-transfer mechanism (equation 2, Fig. 2). Nothing happened while refluxing aldehyde **1a**/salt **2a** system or aldehyde **1a**/Cs_2_CO_3_ system in THF. But the full conversion of salt **2a** into Ph_3_PO was observed by refluxing salt **2a** and Cs_2_CO_3_ in THF, suggesting that the interaction between salt **2a** and Cs_2_CO_3_ initiates this arylation reaction.

**Figure 3:**
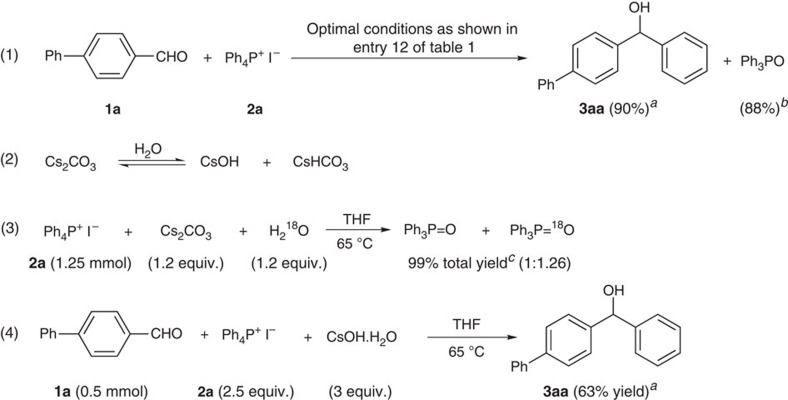
Evidence to support the path via alkaline hydrolysis. (1) Ph_3_PO was isolated as a by-product for phenylation reaction. ^*a*^Isolated yield based on **1a**; ^*b*^Isolated yield based on **2a**; (2) An equilibrium between Cs_2_CO_3_ and CsOH may be established under the reaction conditions. (3) The presence of H_2_^18^O led to the formation of Ph_3_P^18^O. ^*c*^Isolated yield. (4) CsOH can also initiate the phenylation reaction.

The alkaline hydrolysis of phosphonium salts has been well-studied for the past decades^30^,^39^,^40^,^41^,^42^,^43^,^44^,^45^,^46^,^47^. As a third-order reaction (first order with respect to phosphonium salt, second order to hydroxide)^40^,^41^, the alkaline hydrolysis of tetraphenylphosphonium salt would lead to the formation of triphenylphosphine oxide (Ph_3_PO) (refs 46, 48). Since Ph_3_PO was detected by gas chromatography mass spectrometry (GC–MS) in every phenylation reaction system using **2a** (Ph_4_P^+^ I^−^) and Ph_3_PO was isolated in high yield for the phenylation of substrate **1a** (equation 1, Fig. 3), it is reasonable to conceive that the arylation might proceed via this hydrolysis process. Although the solvent THF purchased from commercial source was extra dry (‘Extra Dry over Molecular Sieve',<0.005% water content) and the arylation was performed under N_2_ atmosphere, trace amount of water may still be unavoidable in the reaction system, thus leading to the hydrolysis of caesium carbonate (equation 2). The resulting hydroxide anion would decompose the phosphonium salt to generate Ph_3_PO, suggesting that the oxygen in Ph_3_PO could partially come from water. Indeed, the reaction of phosphonium salt with caesium carbonate in the presence of H_2_^18^O gave both Ph_3_PO and Ph_3_P^18^O (equation 3). The molar ratio of Ph_3_PO: Ph_3_P^18^O determined by ^31^P NMR was 1:1.26. This ratio was consistent with elemental analysis of the mixture (see Supplementary Methods for the formation of Ph_3_P^18^O). It seems that this hydrolysis process might be involved in the arylation, which was further supported by the observation that the direct use of caesium hydroxide instead of caesium carbonate also gave the expected product in 63% yield (equation 4). Compared with the 90% yield while using Cs_2_CO_3_, lower yield might result from the presence of hydrated water in CsOH (note: anhydrous CsOH is not commercially available). Although water can promote the hydrolysis of Cs_2_CO_3_ to produce CsOH, excessive water would suppress the subsequent arylation through the capture of the *in situ*-generated phenyl nucleophile. In addition, this possible hydrolysis process of Cs_2_CO_3_ could also explain why moderate yield (49%) of phenylation product could be obtained with the deliberate addition of water (entry 14 of Table 1).

**Figure 4:**
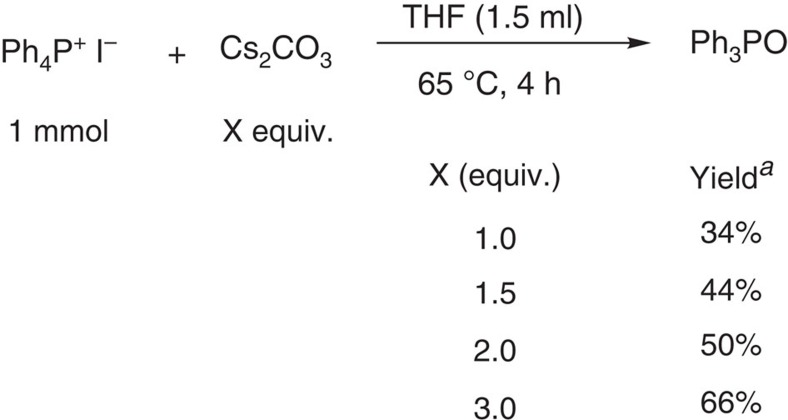
Evidence to support the Cs_2_CO_3_ promoted arylation without water. The more Cs_2_CO_3_ was used for the reaction of Ph_4_P^+^ I^−^ with Cs_2_CO_3_ for the same period of time, the more Ph_3_PO was produced, ^*a*^yields were determined by ^31^P NMR.

Nevertheless, all reagents including highly dry Cs_2_CO_3_ (99.994% purity, metals basis) and the solvent THF (<0.005% water content) were stored in a glove box, and the arylation were conducted under N_2_ atmosphere, suggesting that the water content in the reaction system should be extremely low. This trace amount of water cannot cause the complete hydrolysis of Cs_2_CO_3_, indicating that another reaction process should exist. As shown in Table 2, for the large-scale phenylation to give **3aa**, significantly less reaction time (12 h versus 120 h) was needed to achieve comparable yield (75% versus 84%) with the use of more Cs_2_CO_3_ (5 equivalents versus 3 equivalents). The water contents (trace) in both reaction systems should be the same since these reactions were conducted under the same reaction conditions. The faster arylation with the use of more Cs_2_CO_3_ means that Cs_2_CO_3_ may be able to promote the arylation without the involvement of water. If this is the case, more Cs_2_CO_3_ would result in a faster conversion of Ph_4_P^+^ I^−^ into Ph_3_PO. Indeed, for the reaction of Ph_4_P^+^ I^−^ with Cs_2_CO_3_ in THF for 4 h, the more Cs_2_CO_3_ was used, the more Ph_3_PO was produced (note: Ph_4_P^+^ I^−^ was not completely consumed in every reaction; Fig. 4). These results indicate that Cs_2_CO_3_, for the most part, directly promoted this arylation.

Apparently, nucleophilic phenyl species was formed in the process of the arylation. Salt **2a**, also as an electrophilic species, might be able to trap this nucleophilic phenyl group to give Ph_5_P (ref. 49). To ascertain whether Ph_5_P is generated or not, more evidences were collected. ^31^P NMR measurement of the reaction of **1a** with salt **2a** only detected the formation of Ph_3_PO. Furthermore, instead of the phosphonium salt **2a**, Ph_5_P was directly used in its reaction with aldehyde (See Supplementary Methods for the procedure for arylation of **1a** with Ph_5_P)^50^,^51^. However, no desired product **3aa** was detected without the presence of Cs_2_CO_3_. And even in the presence of Cs_2_CO_3_, the yield of **3aa** was very low (11%), indicating that Ph_5_P may not be involved in the above arylation.

**Figure 5:**
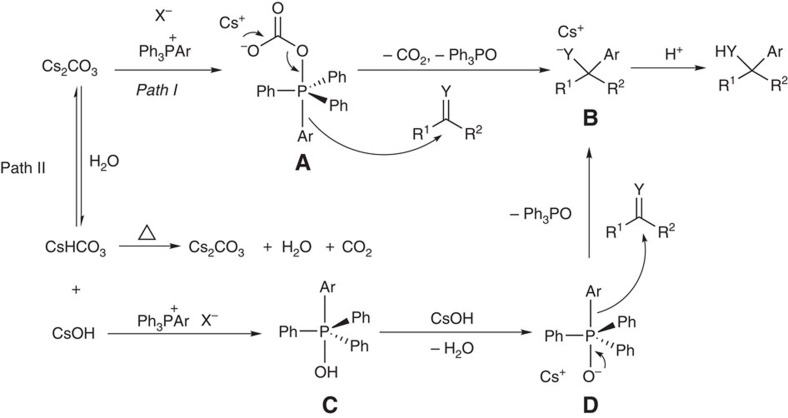
Proposed nucleophilic arylation mechanism. The reaction should be predominantly initiated by Cs_2_CO_3_ (path I), and may also be promoted by CsOH due to the trace amount of water present in the reaction system (path II).

On the basis of the above results, we proposed a plausible mechanism shown in Fig. 5. The direct attack of Cs_2_CO_3_ at the phosphonium cation is the predominant path (Path I). Nucleophilic addition to the positive phosphorus would always be along the axial co-ordinate and pseudorotation would occur afterwards to place the electronegative substituent in the other axial position^30^,^39^,^47^,^52^, generating trigonal bipyramidal tetraaryloxyphosphorane **A**. The simultaneous decarboxylation and nucleophilic attack of Ar in intermediates **A** to carbonyl or imino groups afford Ph_3_PO and the adduct **B**, which is then protonated by acid to furnish the final product. However, owing to the unavoidable presence of trace amount of water, an equilibrium between Cs_2_CO_3_ and CsHCO_3_/CsOH may be established (path II). CsHCO_3_ might gradually decompose into Cs_2_CO_3_, water and carbon dioxide at the reaction temperature. The generated CsOH would promote the subsequent arylation via an alkaline-hydrolysis path^30^,^39^,^40^,^41^,^42^,^43^,^44^,^45^,^46^,^47^. Nucleophilic addition of hydroxide to phosphorus and the subsequent pseudorotation generate intermediate **C**, deprotonation of which by CsOH gives an oxyanionic phosphorane **D**. The expulsion of the aryl anion from intermediate **D** and the nucleophilic addition of this Ar group to substrate also afford the adduct **B** and Ph_3_PO. Therefore, the two paths might coexist. It should be noted that none of the intermediate **A**, **C** or **D** was detected by ^31^P NMR, although *P*-hydroxytetraorganophosphorane has recently been observed and characterized by low-temperature NMR^53^. Considering that cyano and ester groups are stable under the optimized reaction conditions and the enolizable carbonyl compounds were successfully converted, the naked aryl anion is not believed to be formed in the arylation process. Carbon dioxide cannot be detected by GC–MS in the gas phase of the reaction system because the gas phase was full of the solvent THF. Fortunately, when DMF was used instead of THF in the reaction of aldehyde **1a** with salt **2a**, GC–MS analysis of the gas phase of the reaction mixture successfully detected the generation of CO_2_ (see ‘determination of CO_2_ by GC–MS spectroscopy' in Supplementary Methods) albeit that the desired product was isolated in only 60% yield, further supporting the proposed reaction mechanism.

## Discussion

Although phosphonium salts are electrophilic species, we found that a substituent on the positive phosphorus can act as a nucleophile for the construction of C–C bond while initiated by another appropriate nucleophile. The successful application of tetraarylphosphonium salts to the nucleophilic transition-metal-free arylation of carbonyl and imino compounds in the presence of caesium carbonate is described herein. The high P–O affinity has an important role in this arylation strategy. This practical protocol is attractive because the reaction is not particularly sensitive to water, shows wide substrate scope under mild conditions, and is compatible with a variety of functional groups. It provides a highly valuable method for C-arylation and easy access to α-aryl-alcohols and α-aryl-amines. In addition, the success of the strategy means that phosphonium salts may find applications in other areas of chemistry.

## Methods

### General procedure for phenylation of aldehydes

Under N_2_ atmosphere, the mixture of aldehyde (0.50 mmol), tetraphenylphosphonium iodide (583.0 mg, 1.25 mmol) and Cs_2_CO_3_ (488.7 mg, 1.50 mmol) in THF (4 ml) and stirred at 65 °C for 12 h. The reaction was quenched by 3 N HCl (0.5 ml). The resulting mixture was extracted with dichloromethane (DCM; 3 × 30 ml). The combined organic phase was dried over Na_2_SO_4_. After filtration, the solvent was removed by concentration, and the residue was subjected to column chromatography to afford the pure product.

### General procedure for phenylation of ketones

Under N_2_ atmosphere, the mixture of ketone (0.40 mmol), tetraphenylphosphonium iodide (745.8 mg, 1.60 mmol) and Cs_2_CO_3_ (586.4 mg, 1.80 mmol) in THF (4 ml) was stirred at 65 °C for 24 h. The reaction was quenched by 4.5 N HCl (1.5 ml). The resulting mixture was extracted with DCM (3 × 30 ml). The combined organic phase was dried over Na_2_SO_4_. After filtration, the solvent was removed by concentration, and the residue was subjected to column chromatography to afford the pure product.

### General procedure for phenylation of imines

Under N_2_ atmosphere, the mixture of imine (0.50 mmol), tetraphenylphosphonium iodide (583.0 mg, 1.25 mmol) and Cs_2_CO_3_ (488.7 mg, 1.50 mmol) in THF (4 ml) was stirred at 65 °C for 12 h. The reaction was quenched by 3 N HCl (0.5 ml). The resulting mixture was extracted with DCM (3 × 30 ml). The combined organic phase was dried over Na_2_SO_4_. After filtration, the solvent was removed by concentration. The residue was subjected to column chromatography to afford the pure product.

### Arylation of 1a with various teraarylphosphonium salts

*Method A.* Under N_2_ atmosphere, the mixture of [1,1′-biphenyl]-4-carbaldehyde (**1a**; 91.2 mg, 0.50 mmol), tetraarylphosphonium iodide (1.25 mmol) and Cs_2_CO_3_ (488.7 mg, 1.50 mmol) in THF (4 ml) was stirred at 65 °C until phosphonium salt was completely consumed as monitored by ^31^P NMR. The reaction was quenched by 3 N HCl (0.5 ml). The resulting mixture was extracted with DCM (3 × 30 ml). The combined organic phase was dried over Na_2_SO_4_, and the residue was subjected to column chromatography to afford the pure product.

*Method B*. Under N_2_ atmosphere, into the mixture of [1,1′-biphenyl]-4-carbaldehyde (**1a**; 91.2 mg, 0.50 mmol) and Cs_2_CO_3_ (488.7 mg, 1.50 mmol) in THF (2 ml) at 65 °C was added the solution of phosphonium bis(trifluoromethanesulfonyl)amide (1.25 mmol) in THF (2 ml) slowly for 6 h. On completion of addition, the reaction was stirred for another 10 min. The mixture was quenched by 3 N HCl (0.5 ml) and extracted with DCM (3 × 30 ml). The combined organic phase was dried over Na_2_SO_4_. After filtration, the solvent was removed by concentration, and the residue was subjected to column chromatography to afford the pure product.

## Additional information

**How to cite this article:** Deng, Z. *et al*. Nucleophilic arylation with tetraarylphosphonium salts. *Nat. Commun.* 7:10337 doi: 10.1038/ncomms10337 (2016).

## Supplementary Material

Supplementary Information Supplementary Figures 1-175, Supplementary Table 1, Supplementary Methods and Supplementary References

## Figures and Tables

**Figure 1:**
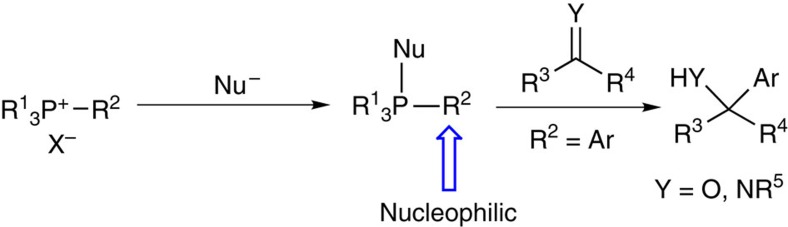
Design of arylation reaction with phosphonium salt. An aryl substituent on phosphorus can act as a nucleophile to realize the nucleophilic arylation of carbonyls and imines.

**Table 1:**
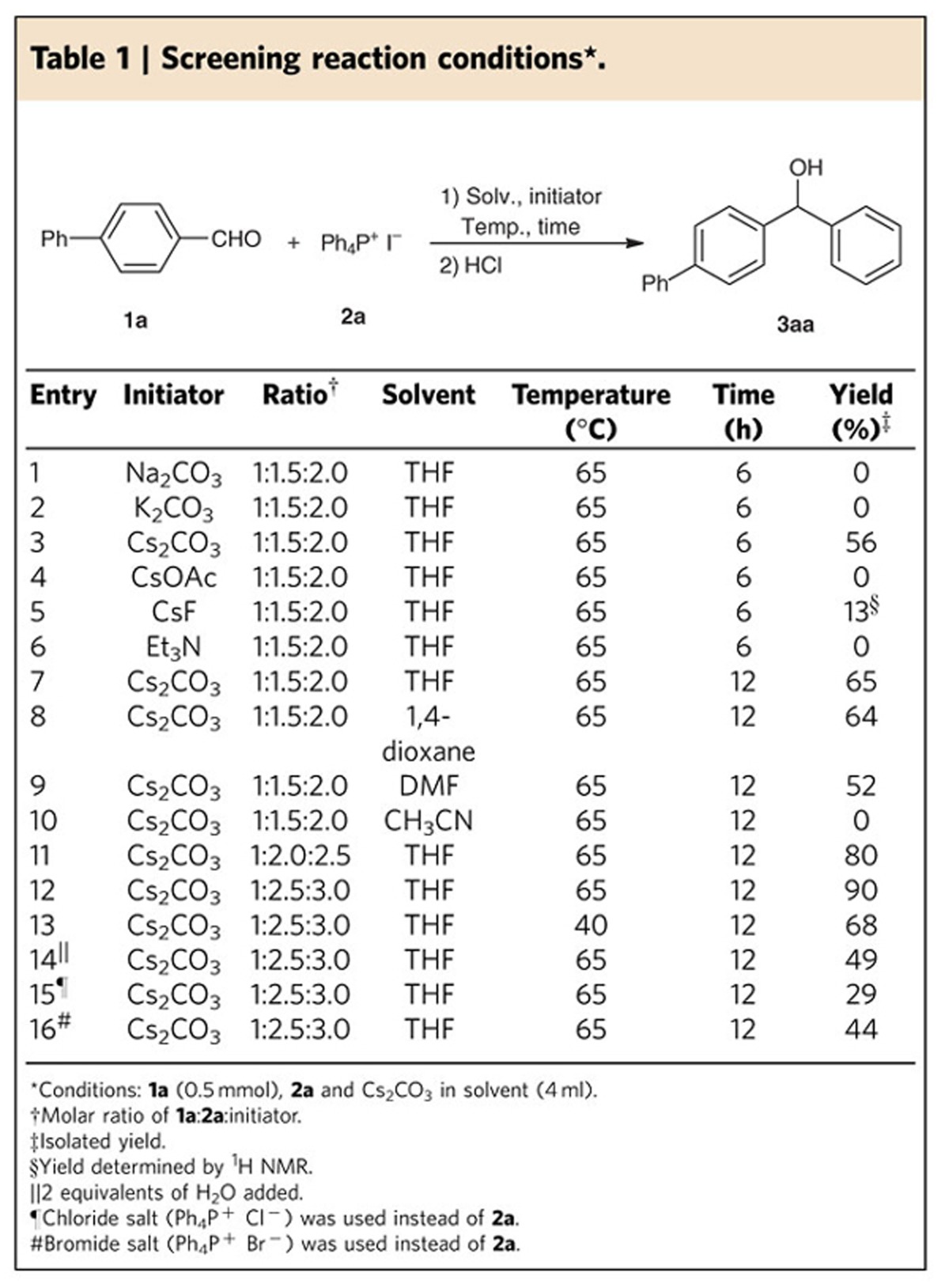
Screening reaction conditions^*^.

**Table 2:**
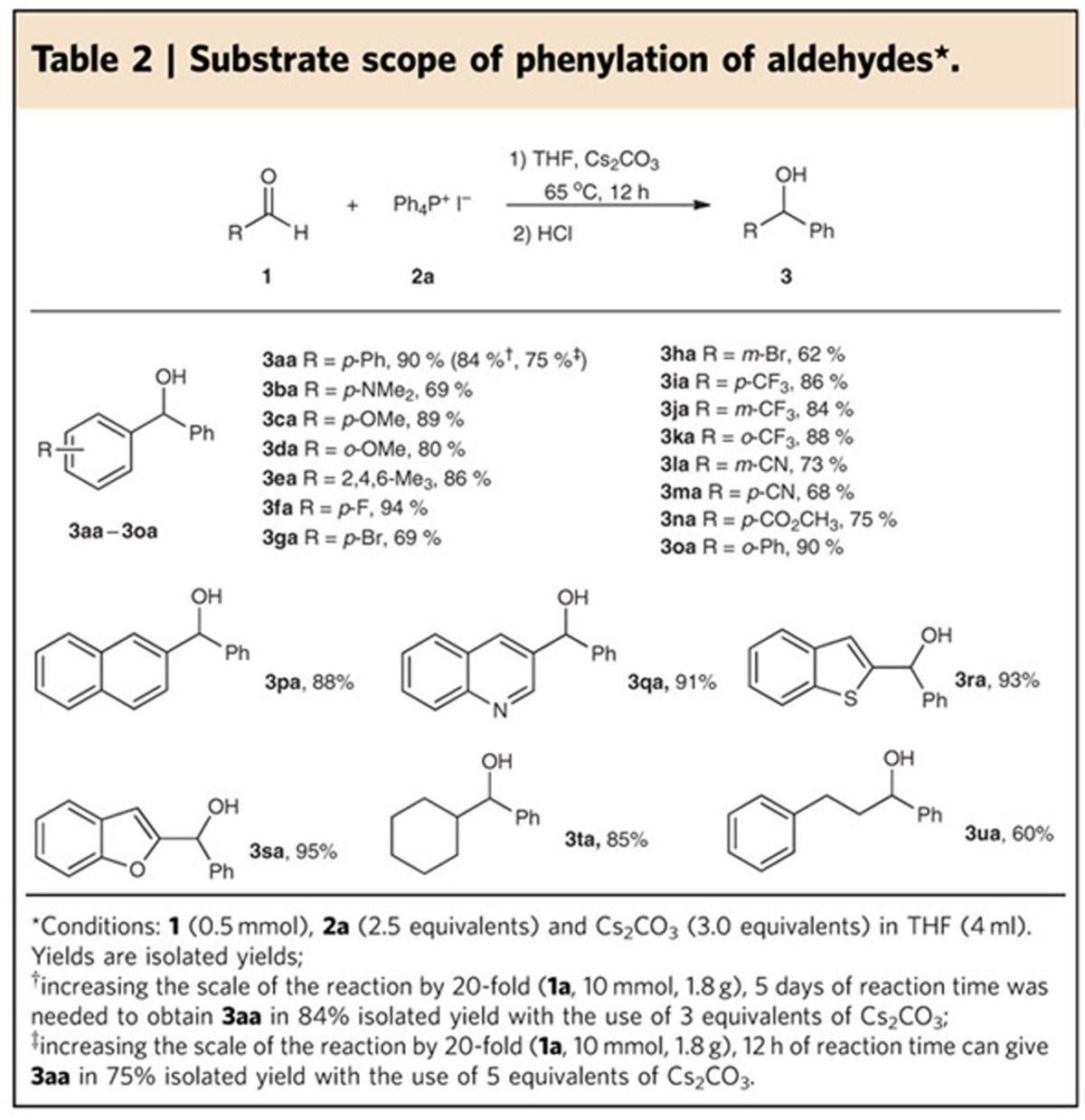
Substrate scope of phenylation of aldehydes^*^.

**Table 3:**
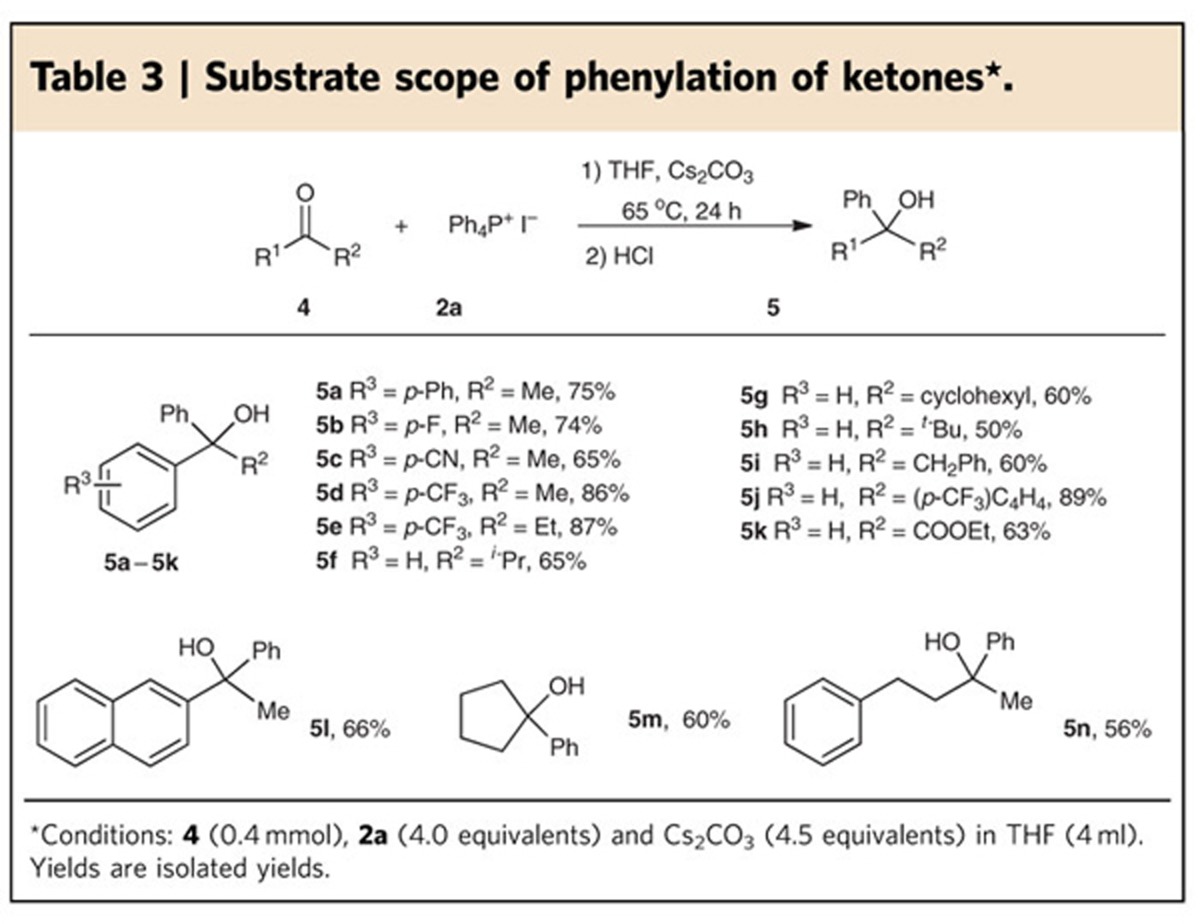
Substrate scope of phenylation of ketones^*^.

**Table 4:**
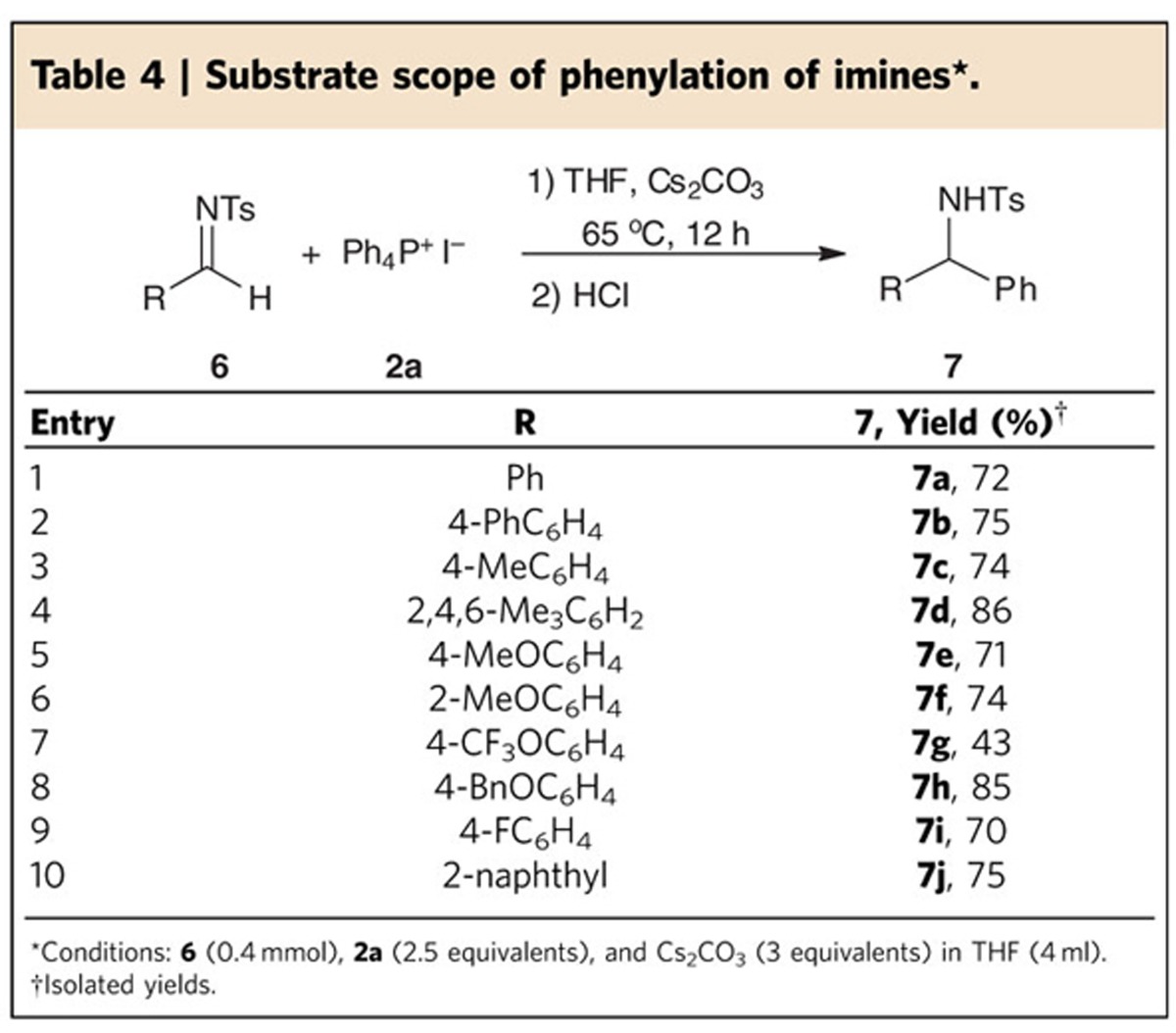
Substrate scope of phenylation of imines^*^.

**Table 5:**
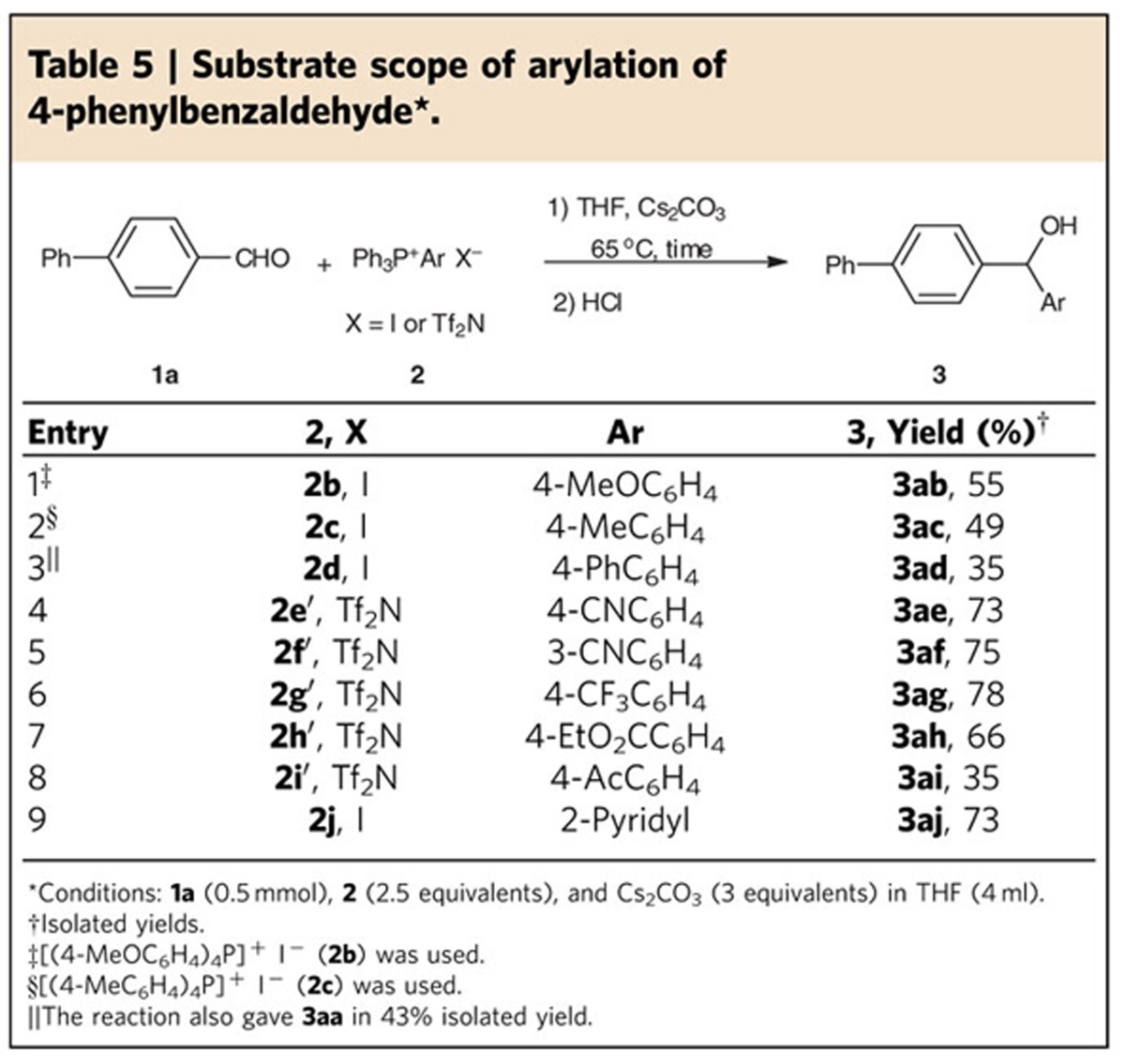
Substrate scope of arylation of 4-phenylbenzaldehyde^*^>.
