# Supplementary material for: Nucleophilic arylation with tetraarylphosphonium salts
Source: Nat Commun. 2016 Jan 29;7:10337. doi: 10.1038/ncomms10337 (PMC4740112; doi:10.1038/ncomms10337)
Supplement: Supplementary Information — Supplementary Figures 1-175, Supplementary Table 1, Supplementary Methods and Supplementary References [file ncomms10337-s1.pdf]

**Supplementary Figure 1. $^{1}$H NMR of Tetrakis(4-methoxyphenyl)phosphonium iodide 2b**

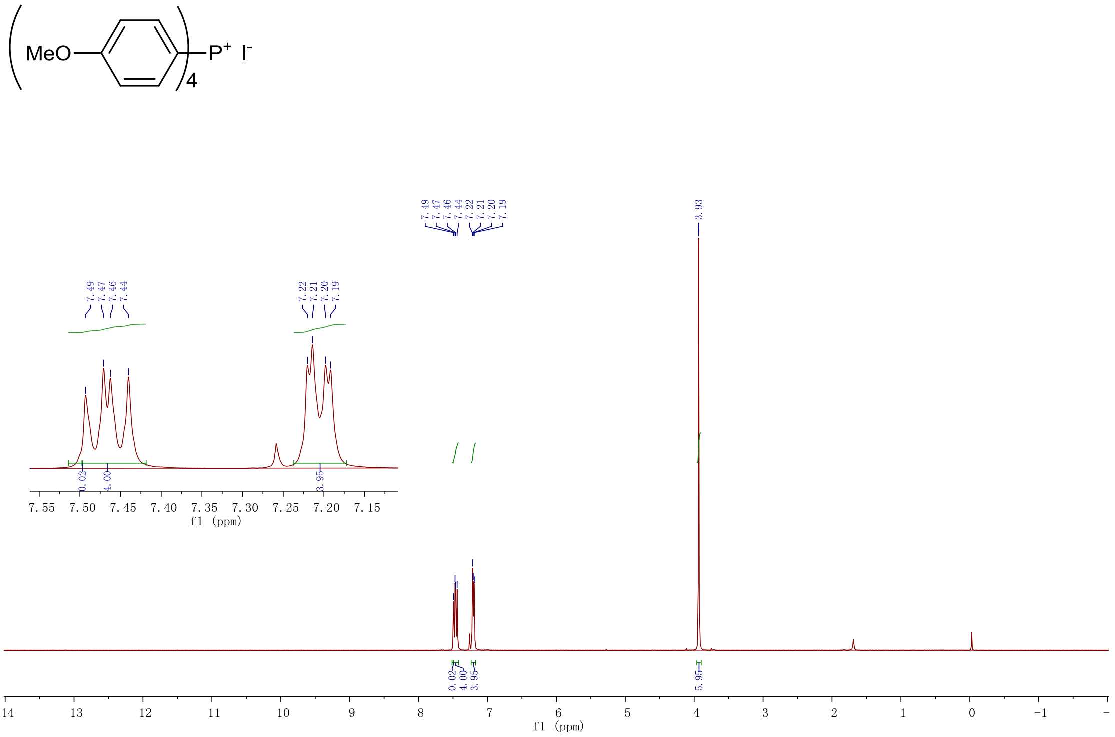


**Supplementary Figure 2. $^{31}$P NMR of Tetrakis(4-methoxyphenyl)phosphonium iodide 2b**

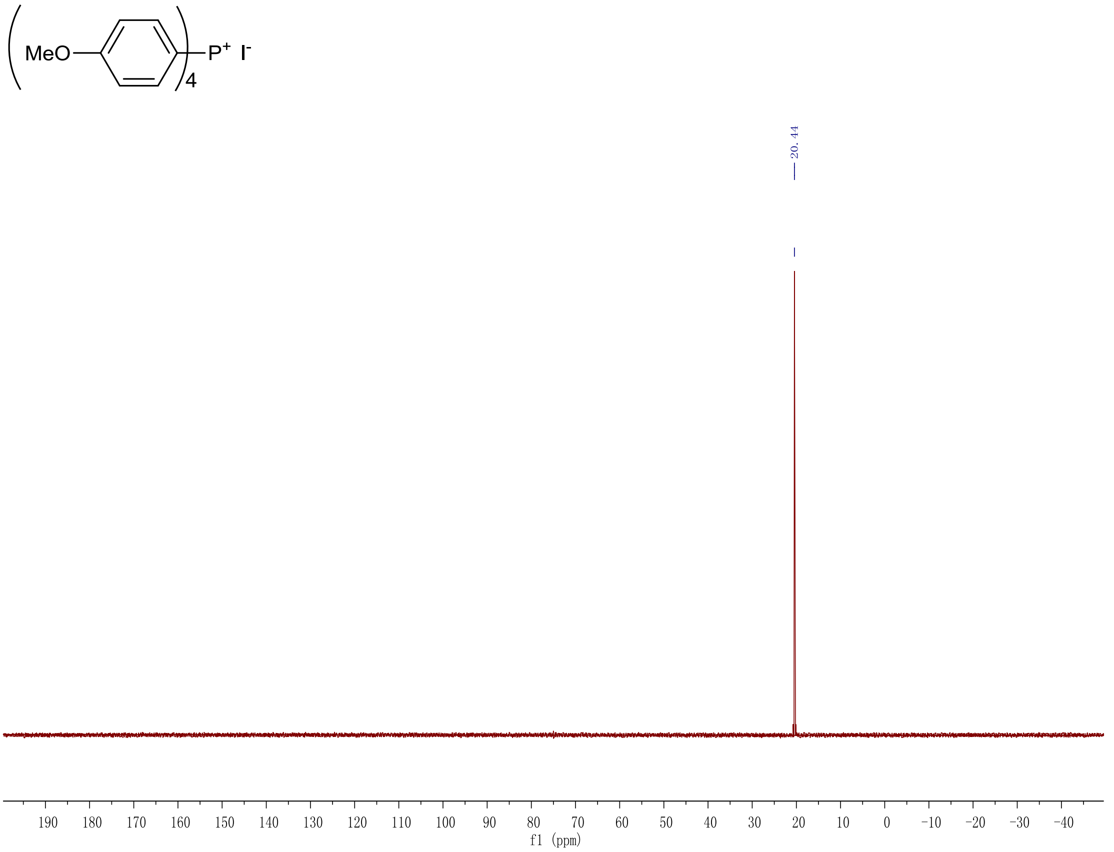

**Supplementary Figure 3. $^{13}$C NMR of Tetrakis(4-methoxyphenyl)phosphonium iodide 2b**

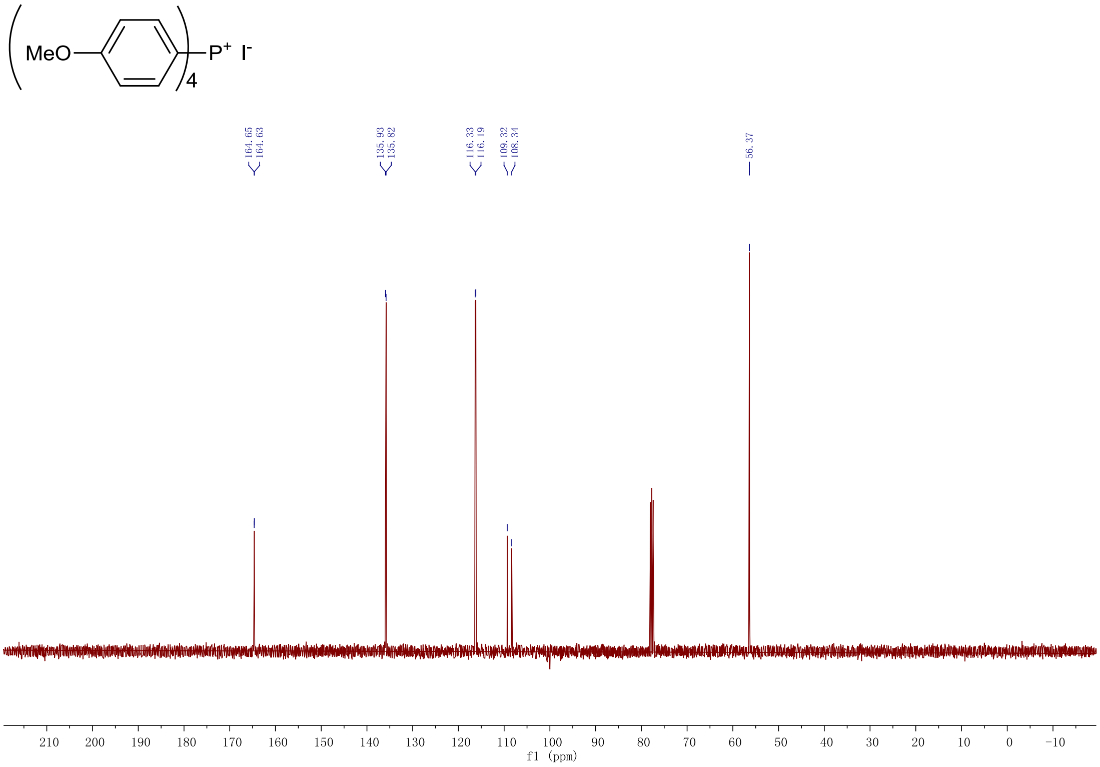


**Supplementary Figure 4. $^{1}$H NMR of Tetra-p-tolylphosphonium iodide 2c**

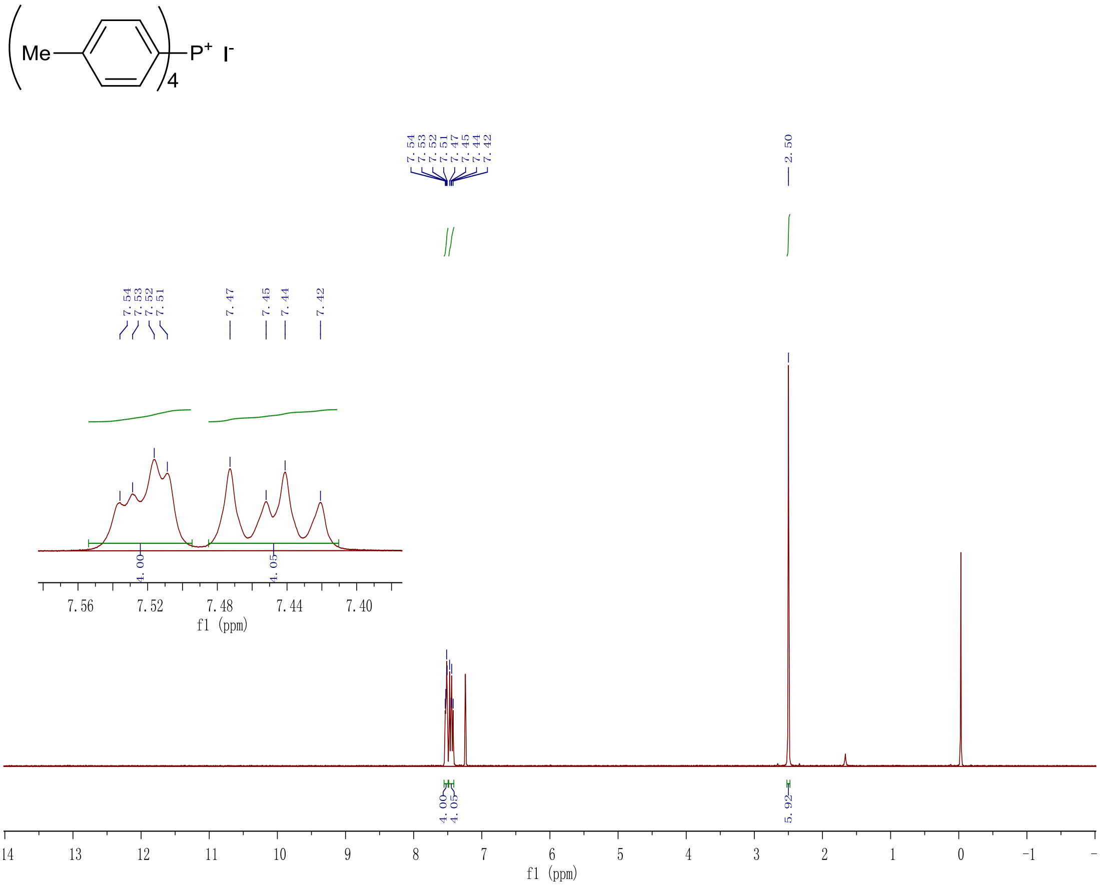

## Supplementary Figure 5. $^{31}$P NMR of Tetra-p-tolylphosphonium iodide 2c

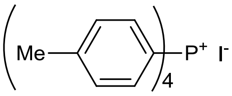


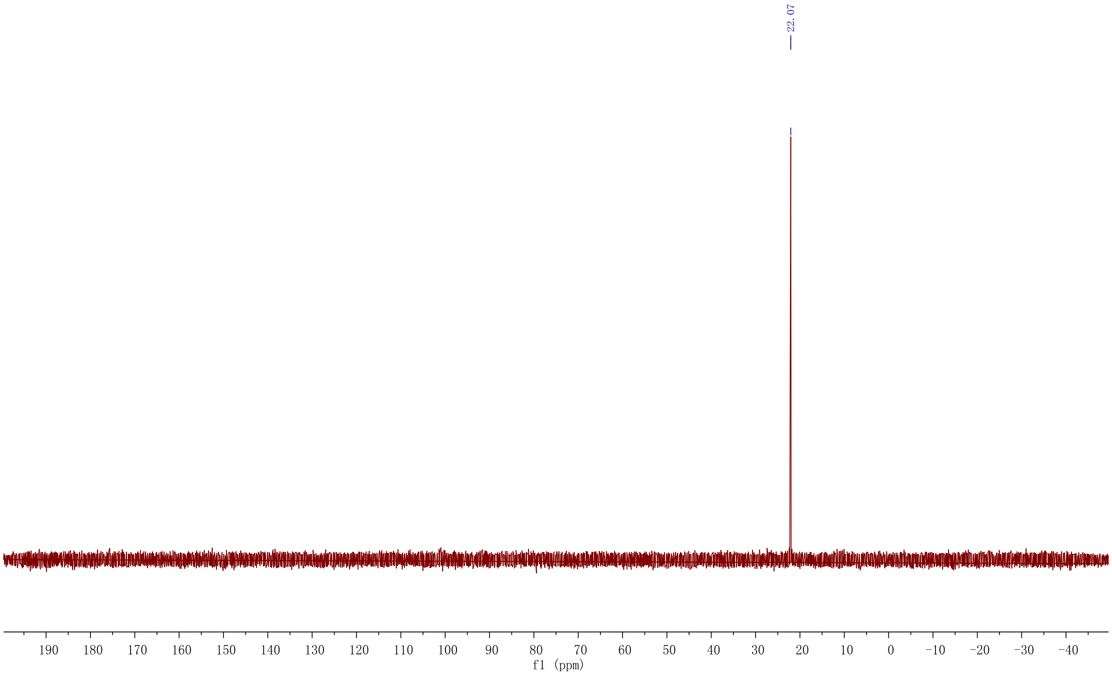


## Supplementary Figure 6. $^{13}$C NMR of Tetra-p-tolylphosphonium iodide 2c

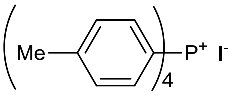


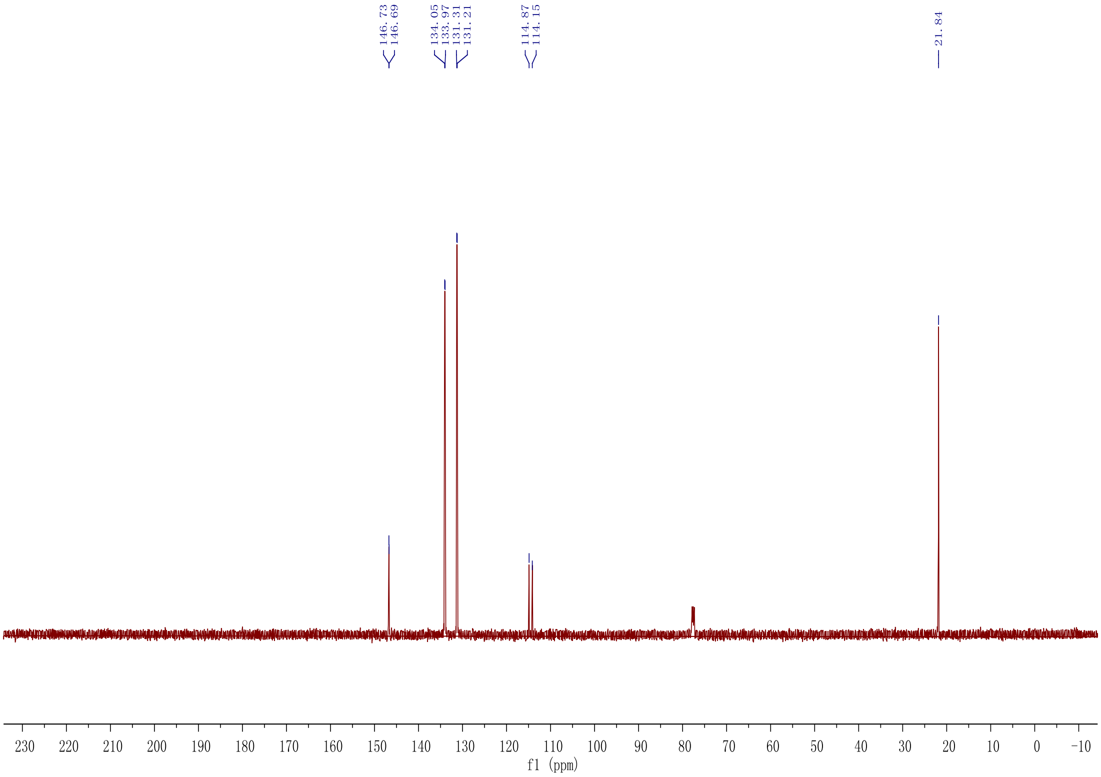

**Supplementary Figure 7. $^{1}$H NMR of [1,1'-Biphenyl]-4-yltriphenylphosphonium iodide 2d**

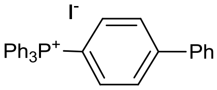


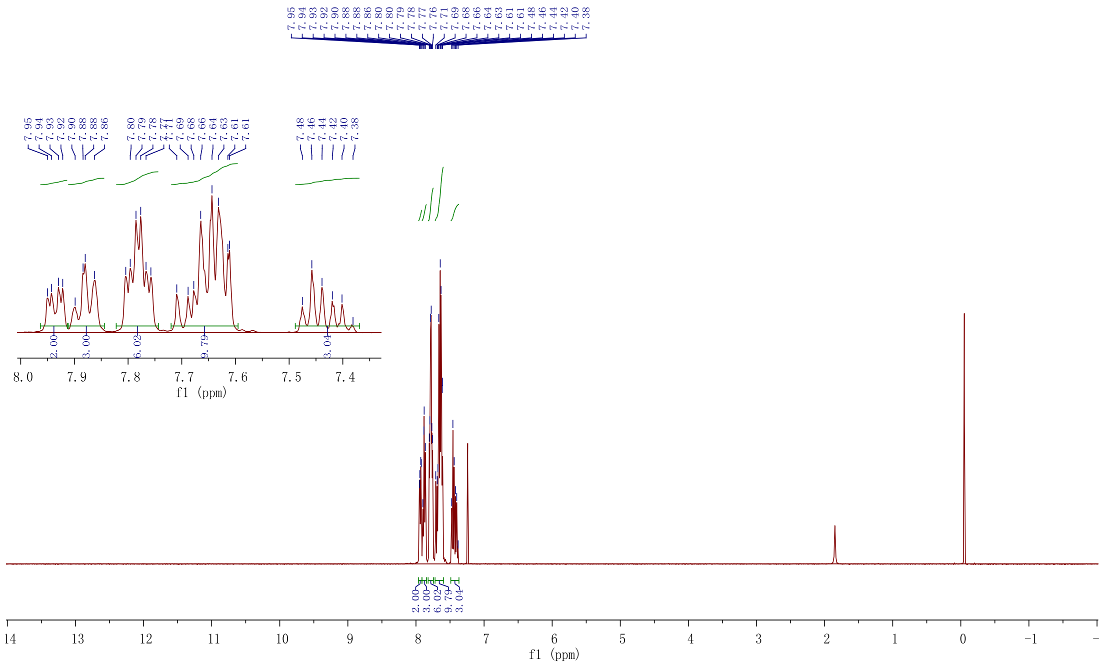

**Supplementary Figure 8. $^{31}$P NMR of [1,1'-Biphenyl]-4-yltriphenylphosphonium iodide 2d**

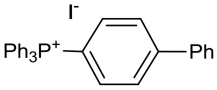


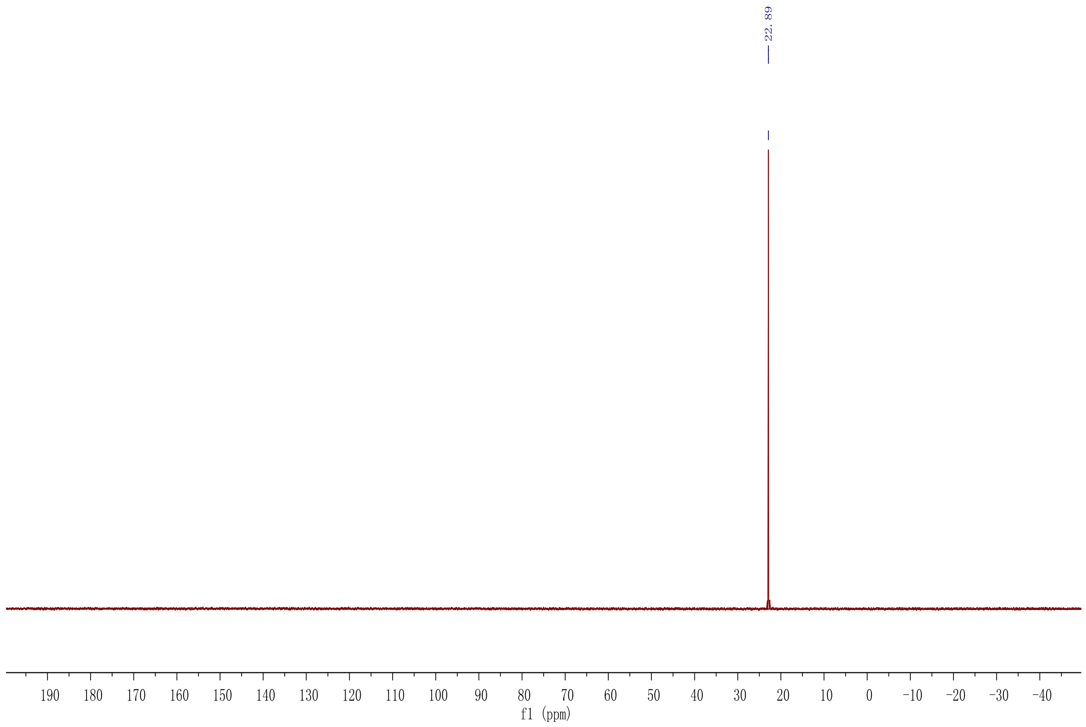

**Supplementary Figure 9. $^{1}$H NMR of (4-Cyanophenyl)triphenylphosphonium iodide 2e**

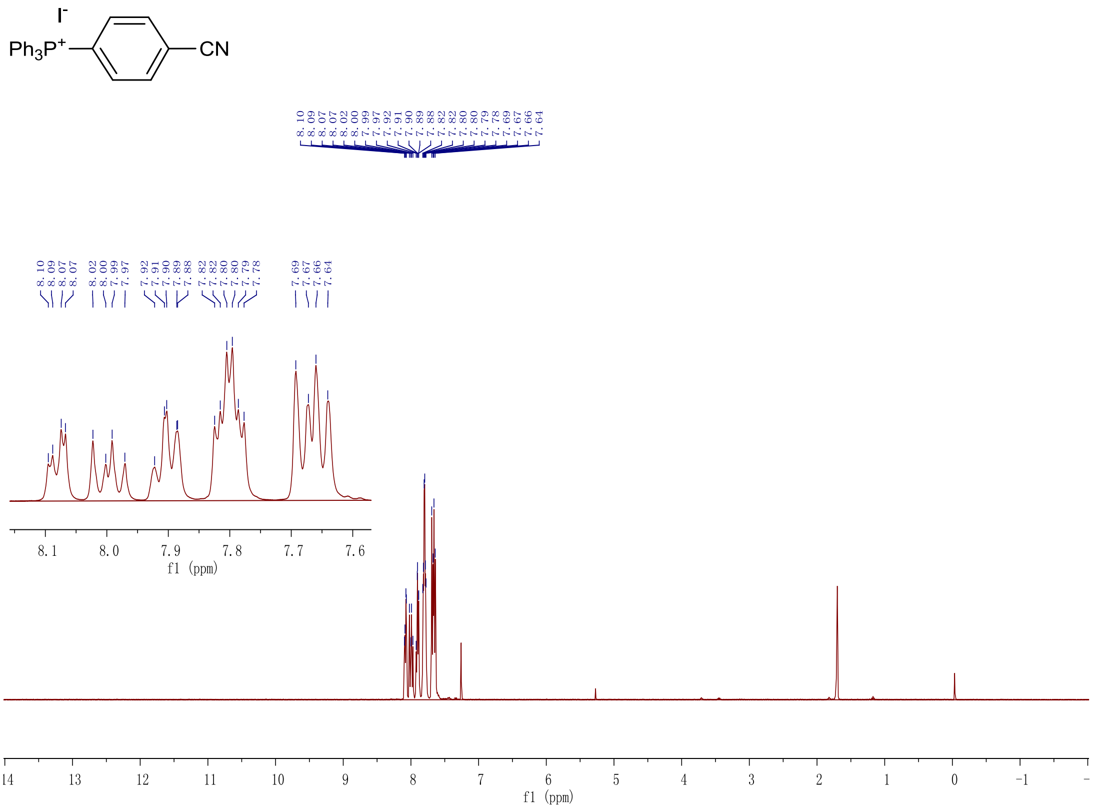


**Supplementary Figure 10. $^{31}$P NMR of (4-Cyanophenyl)triphenylphosphonium iodide 2e**

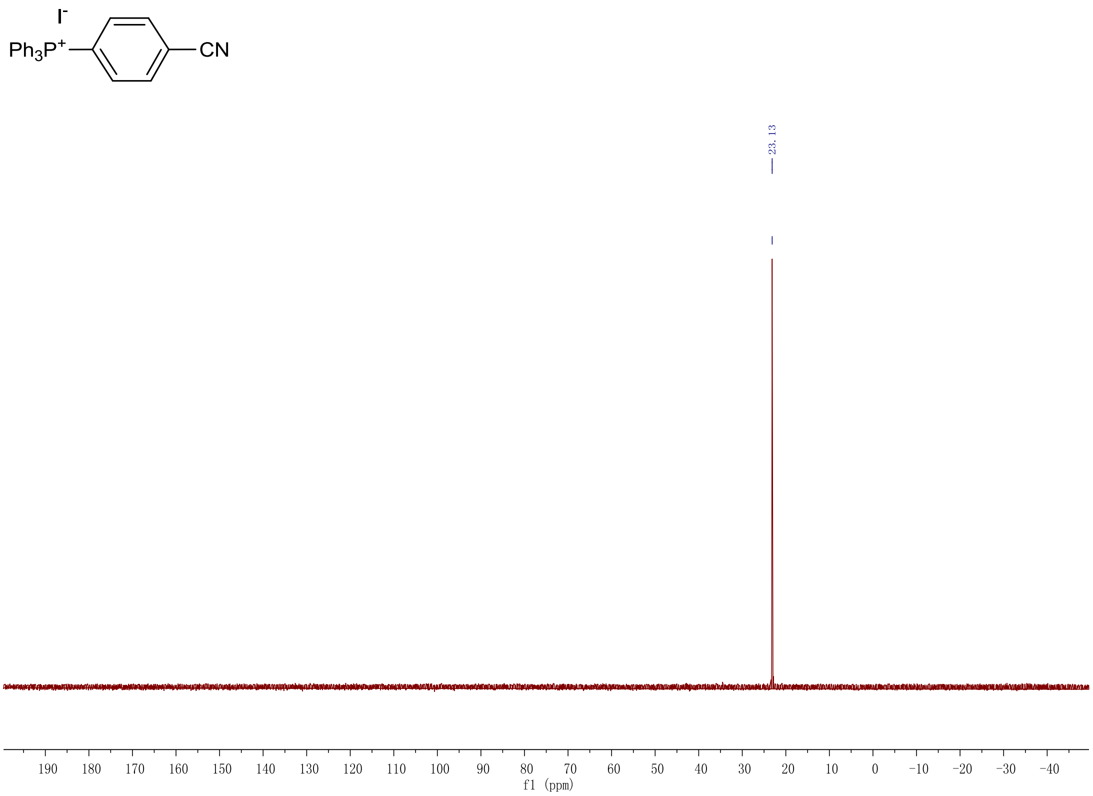

**Supplementary Figure 11. $^{13}C$ NMR of (4-Cyanophenyl)triphenylphosphonium iodide 2e**

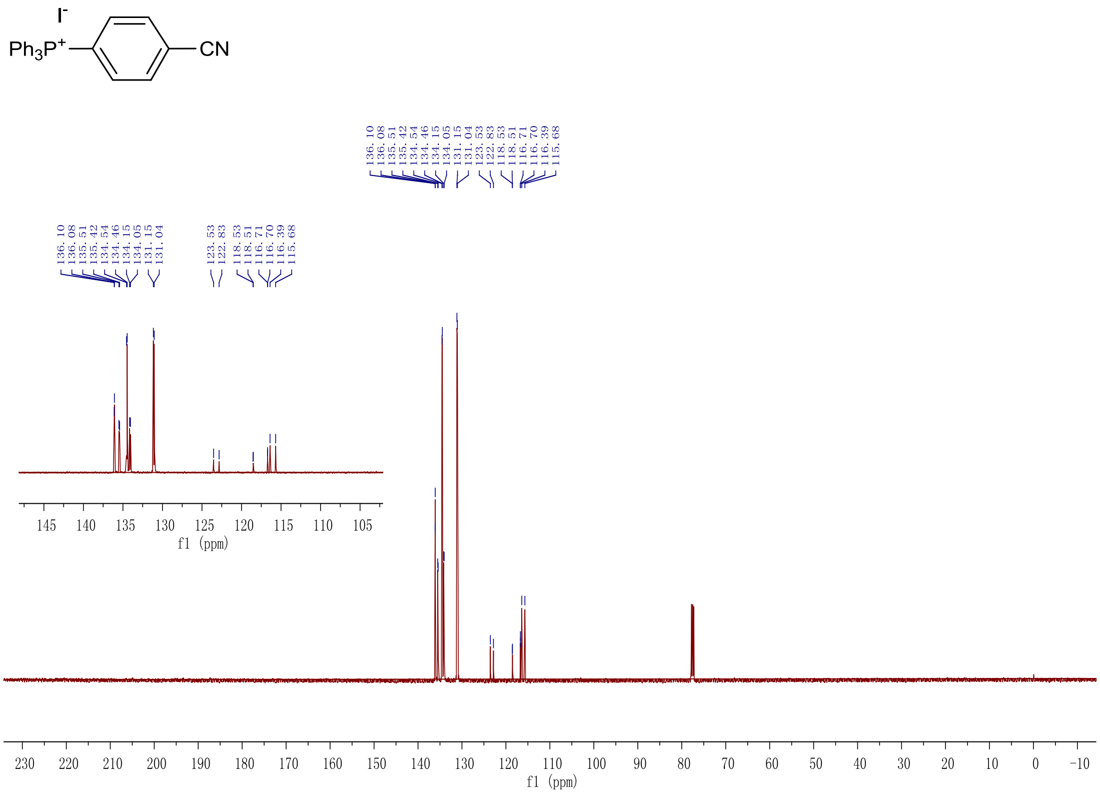


**Supplementary Figure 12. $^{1}H$ NMR of (3-Cyanophenyl)triphenylphosphonium iodide 2f**

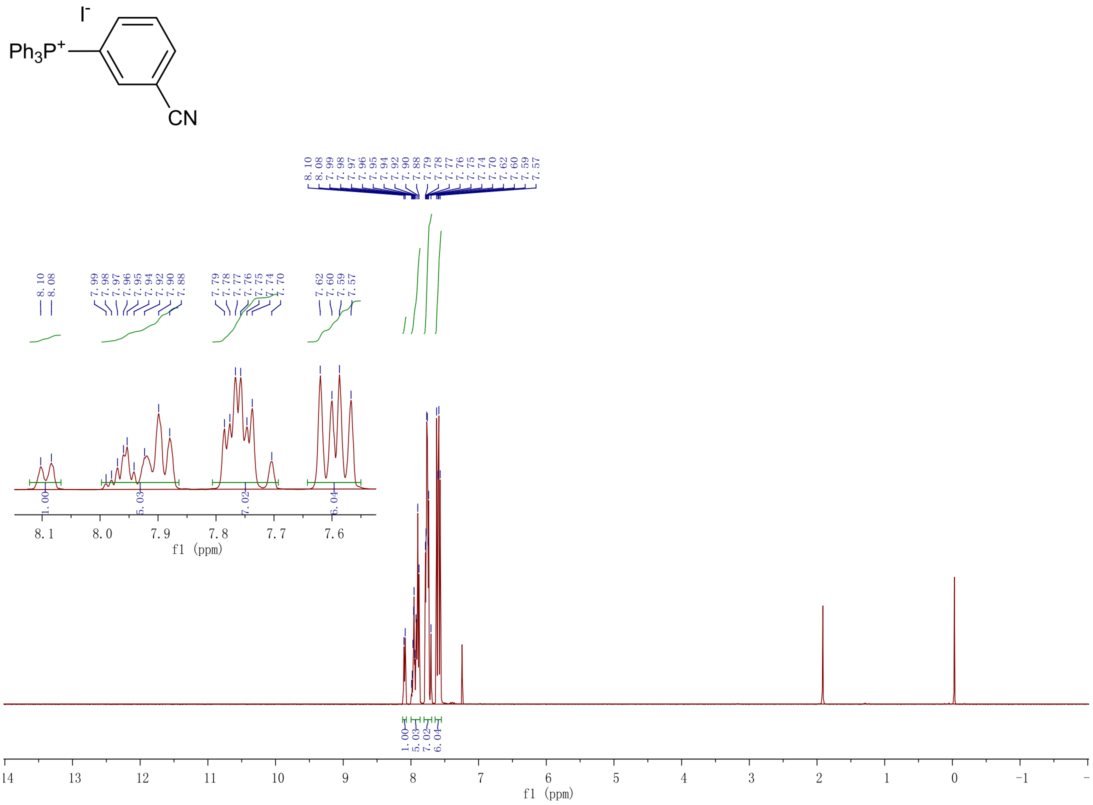

**Supplementary Figure 13. $^{31}$P NMR of (3-Cyanophenyl)triphenylphosphonium iodide 2f**

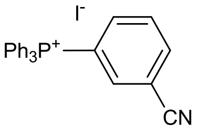


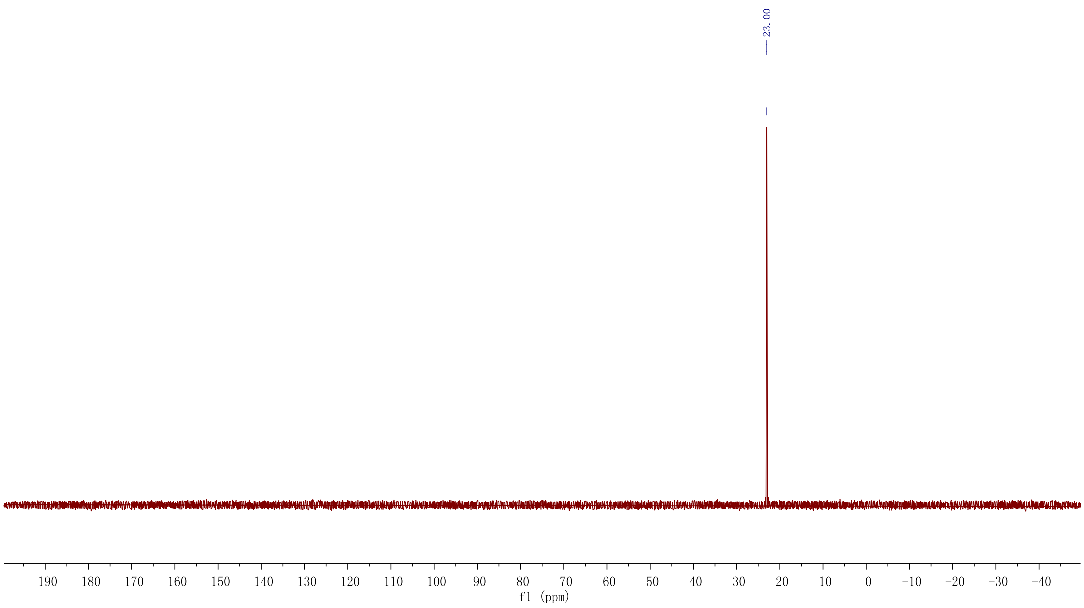


**Supplementary Figure 14. $^{13}$C NMR of (3-Cyanophenyl)triphenylphosphonium iodide 2f**

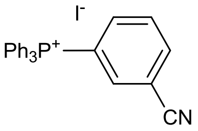


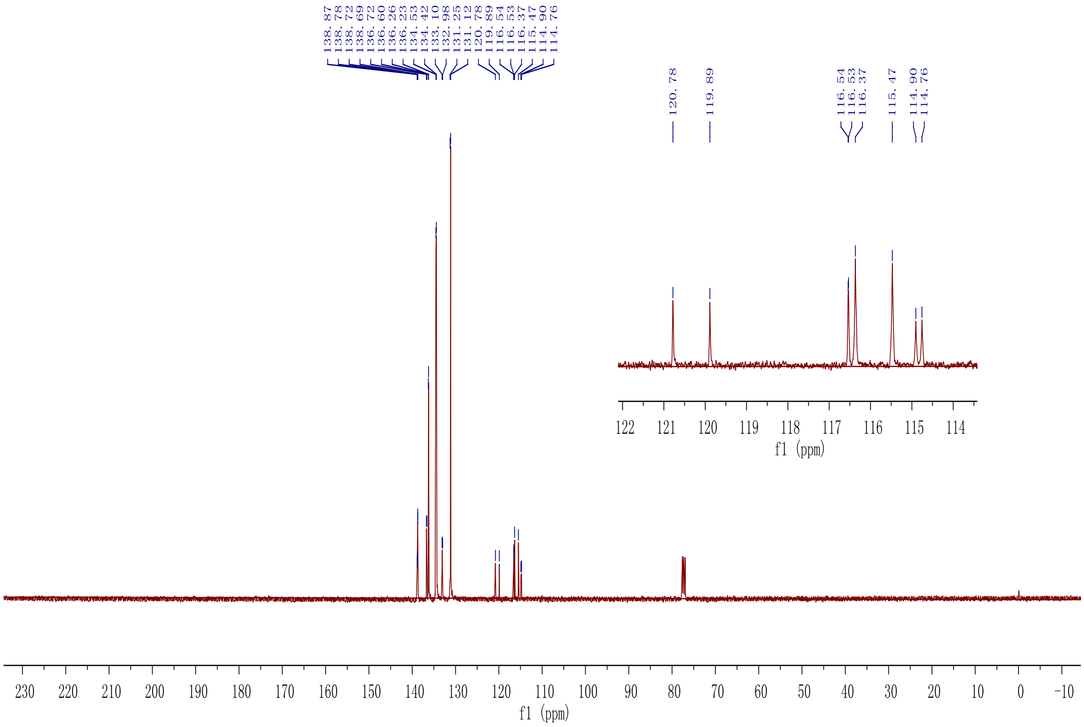

**Supplementary Figure 15. $^{1}$H NMR of Triphenyl(4-(trifluoromethyl)phenyl)-phosphonium iodide 2g**

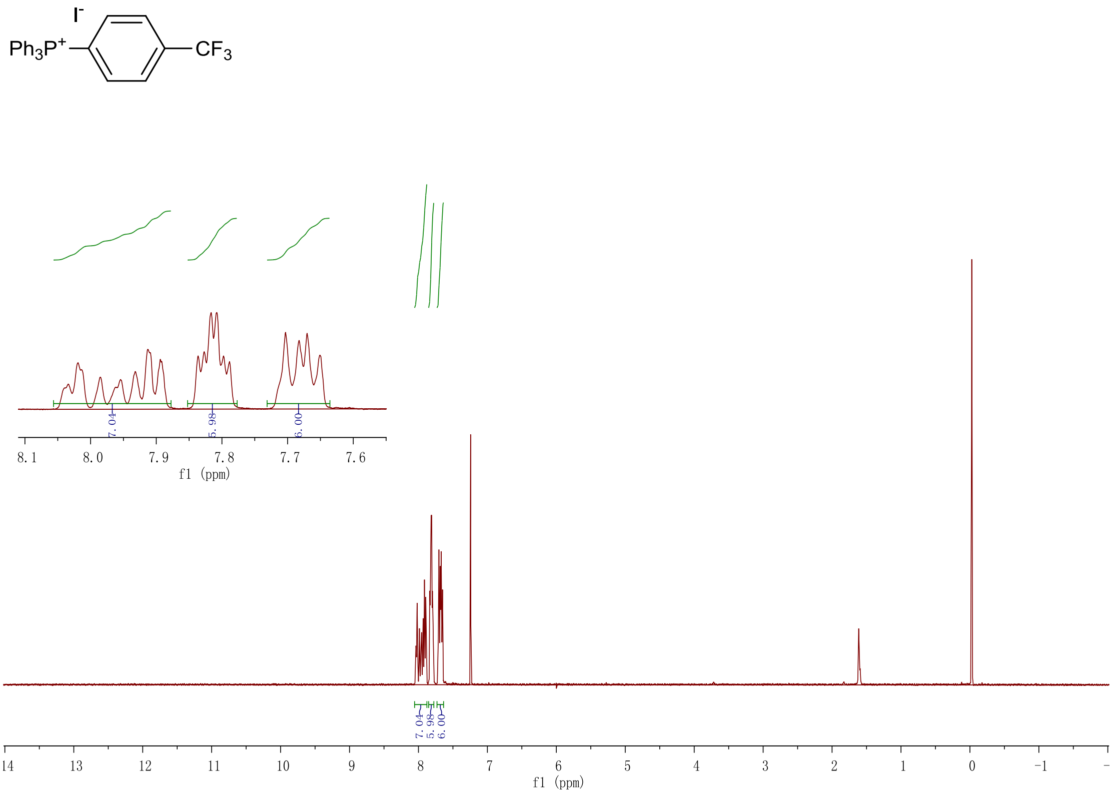


**Supplementary Figure 16. $^{19}$F NMR of Triphenyl(4-(trifluoromethyl)phenyl)-phosphonium iodide 2g**

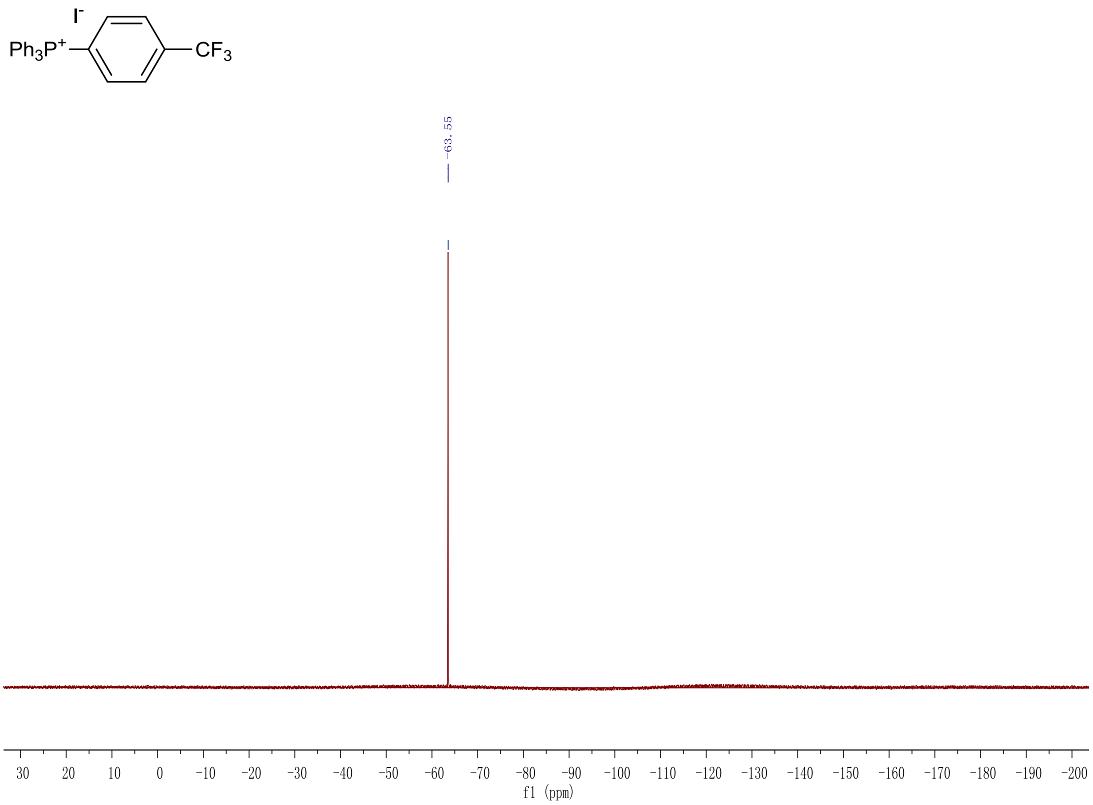

**Supplementary Figure 17. $^{31}$P NMR of Triphenyl(4-(trifluoromethyl)phenyl)-phosphonium iodide 2g**

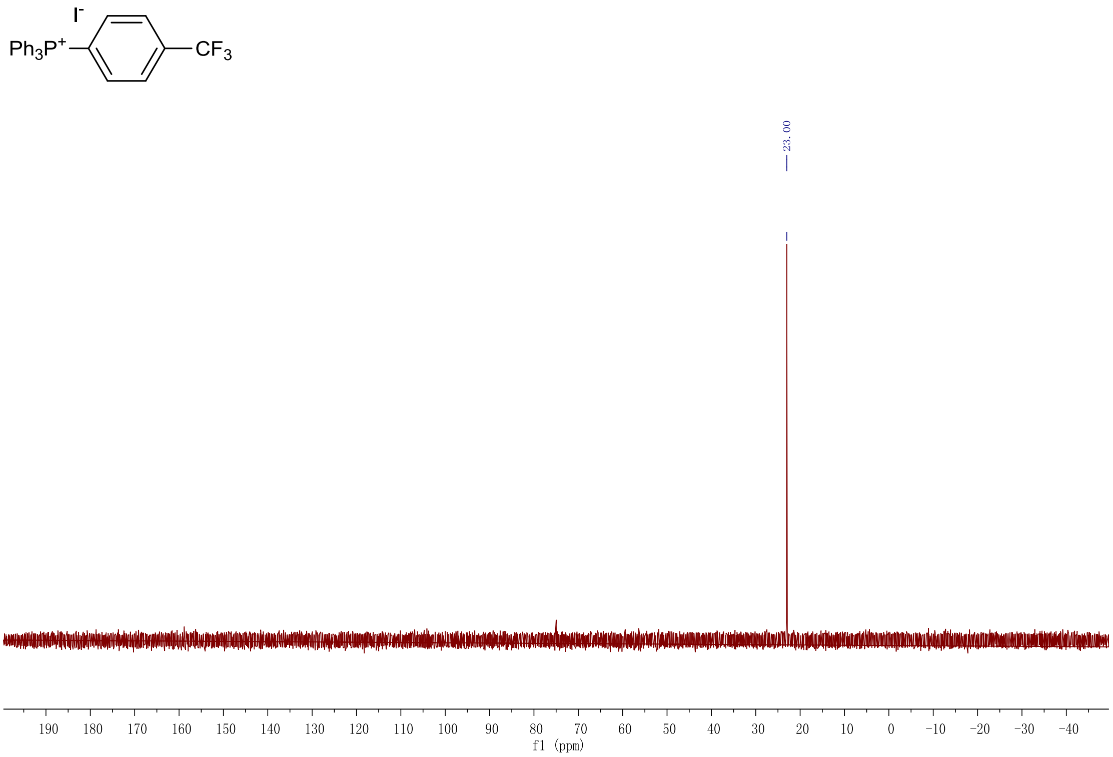


**Supplementary Figure 18. $^{13}$C NMR of Triphenyl(4-(trifluoromethyl)phenyl)-phosphonium iodide 2g**

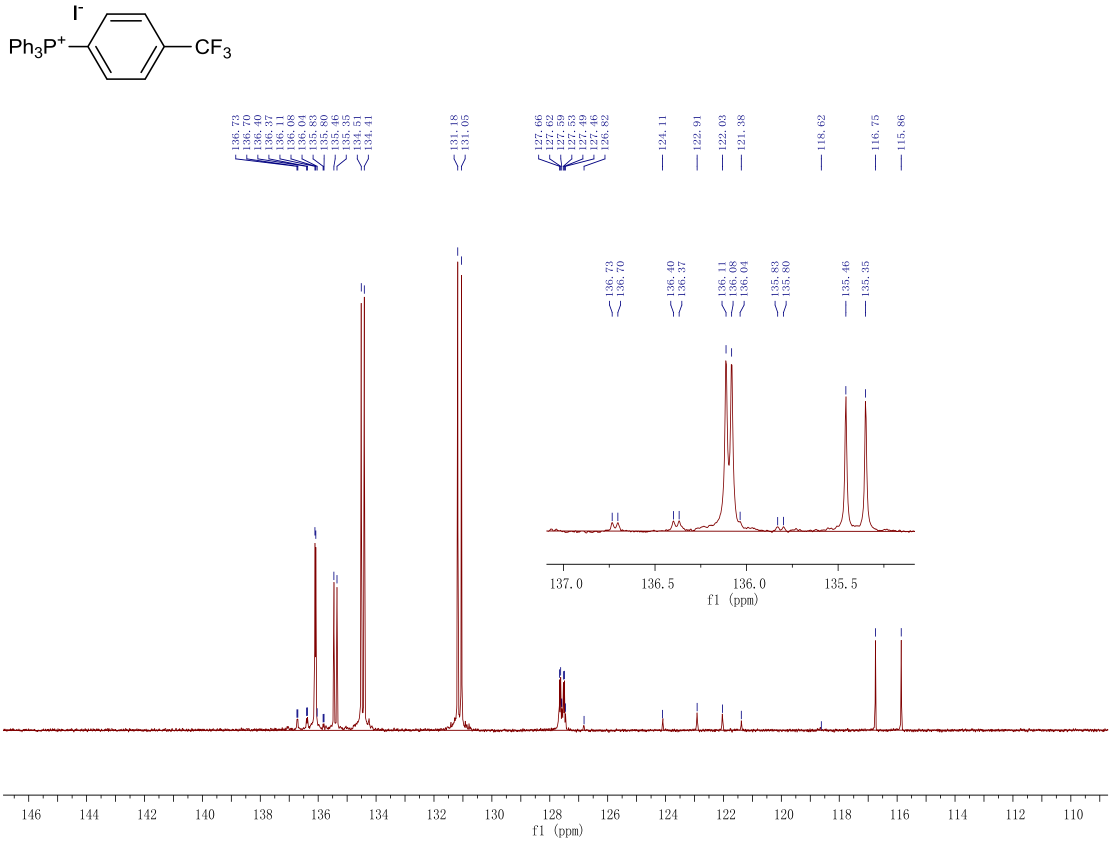

**Supplementary Figure 19. $^{1}$H NMR of (4-(Ethoxycarbonyl)phenyl)triphenyl-phosphonium iodide 2h**

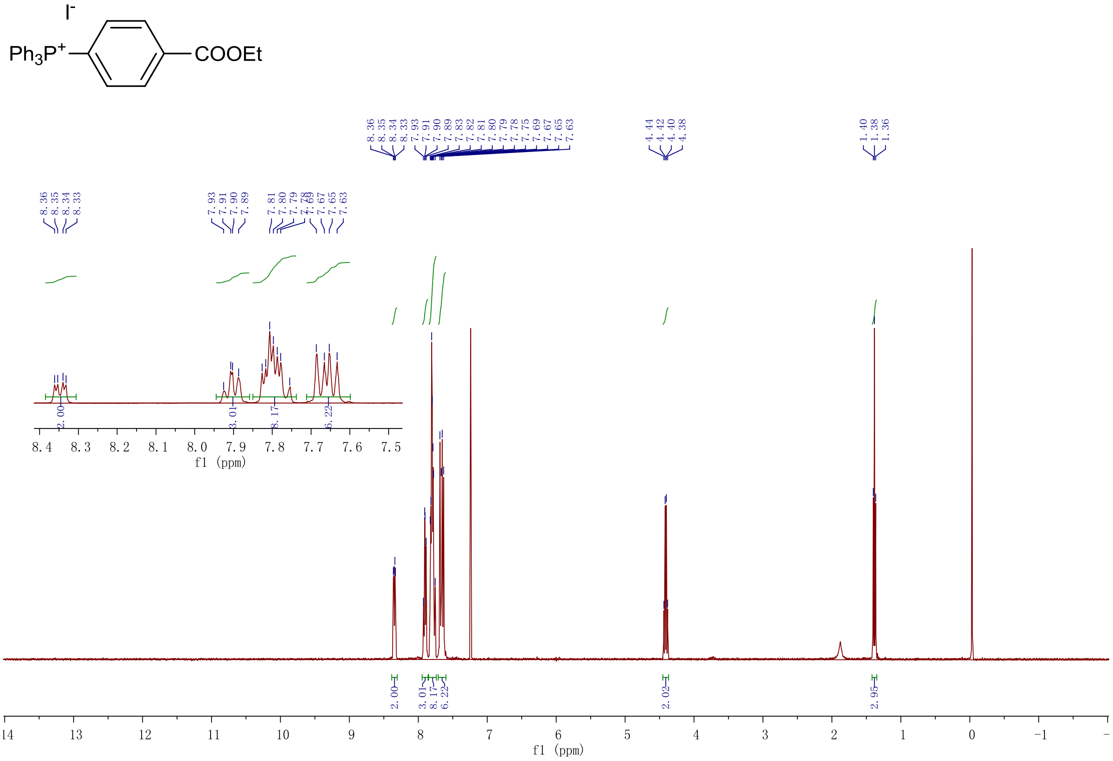


**Supplementary Figure 20. $^{31}$P NMR of (4-(Ethoxycarbonyl)phenyl)triphenyl-phosphonium iodide 2h**

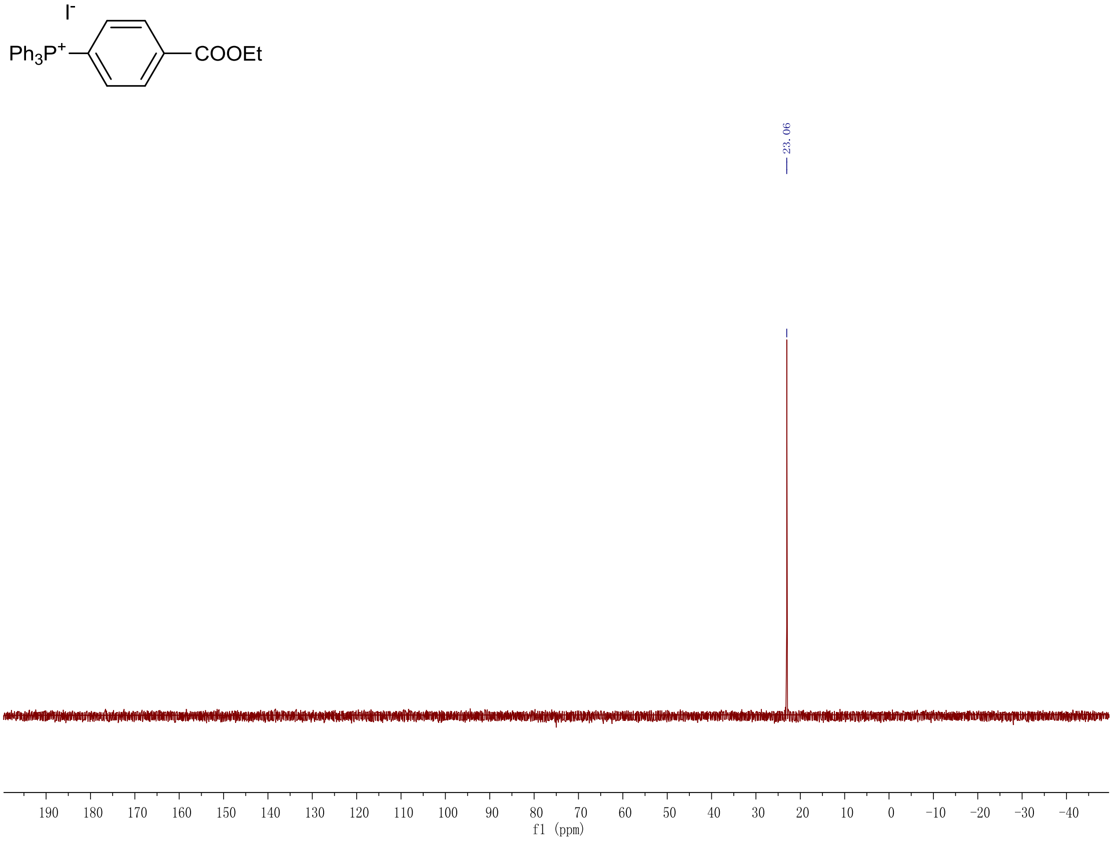

**Supplementary Figure 21. $^{13}$C NMR of (4-(Ethoxycarbonyl)phenyl)triphenyl-phosphonium iodide 2h**

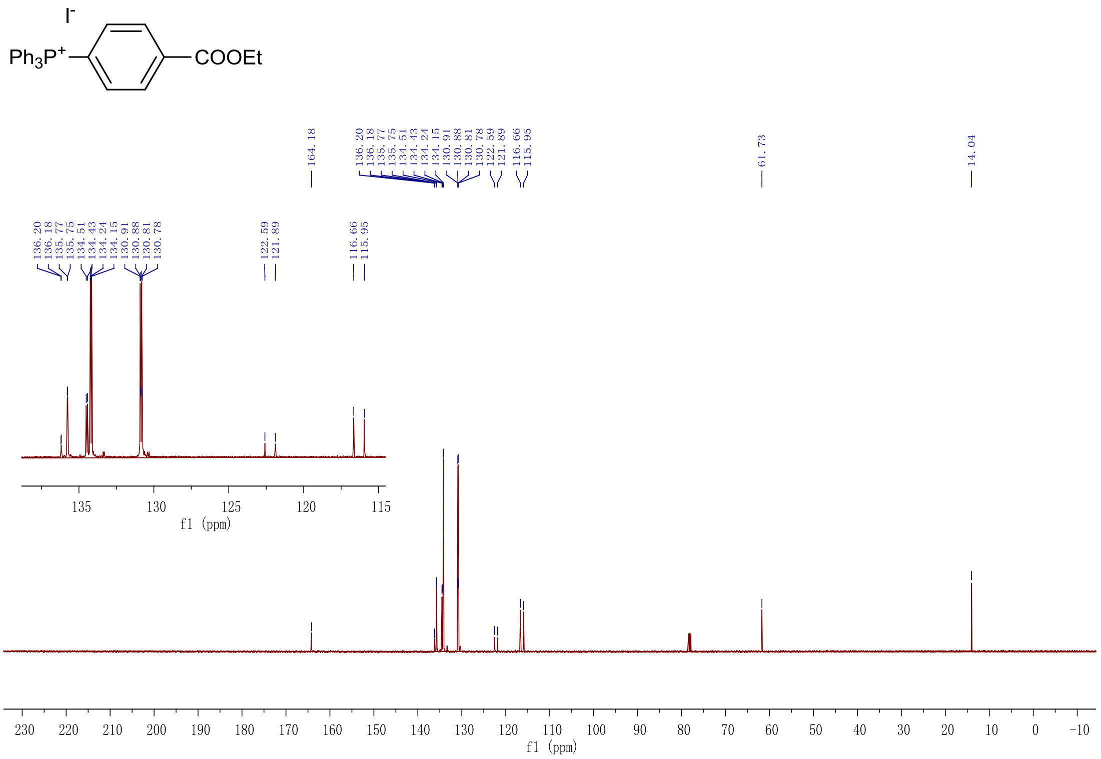


**Supplementary Figure 22. $^{1}$H NMR of (4-Acetylphenyl)triphenylphosphonium iodide 2i**

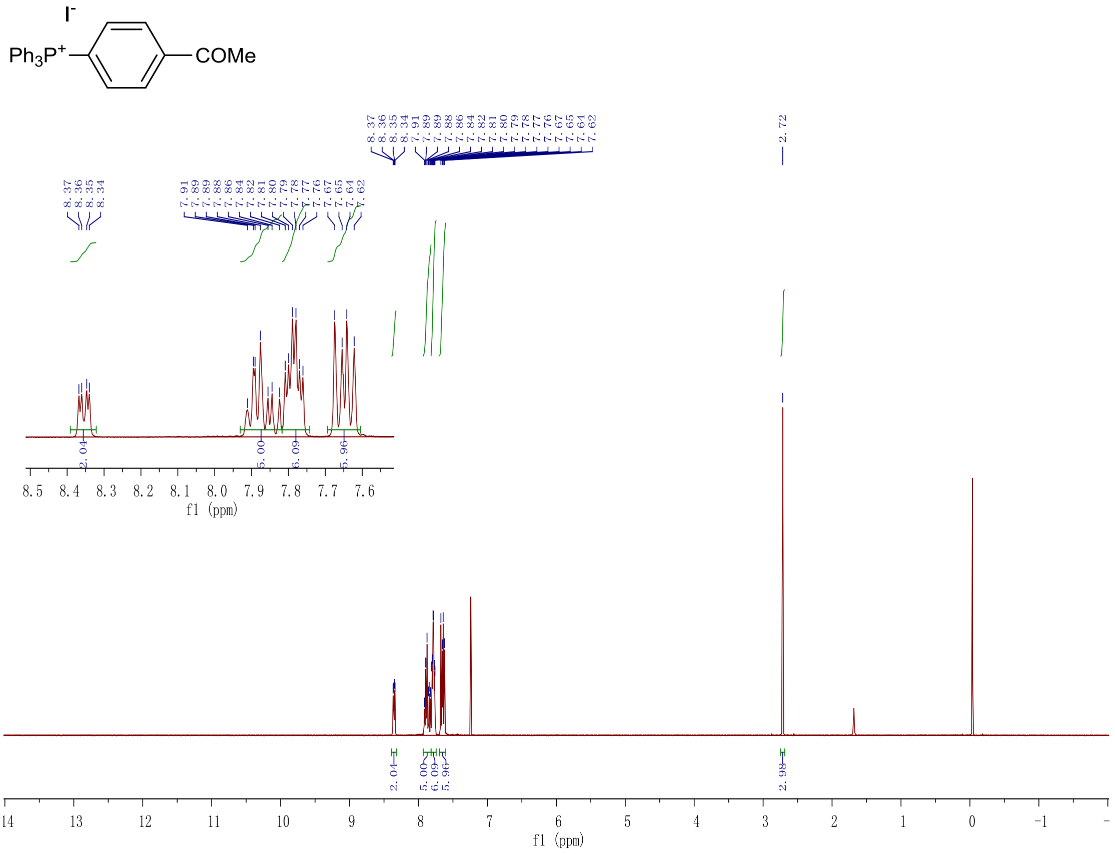

**Supplementary Figure 23.** $^{31}$**P NMR of (4-Acetylphenyl)triphenylphosphonium iodide 2i**

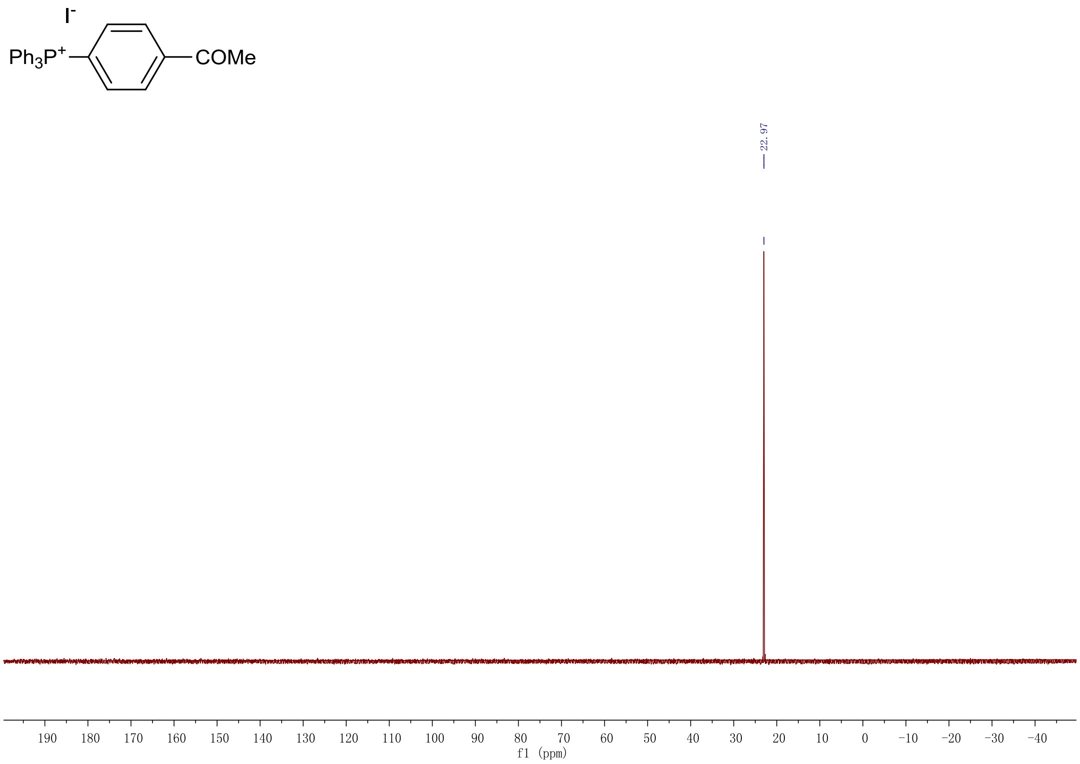


**Supplementary Figure 24.** $^{1}$**H NMR of (4-Cyanophenyl)triphenylphosphonium bis((trifluoromethyl)sulfonyl)amide 2e'**

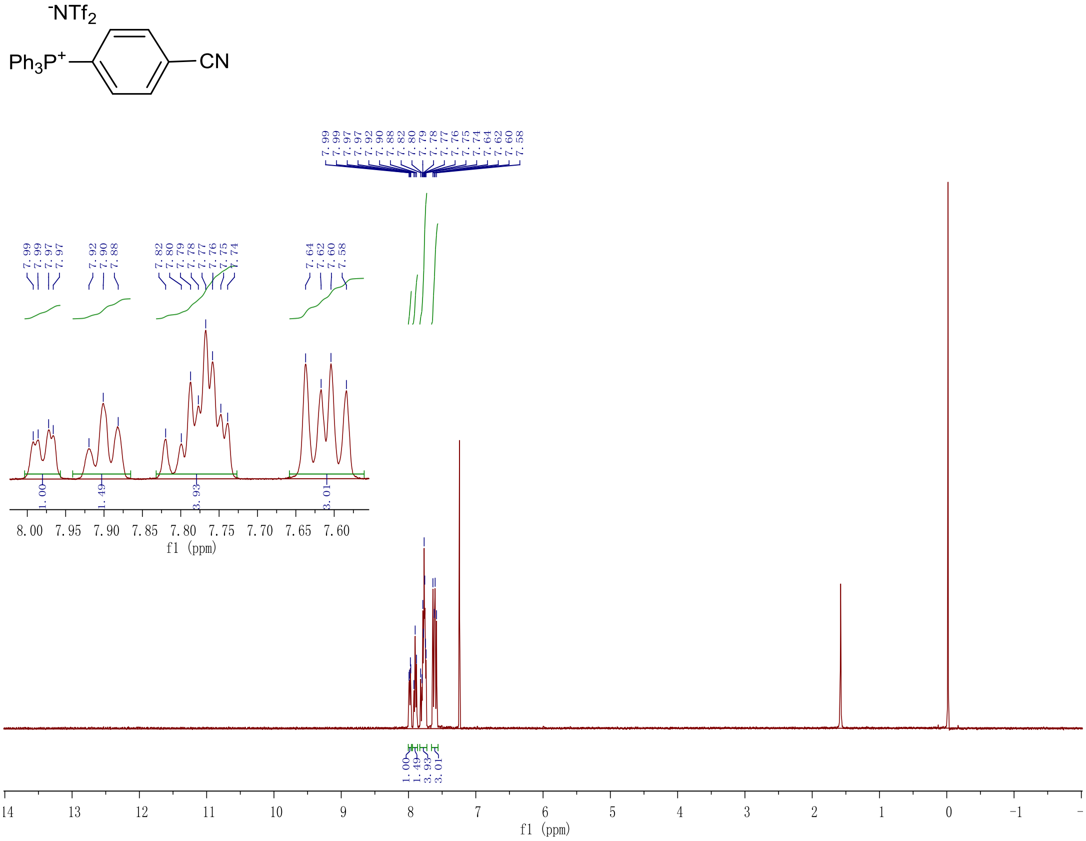

**Supplementary Figure 25. $^{19}$F NMR of (4-Cyanophenyl)triphenylphosphonium bis((trifluoromethyl)sulfonyl)amide 2e'**

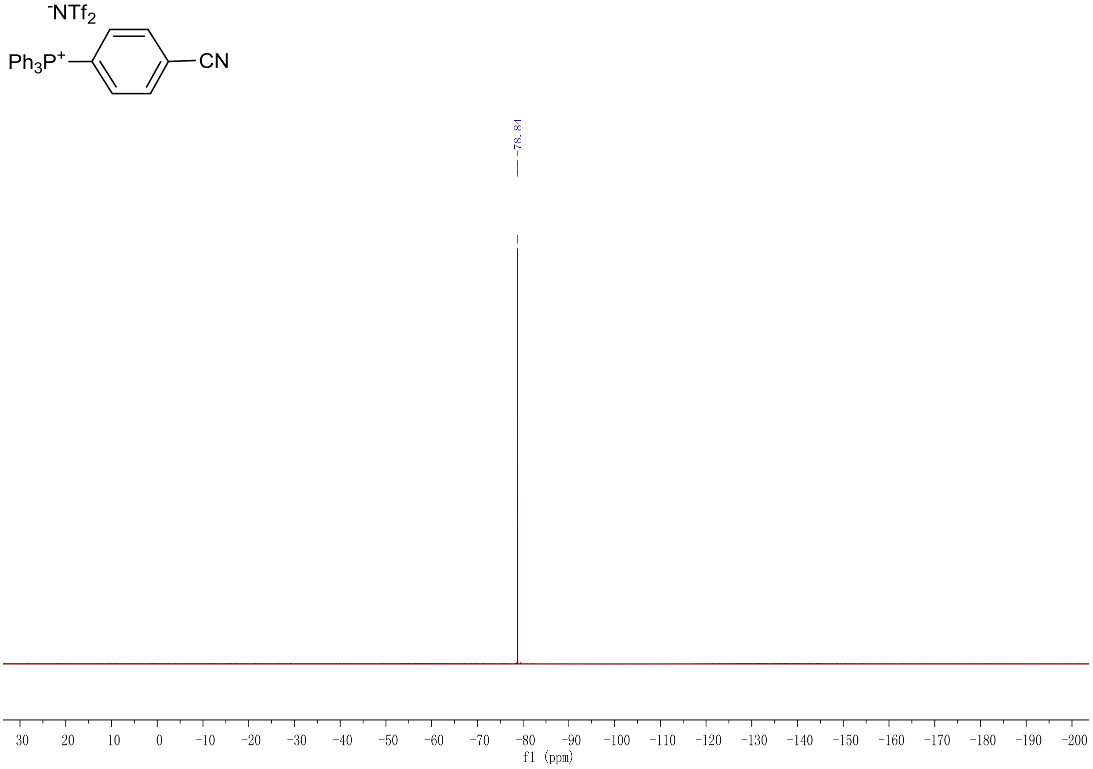


**Supplementary Figure 26. $^{31}$P NMR of (4-Cyanophenyl)triphenylphosphonium bis((trifluoromethyl)sulfonyl)amide 2e'**

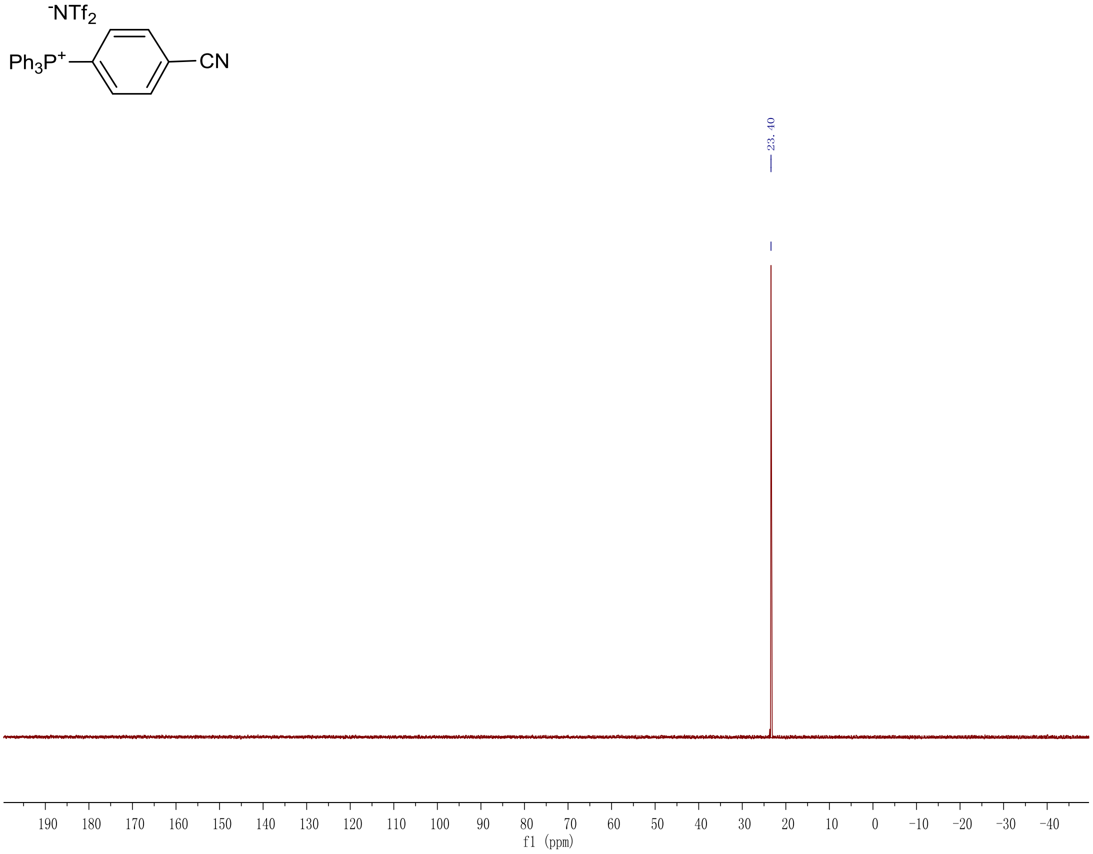

**Supplementary Figure 27. $^{13}$C NMR of (4-Cyanophenyl)triphenylphosphonium bis((trifluoromethyl)sulfonyl)amide 2e'**

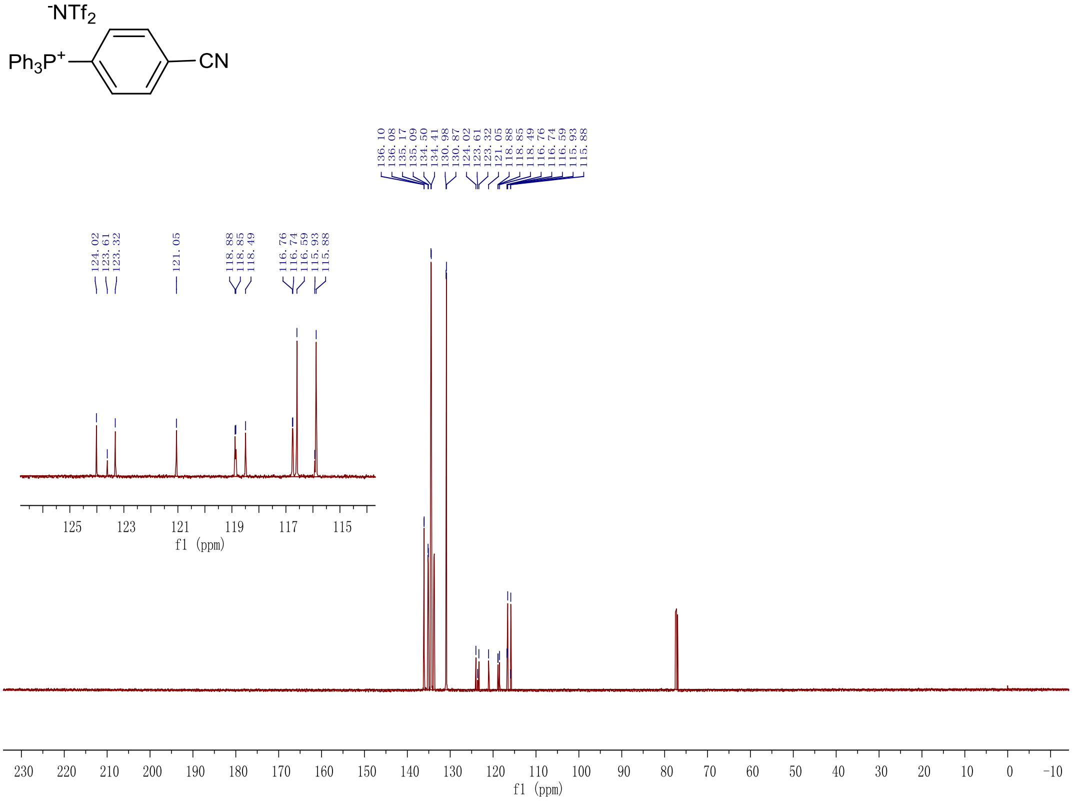

**Supplementary Figure 28. $^{1}$H NMR of (3-Cyanophenyl)triphenylphosphonium bis((trifluoromethyl)sulfonyl)amide 2f'**

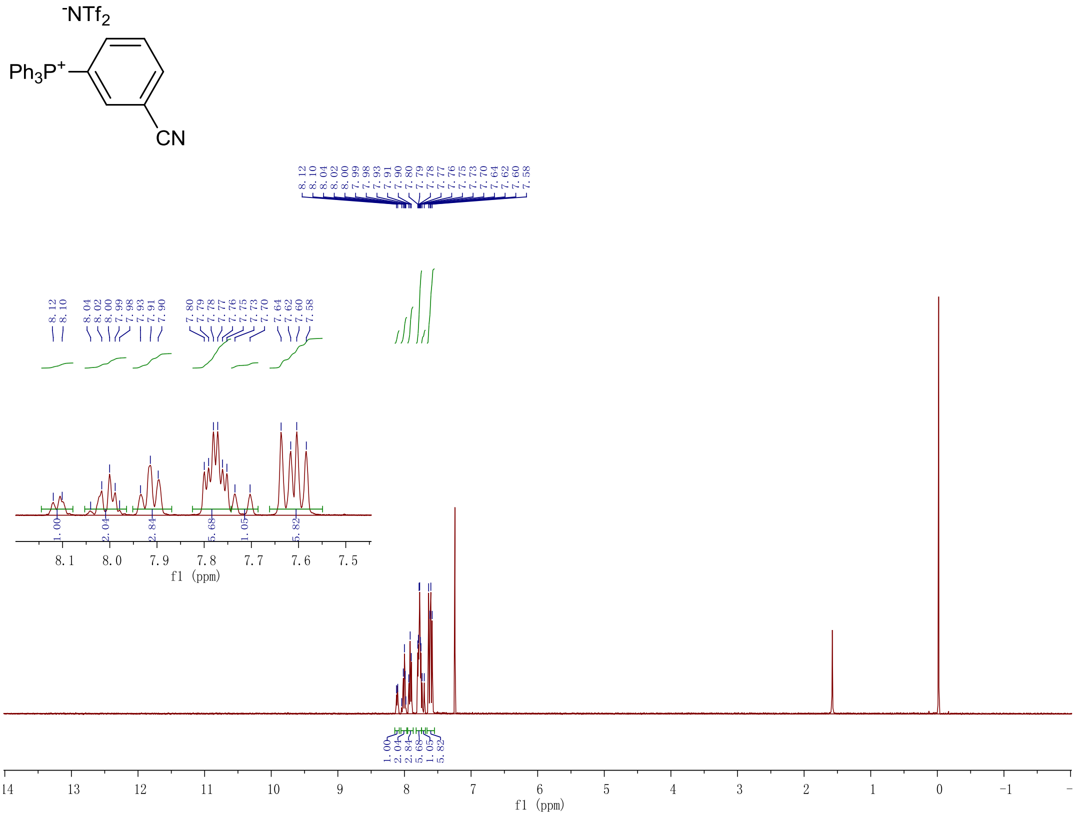

**Supplementary Figure 29. $^{19}$F NMR of (3-Cyanophenyl)triphenylphosphonium bis((trifluoromethyl)sulfonyl)amide 2f’**

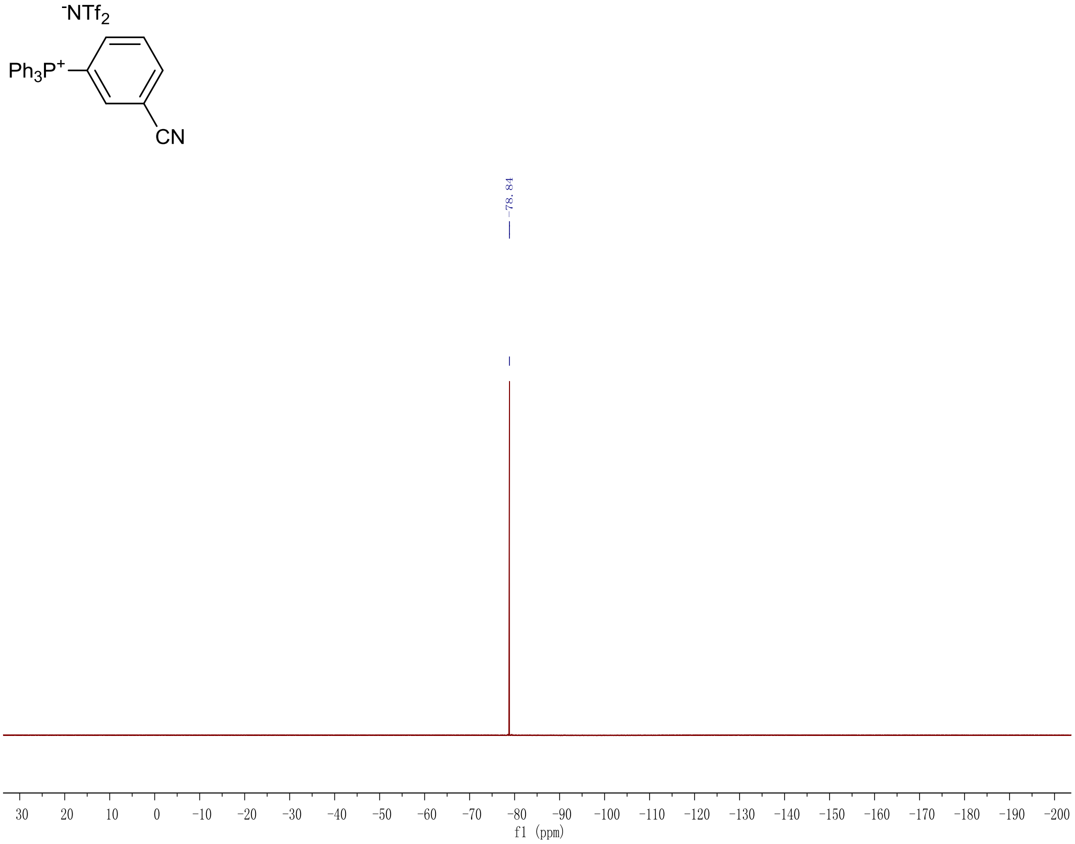


**Supplementary Figure 30. $^{31}$P NMR of (3-Cyanophenyl)triphenylphosphonium bis((trifluoromethyl)sulfonyl)amide 2f’**

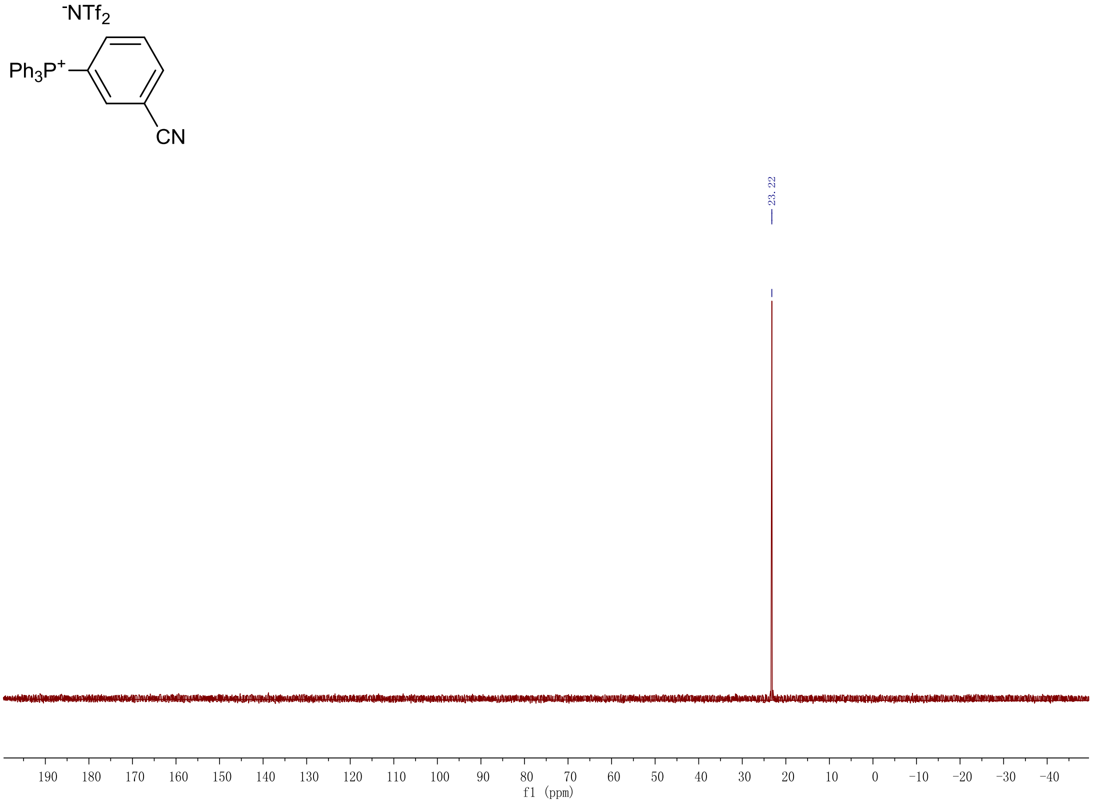

## Supplementary Figure 31. $^{13}C$ NMR of (3-Cyanophenyl)triphenylphosphonium bis((trifluoromethyl)sulfonyl)amide 2f’

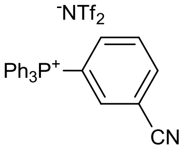


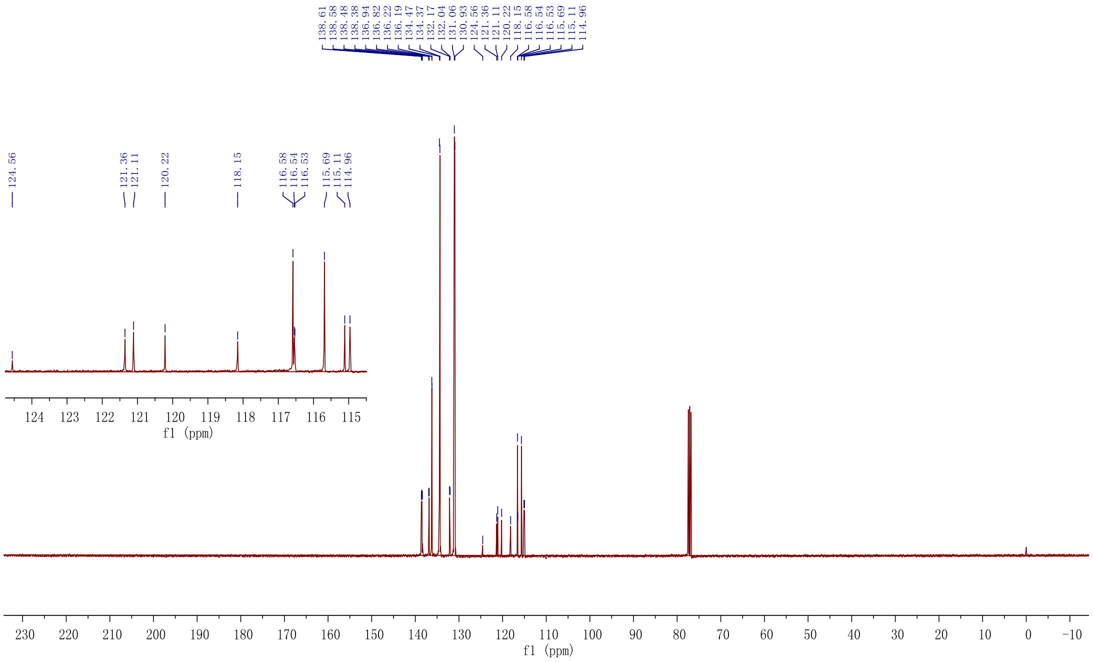


## Supplementary Figure 32. $^{1}H$ NMR of Triphenyl(4-(trifluoromethyl)phenyl) -phosphonium bis((trifluoromethyl)sulfonyl)amide 2g’

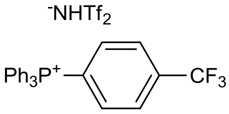


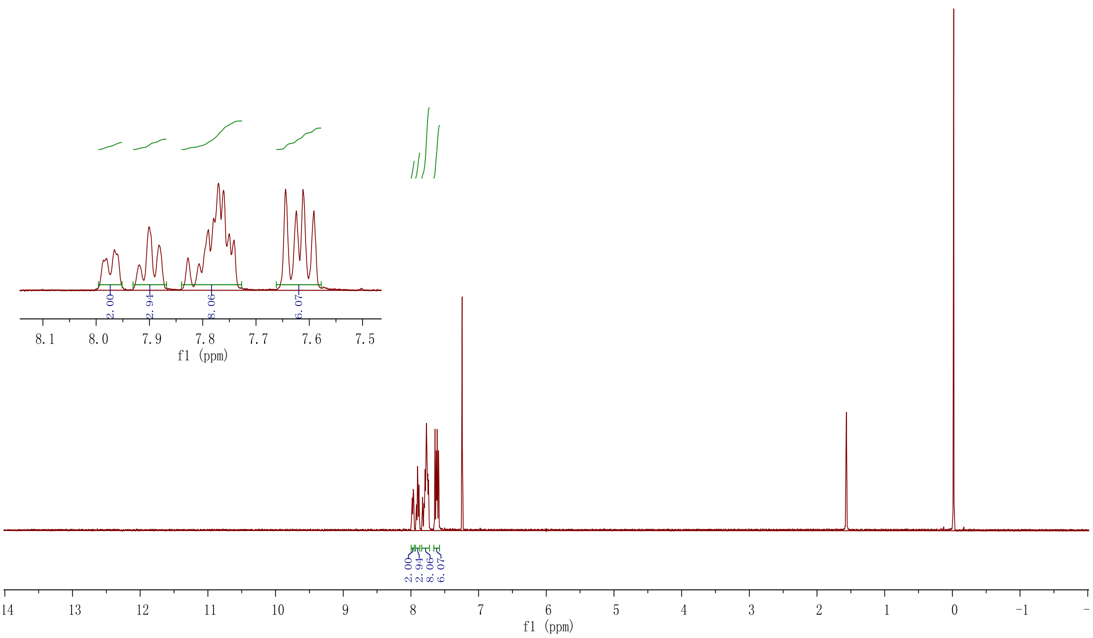

**Supplementary Figure 33. $^{19}$F NMR of Triphenyl(4-(trifluoromethyl)phenyl)-phosphonium bis((trifluoromethyl)sulfonyl)amide 2g'**

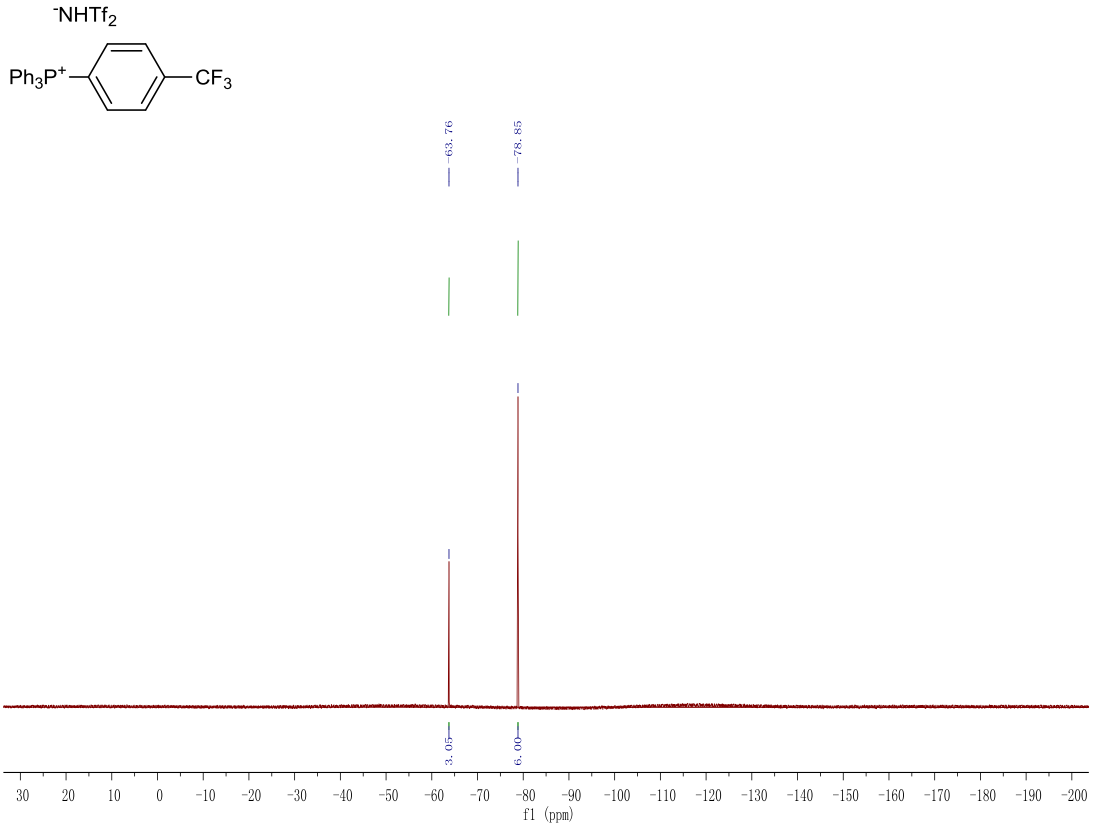


**Supplementary Figure 34. $^{31}$P NMR of Triphenyl(4-(trifluoromethyl)phenyl)-phosphonium bis((trifluoromethyl)sulfonyl)amide 2g'**

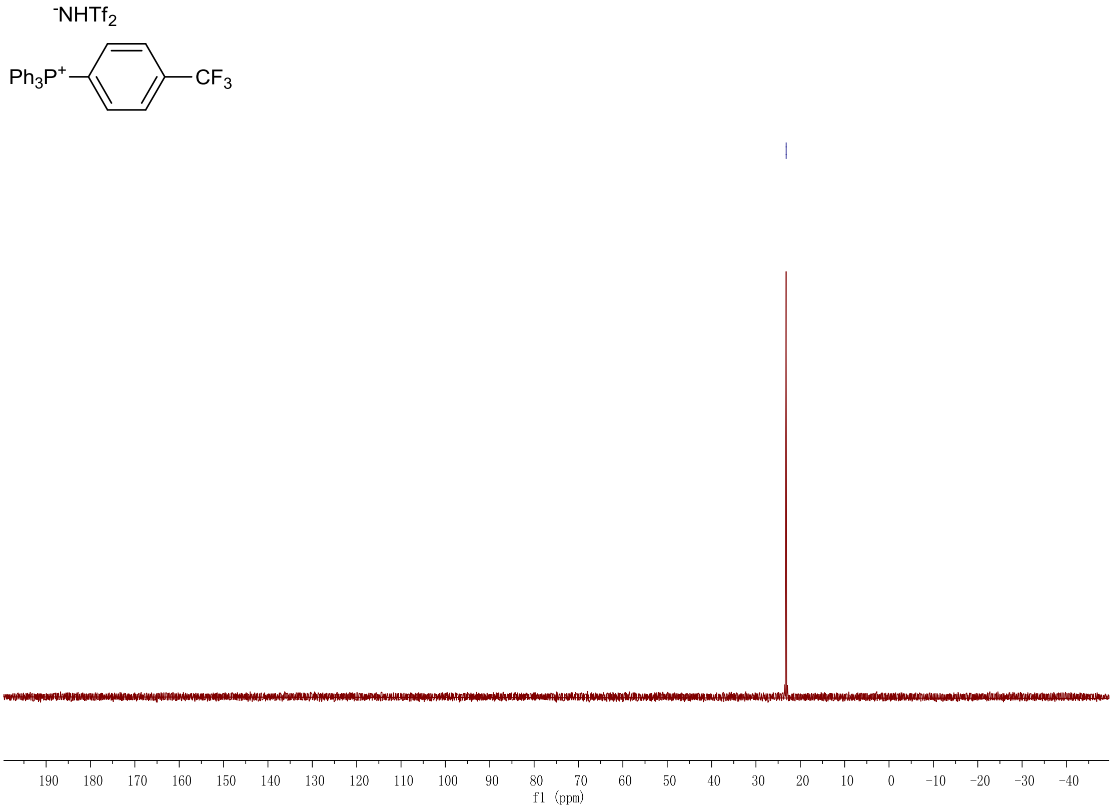

**Supplementary Figure 35. $^{13}C$ NMR of Triphenyl(4-(trifluoromethyl)phenyl)-phosphonium bis((trifluoromethyl)sulfonyl)amide 2g'**

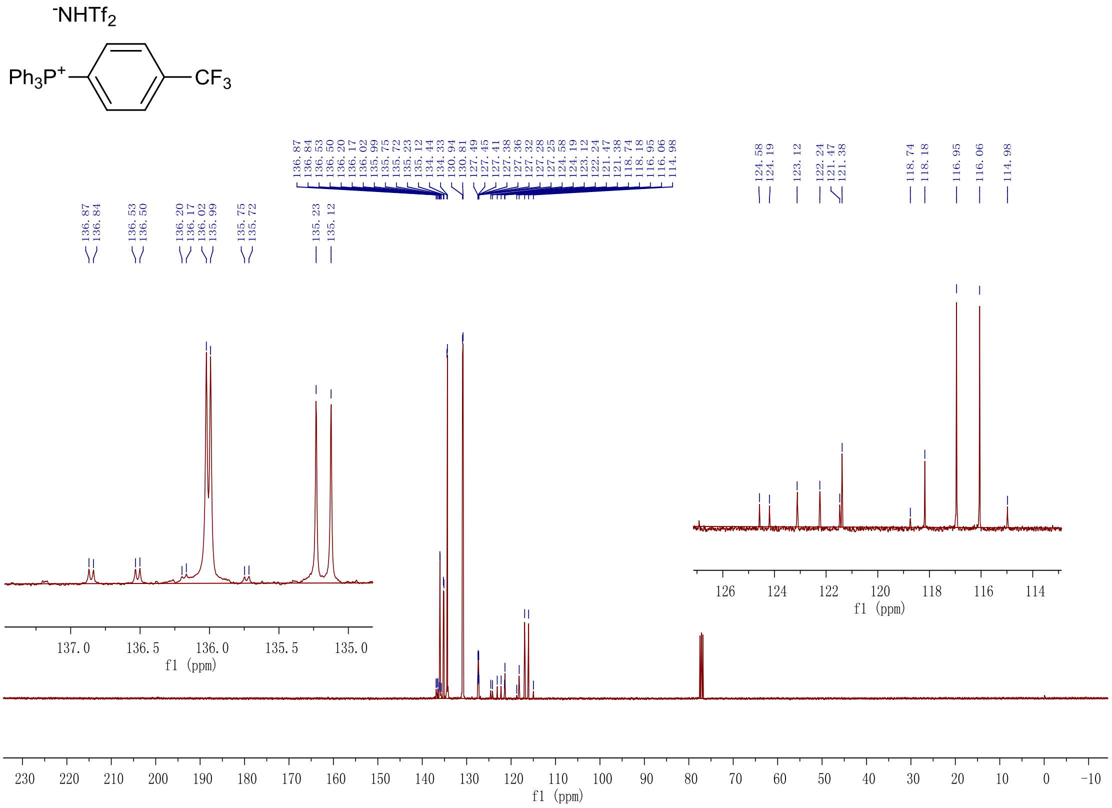


**Supplementary Figure 36. $^{1}H$ NMR of (4-(Ethoxycarbonyl)phenyl)triphenyl-phosphonium bis((trifluoromethyl)sulfonyl)amide 2h'**

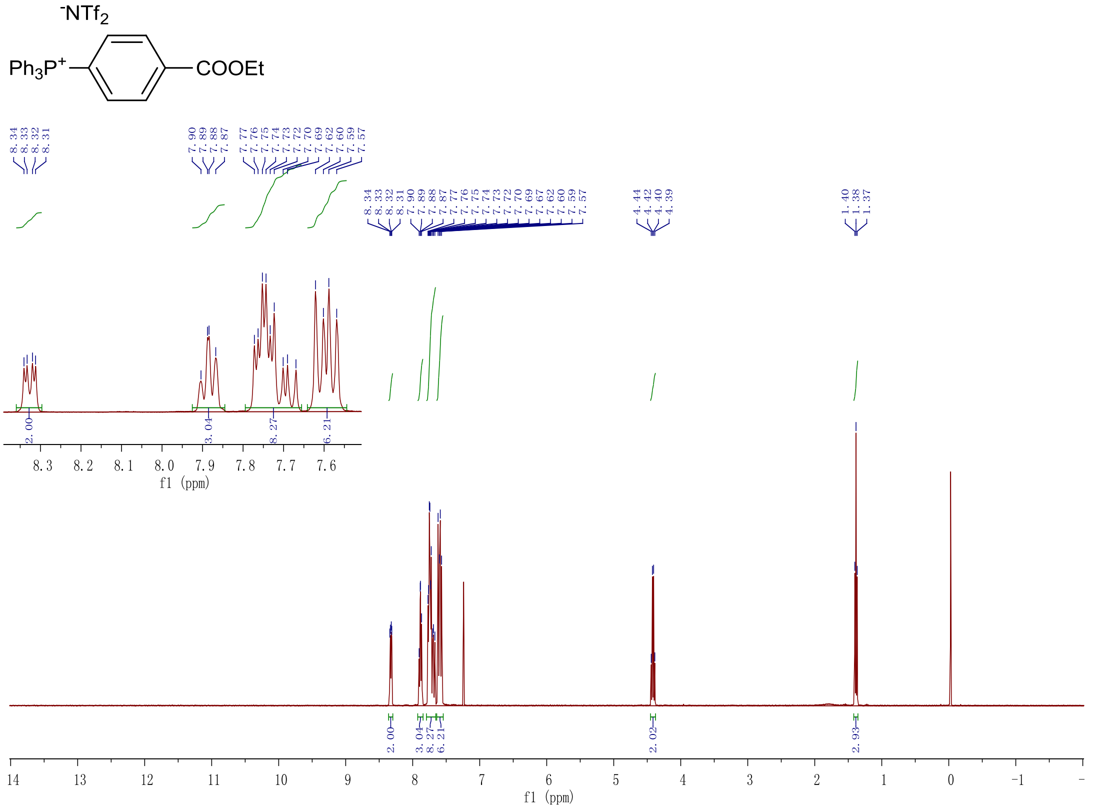

**Supplementary Figure 37. $^{19}$F NMR of (4-(Ethoxycarbonyl)phenyl)triphenyl -phosphonium bis((trifluoromethyl)sulfonyl)amide 2h'**

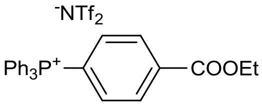


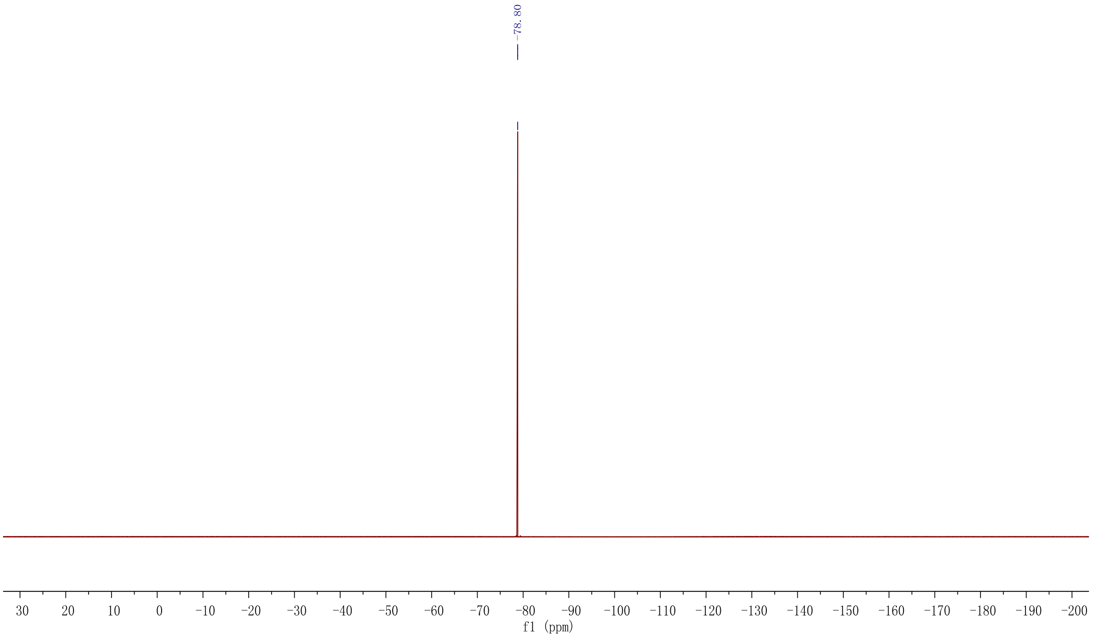


**Supplementary Figure 38. $^{31}$P NMR of (4-(Ethoxycarbonyl)phenyl)triphenyl -phosphonium bis((trifluoromethyl)sulfonyl)amide 2h'**

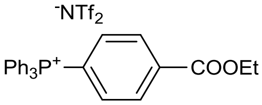


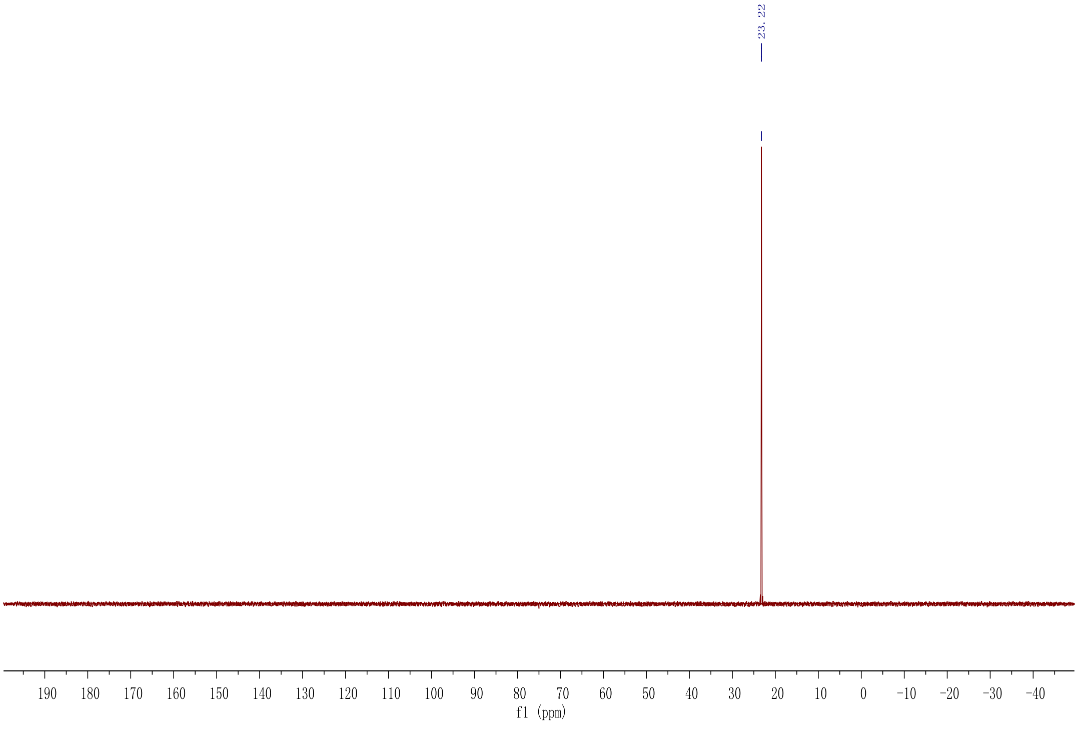

**Supplementary Figure 39. $^{13}$C NMR of (4-(Ethoxycarbonyl)phenyl)triphenyl -phosphonium bis((trifluoromethyl)sulfonyl)amide 2h'**

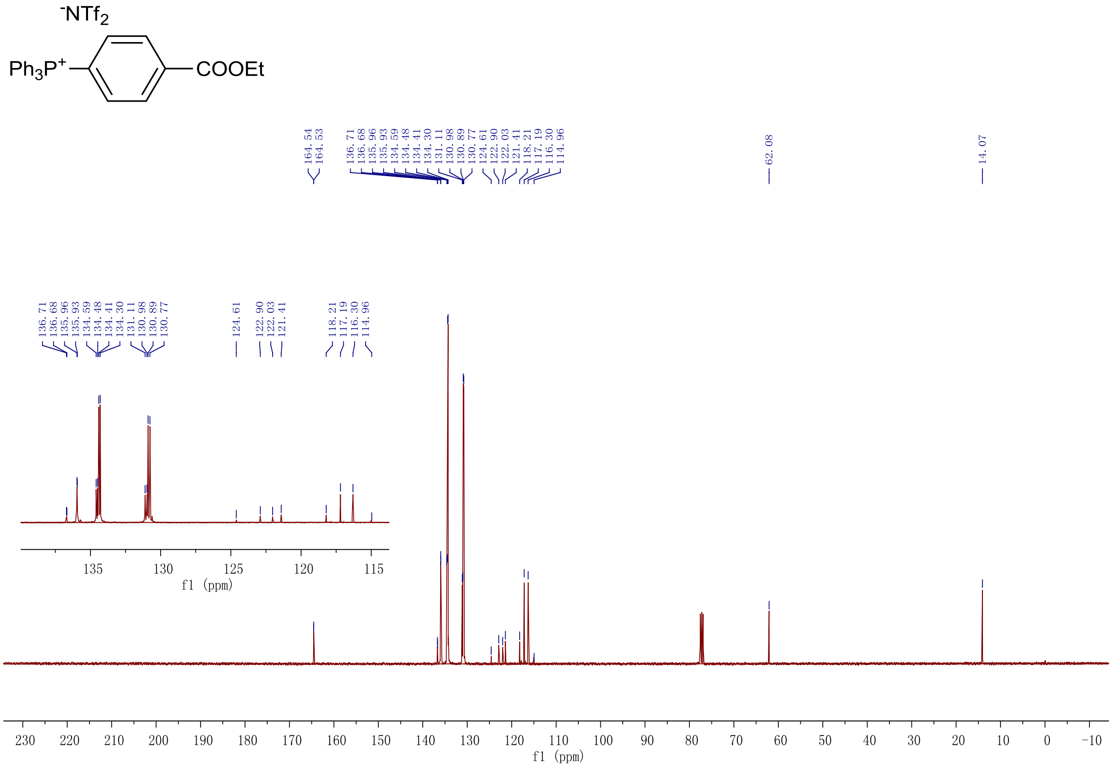


**Supplementary Figure 40. $^{1}$H NMR of (4-Acetylphenyl)triphenylphosphonium Bis((trifluoromethyl)sulfonyl)amide 2i'**

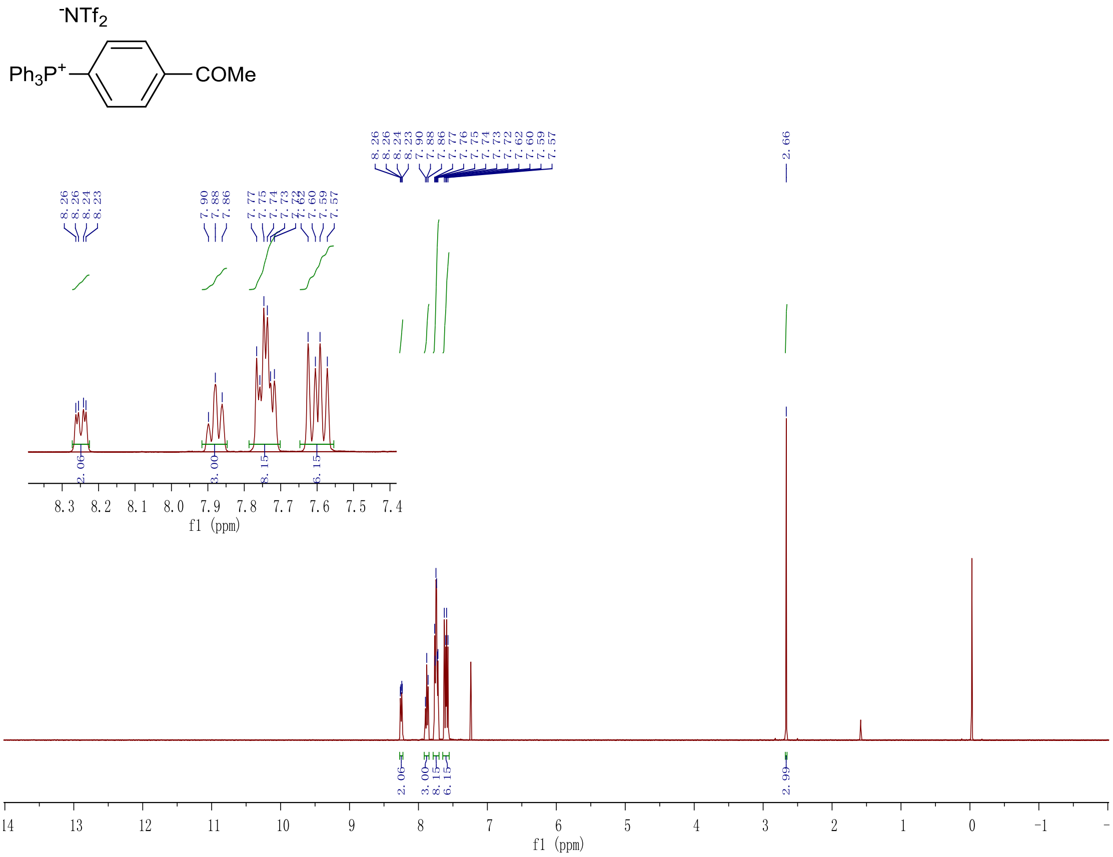

**Supplementary Figure 41. $^{31}$P NMR of (4-Acetylphenyl)triphenylphosphonium Bis((trifluoromethyl)sulfonyl)amide 2i'**

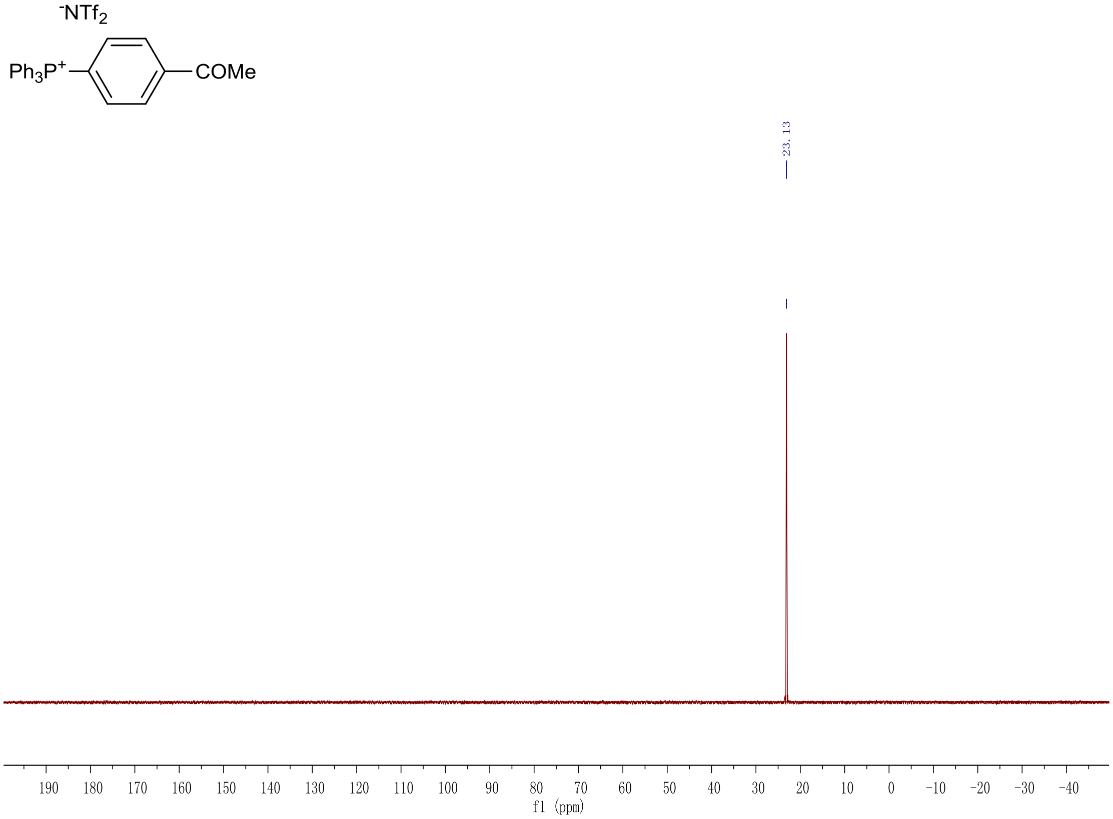


**Supplementary Figure 42. $^{19}$F NMR of (4-Acetylphenyl)triphenylphosphonium Bis((trifluoromethyl)sulfonyl)amide 2i'**

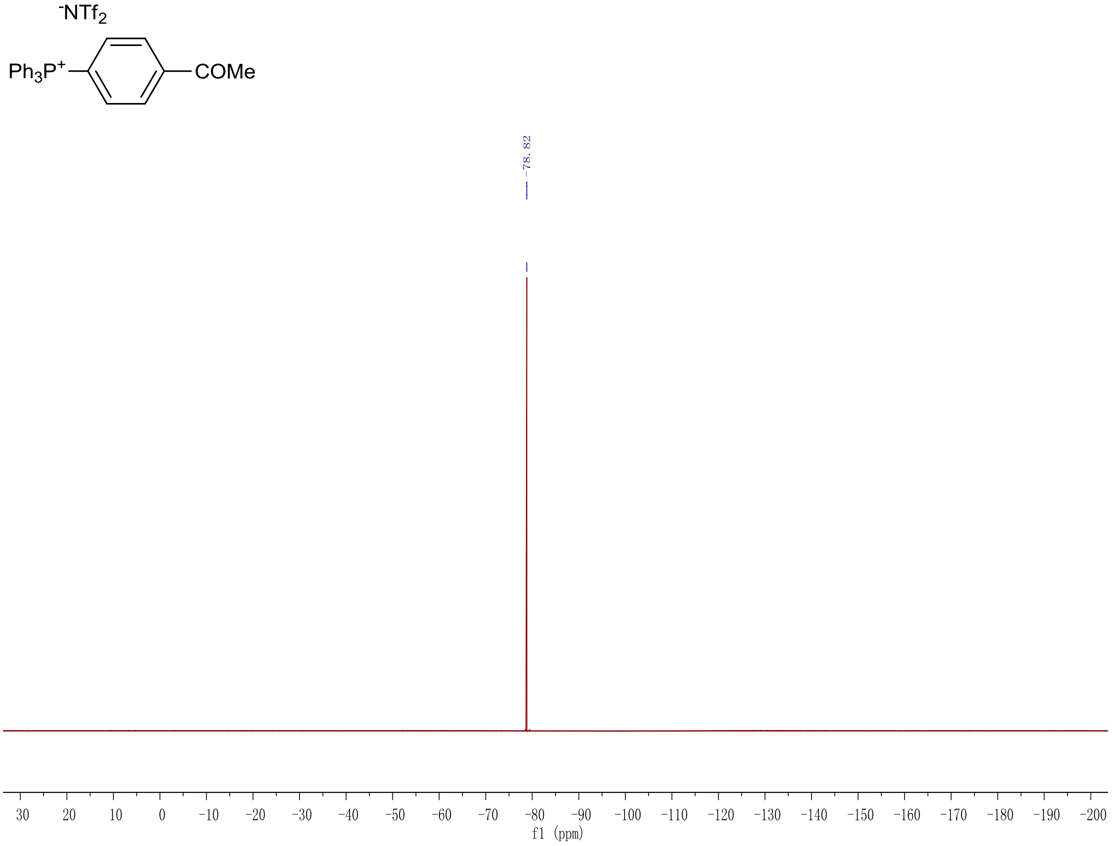

**Supplementary Figure 43. $^{13}$C NMR of (4-Acetylphenyl)triphenylphosphonium Bis((trifluoromethyl)sulfonyl)amide 2i'**

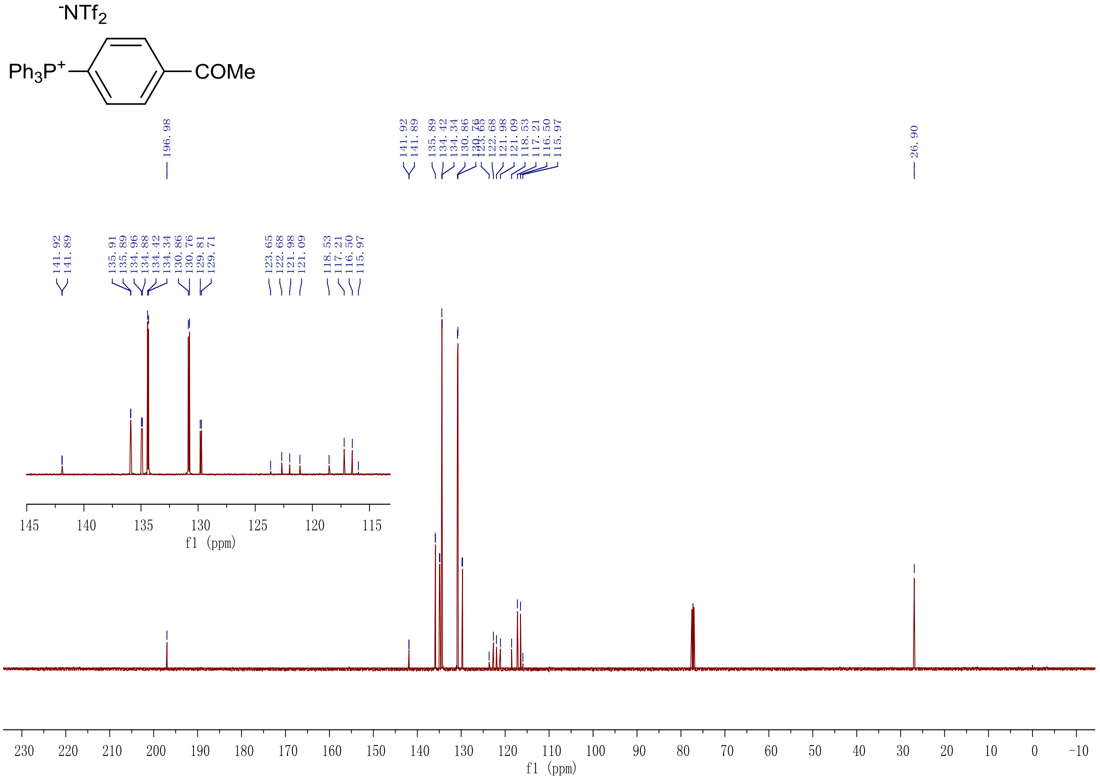

**Supplementary Figure 44. $^{1}$H NMR of Triphenyl(pyridin-2-yl)phosphonium iodide 2j**

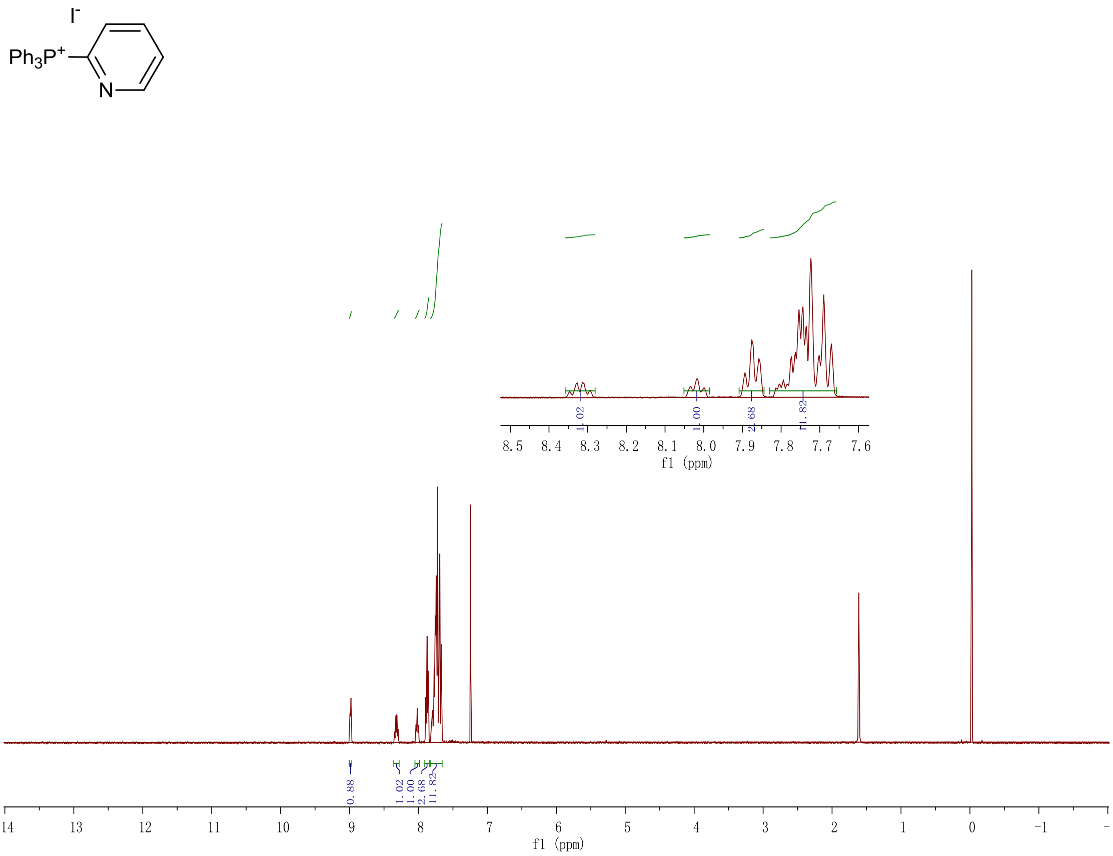

**Supplementary Figure 45. $^{31}$P NMR of Triphenyl(pyridin-2-yl)phosphonium iodide 2j**

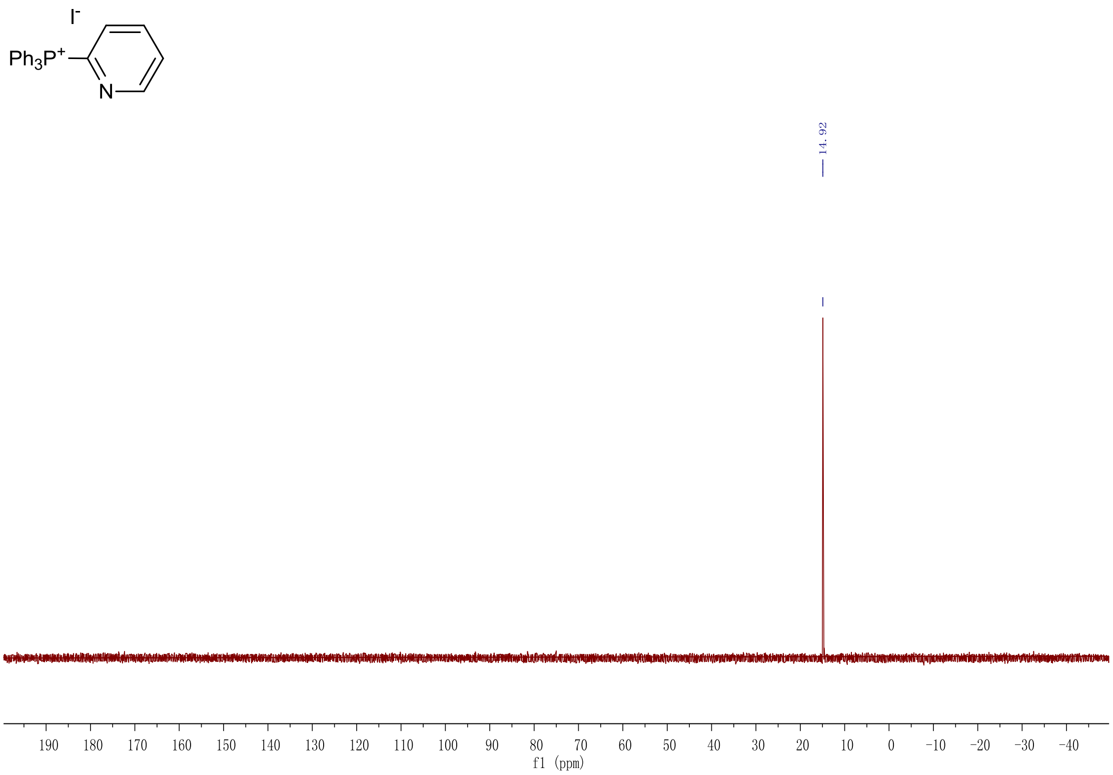


**Supplementary Figure 46. $^{1}$H NMR of [1,1'-Biphenyl]-4-yl(phenyl)methanol 3aa**

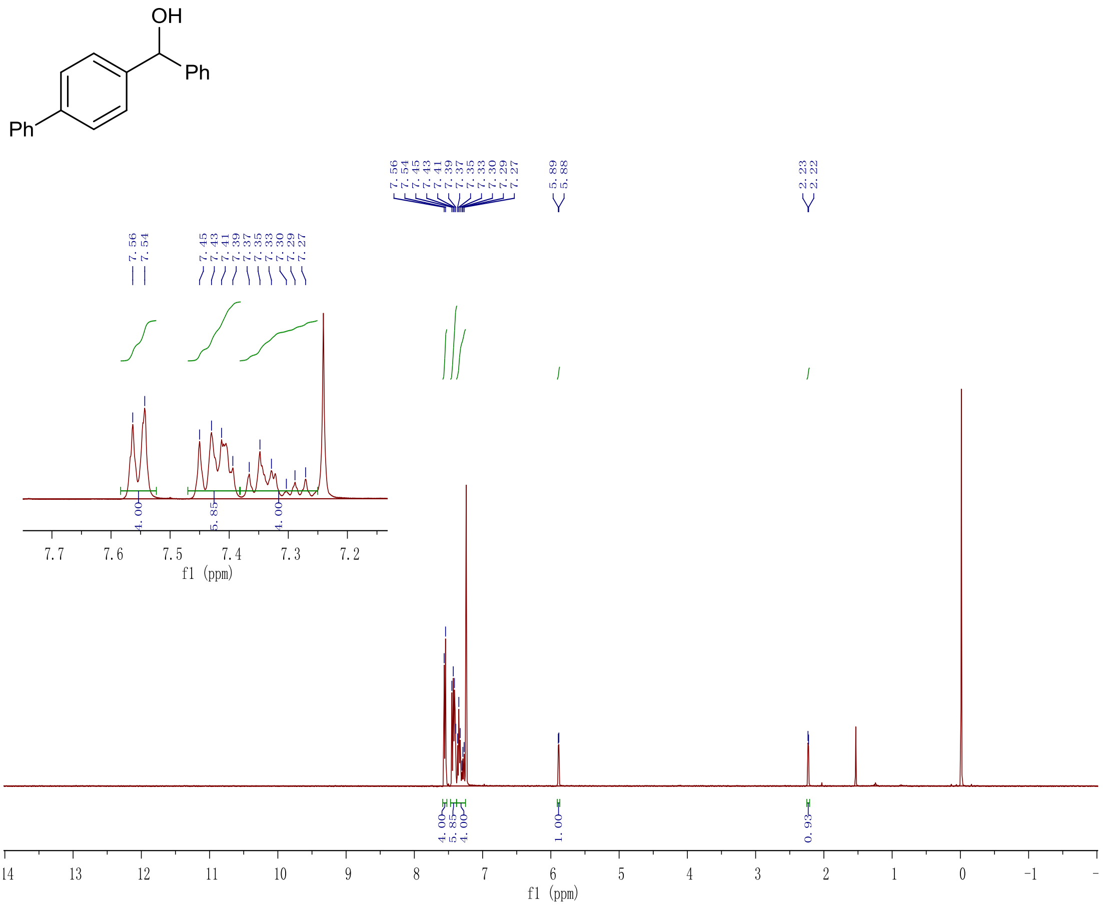

**Supplementary Figure 47. $^1$H NMR of (4-(Dimethylamino)phenyl)(phenyl)-methanol 3ba**

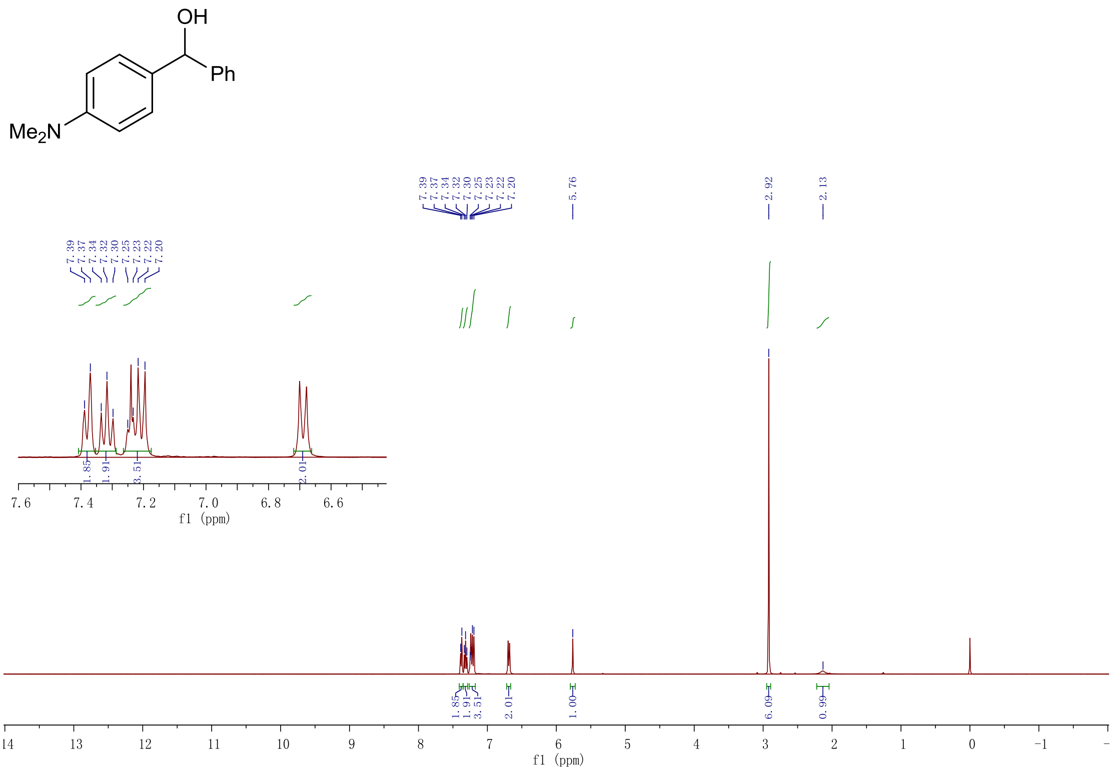


**Supplementary Figure 48. $^1$H NMR of (4-Methoxyphenyl)(phenyl)methanol 3ca**

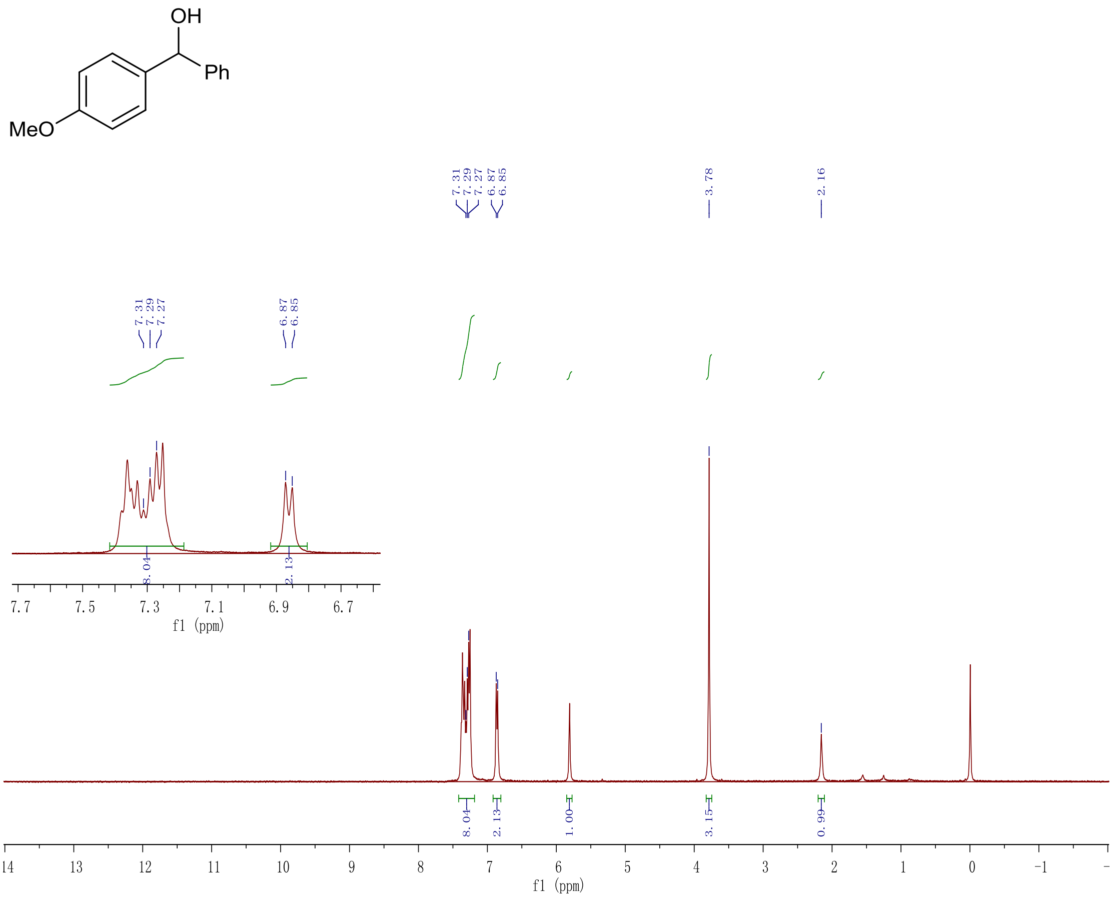

## Supplementary Figure 49. $^{1}$H NMR of (2-Methoxyphenyl)(phenyl)methanol 3da

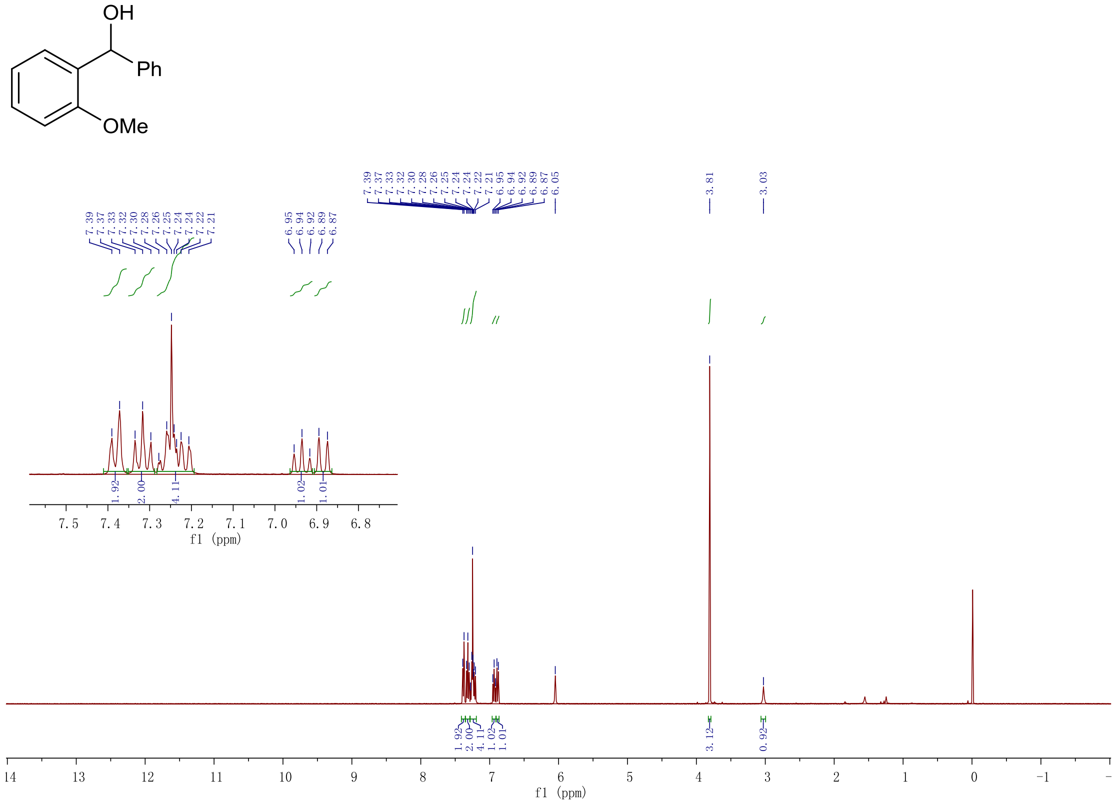


## Supplementary Figure 50. $^{1}$H NMR of Mesityl(phenyl)methanol 3ea

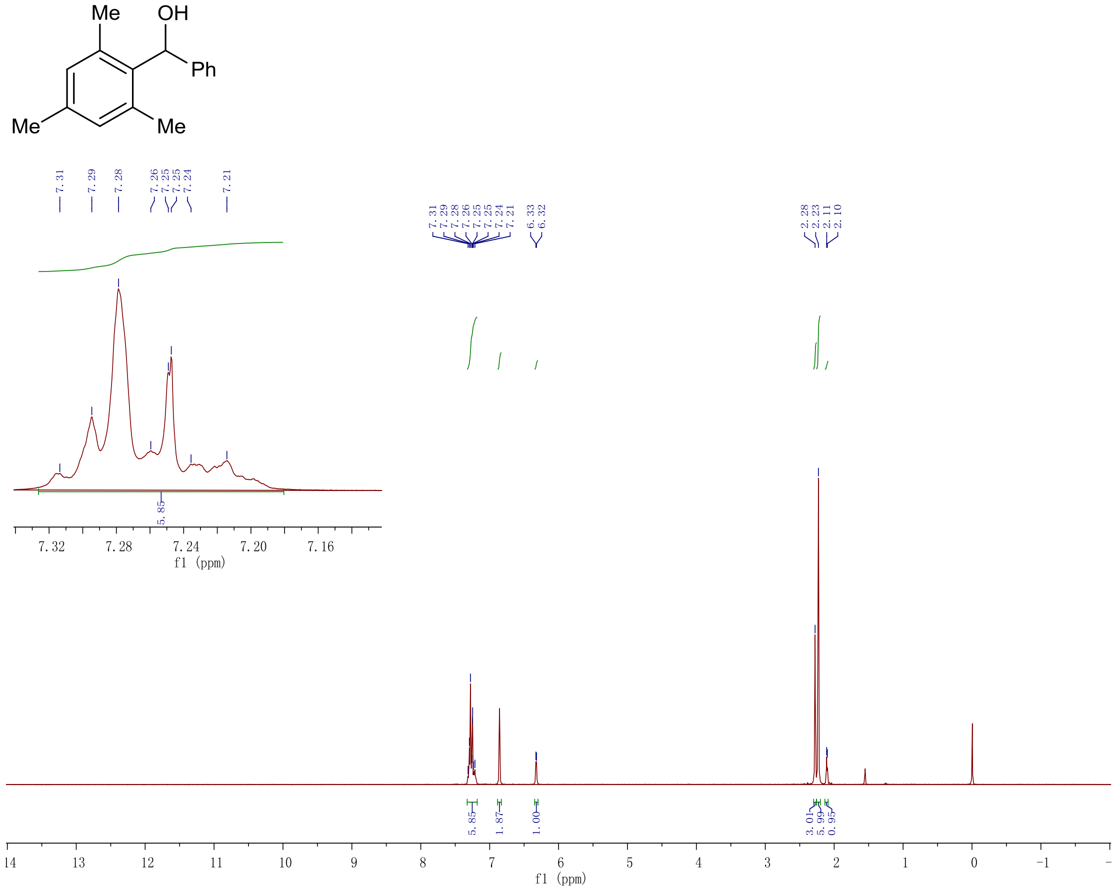

## Supplementary Figure 51. $^{1}$H NMR of (4-Fluorophenyl)(phenyl)methanol 3fa

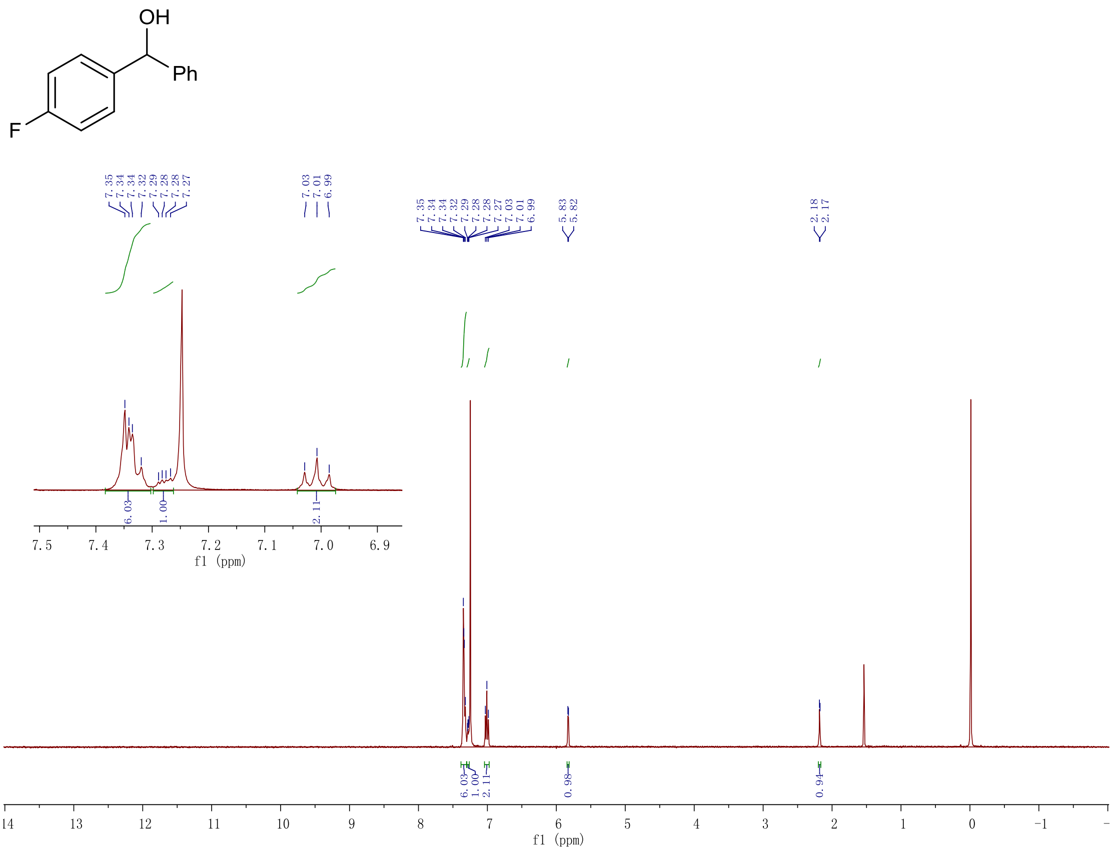


## Supplementary Figure 52. $^{19}$F NMR of (4-Fluorophenyl)(phenyl)methanol 3fa

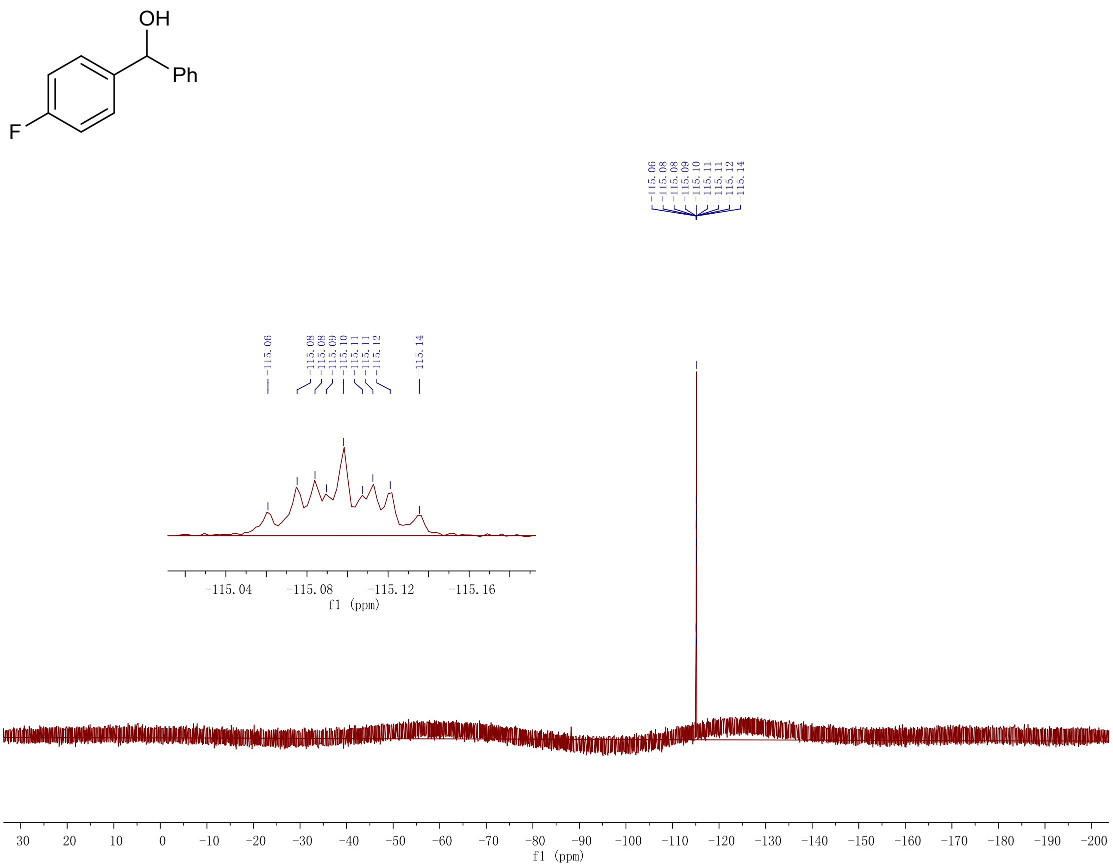

## Supplementary Figure 53. $^{1}$H NMR of (4-Bromophenyl)(phenyl)methanol 3ga

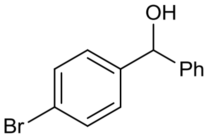


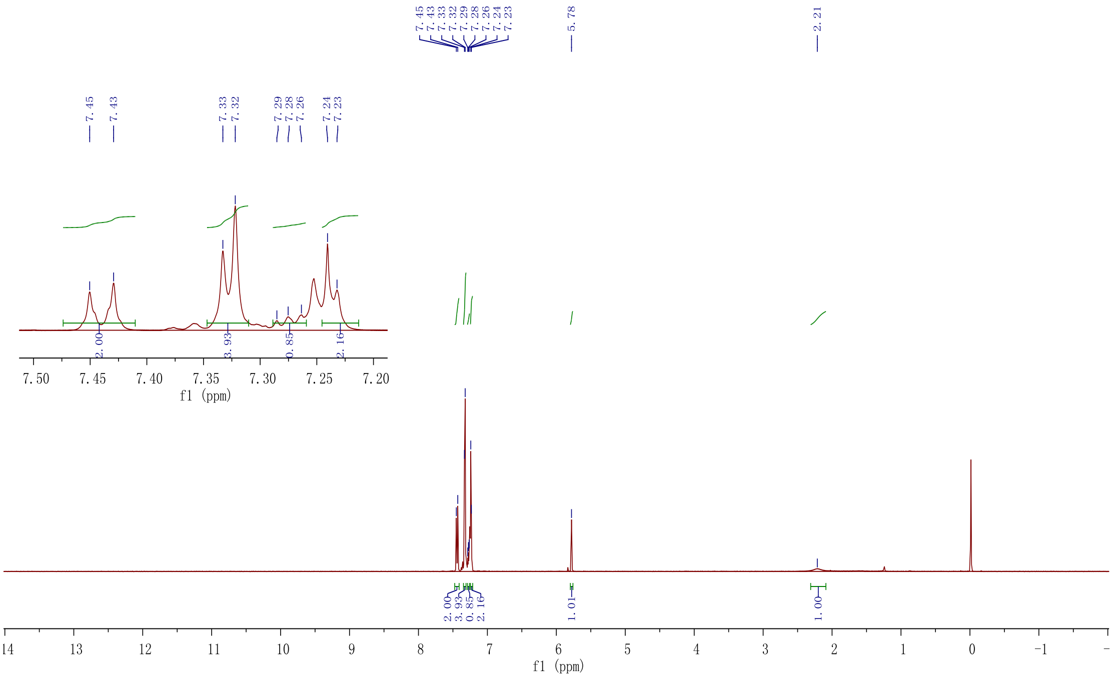


## Supplementary Figure 54. $^{1}$H NMR of (3-Bromophenyl)(phenyl)methanol 3ha

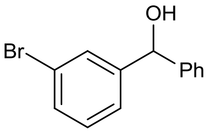


**3ha**

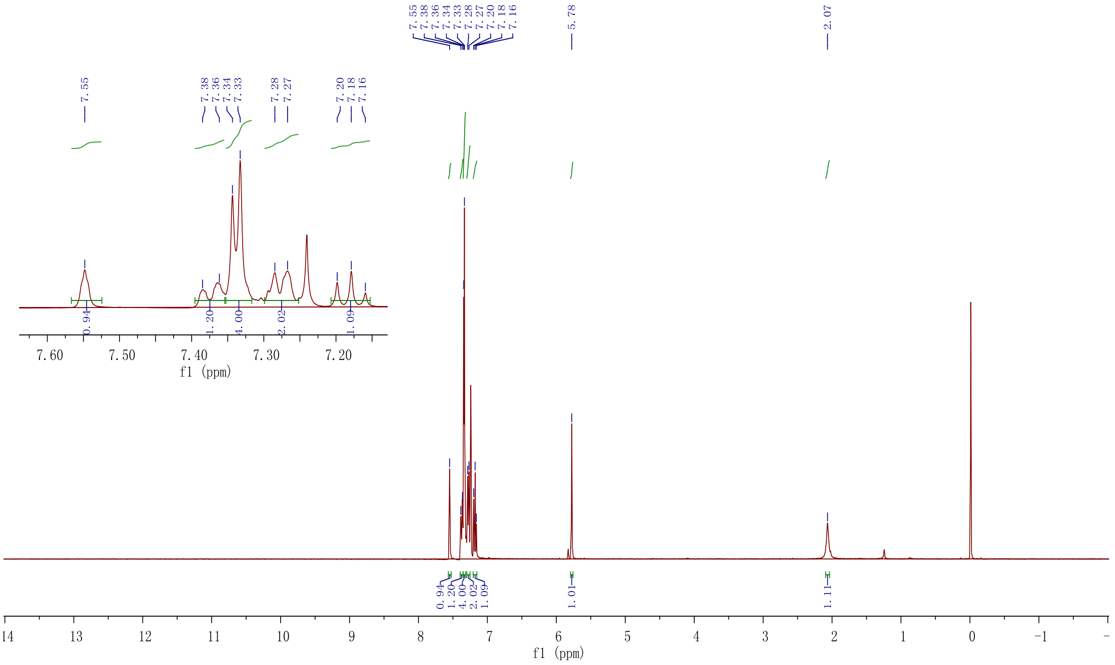

**Supplementary Figure 55. $^{1}$H NMR of Phenyl(4-(trifluoromethyl)phenyl)-methanol 3ia**

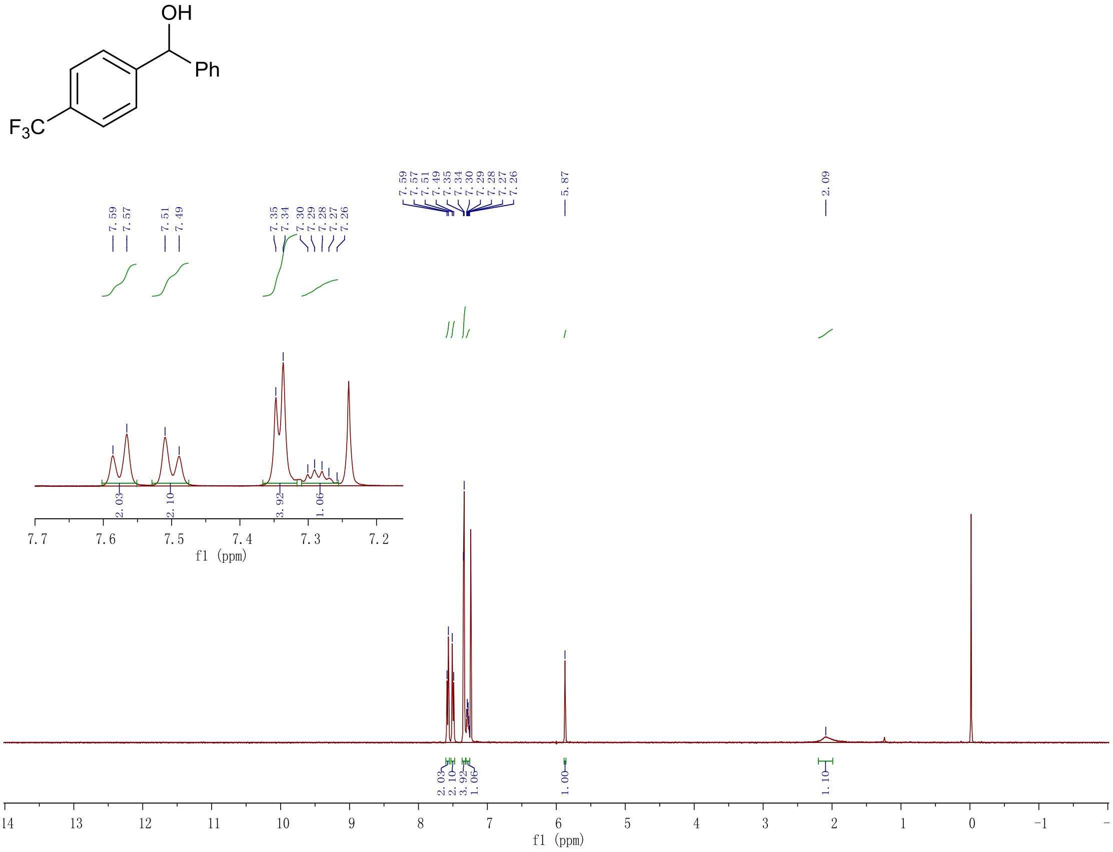


**Supplementary Figure 56. $^{19}$F NMR of Phenyl(4-(trifluoromethyl)phenyl)-methanol 3ia**

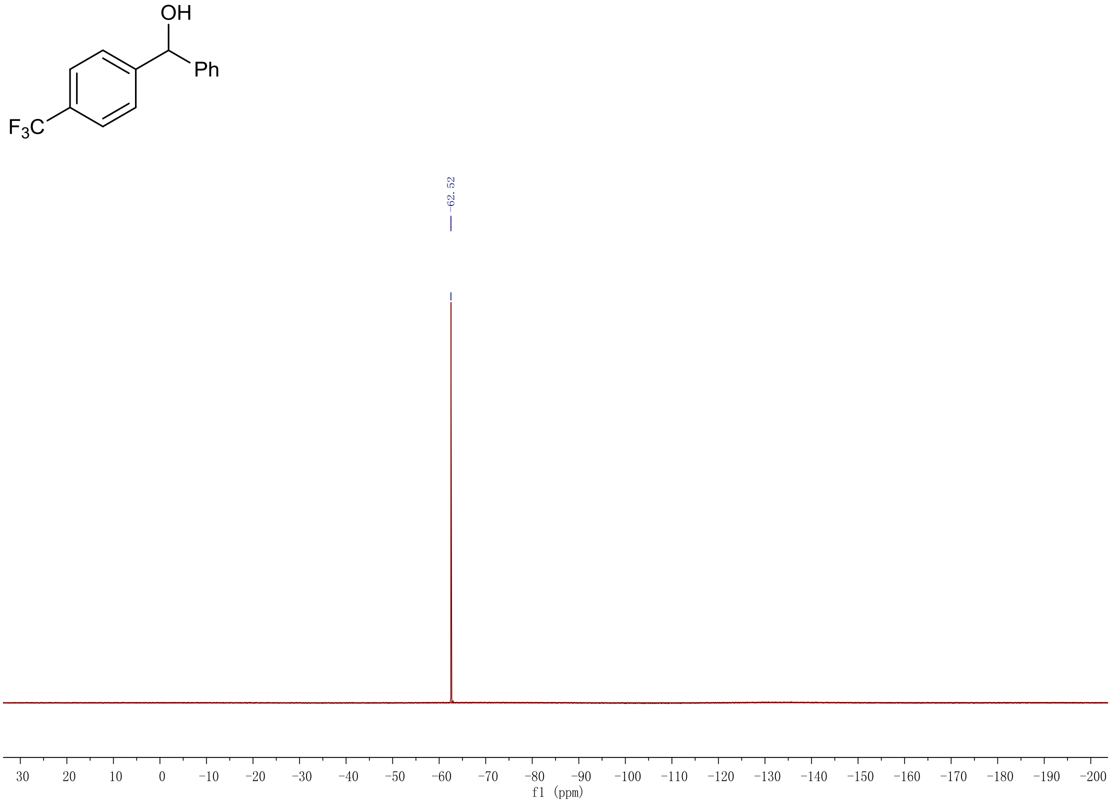

**Supplementary Figure 57. $^1$H NMR of Phenyl(3-(trifluoromethyl)phenyl)-methanol 3ja**

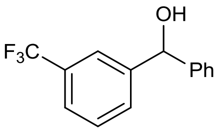


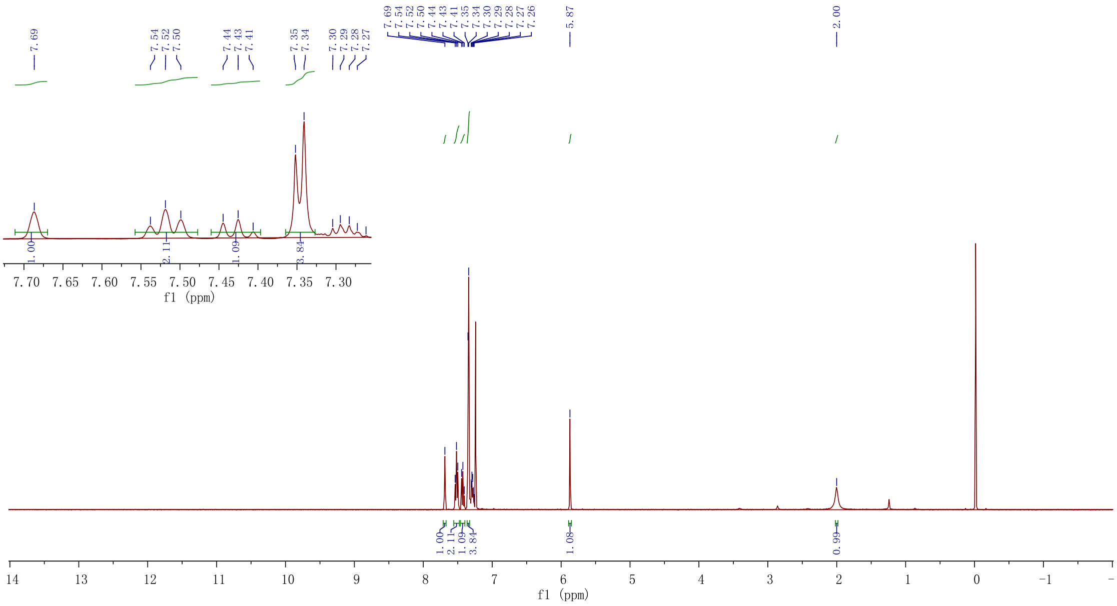


**Supplementary Figure 58. $^{19}$F NMR of Phenyl(3-(trifluoromethyl)phenyl)-methanol 3ja**

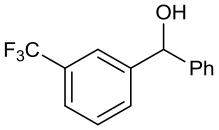


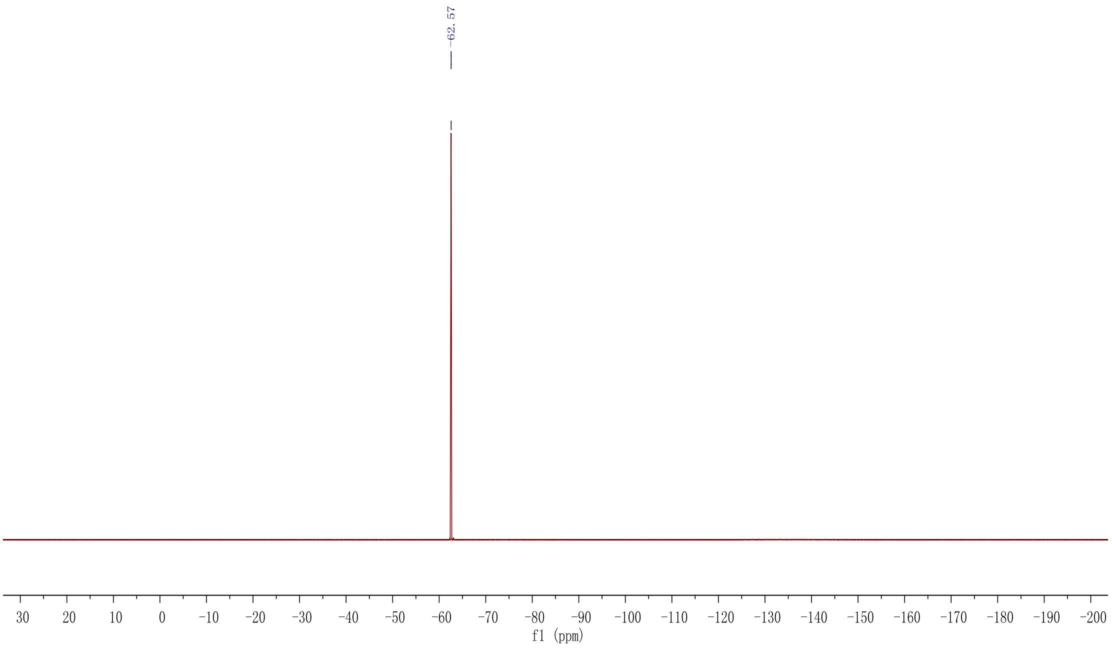

**Supplementary Figure 59. $^{1}$H NMR of Phenyl(2-(trifluoromethyl)phenyl)-methanol 3ka**

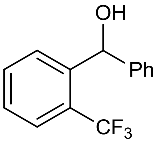


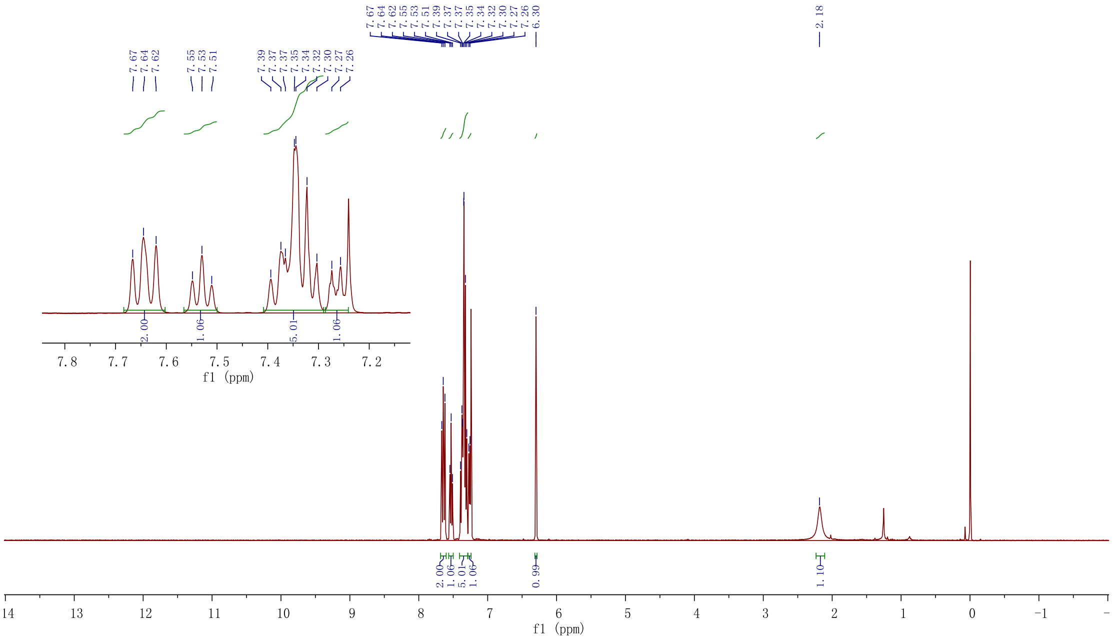


**Supplementary Figure 60. $^{19}$F NMR of Phenyl(2-(trifluoromethyl)phenyl)-methanol 3ka**

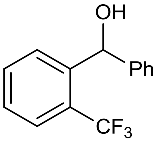


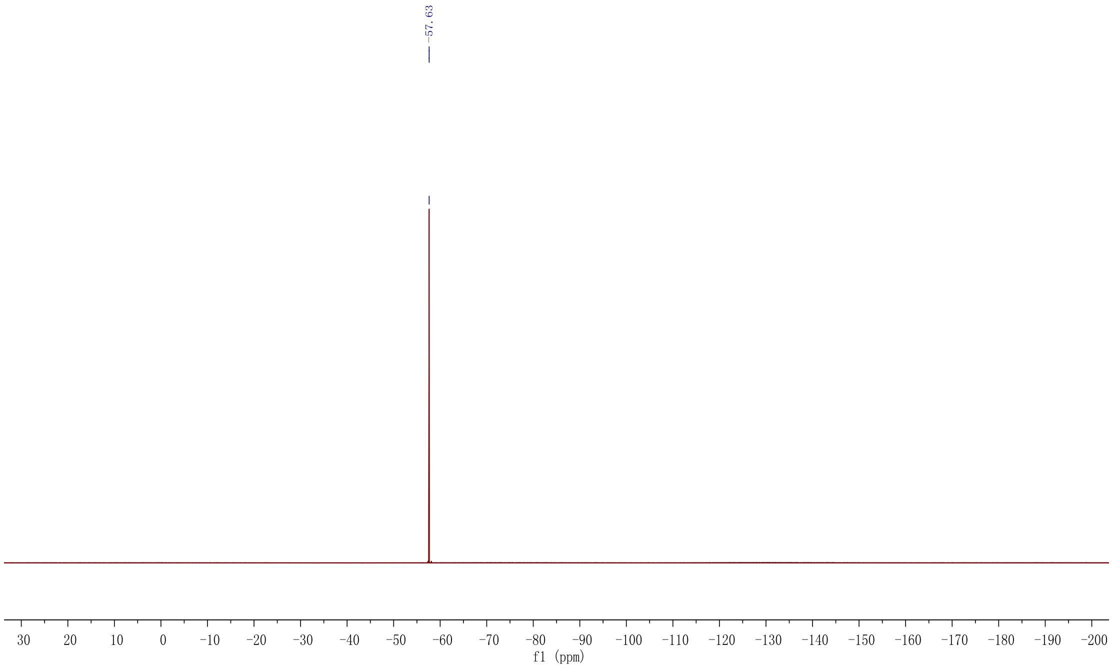

**Supplementary Figure 61. $^1$H NMR of 3-(Hydroxy(phenyl)methyl)benzonitrile 3la**

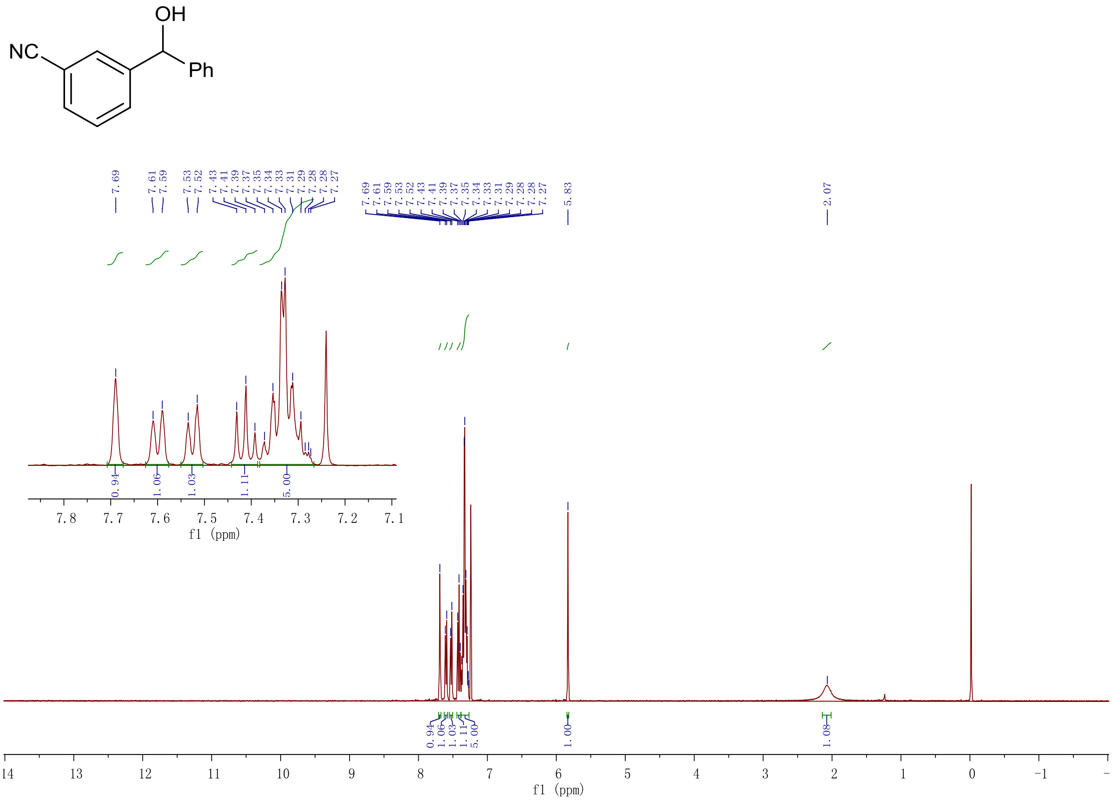


**Supplementary Figure 62. $^1$H NMR of 4-(Hydroxy(phenyl)methyl)benzonitrile 3ma**

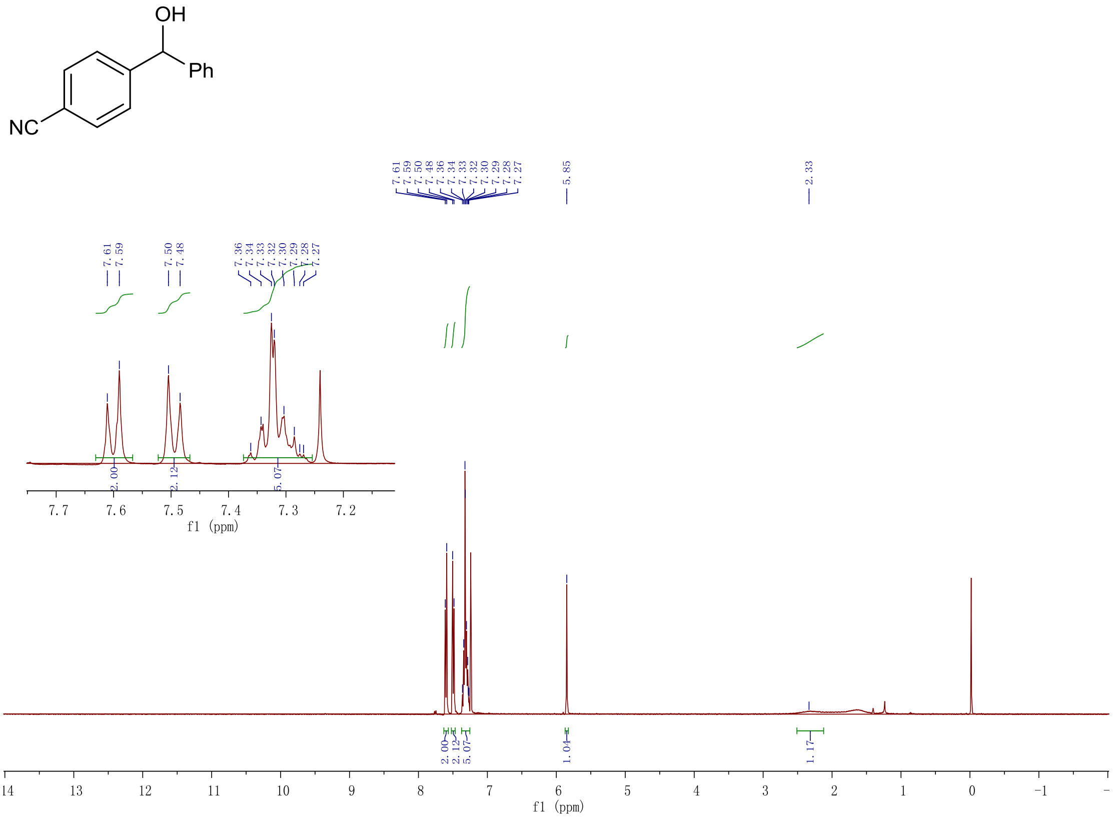

**Supplementary Figure 63. ¹H NMR of Methyl 4-(hydroxy(phenyl)methyl)-benzoate 3na**

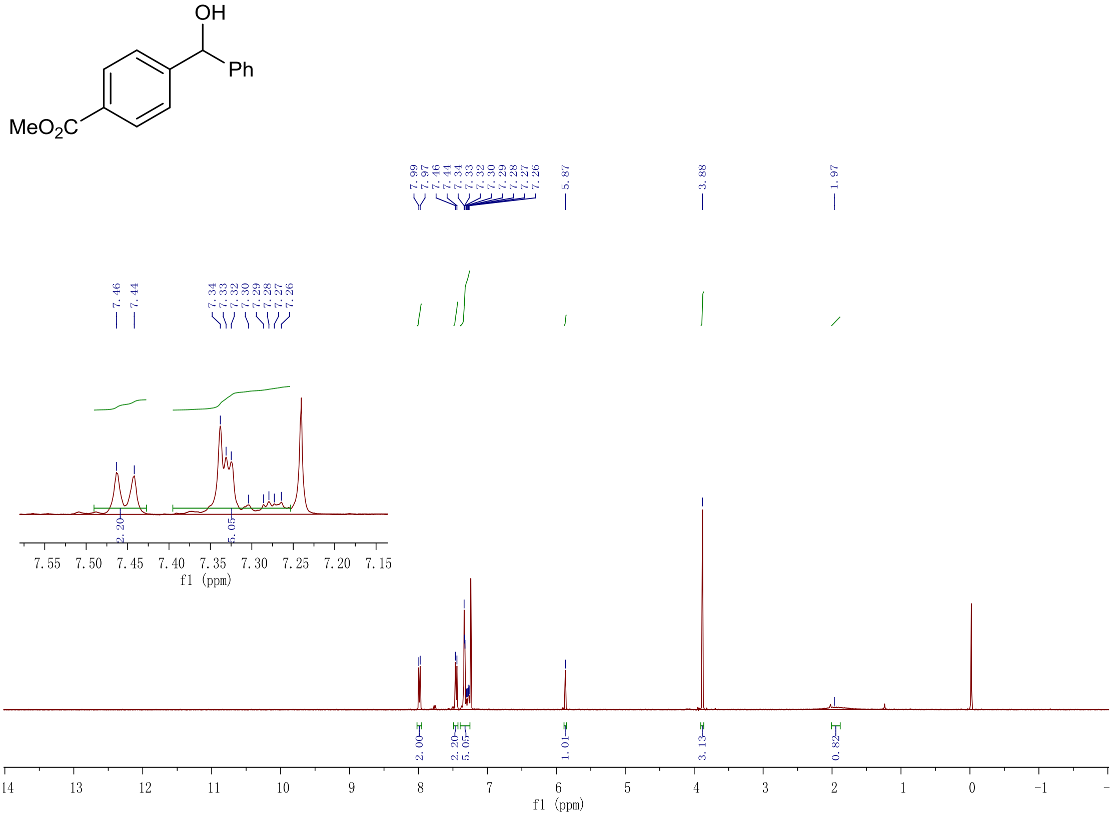


**Supplementary Figure 64. ¹H NMR of [1,1'-Biphenyl]-2-yl(phenyl)methanol 3oa**

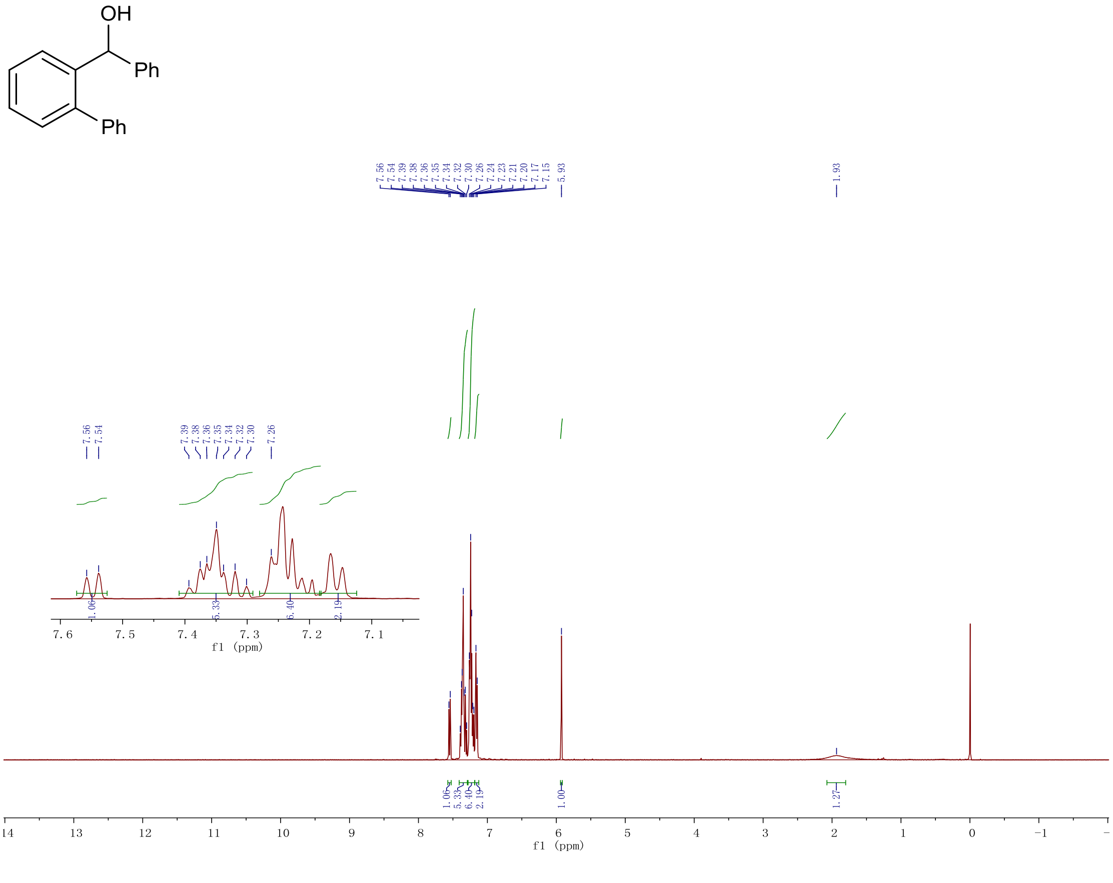

## Supplementary Figure 65. $^1$H NMR of Naphthalen-2-yl(phenyl)methanol 3pa

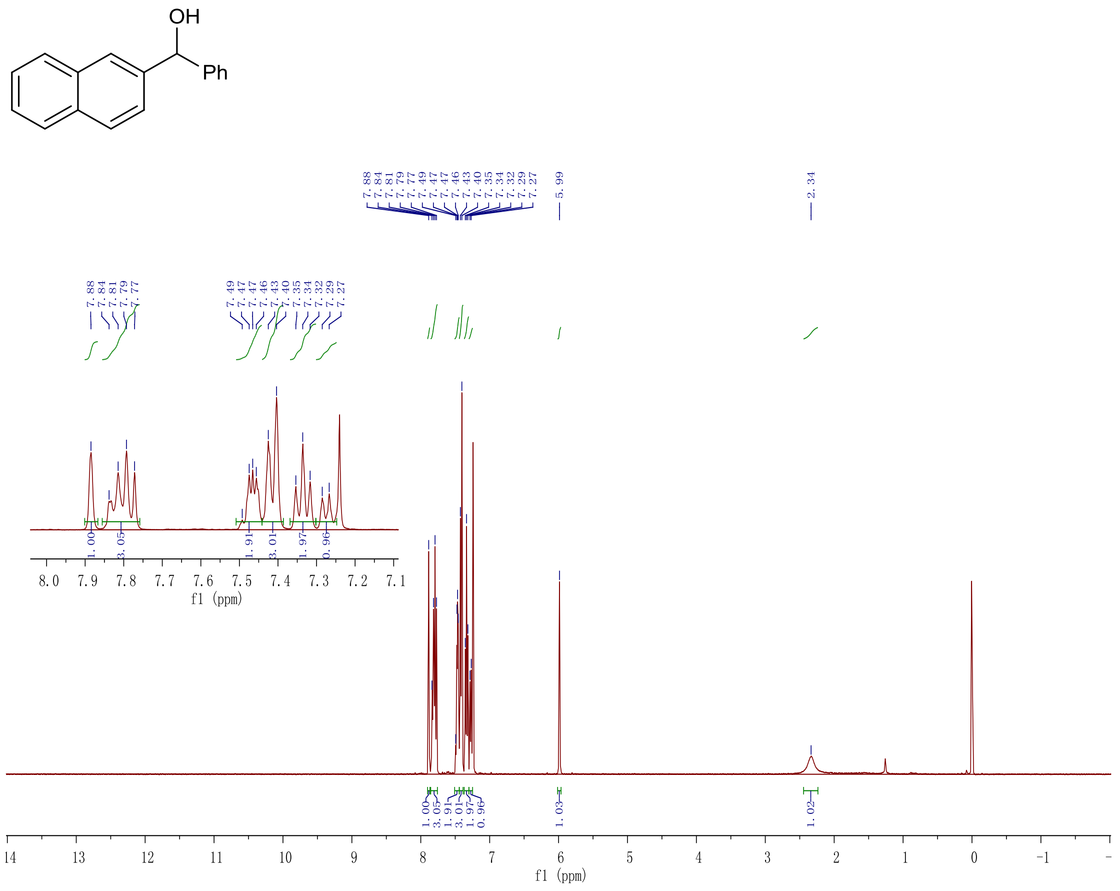

## Supplementary Figure 66. $^1$H NMR of Phenyl(quinolin-3-yl)methanol 3qa

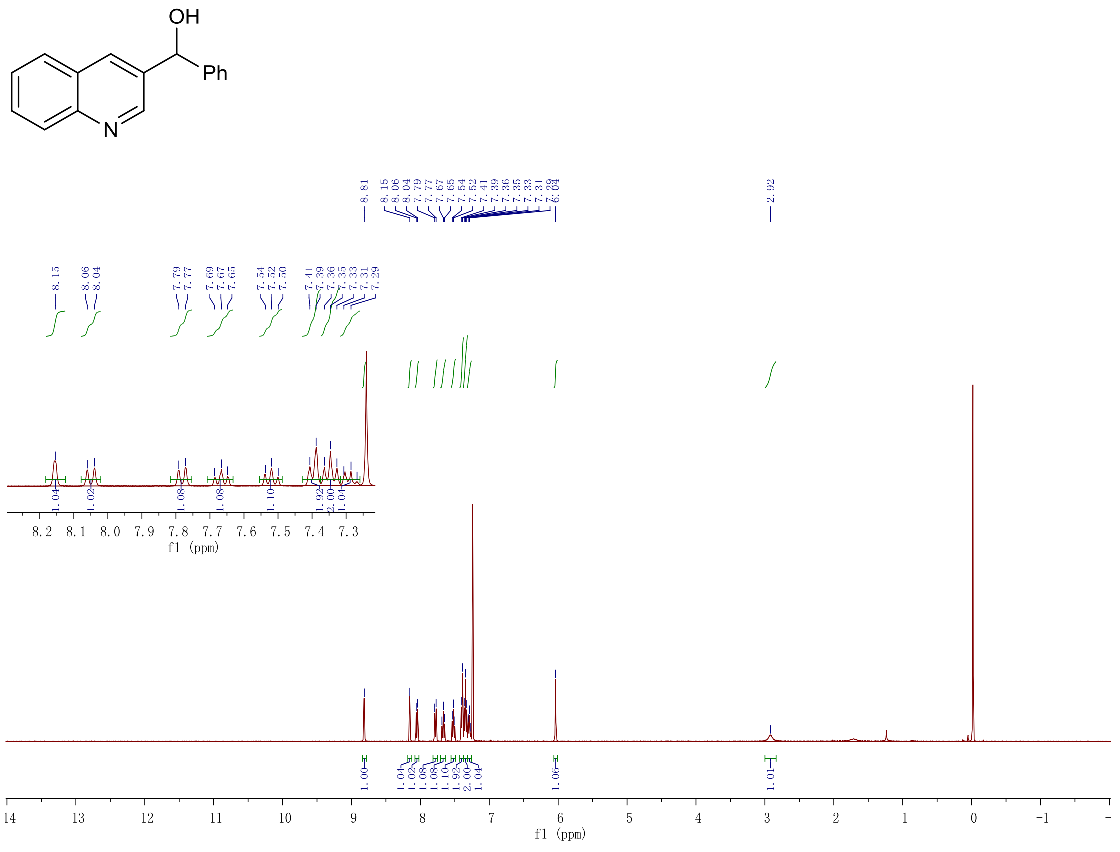

## Supplementary Figure 67. $^{1}$H NMR of Benzo[b]thiophen-2-yl(phenyl)methanol 3ra

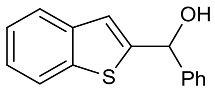


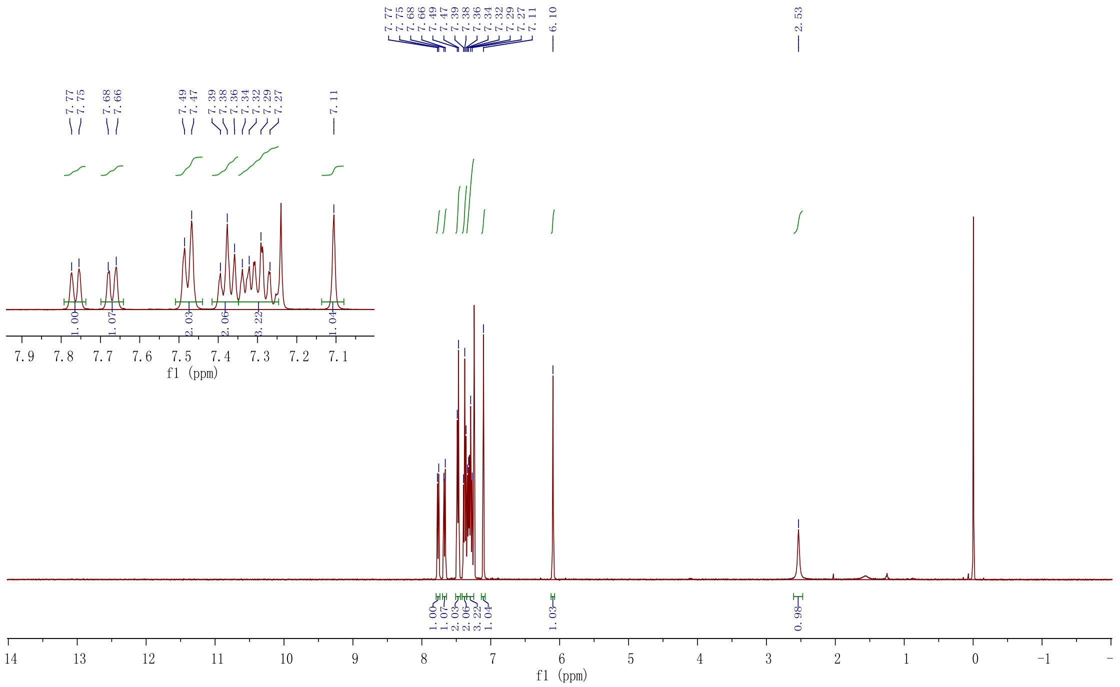


## Supplementary Figure 68. $^{1}$H NMR of Benzofuran-2-yl(phenyl)methanol 3sa

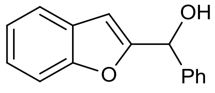


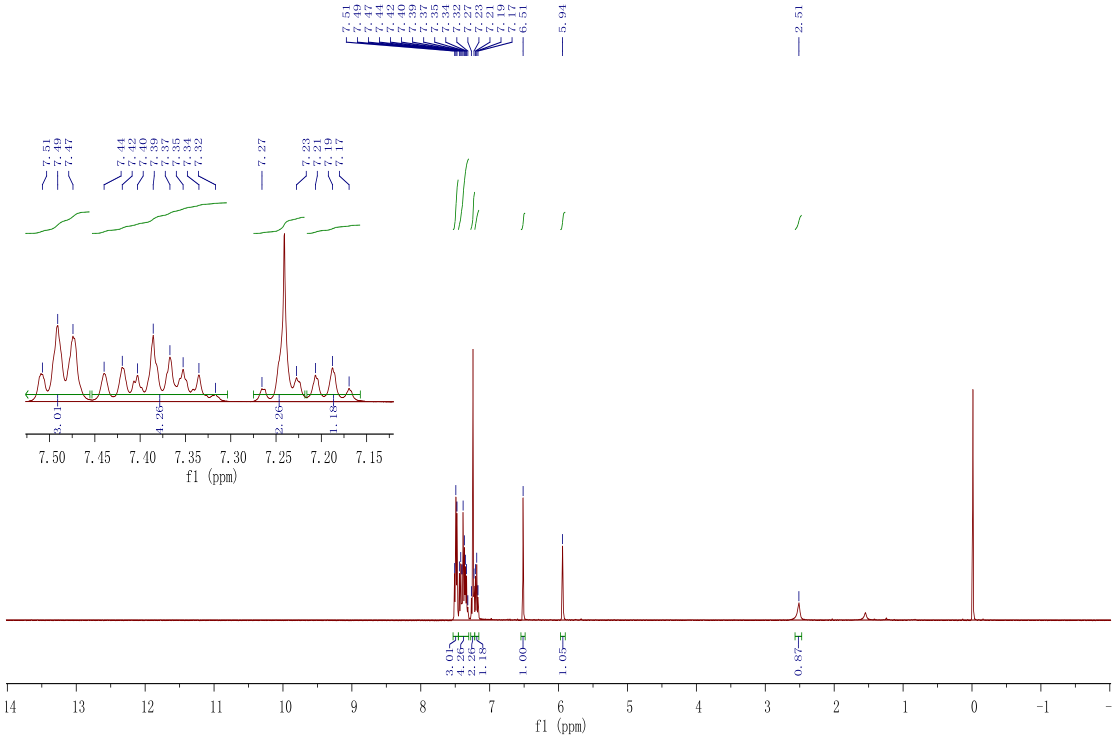

## Supplementary Figure 69. $^{1}H$ NMR of Cyclohexyl(phenyl)methanol 3ta

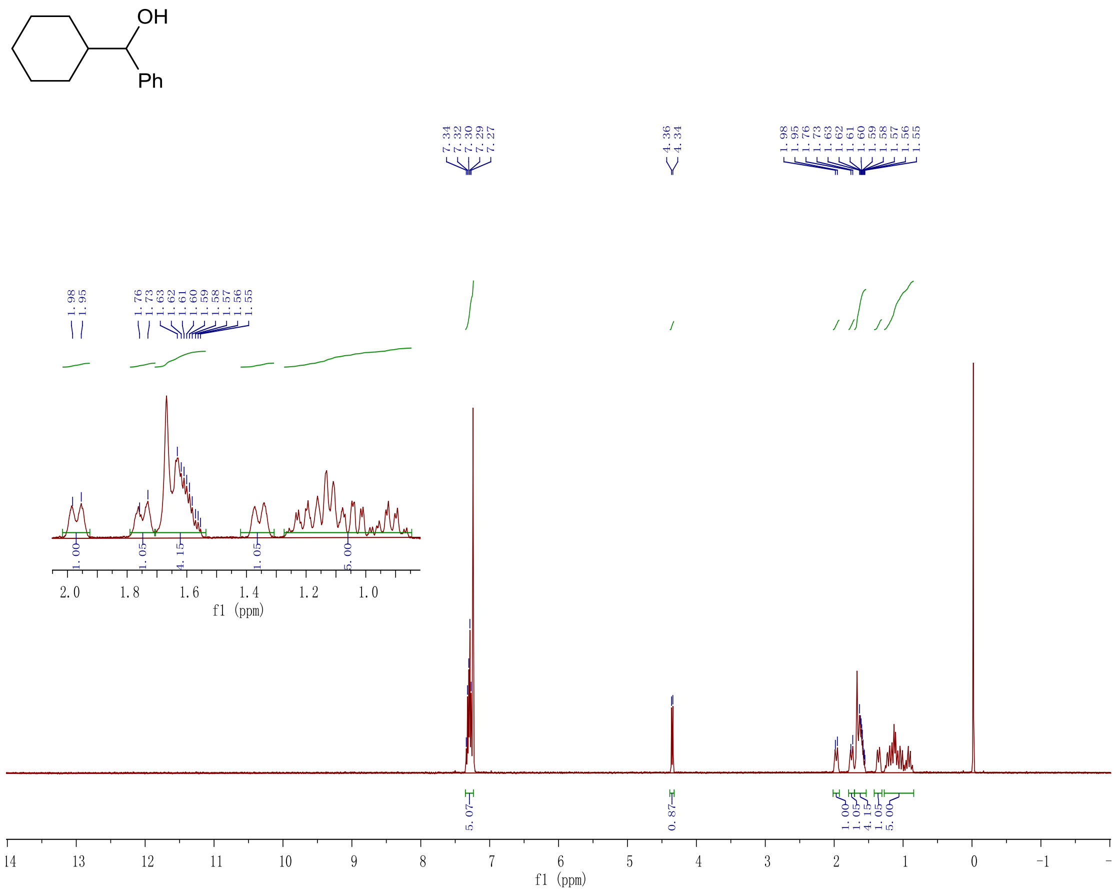


## Supplementary Figure 70. NMR of 1,3-Diphenylpropan-1-ol 3ua

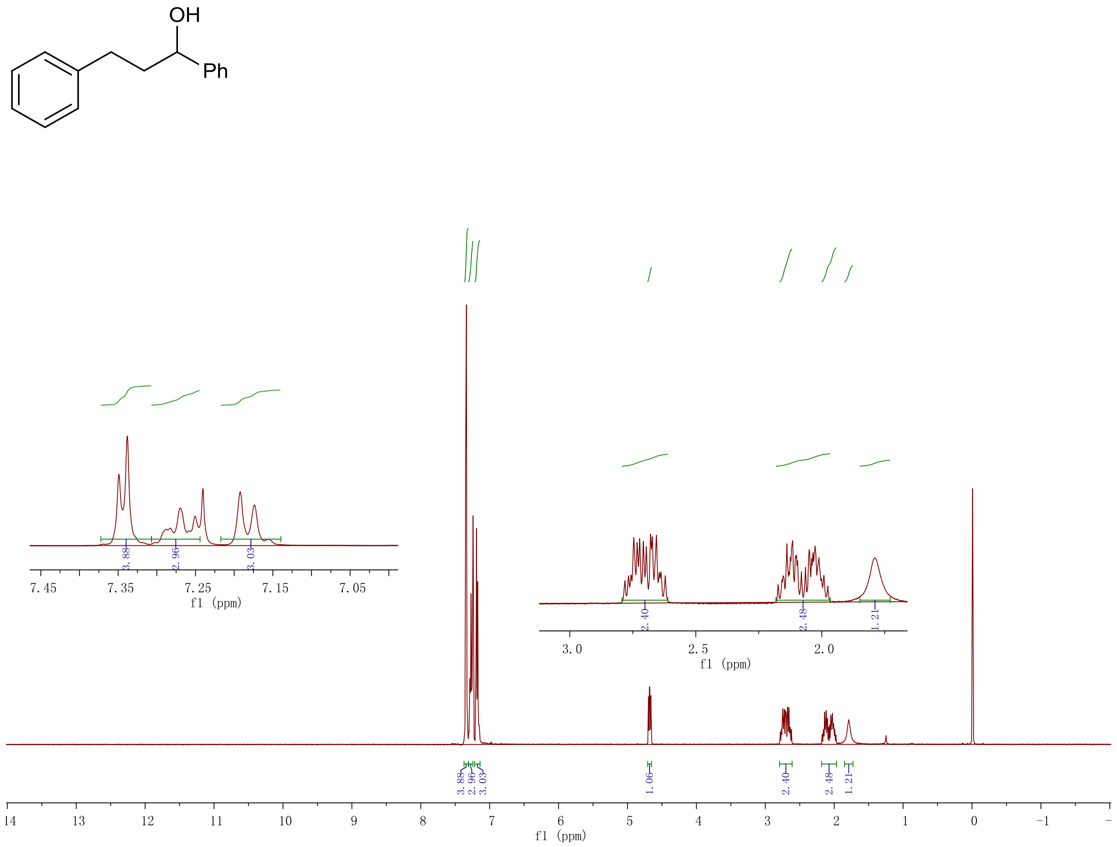

**Supplementary Figure 71 $^{1}$H NMR of 1-([1,1'-Biphenyl]-4-yl)-1-phenylethanol 5a**

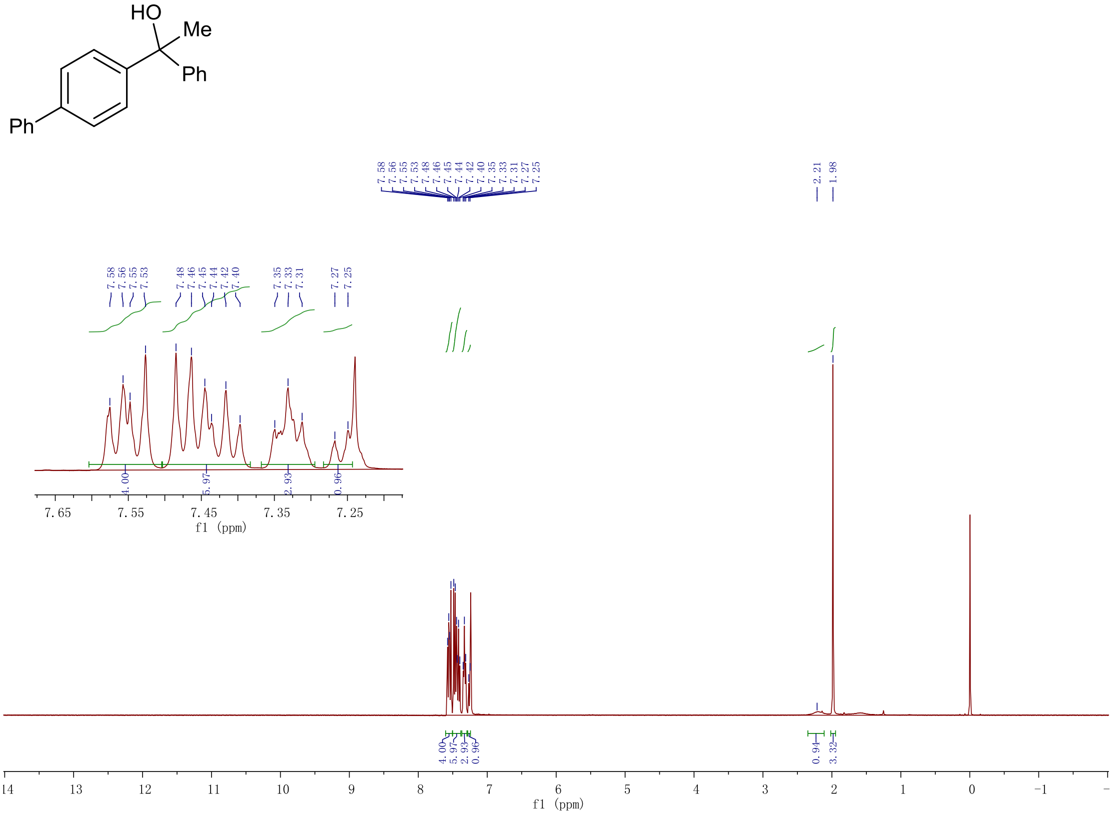


**Supplementary Figure 72 $^{1}$H NMR of 1-(4-Fluorophenyl)-1-phenylethanol 5b**

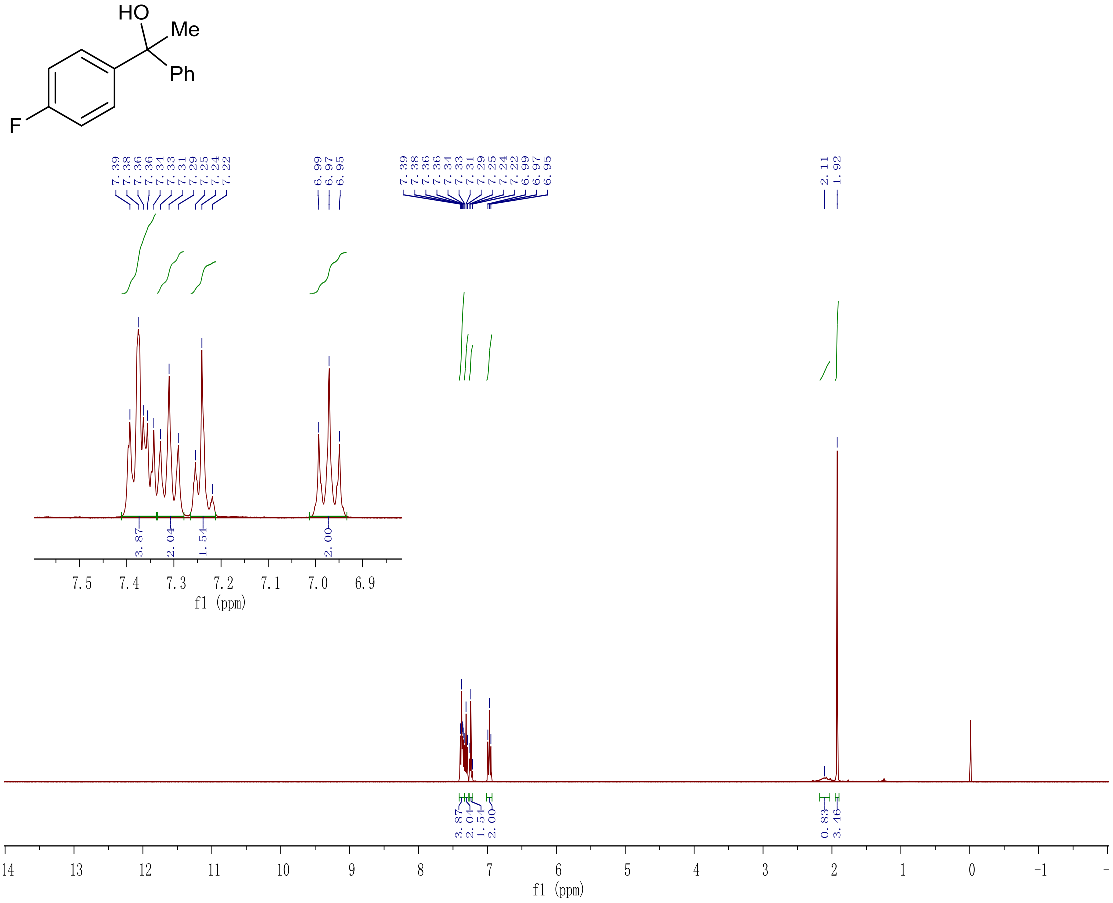

## Supplementary Figure 73 $^{19}$F NMR of 1-(4-Fluorophenyl)-1-phenylethanol 5b

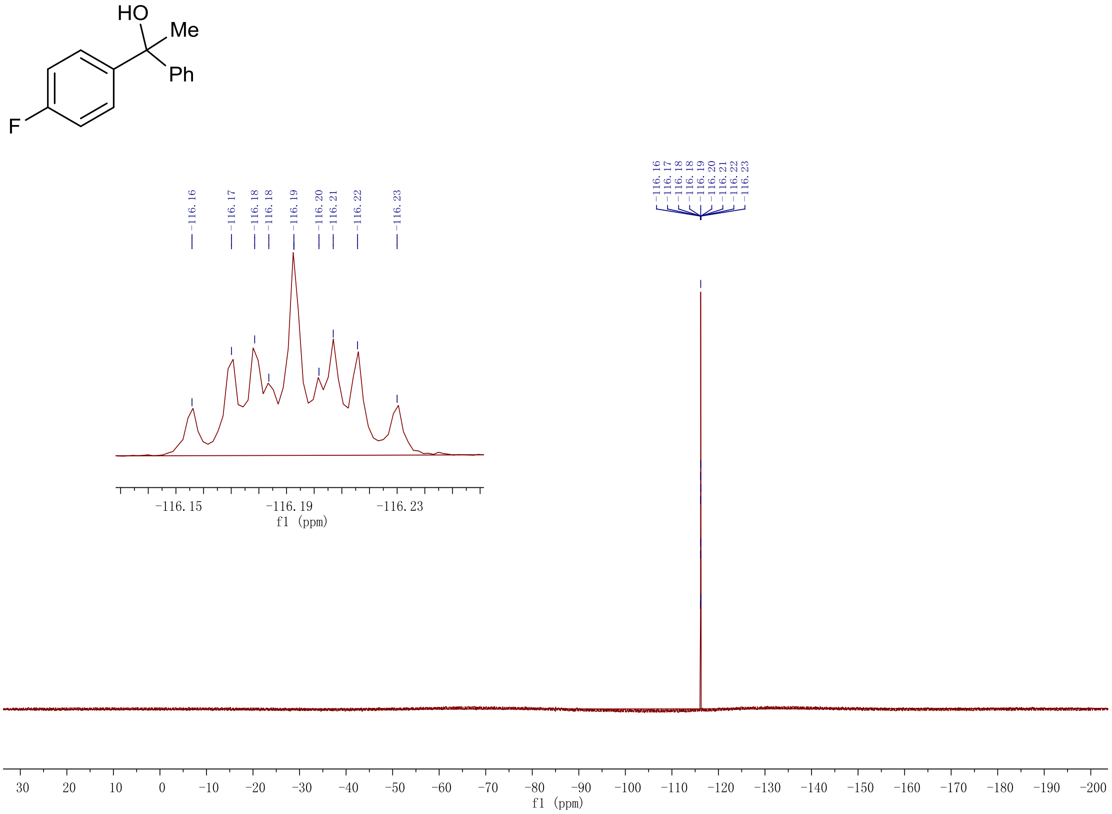

## Supplementary Figure 74 $^{1}$H NMR of 4-(1-Hydroxy-1-phenylethyl)benzonitrile 5c

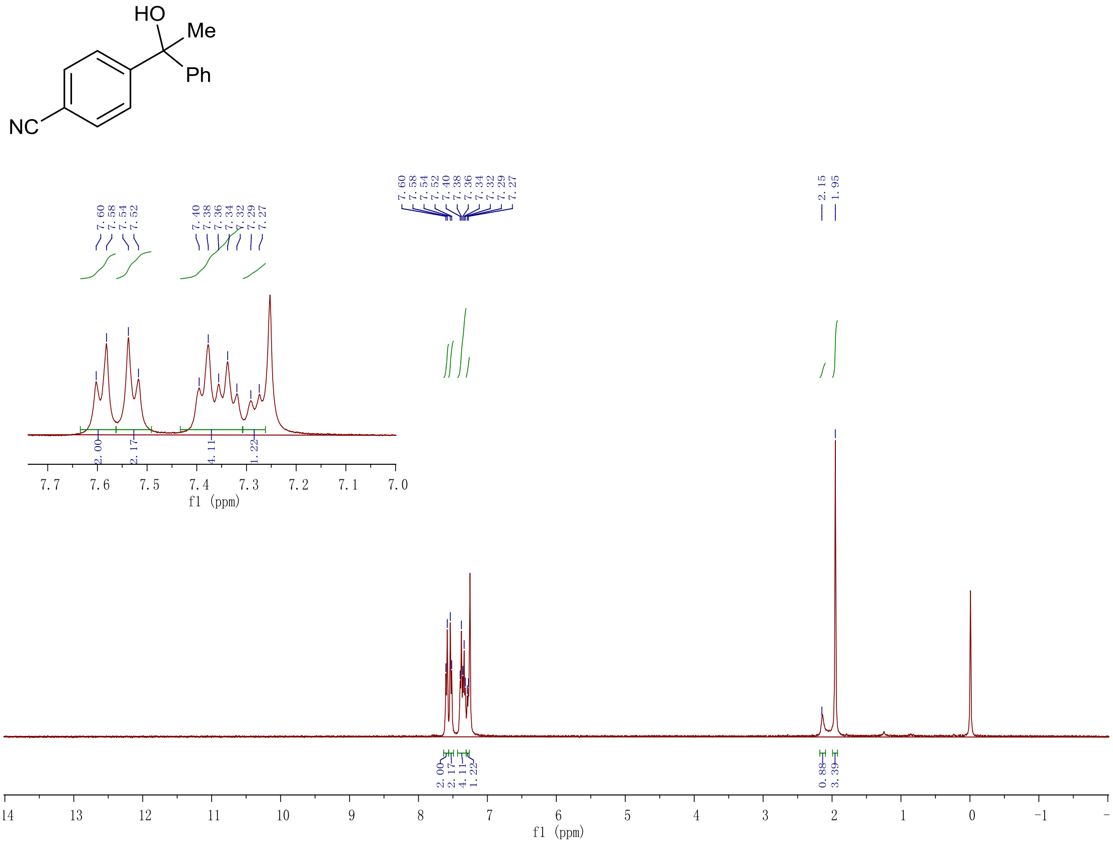

**Supplementary Figure 75 $^{1}$H NMR of Phenyl-1-(4-(trifluoromethyl)phenyl)-ethanol 5d**

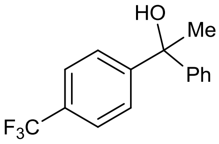


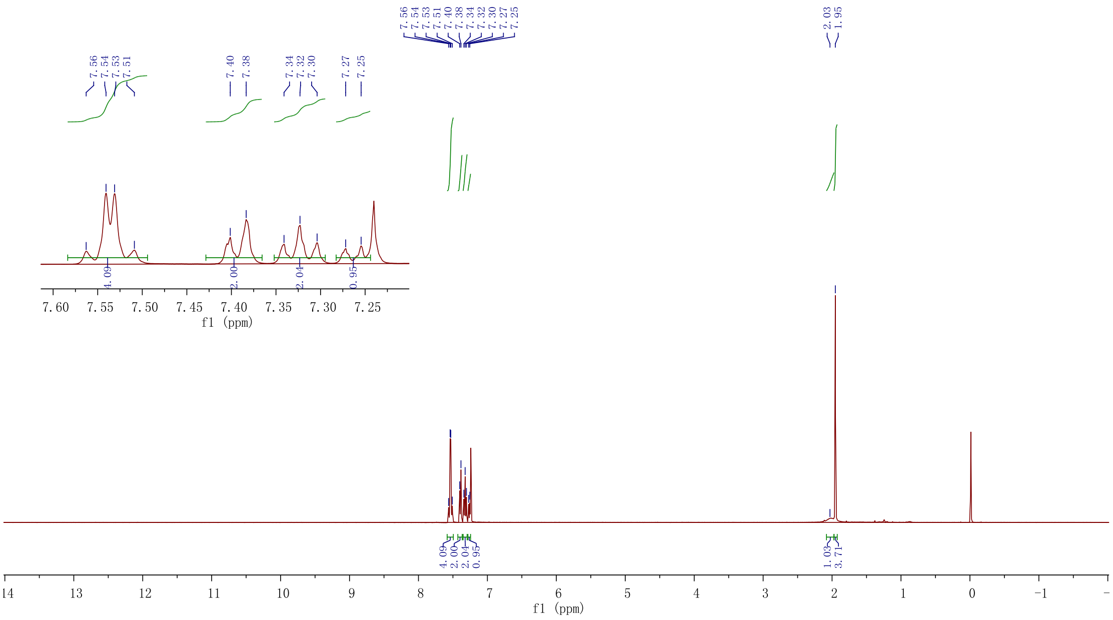


**Supplementary Figure 76 $^{19}$F NMR of Phenyl-1-(4-(trifluoromethyl)phenyl)-ethanol 5d**

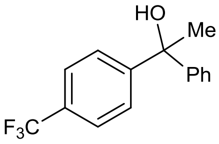


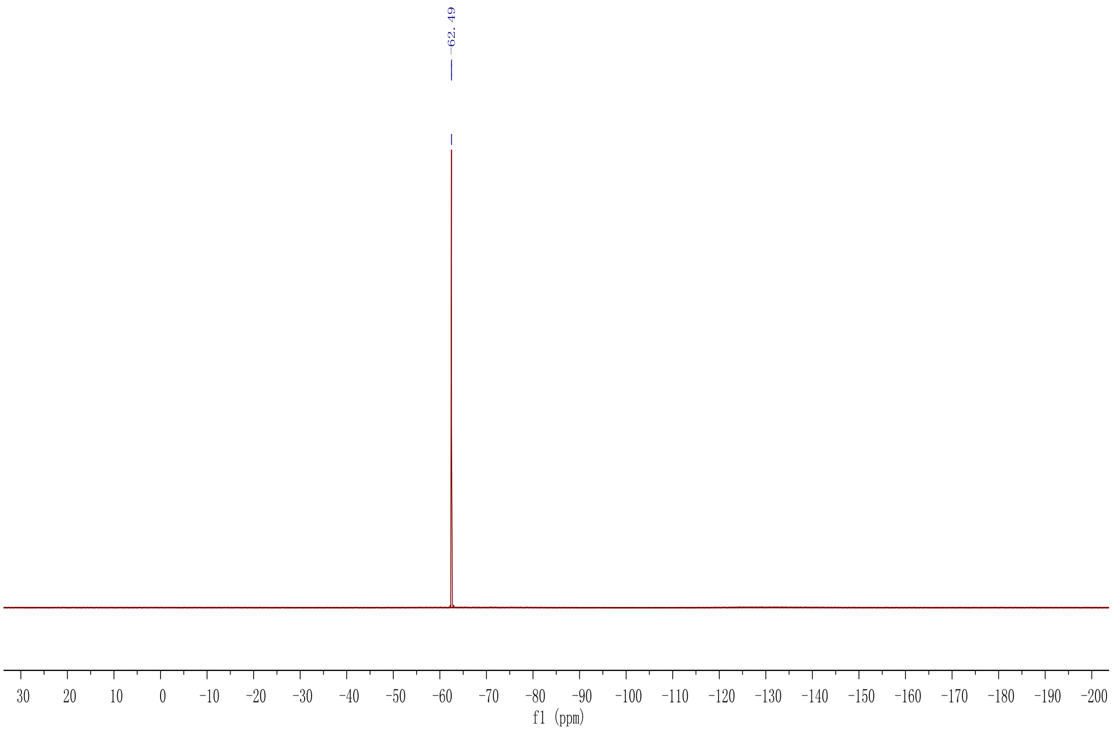

**Supplementary Figure 77 $^{1}H$ NMR of Phenyl-1-(4-(trifluoromethyl)phenyl)-propan-1-ol 5e**

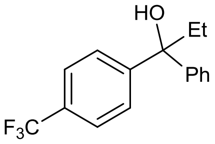


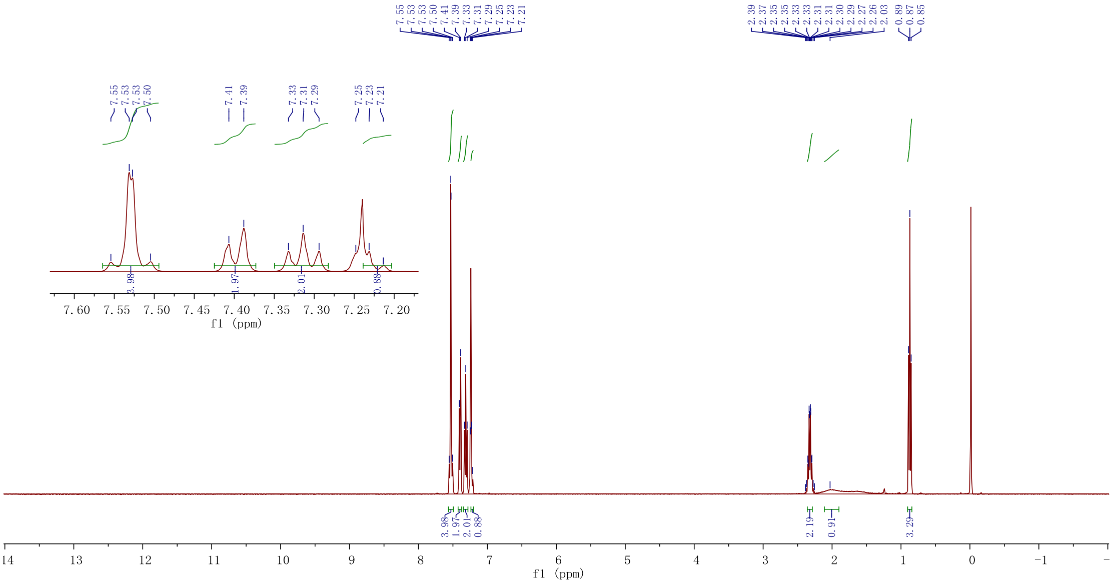

**Supplementary Figure 78 $^{19}F$ NMR of Phenyl-1-(4-(trifluoromethyl)phenyl)-propan-1-ol 5e**

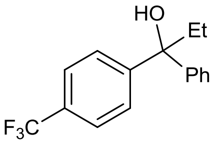


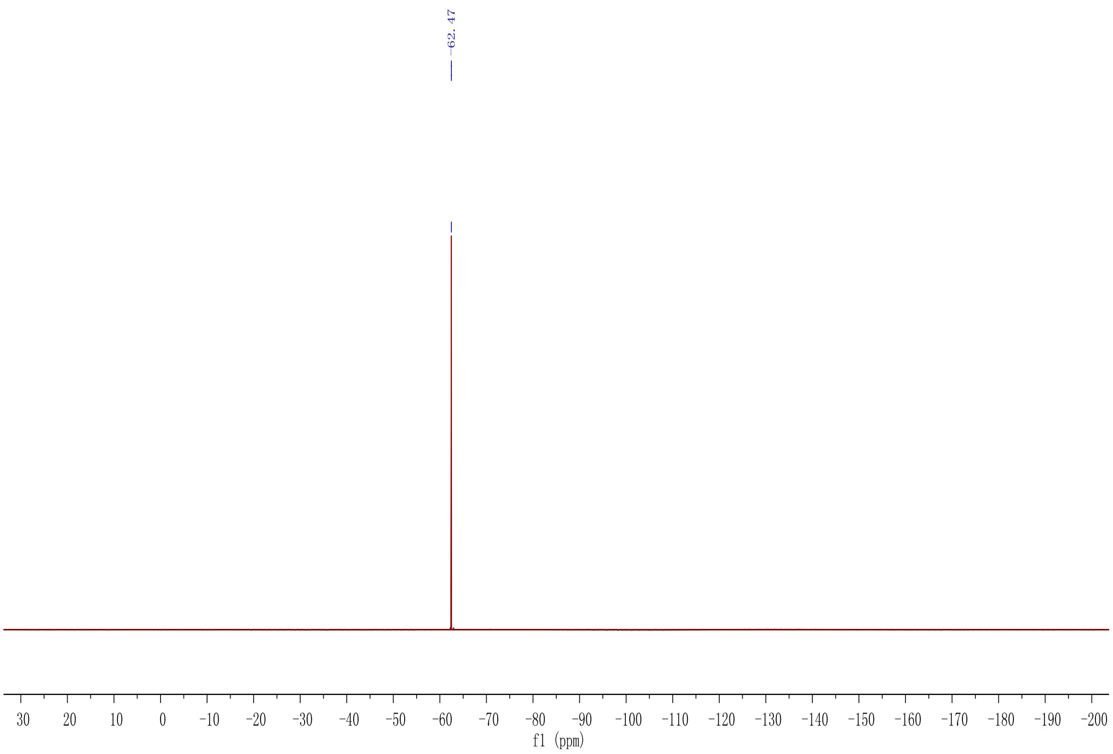

## Supplementary Figure 79 $^1$H NMR of 2-Methyl-1,1-diphenylpropan-1-ol 5f

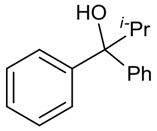


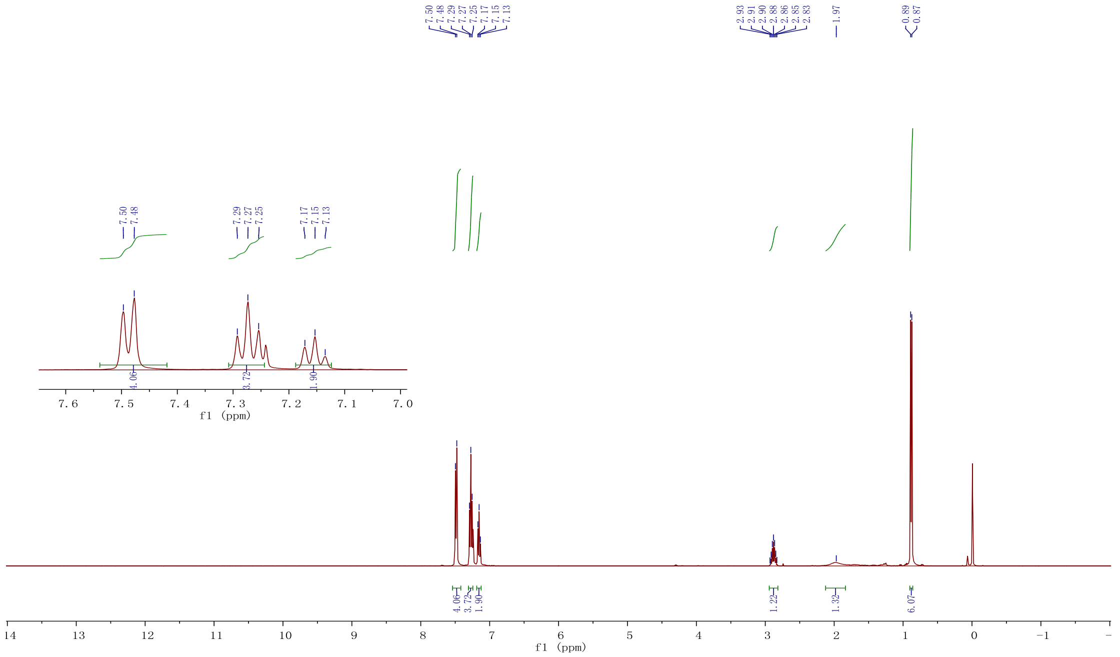


## Supplementary Figure 80 $^1$H NMR of Pyclohexyldiphenylmethanol 5g

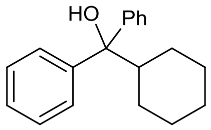


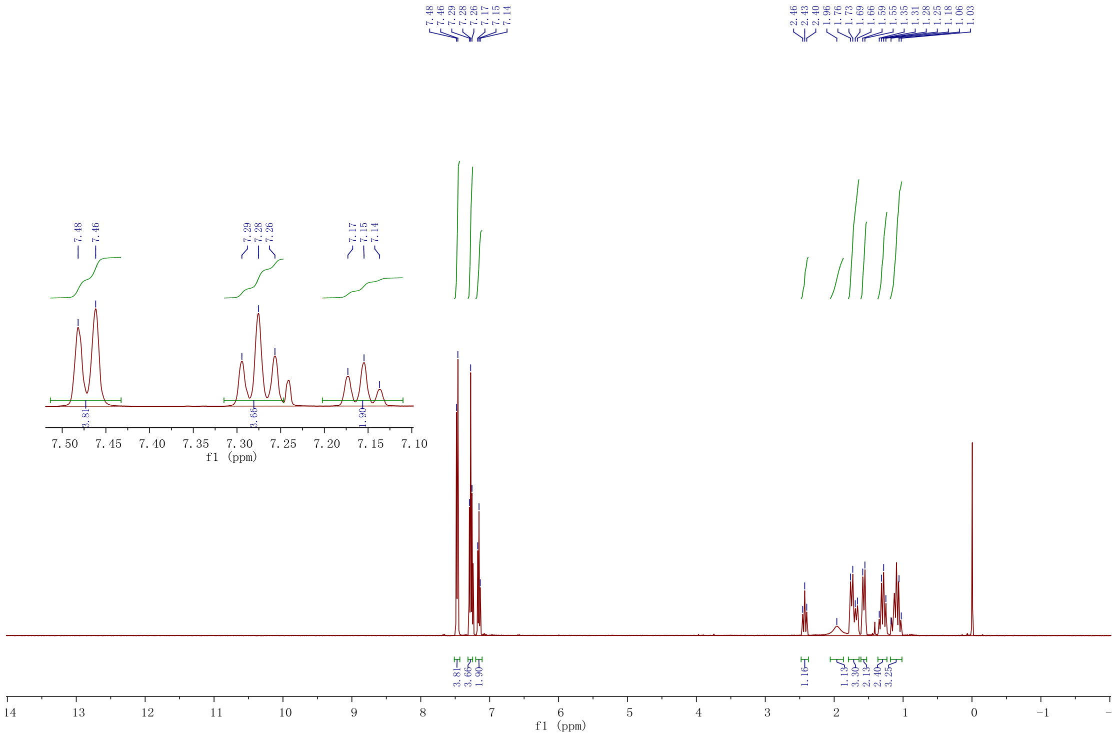

## Supplementary Figure 81 $^{1}$H NMR of 2,2-Dimethyl-1,1-diphenylpropan-1-ol 5h

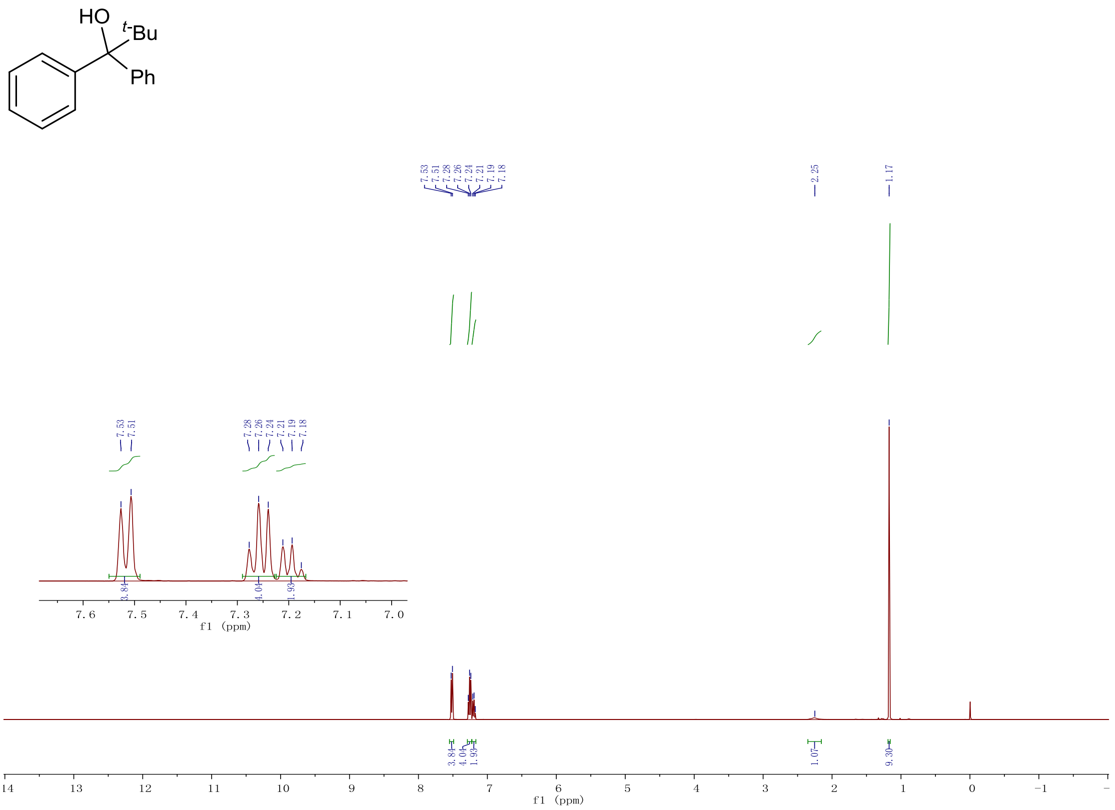


## Supplementary Figure 82 $^{1}$H NMR of 1,1,2-Triphenylethanol 5i

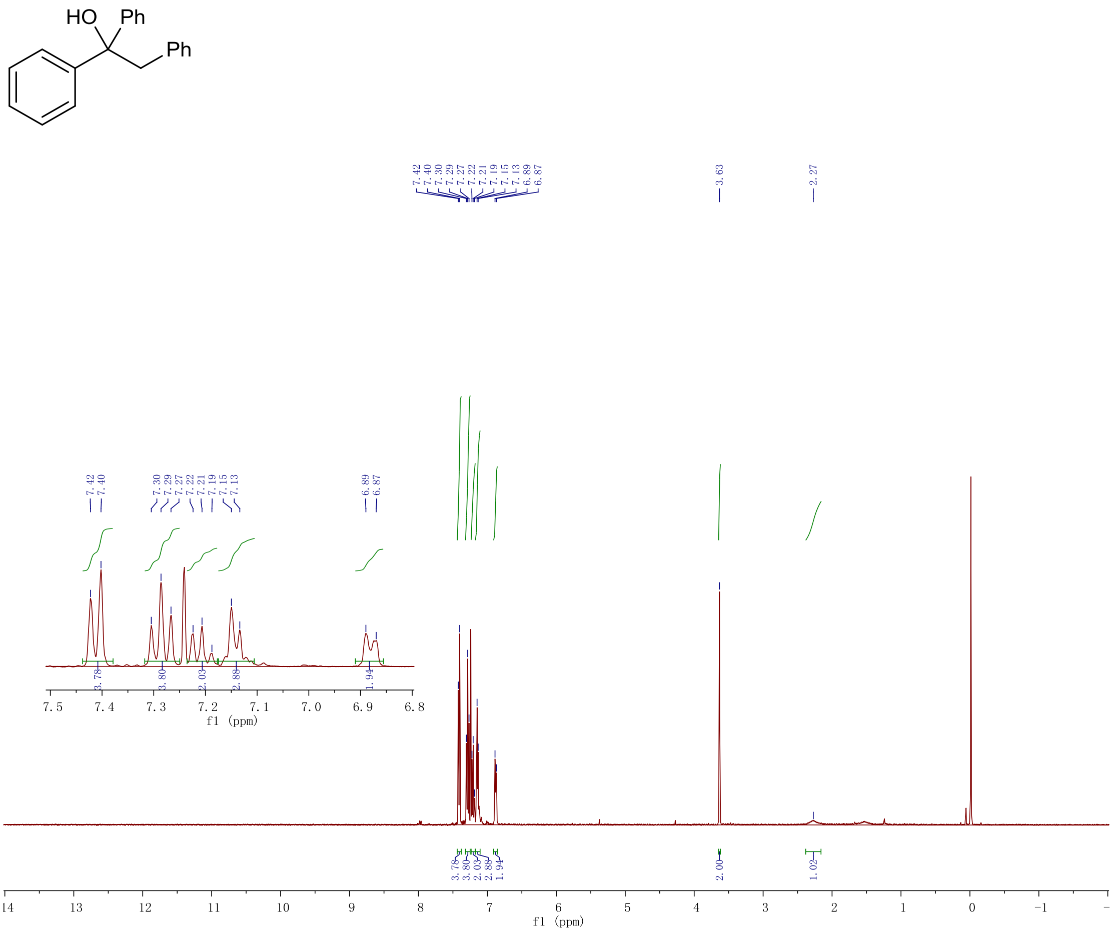

**Supplementary Figure 83 $^{1}H$ NMR of Diphenyl(4-(trifluoromethyl)phenyl)-methanol 5j**

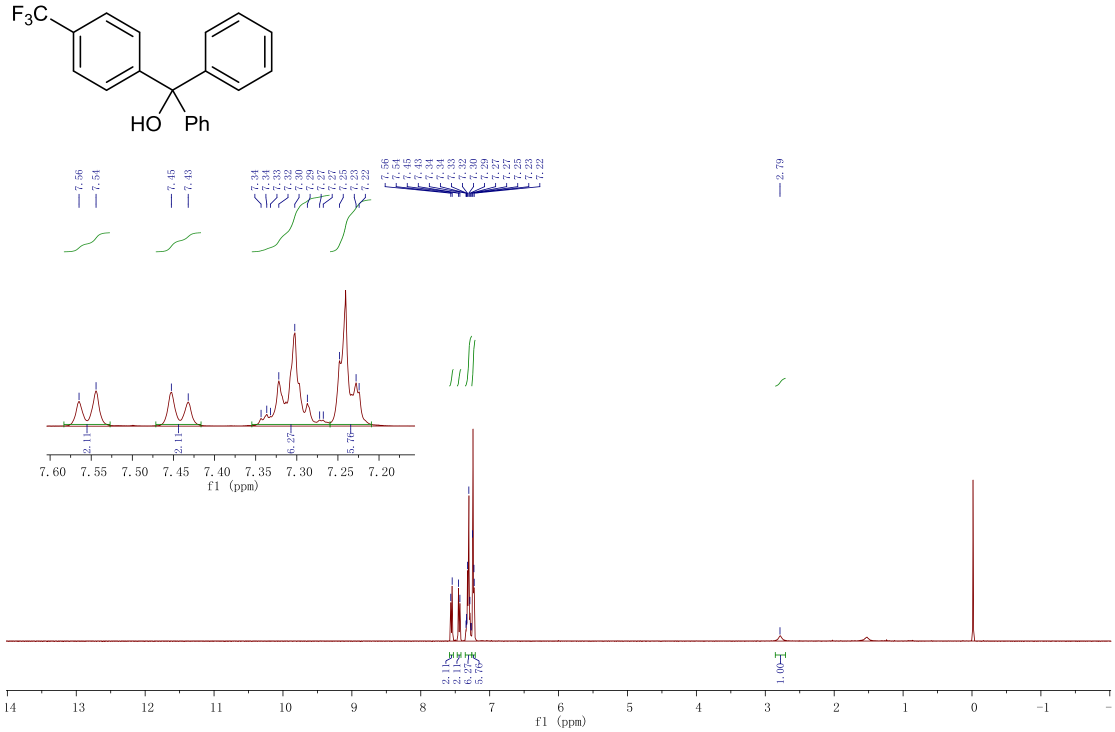


**Supplementary Figure 84 $^{19}F$ NMR of Diphenyl(4-(trifluoromethyl)phenyl)-methanol 5j**

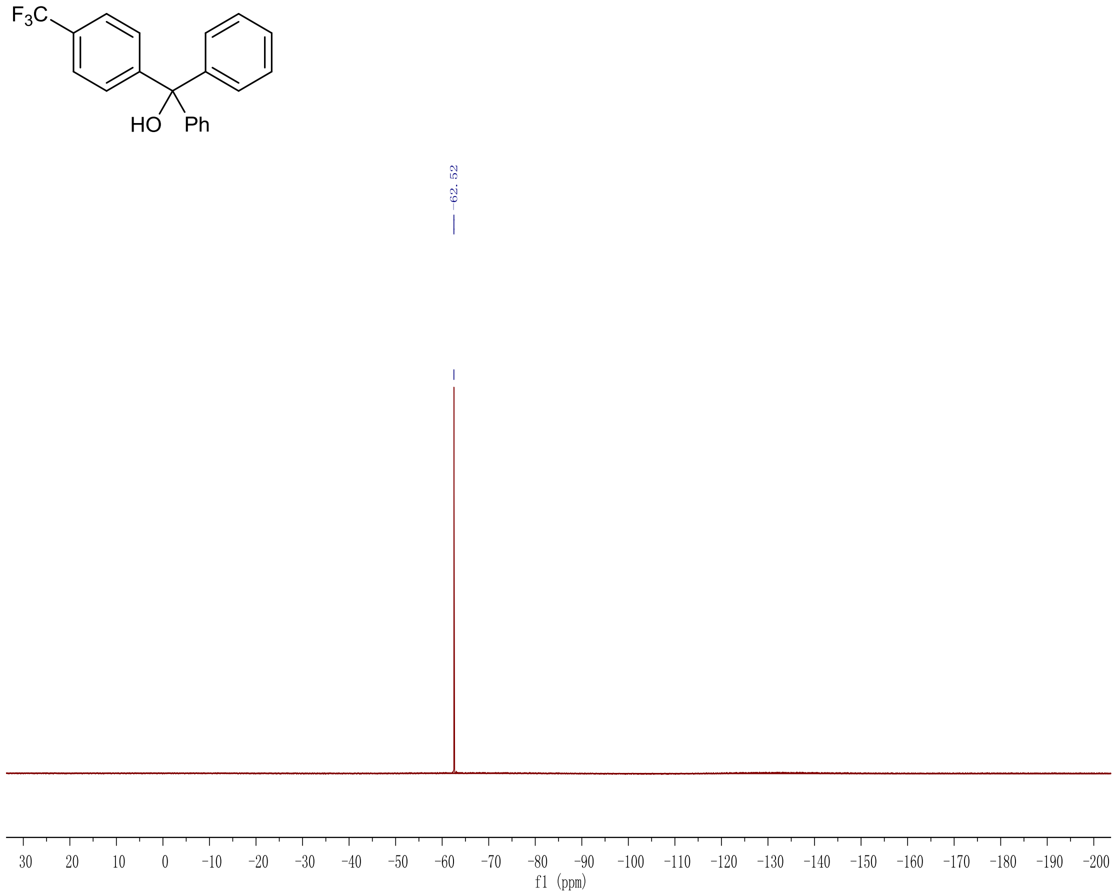

## Supplementary Figure 85 $^{13}$C NMR of Diphenyl(4-(trifluoromethyl)phenyl) -methanol 5j

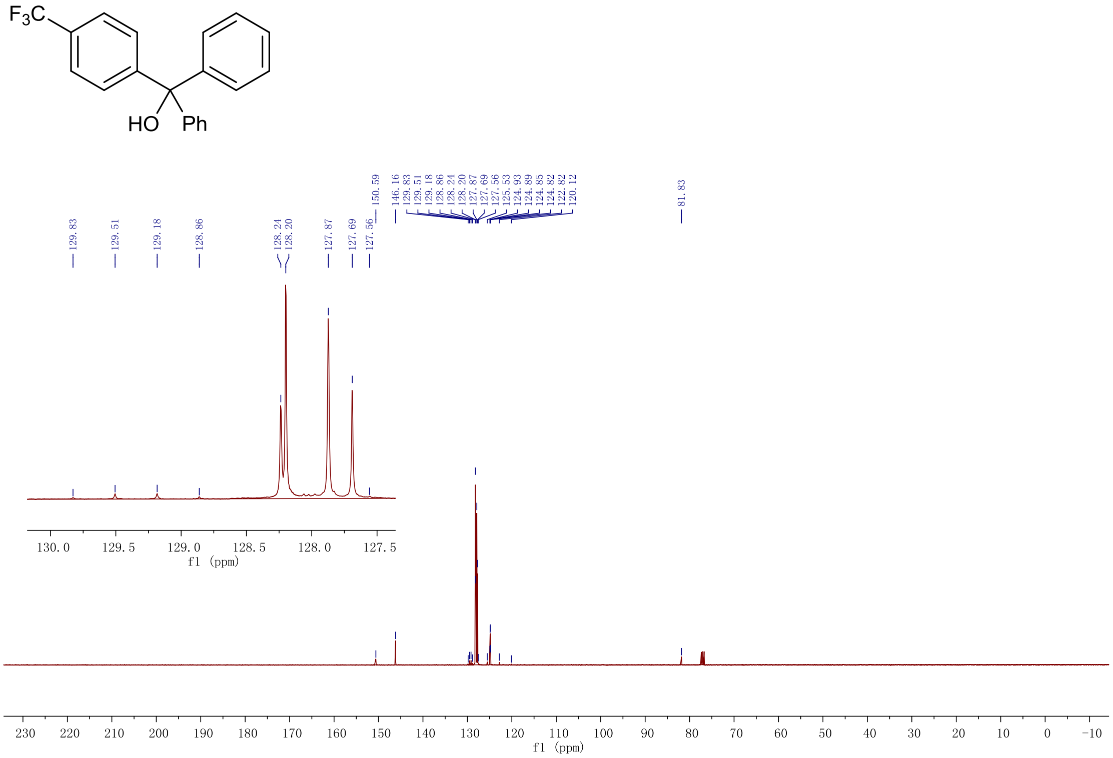


## Supplementary Figure 86 $^{1}$H NMR of Ethyl 2-hydroxy-2,2-diphenylacetate 5k

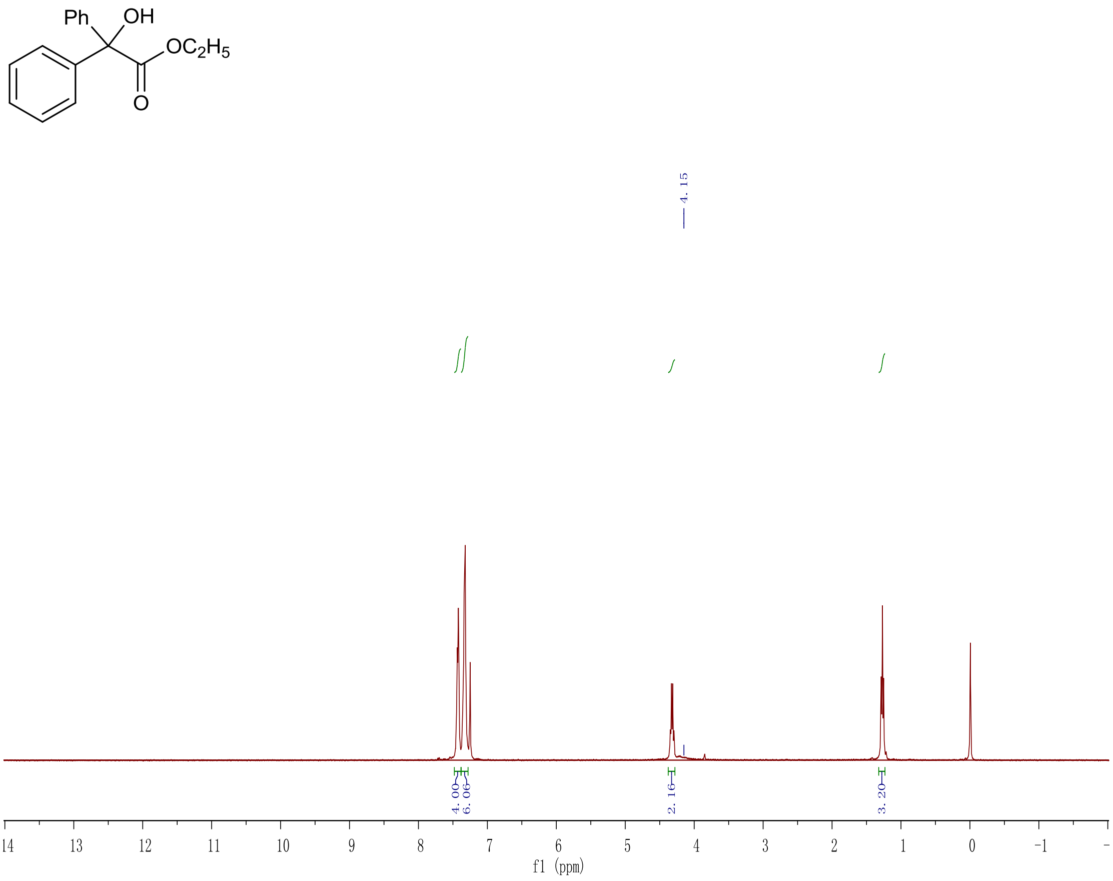

**Supplementary Figure 87 $^{1}$H NMR of 1-(Naphthalen-2-yl)-1-phenylethanol 5l**

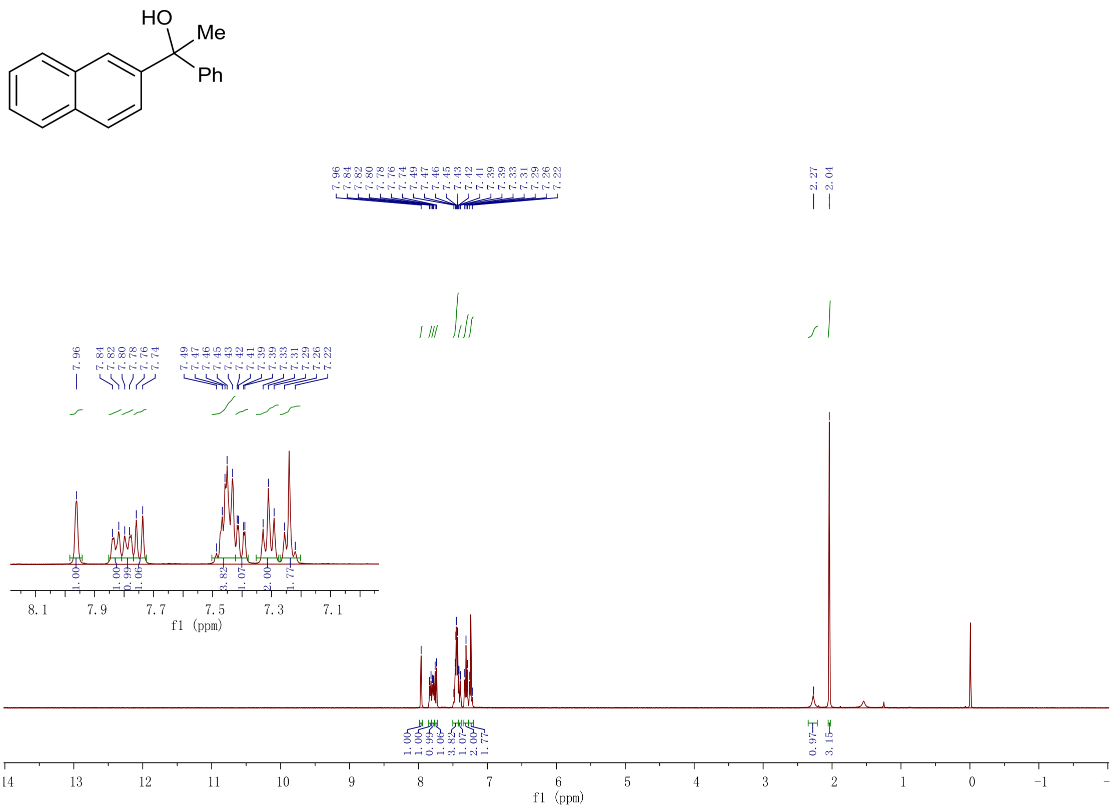


**Supplementary Figure 88 $^{1}$H NMR of 1-Phenylcyclopentanol 5m**

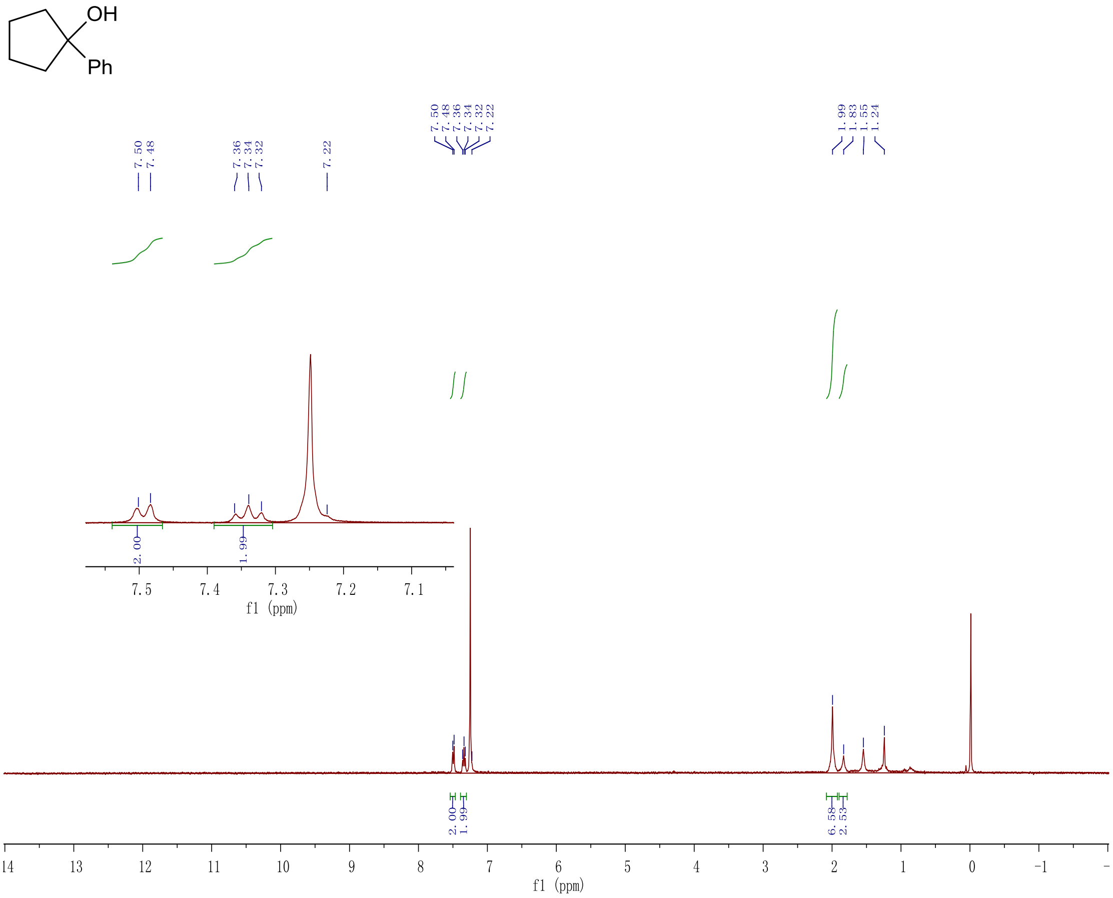

## Supplementary Figure 89 $^{1}$H NMR of 2,4-Diphenylbutan-2-ol 5n

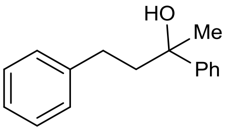

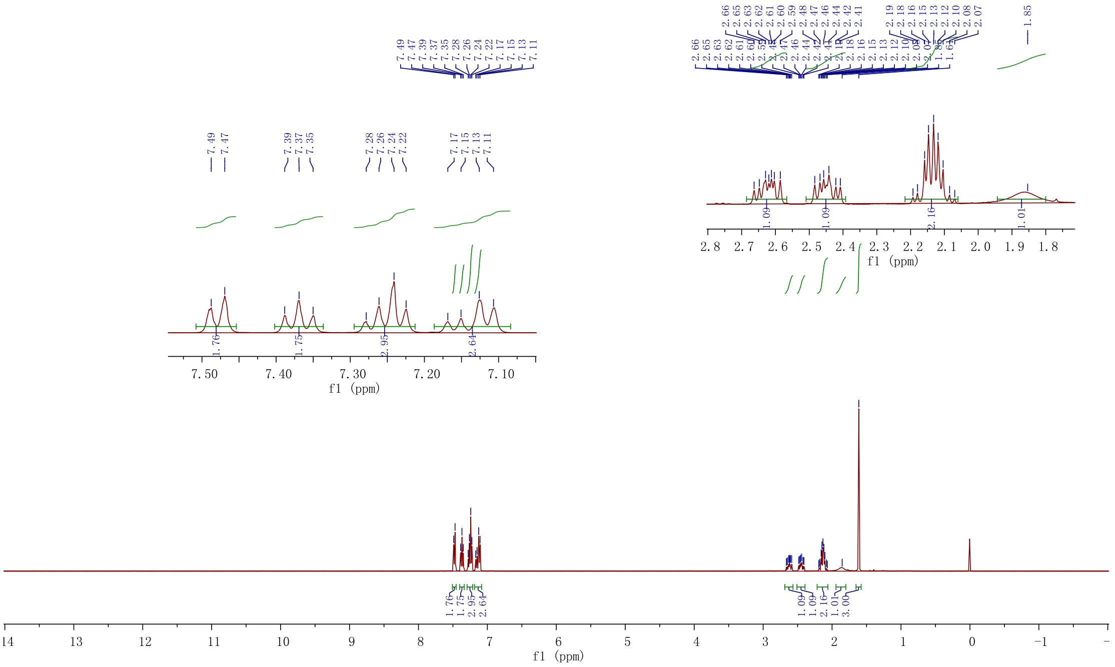

## Supplementary Figure 90 $^{1}$H NMR of (*E*)-*N*-Benzylidene-4-methylbenzenesulfon-amide 6a

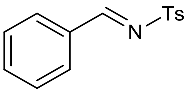

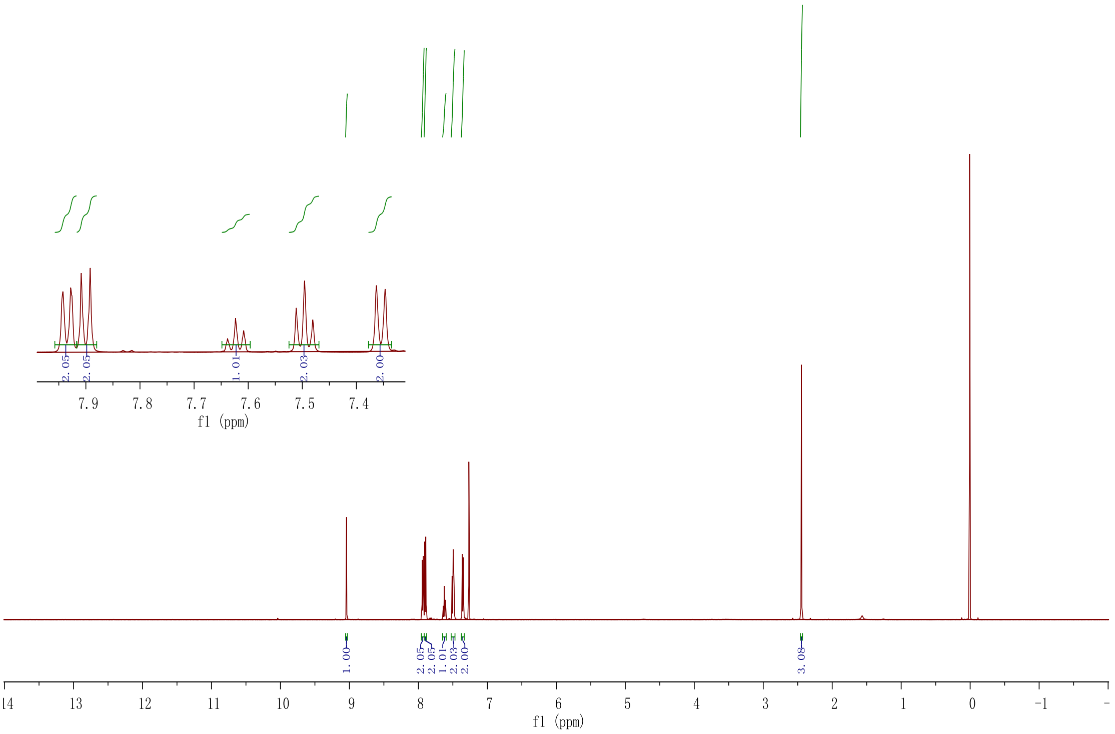

**Supplementary Figure 91 $^{1}$H NMR of (*E*)-*N*-([1,1'-Biphenyl]-4-ylmethylene)-4-methylbenzenesulfonamide 6b**

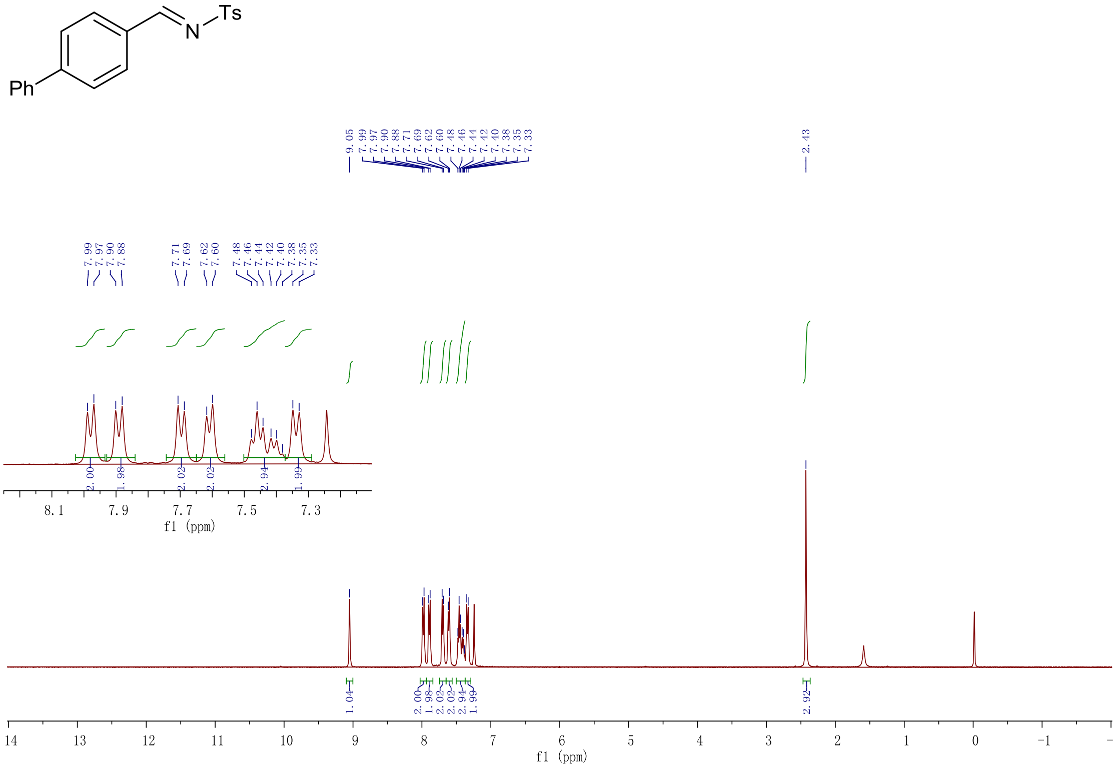


**Supplementary Figure 92 $^{1}$H NMR of (*E*)-4-Methyl-*N*-(4-methylbenzylidene) -benzenesulfonamide 6c**

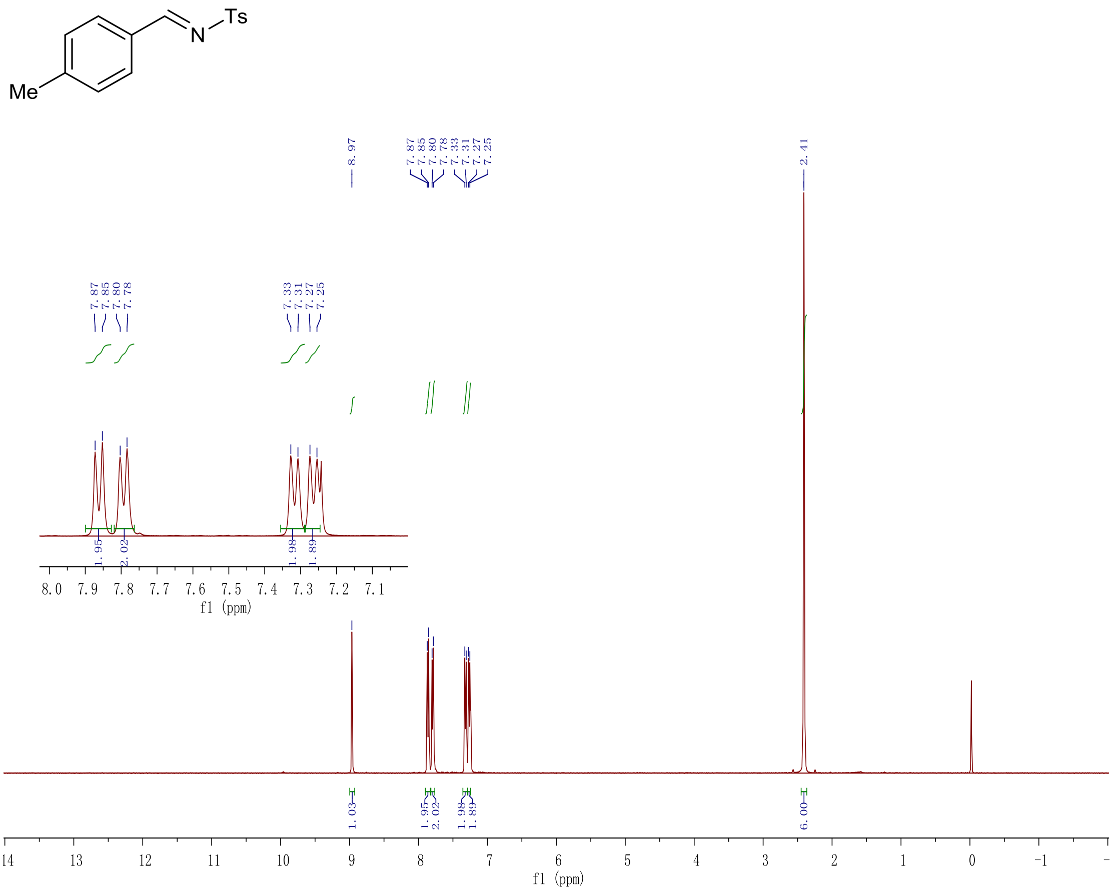

**Supplementary Figure 93 $^{1}$H NMR of (*E*)-4-Methyl-*N*-(2,4,6-trimethyl-benzylidene)benzenesulfonamide 6d**

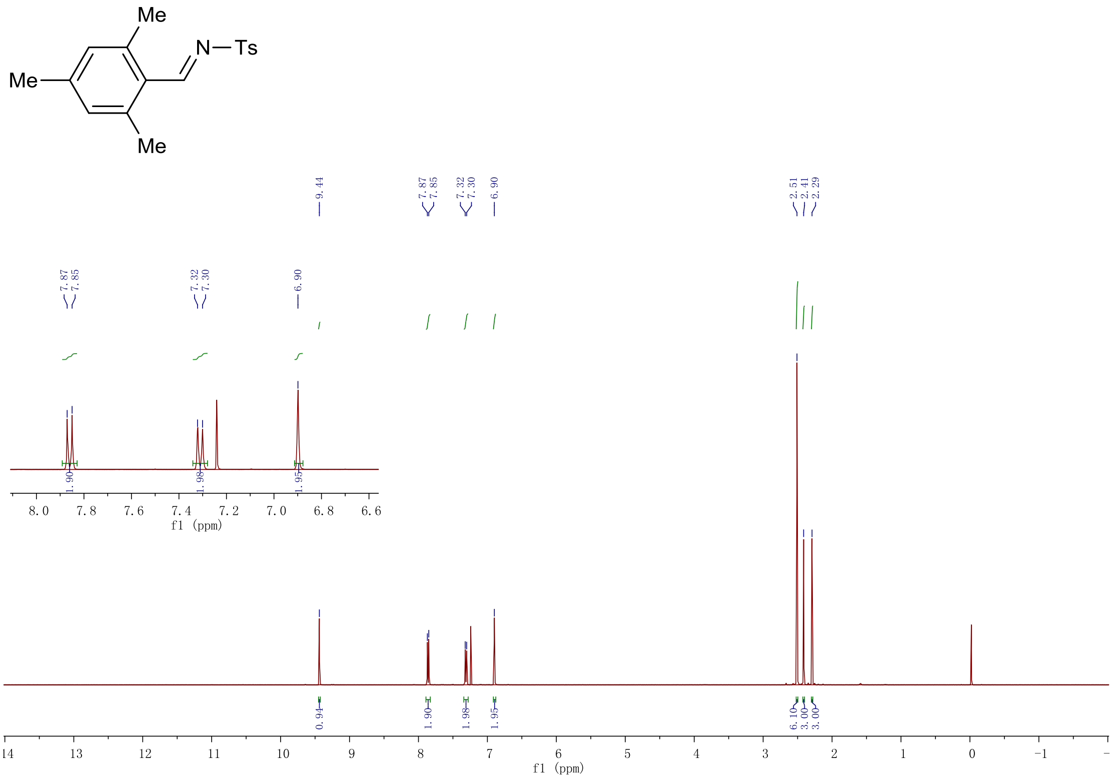


**Supplementary Figure 94 $^{13}$C NMR of (*E*)-4-Methyl-*N*-(2,4,6-trimethyl-benzylidene)benzenesulfonamide 6d**

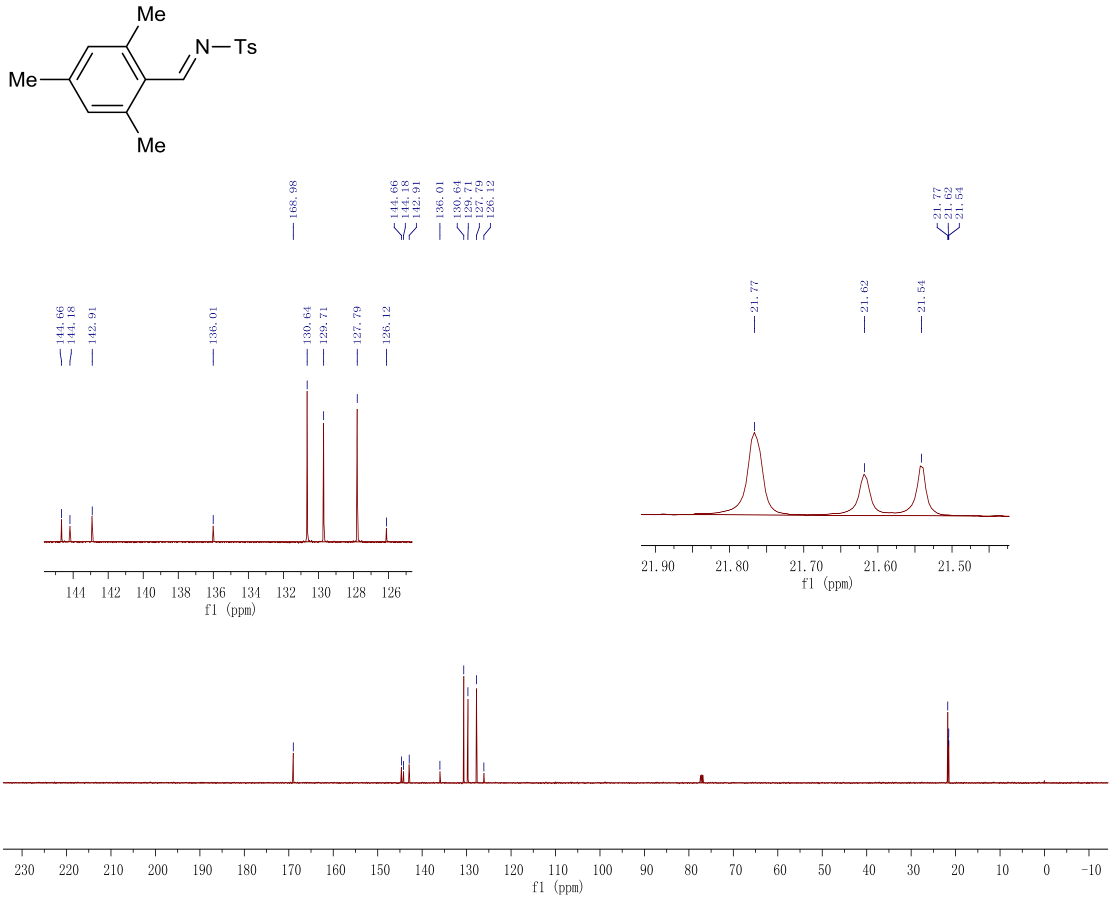

**Supplementary Figure 95 $^1$H NMR of (*E*)-*N*-(4-Methoxybenzylidene)-4-methyl-benzenesulfonamide 6e**

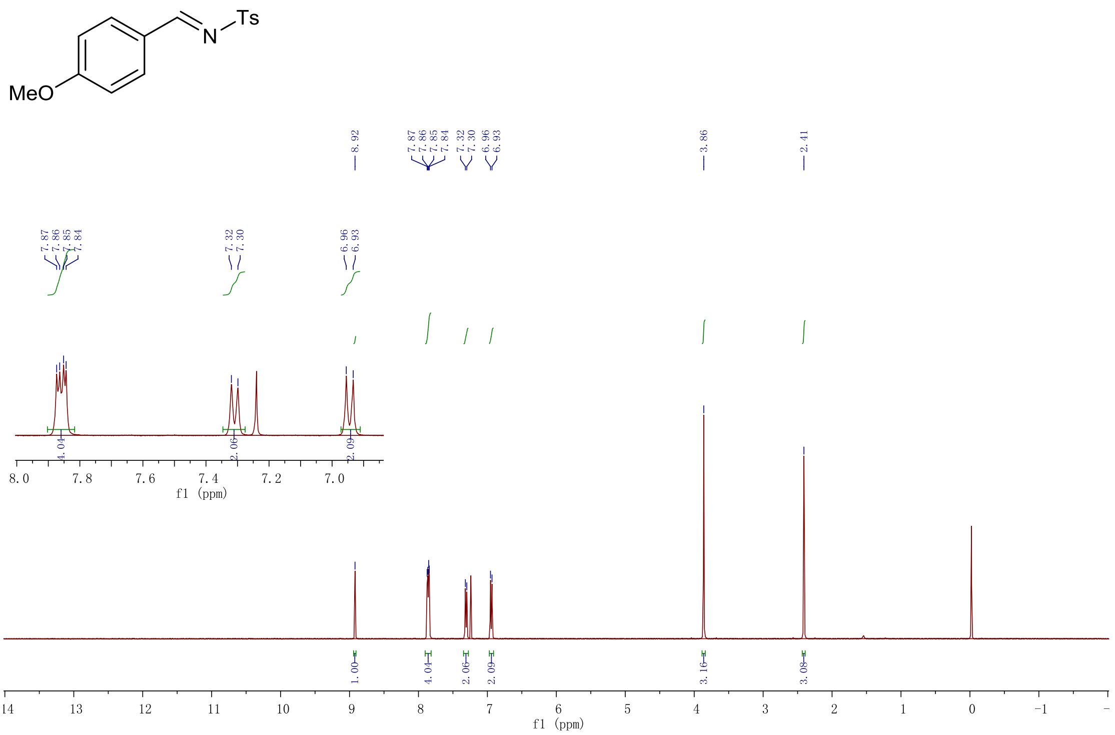


**Supplementary Figure 96 $^1$H NMR of (*E*)-*N*-(2-Methoxybenzylidene)-4-methyl-benzenesulfonamide 6f**

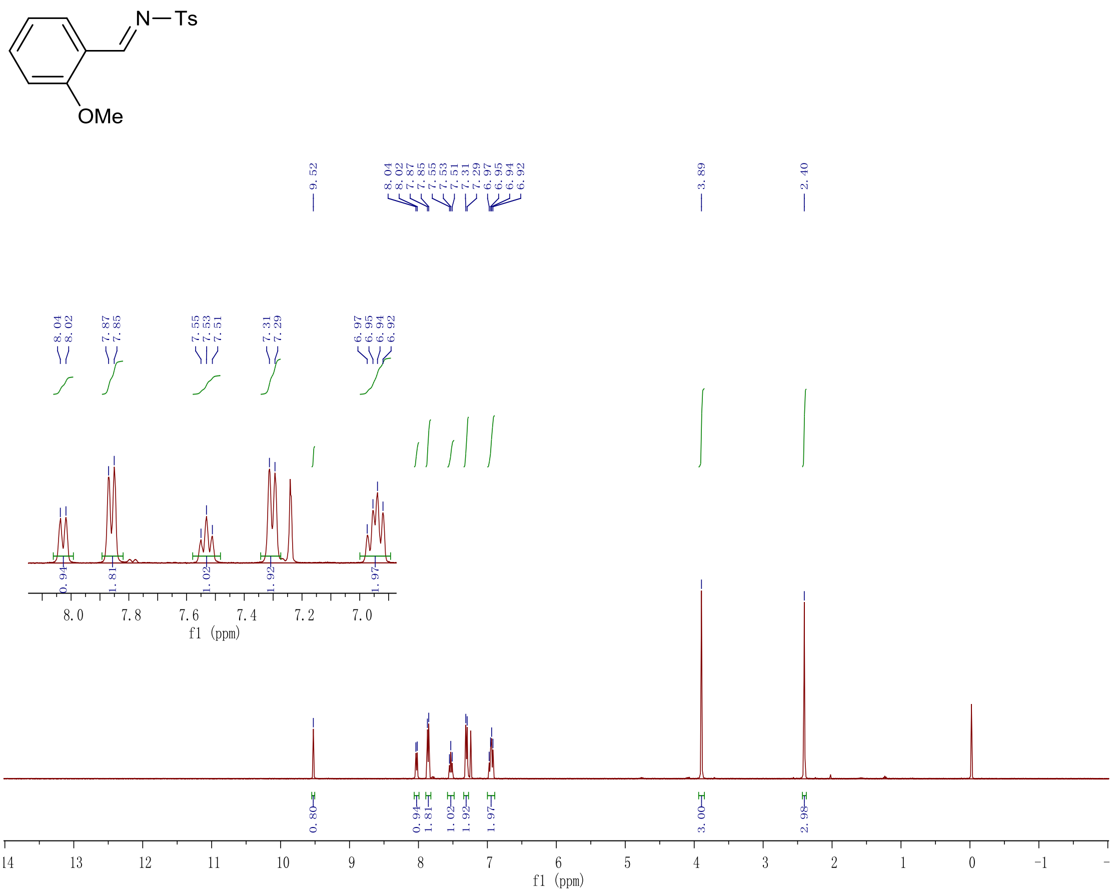

**Supplementary Figure 97 $^{1}$H NMR of (*E*)-4-Methyl-*N*-(4-(trifluoromethoxy)-benzylidene)benzenesulfonamide 6g**

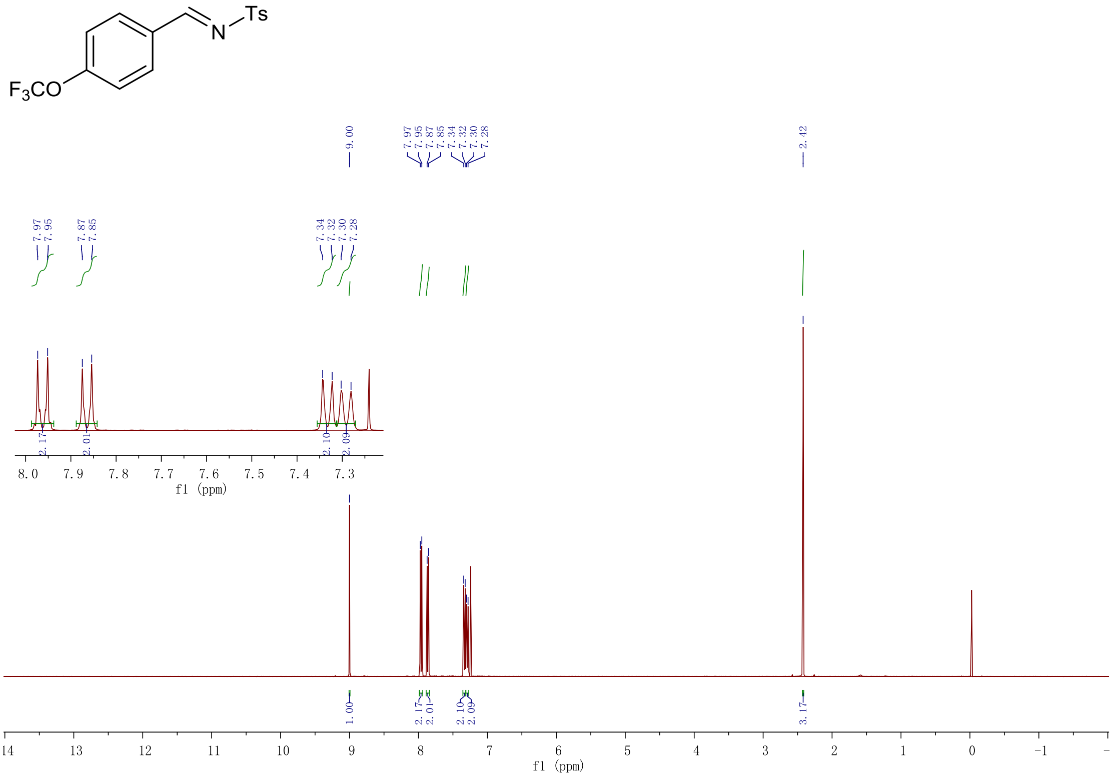

**Supplementary Figure 98 $^{19}$F NMR of (*E*)-4-Methyl-*N*-(4-(trifluoromethoxy)-benzylidene)benzenesulfonamide 6g**

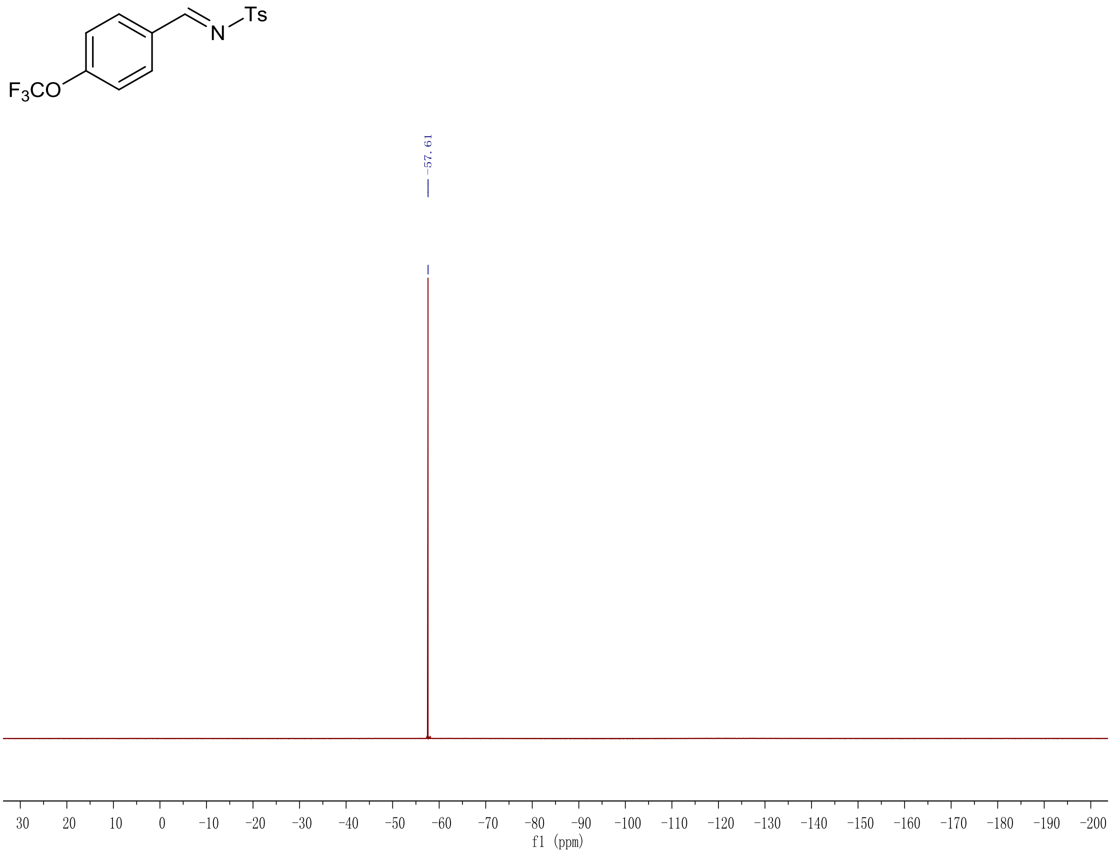

**Supplementary Figure 99.** $^{13}$**C NMR of (*E*)-4-Methyl-*N*-(4-(trifluoromethoxy)-benzylidene)benzenesulfonamide 6g**

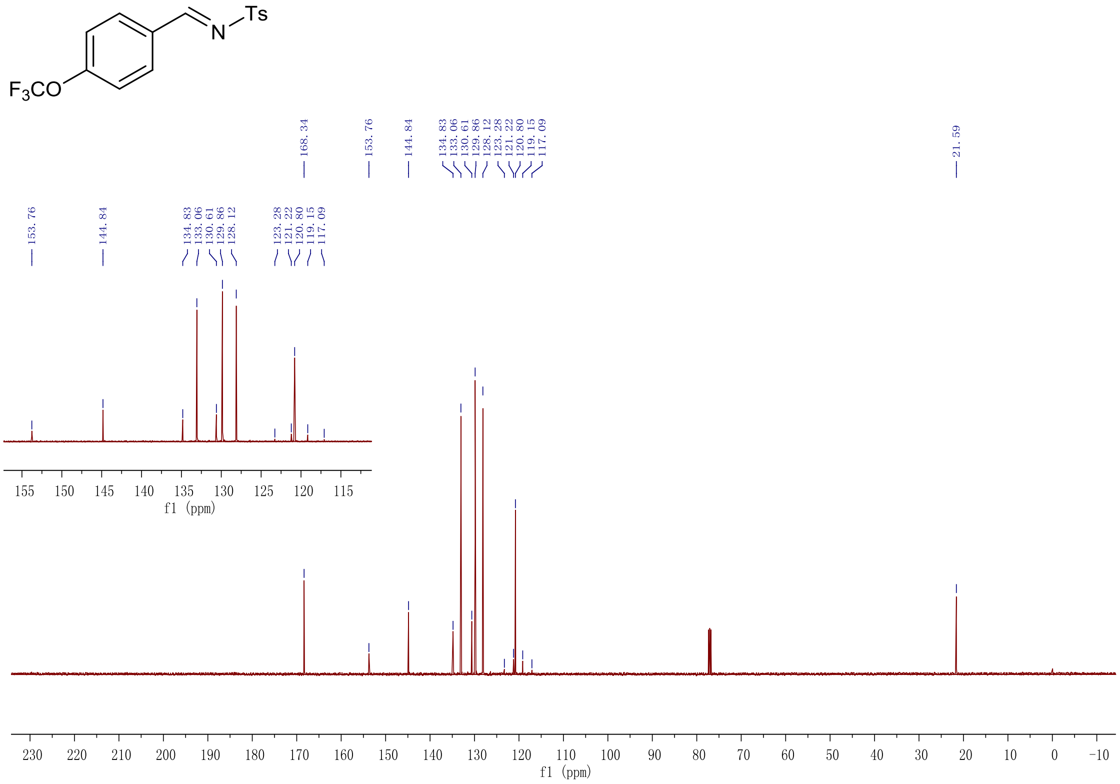


**Supplementary Figure 100.** $^{1}$**H NMR of (*E*)-*N*-(4-(Benzyloxy)benzylidene)-4-methylbenzenesulfonamide 6h**

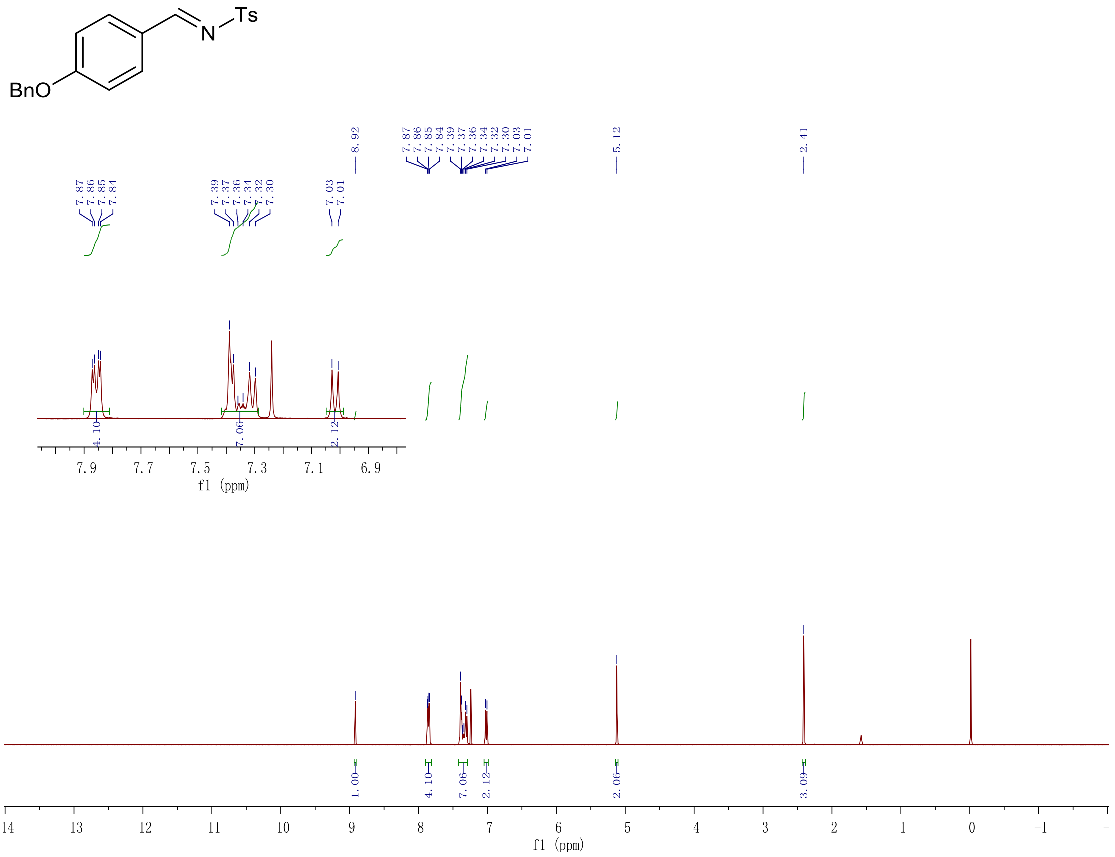

**Supplementary Figure 101. $^{13}$C NMR of (*E*)-*N*-(4-(Benzyloxy)benzylidene)-4-methylbenzenesulfonamide 6h**

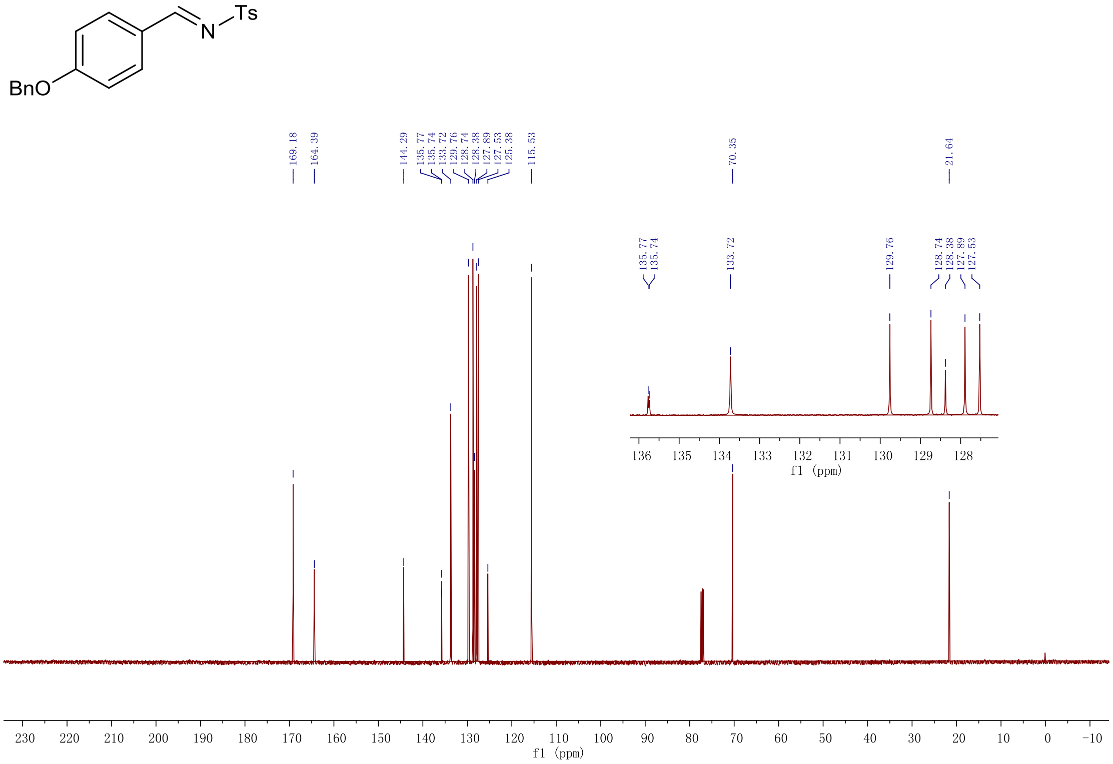


**Supplementary Figure 102. $^{1}$H NMR of (E)-N-(4-Fluorobenzylidene)-4-methyl-benzenesulfonamide 6i**

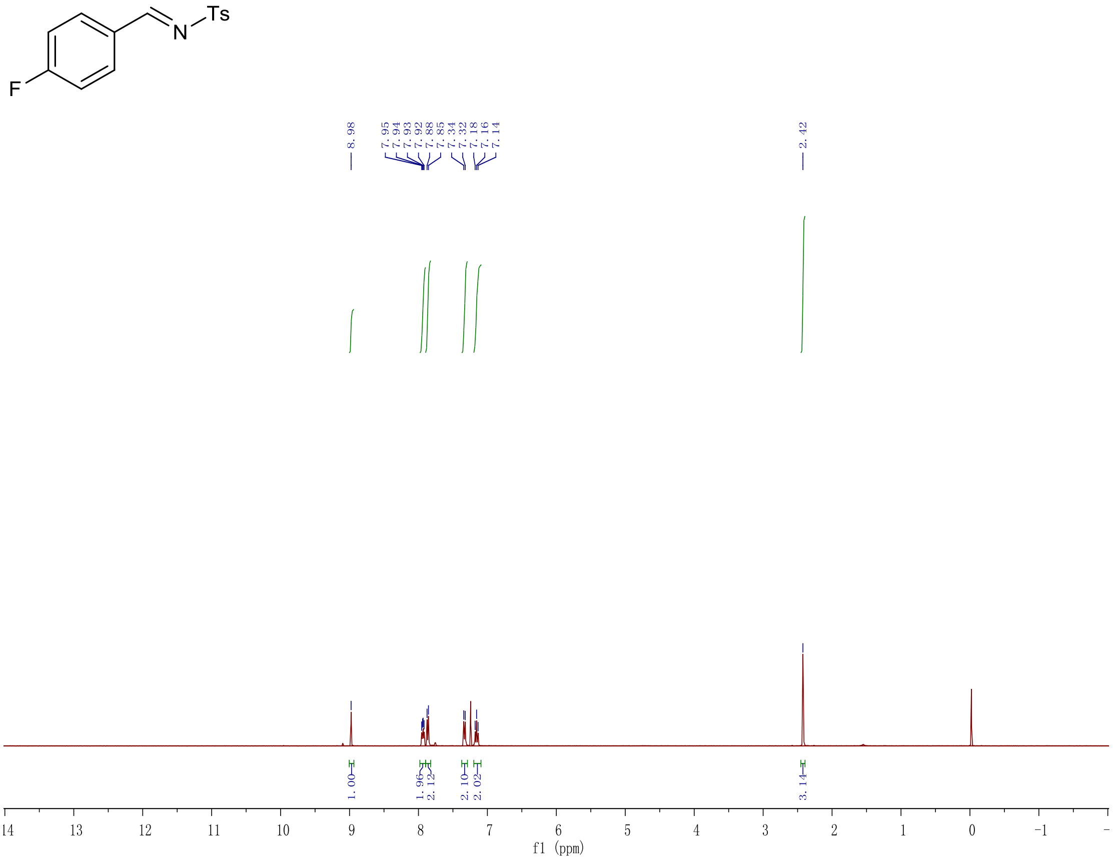

**Supplementary Figure 103. $^{19}$F NMR of (E)-N-(4-Fluorobenzylidene)-4-methyl -benzenesulfonamide 6i**

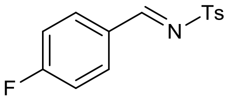


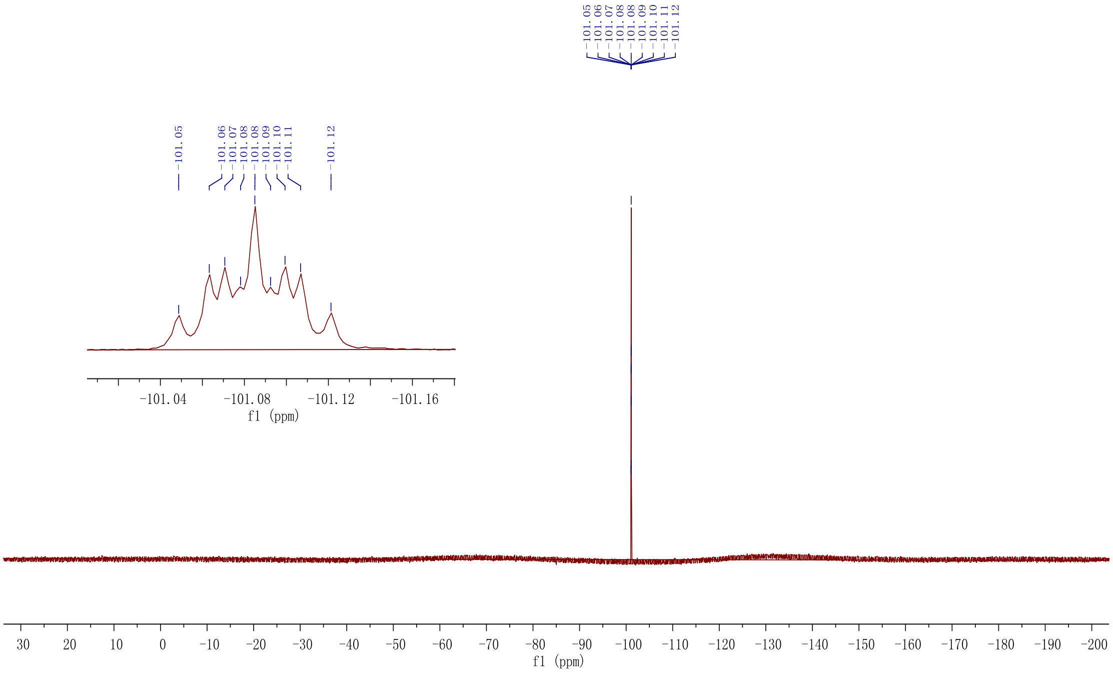


**Supplementary Figure 104. $^{1}$H NMR of (E)-4-Methyl-N-(naphthalen-2-ylmethylene)benzenesulfonamide 6j**

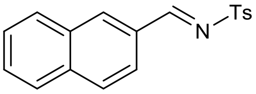


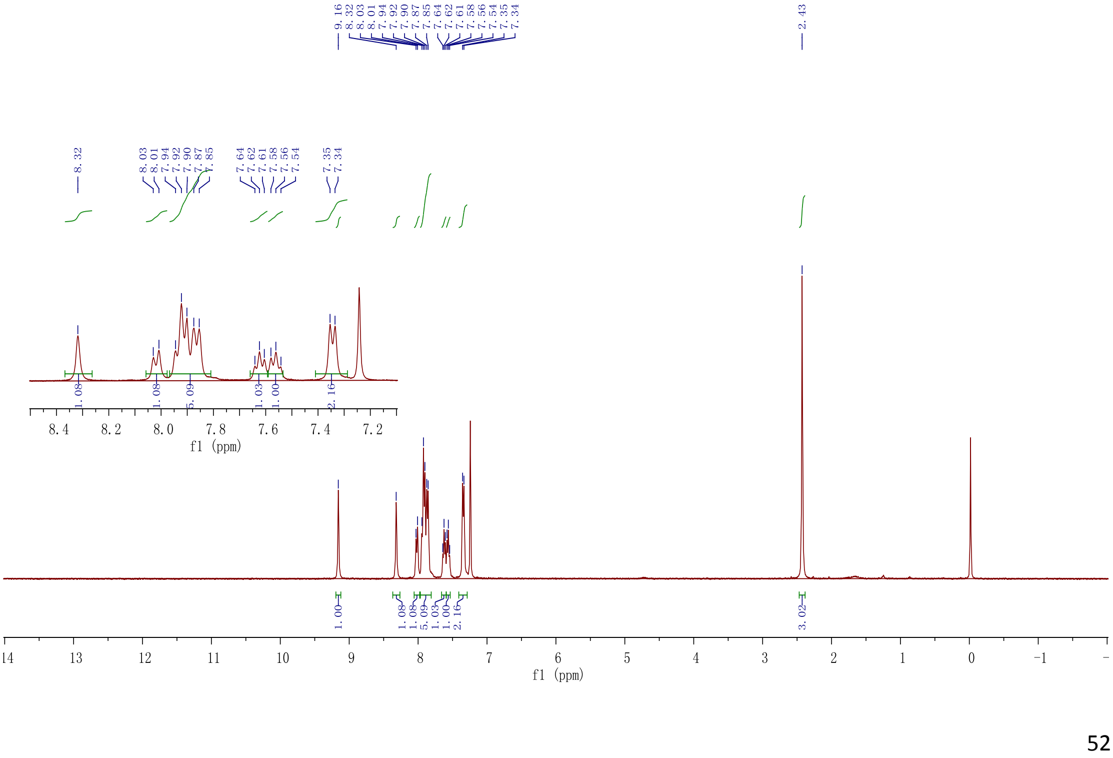

**Supplementary Figure 105. $^{1}$H NMR of *N*-Benzhydryl-4-methylbenzenesulfon -amide 7a**

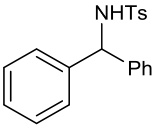


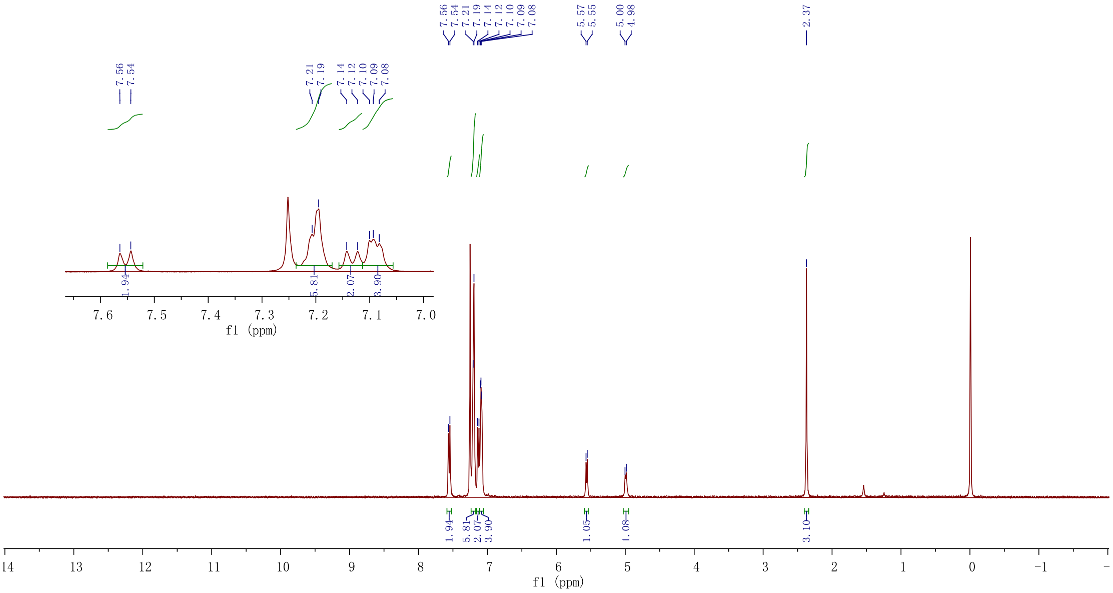

**Supplementary Figure 106. $^{1}$H NMR of *N*-([1,1'-Biphenyl]-4-yl(phenyl)methyl) -methyl-benzenesulfonamide 7b**

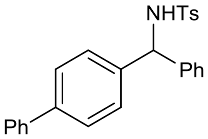


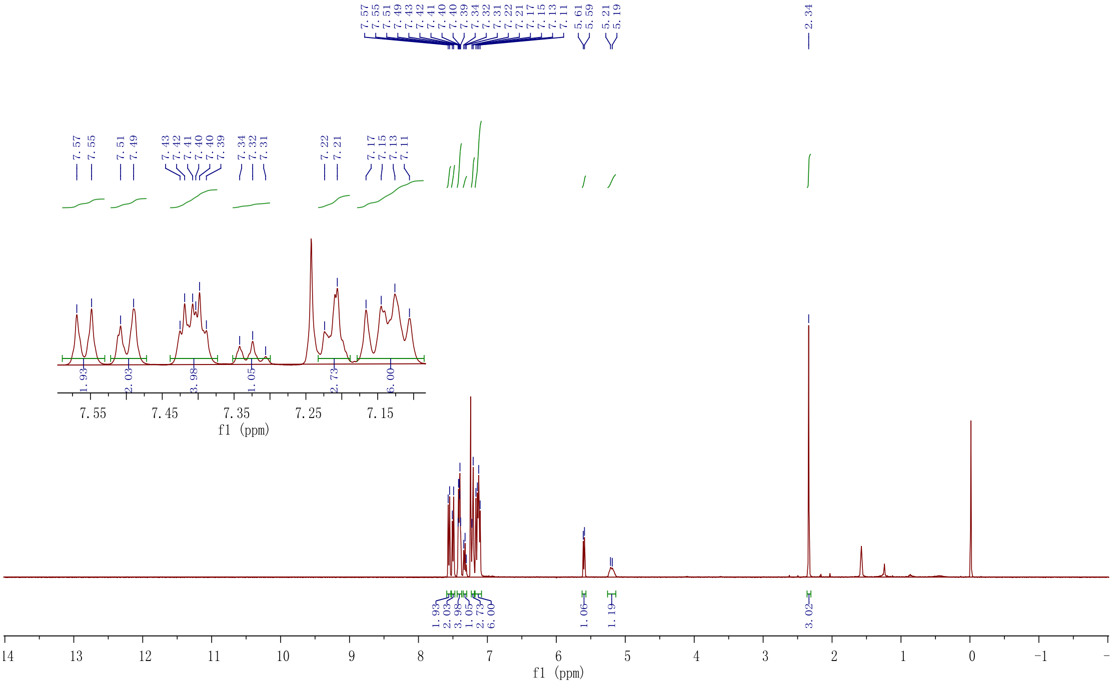

**Supplementary Figure 107. $^{1}$H NMR of 4-Methyl-*N*-(phenyl(p-tolyl)methyl) -benzenesulfonamide 7c**

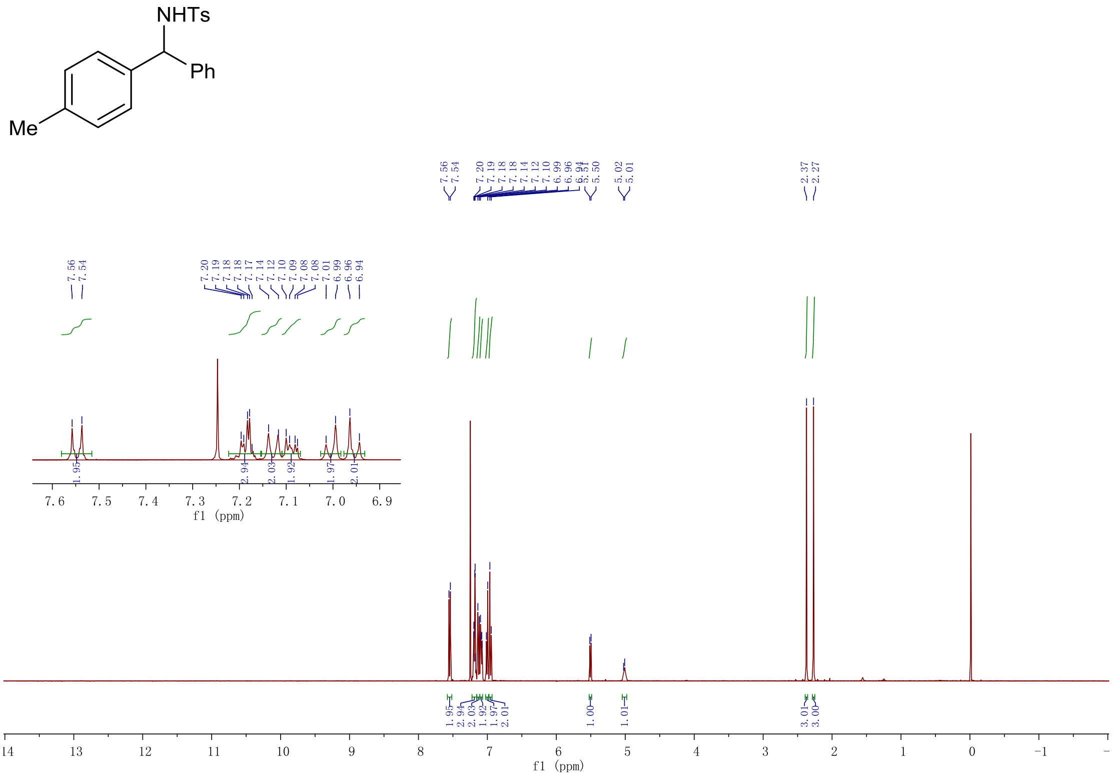


**Supplementary Figure 108. $^{1}$H NMR of *N*-(Mesityl(phenyl)methyl)-4-methyl -benzenesulfonamide 7d**

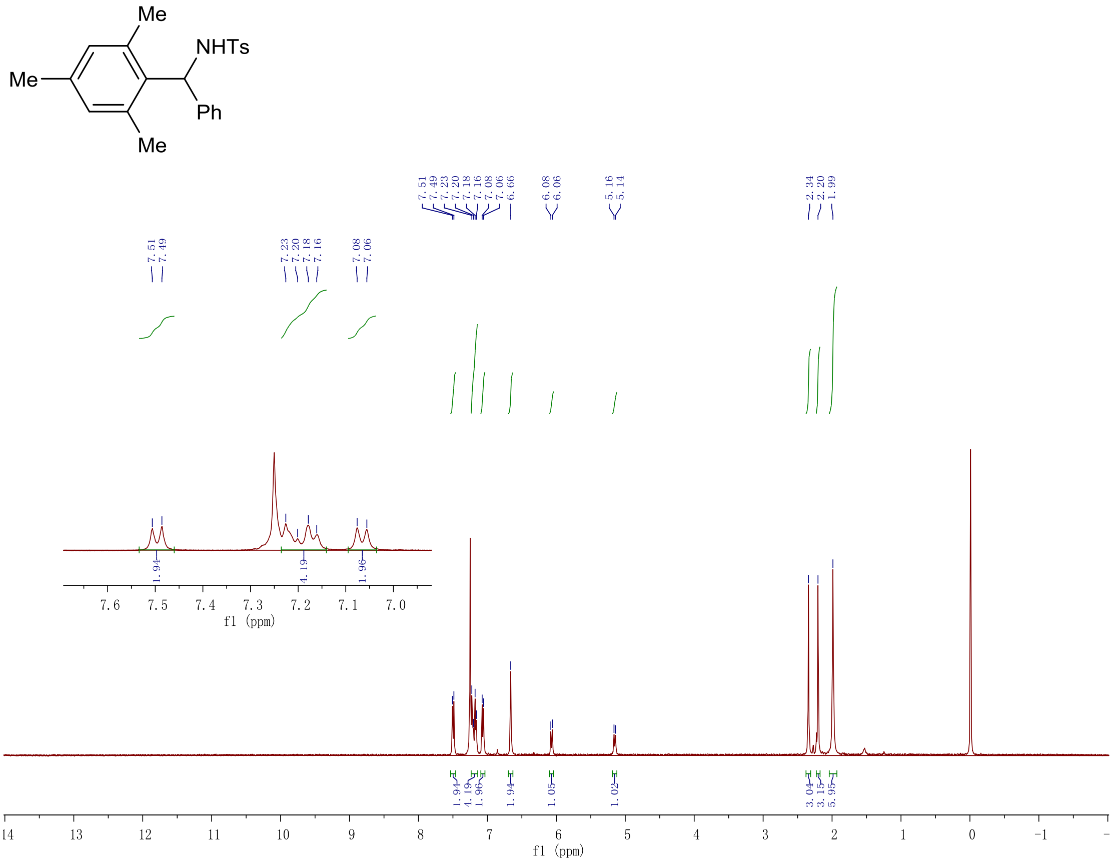

**Supplementary Figure 109. $^{13}$C NMR of *N*-(Mesityl(phenyl)methyl)-4-methyl -benzenesulfonamide 7d**

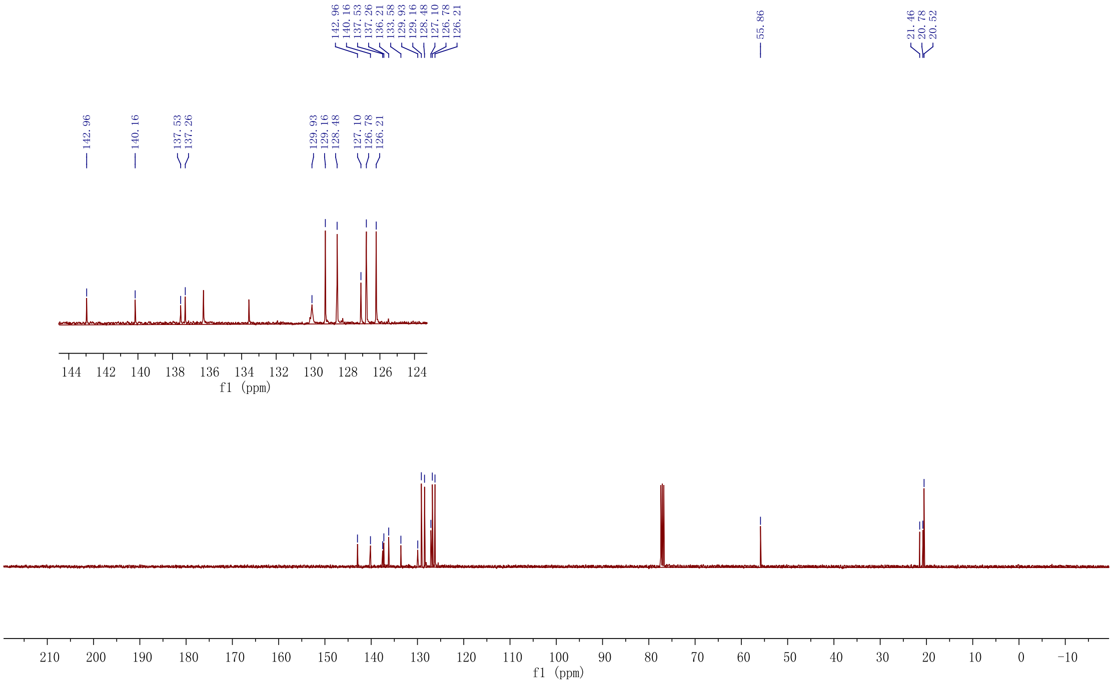


**Supplementary Figure 110. $^{1}$H NMR of N-((4-Methoxyphenyl) (phenyl)methyl) -4-methylbenzenesulfonamide 7e**

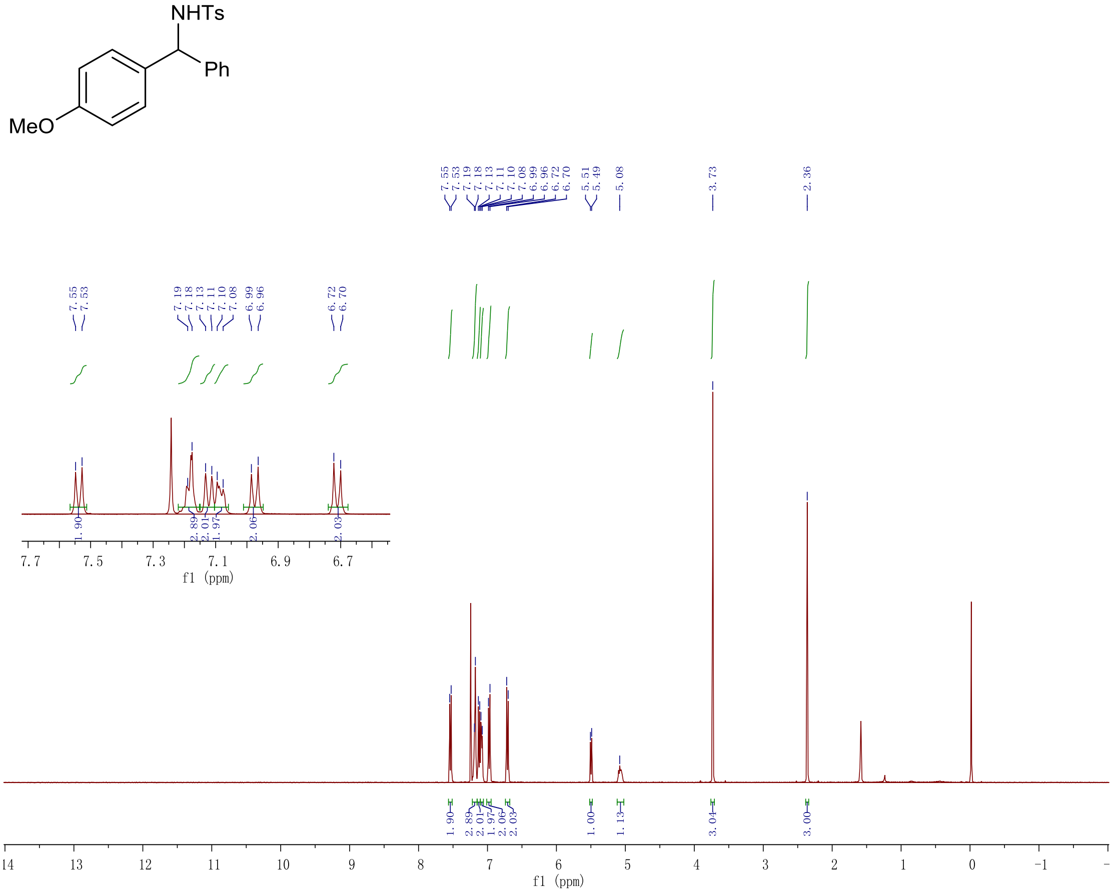

**Supplementary Figure 111. $^{1}$H NMR of *N*-((2-Methoxyphenyl)(phenyl)methyl)-4-methylbenzenesulfonamide 7f**

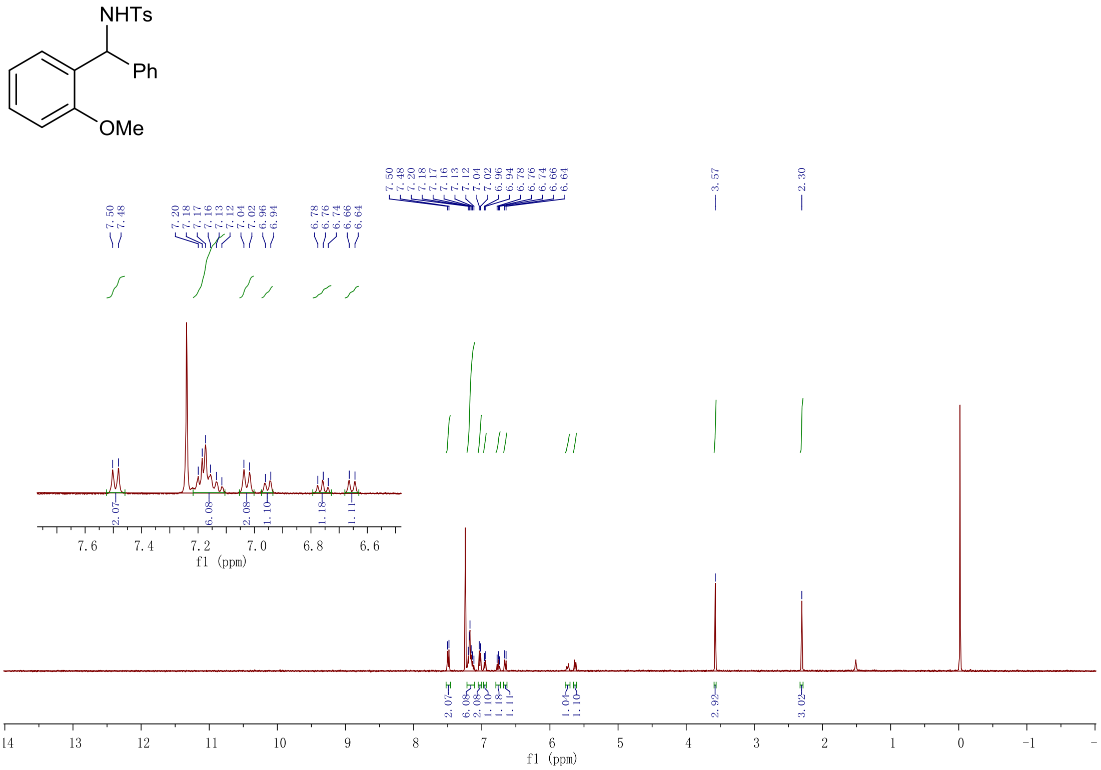


**Supplementary Figure 112. $^{1}$H NMR of 4-Methyl-*N*-(phenyl(4-(trifluoromethoxy)-phenyl)methyl)benzenesulfonamide 7g**

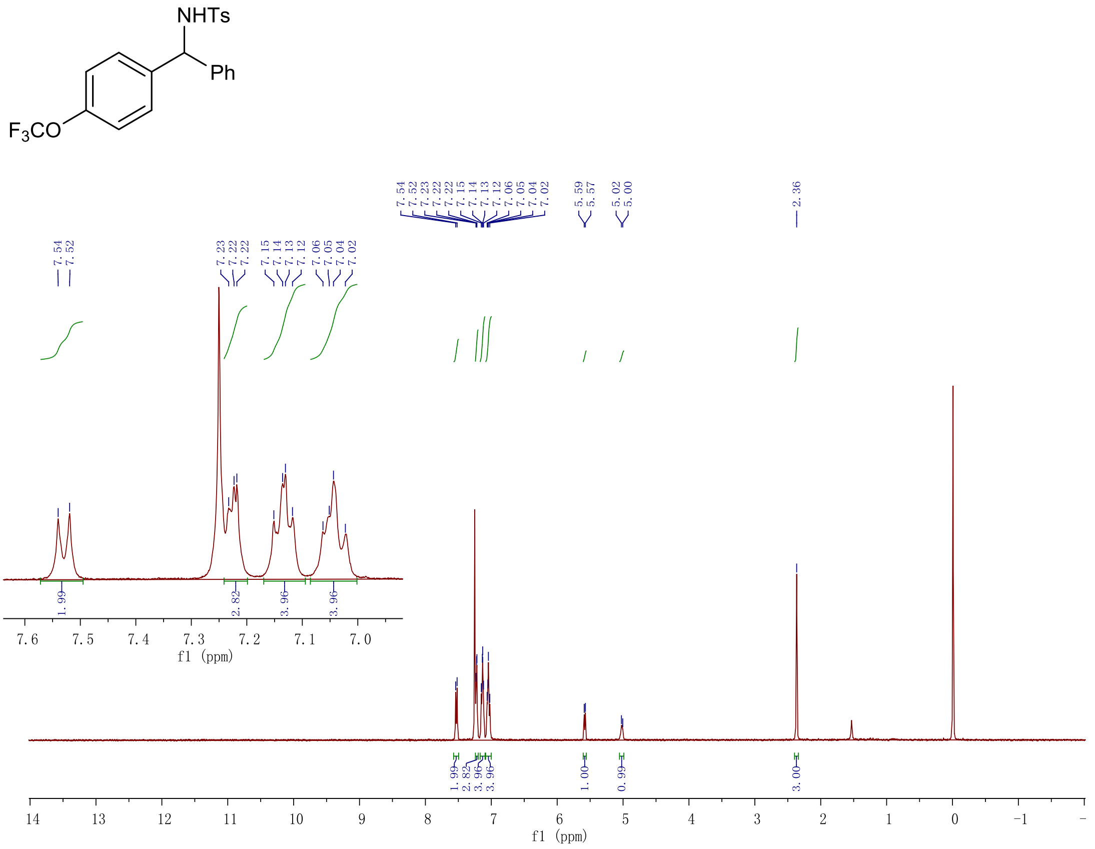

**Supplementary Figure 113. $^{19}$F NMR of 4-Methyl-*N*-(phenyl(4-(trifluoromethoxy) -phenyl)methyl)benzenesulfonamide 7g**

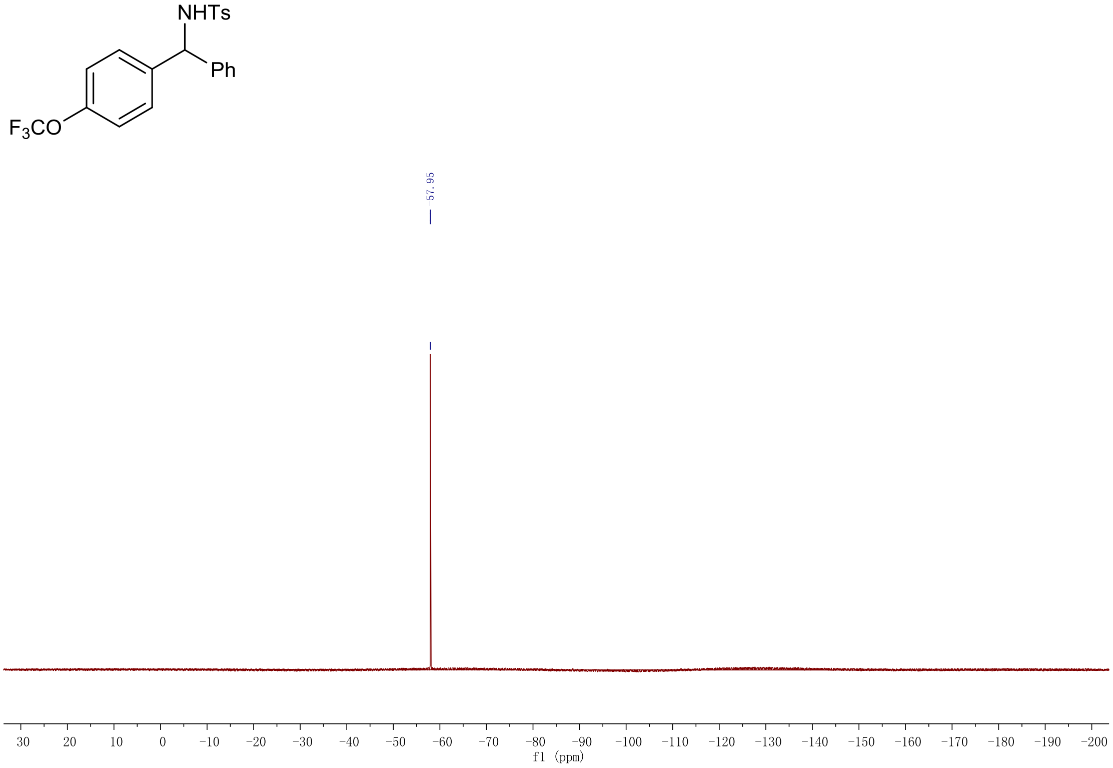


**Supplementary Figure 114. $^{13}$C NMR of 4-Methyl-*N*-(phenyl(4-(trifluoromethoxy) -phenyl)methyl)benzenesulfonamide 7g**

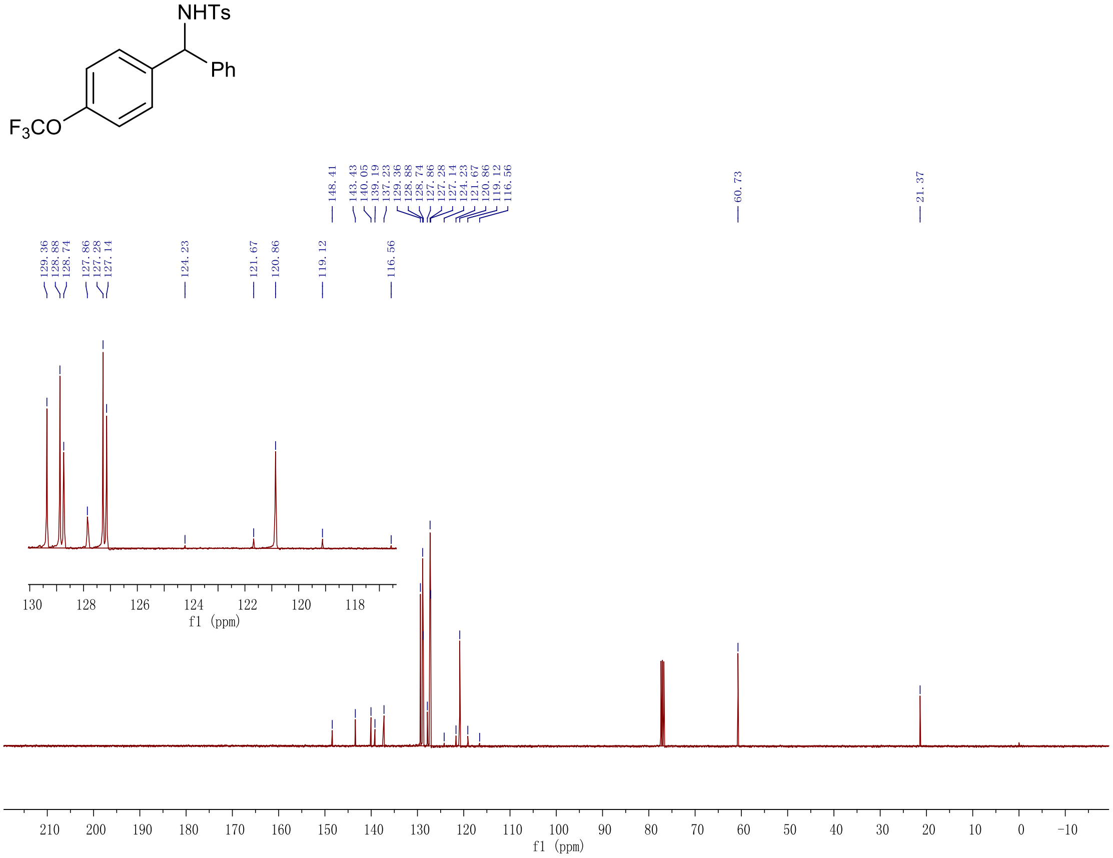

**Supplementary Figure 115. $^{1}$H NMR of *N*-((4-(Benzyloxy)phenyl)(phenyl)methyl)-4-methylbenzenesulfonamide 7h**

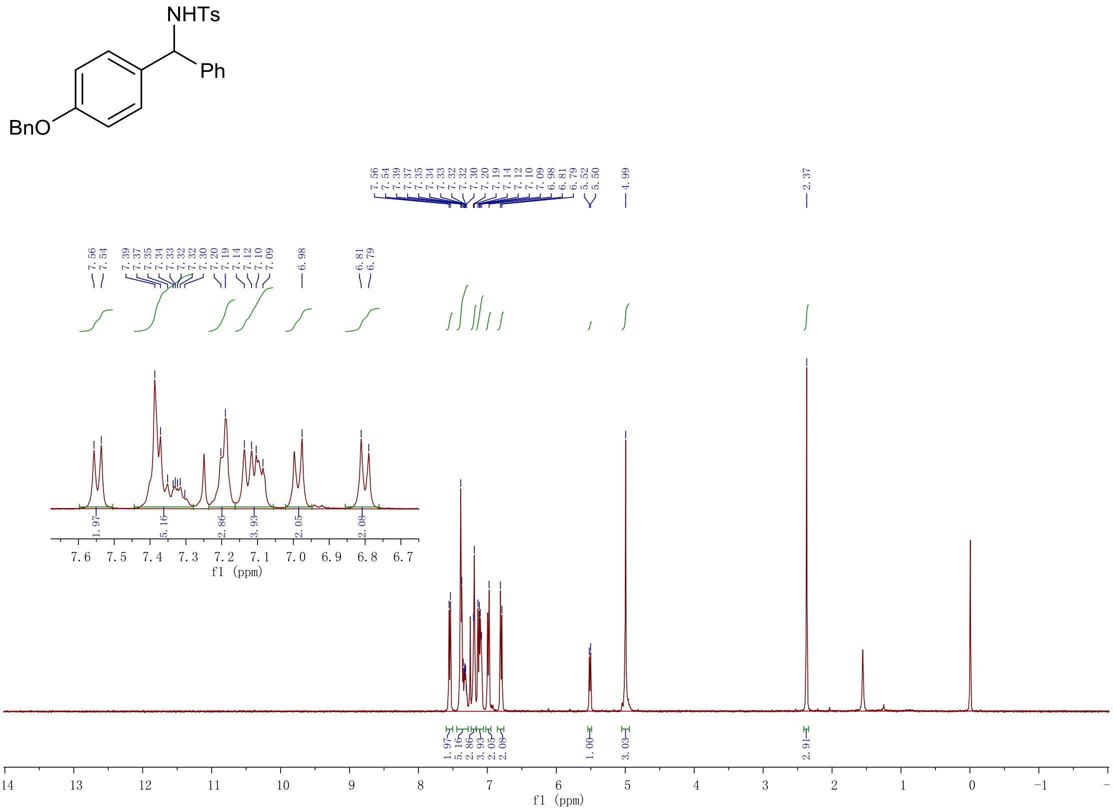

**Supplementary Figure 116. $^{13}$C NMR of *N*-((4-(Benzyloxy)phenyl)(phenyl)methyl)-4-methylbenzenesulfonamid 7h**

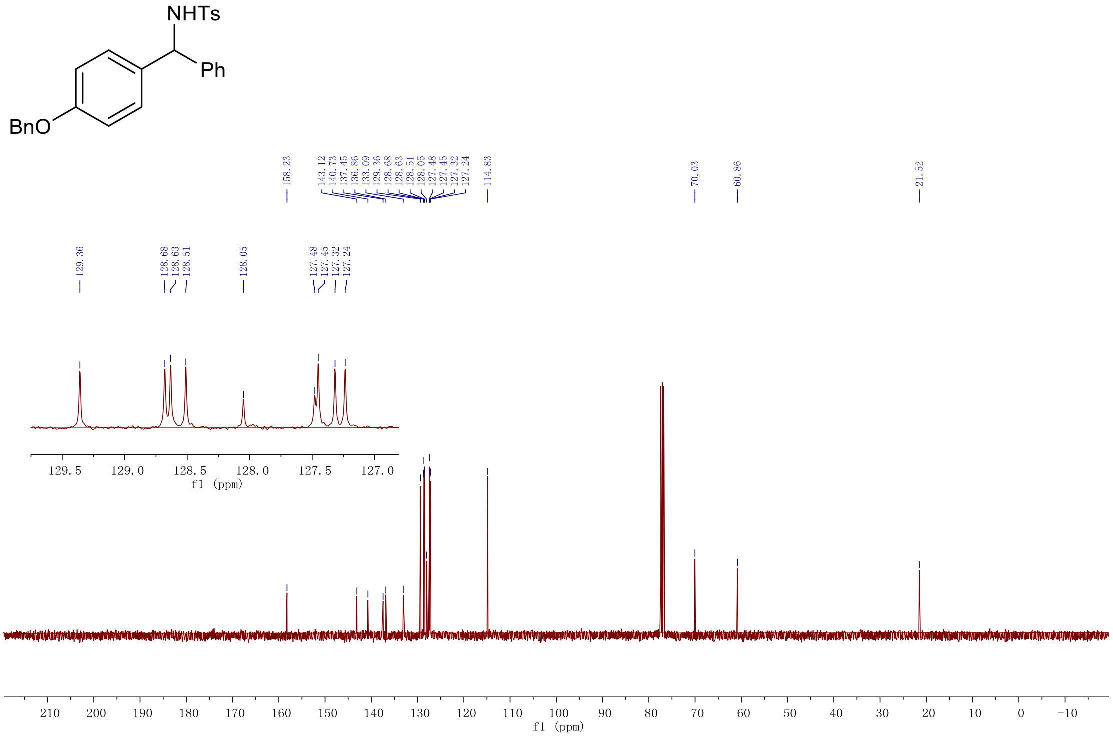

**Supplementary Figure 117.** $^{1}$**H NMR of *N*-((4-Fluorophenyl)(phenyl)methyl)-4-methylbenzenesulfonamide 7i**

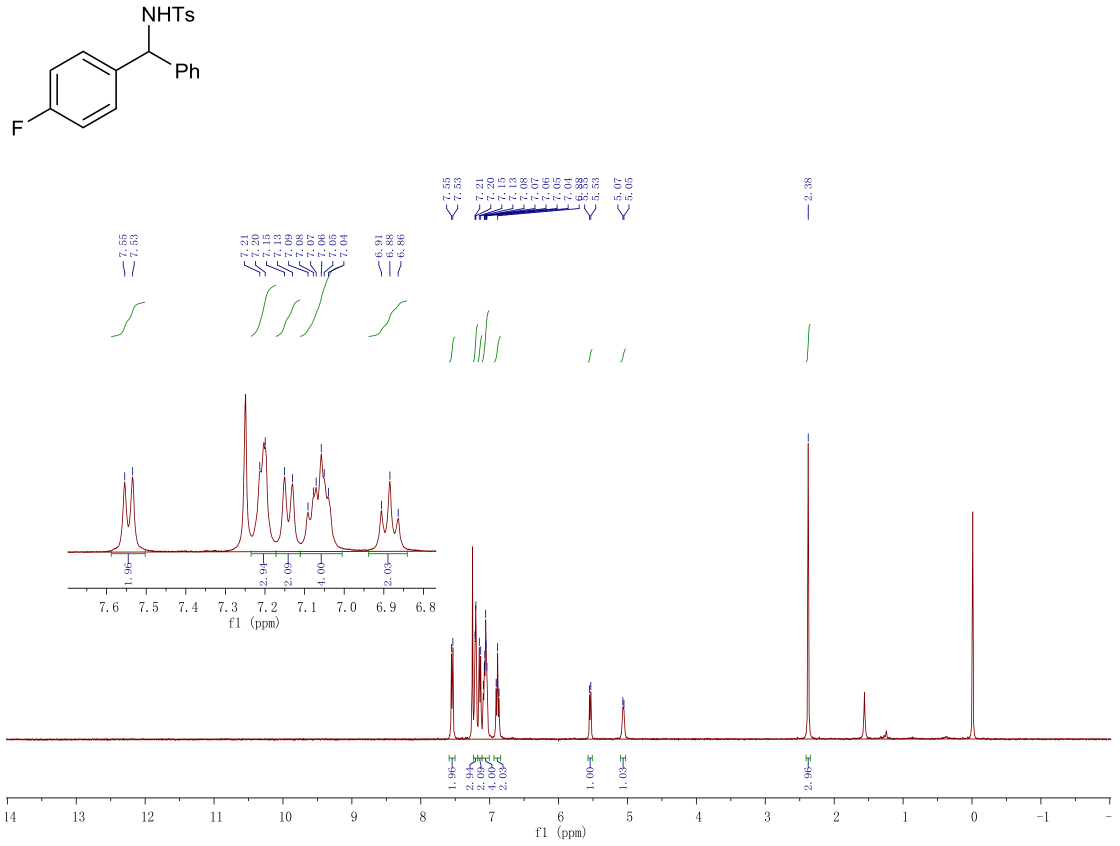


**Supplementary Figure 118.** $^{19}$**F NMR of *N*-((4-Fluorophenyl)(phenyl)methyl)-4-methylbenzenesulfonamide 7i**

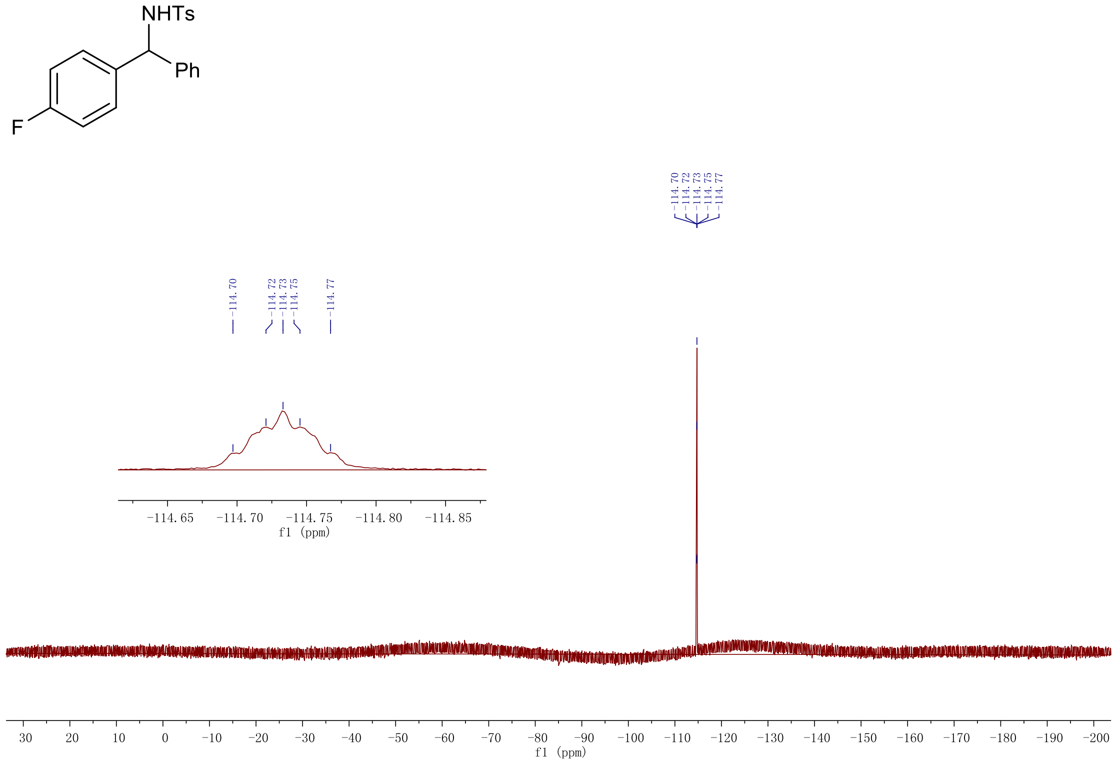

**Supplementary Figure 119. $^{1}$H NMR of 4-Methyl-*N*-(naphthalen-2-yl(phenyl)-methyl)benzenesulfonamide 7j**

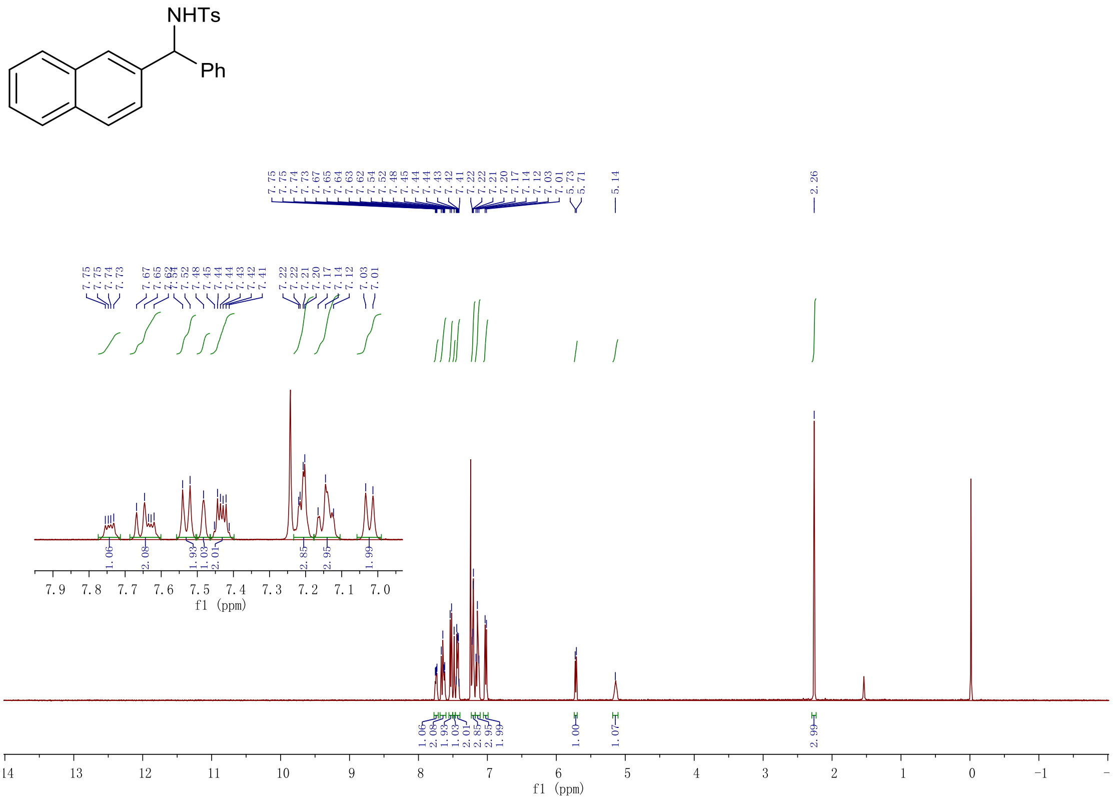

**Supplementary Figure 120. $^{1}$H NMR of [1,1'-Biphenyl]-4-yl(4-methoxyphenyl)-methanol 3ab**

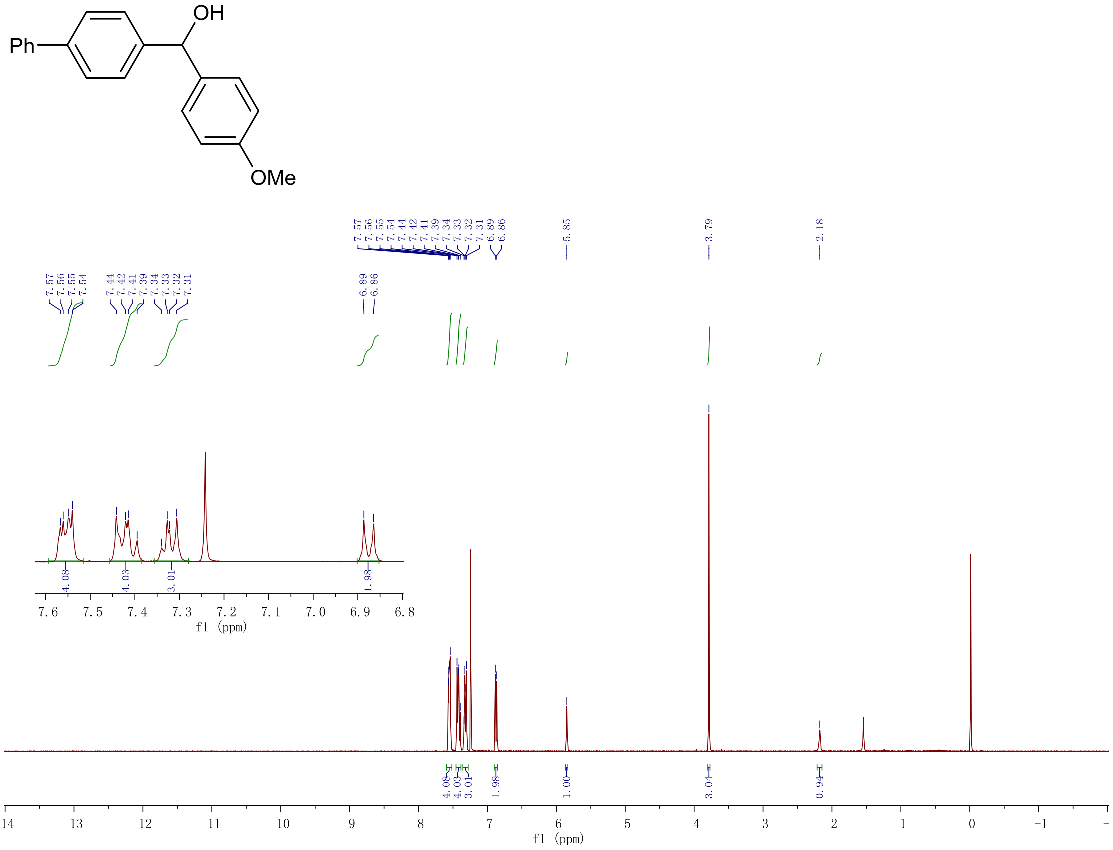

## Supplementary Figure 121. $^{1}$H NMR of [1,1'-Biphenyl]-4-yl(p-tolyl)methanol 3ac

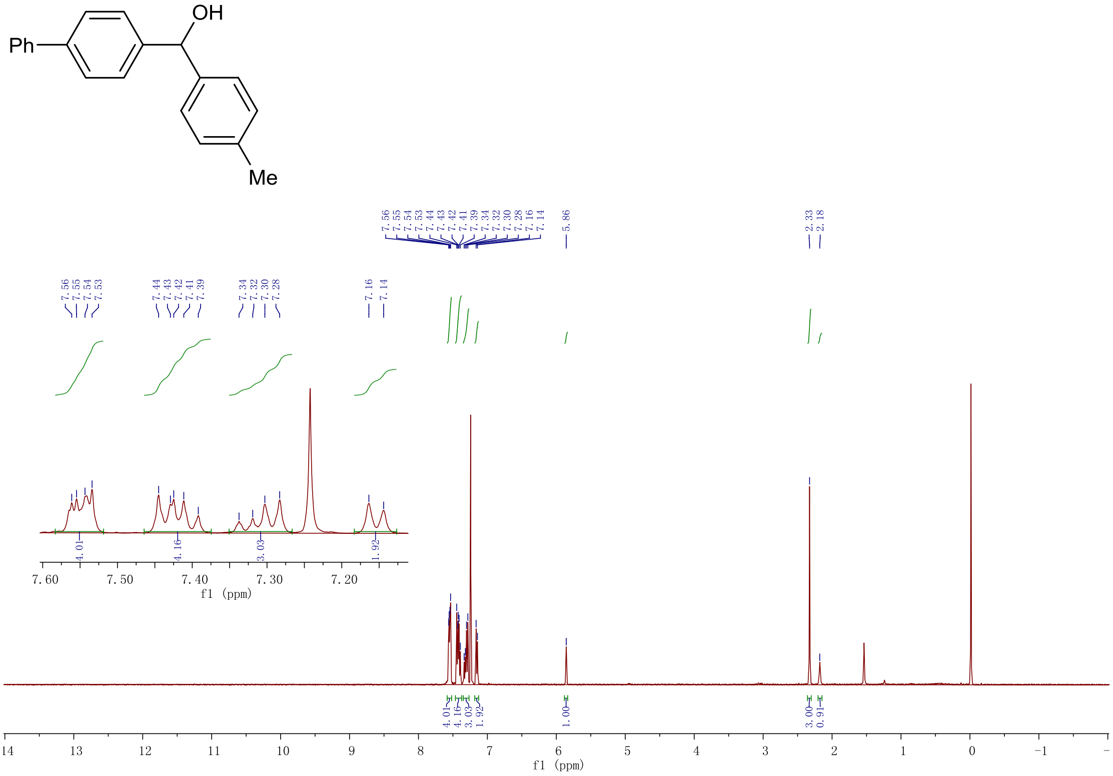


## Supplementary Figure 122. $^{1}$H NMR of di([1,1'-Biphenyl]-4-yl)methanol 3ad

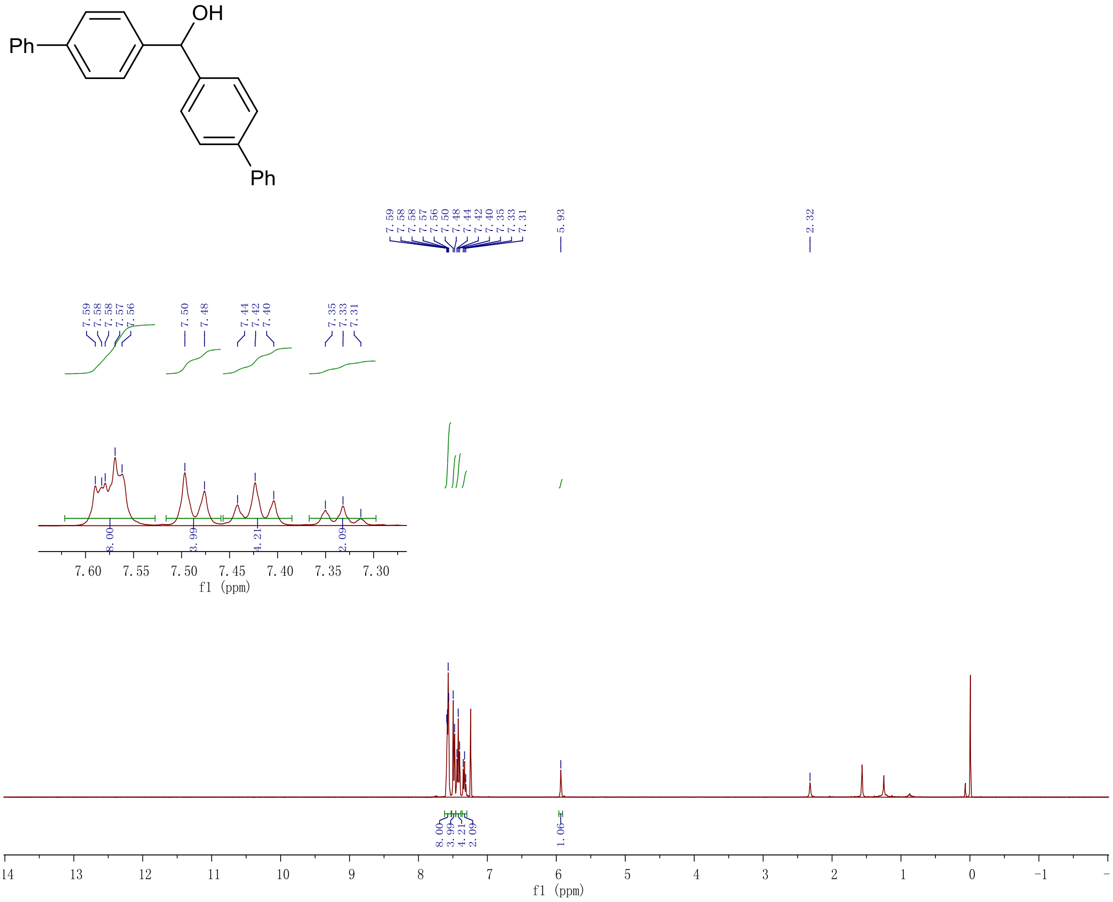

**Supplementary Figure 123. ¹H NMR of 4-([1,1'-Biphenyl]-4-yl(hydroxy)methyl) -benzonitrile 3ae**

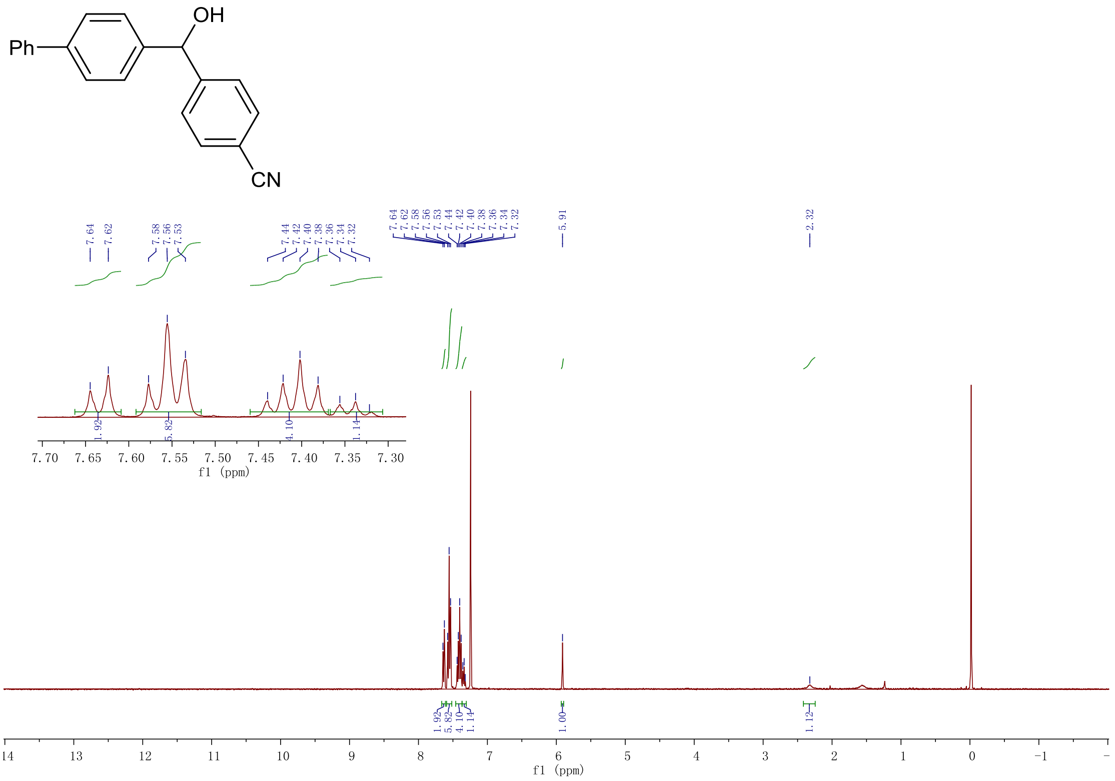


**Supplementary Figure 124. ¹H NMR of 3-([1,1'-Biphenyl]-4-yl(hydroxy)methyl) -benzonitrile 3af**

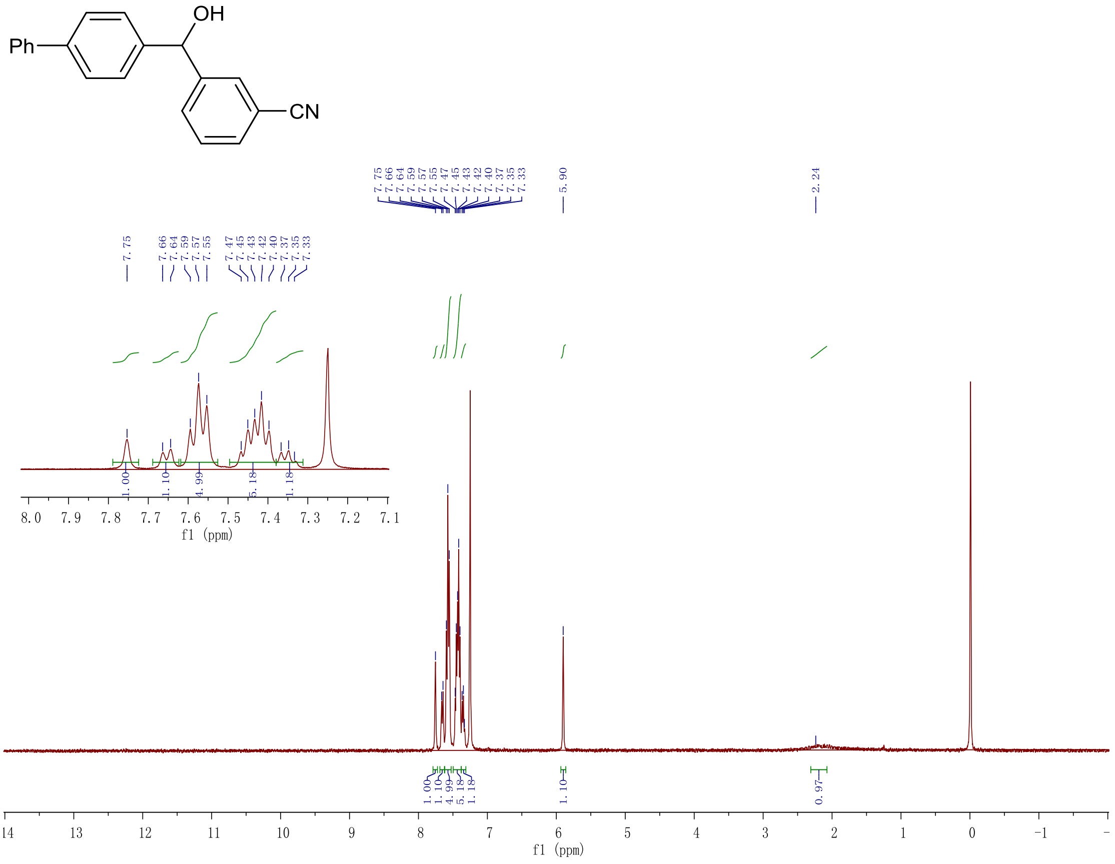

**Supplementary Figure 125. $^{13}$C NMR of 3-([1,1'-Biphenyl]-4-yl(hydroxy)methyl)-benzonitrile 3af**

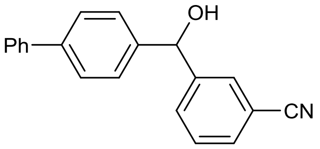

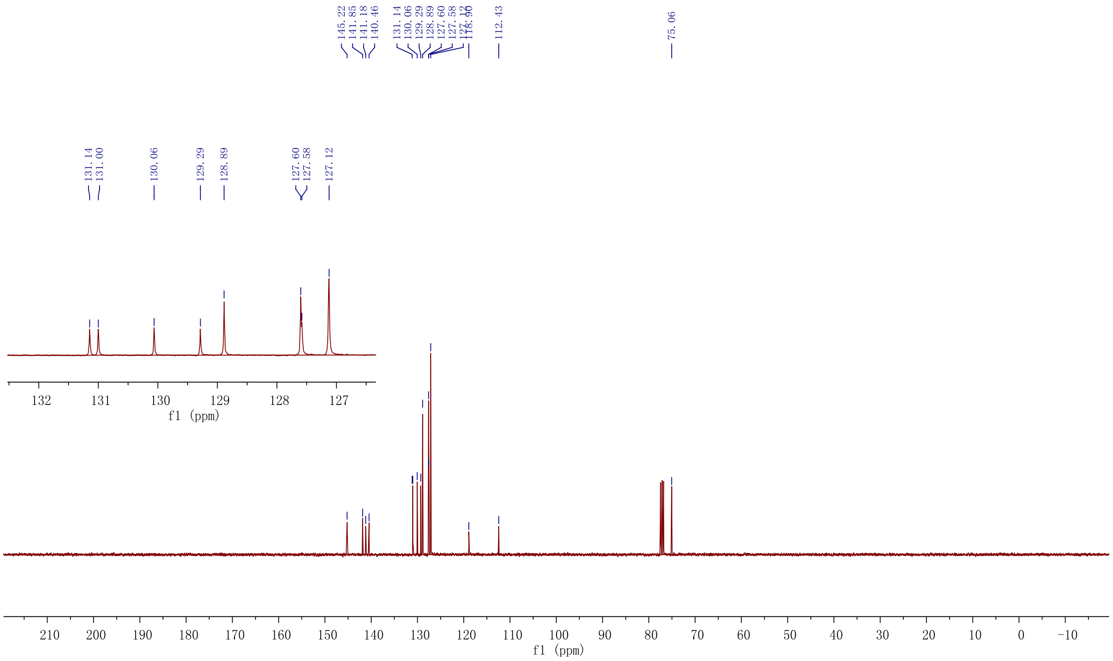

**Supplementary Figure 126. $^{1}$H NMR of [1,1'-Biphenyl]-4-yl(4-(trifluoromethyl)-phenyl)methanol 3ag**

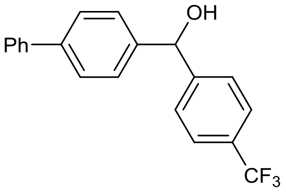

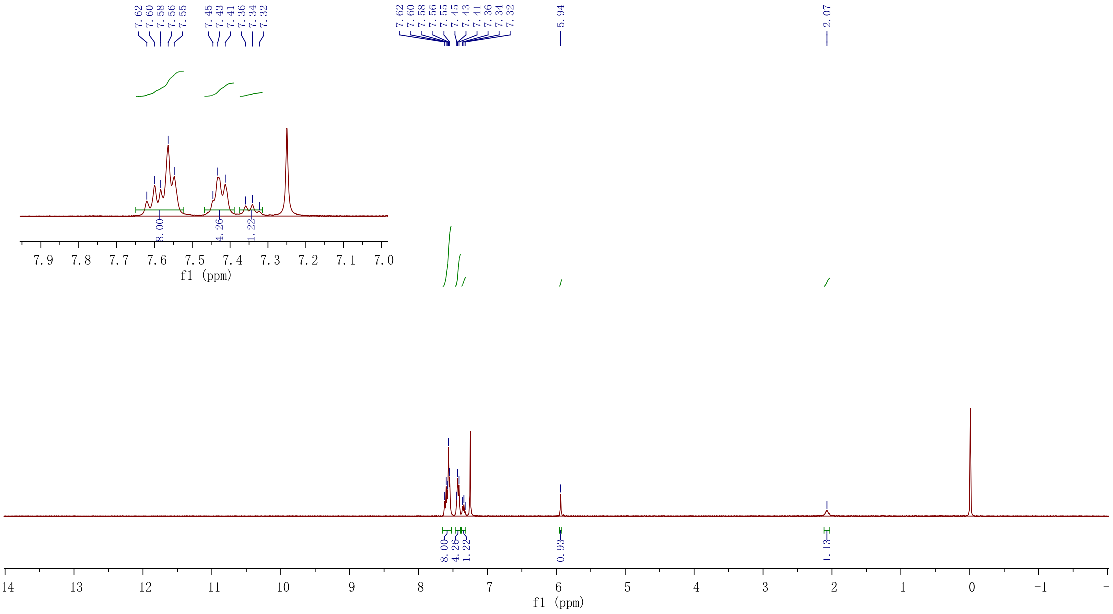

**Supplementary Figure 127. $^{19}$F NMR of [1,1'-Biphenyl]-4-yl(4-(trifluoromethyl) -phenyl)methanol 3ag**

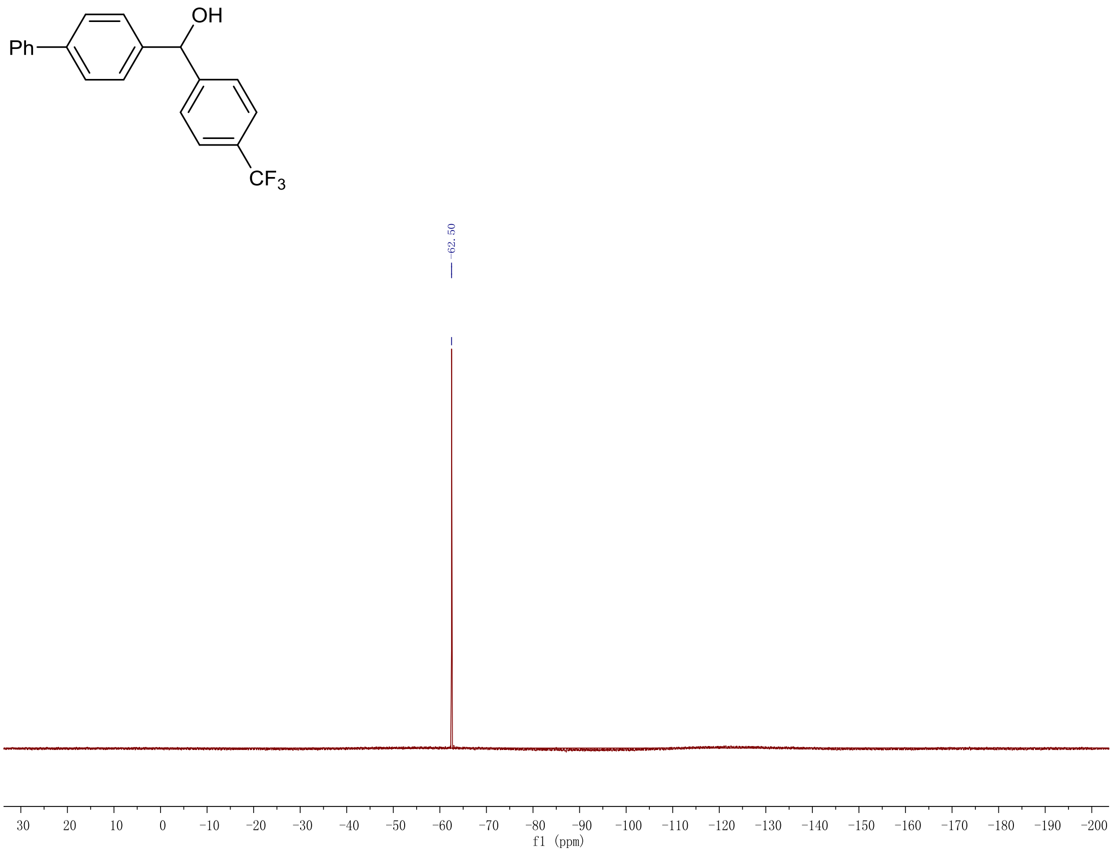


**Supplementary Figure 128. $^{13}$C NMR of [1,1'-Biphenyl]-4-yl(4- (trifluoromethyl) -phenyl)methanol 3ag**

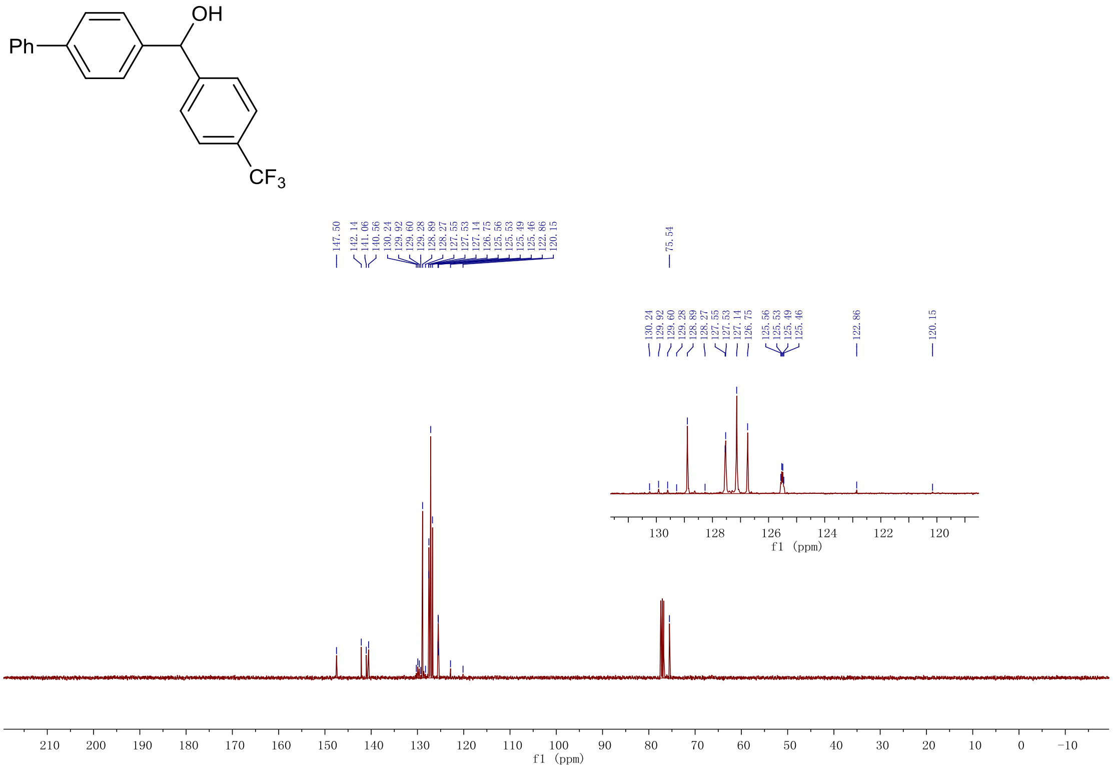

**Supplementary Figure 129. $^{1}$H NMR of ethyl 4-([1,1'-Biphenyl]-4-yl(hydroxy)-methyl)benzoate 3ah**

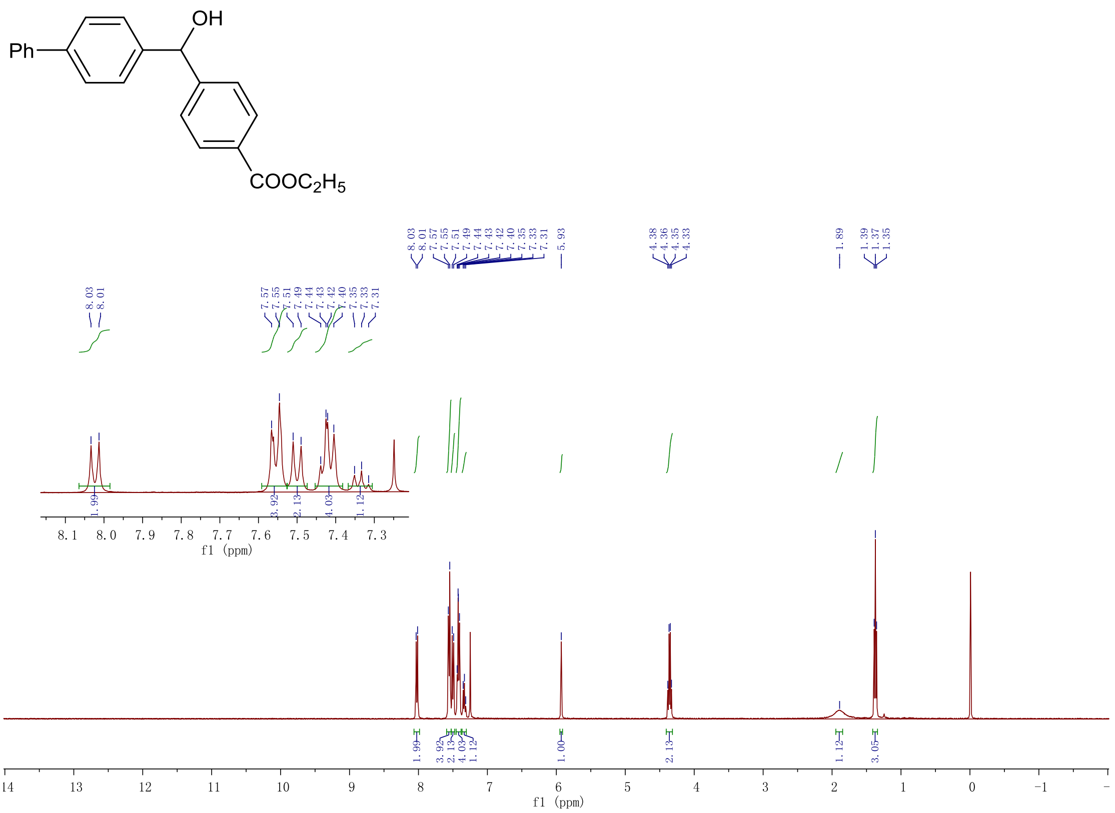

**Supplementary Figure 130. $^{13}$C NMR of ethyl 4-([1,1'-Biphenyl]-4-yl(hydroxy)-methyl)benzoate 3ah**

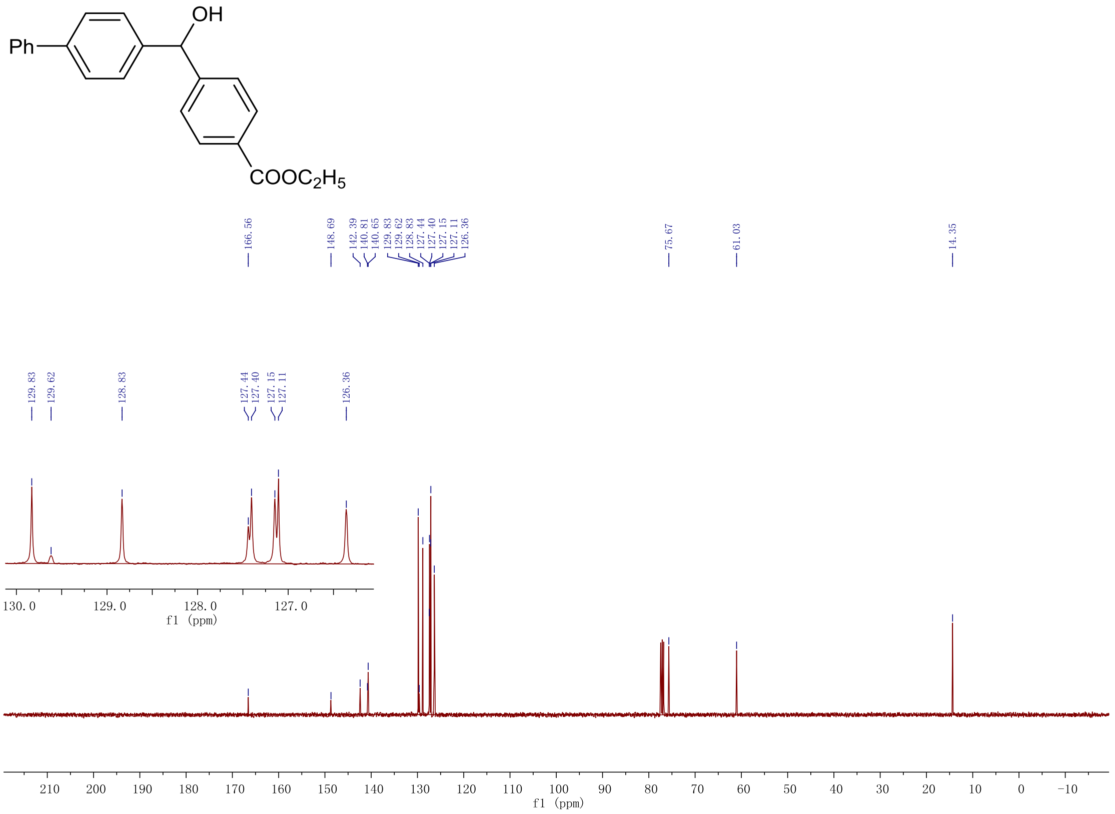

**Supplementary Figure 131. $^{1}$H NMR of 1-(4-([1,1'-Biphenyl]-4-yl(hydroxy)-methyl)phenyl)ethanone 3ai**

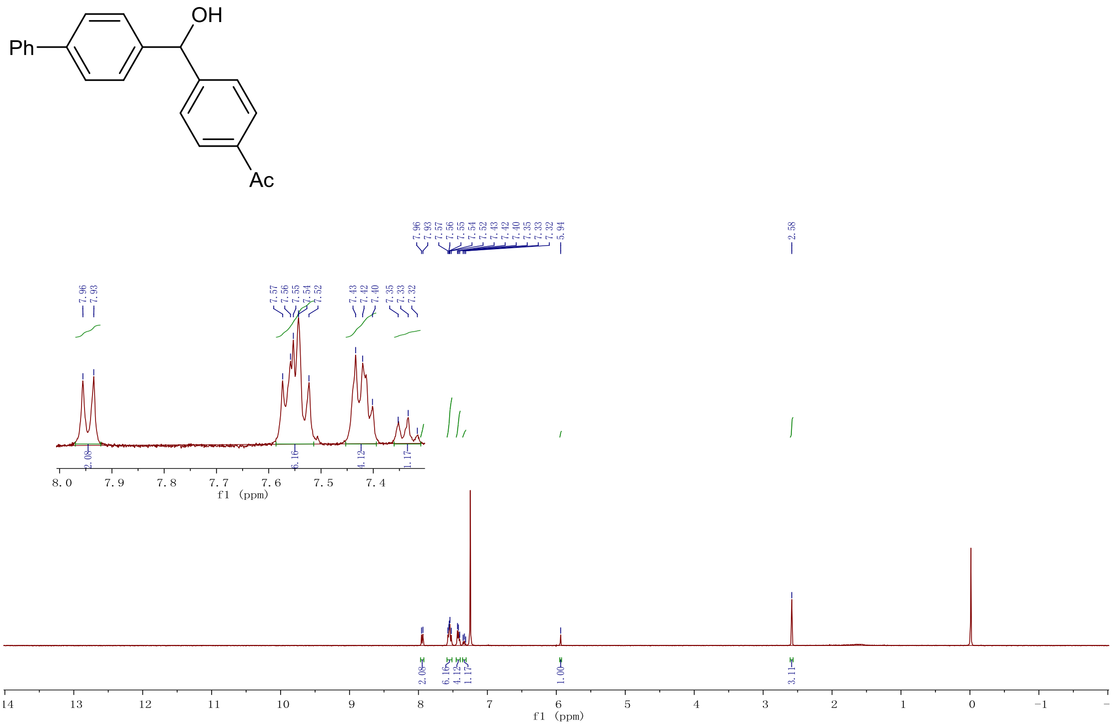


**Supplementary Figure 132. $^{13}$C NMR of 1-(4-([1,1'-Biphenyl]-4-yl(hydroxy)-methyl)phenyl)ethanone 3ai**

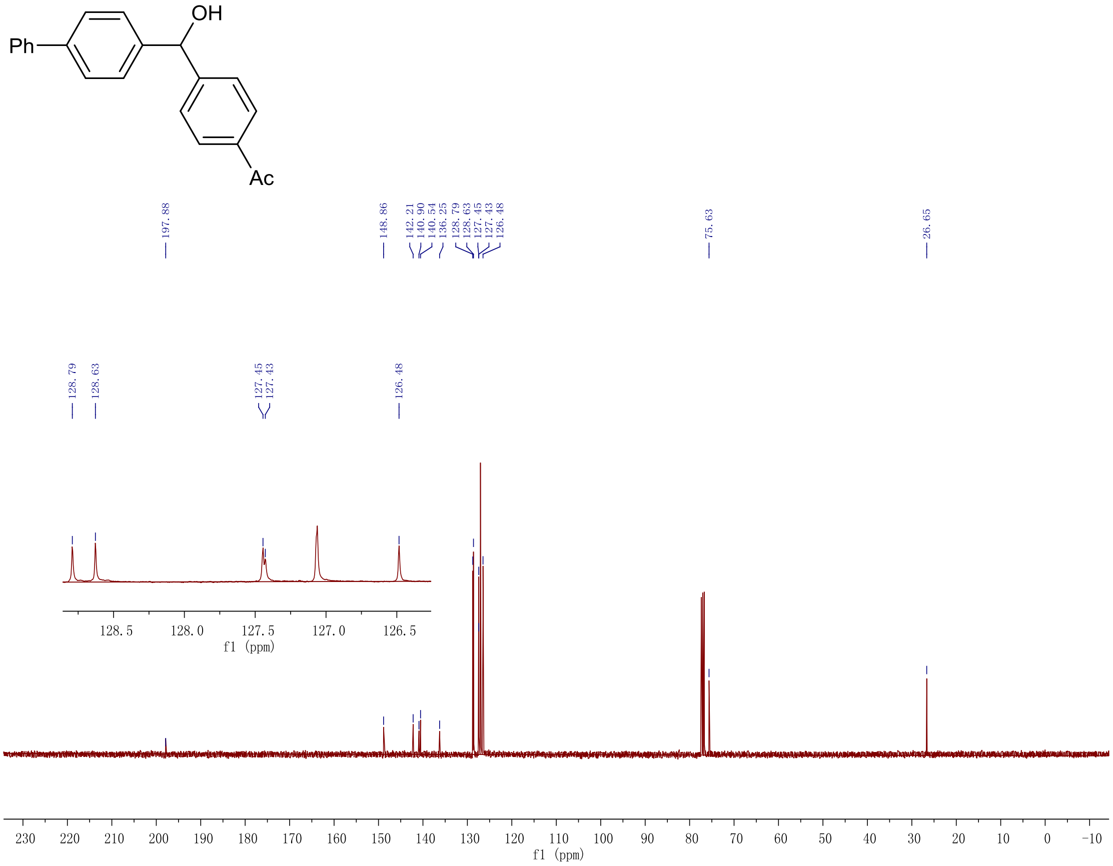

**Supplementary Figure 133. $^1$H NMR of [1,1'-Biphenyl]-4-yl(pyridin-2-yl)methanol 3aj**

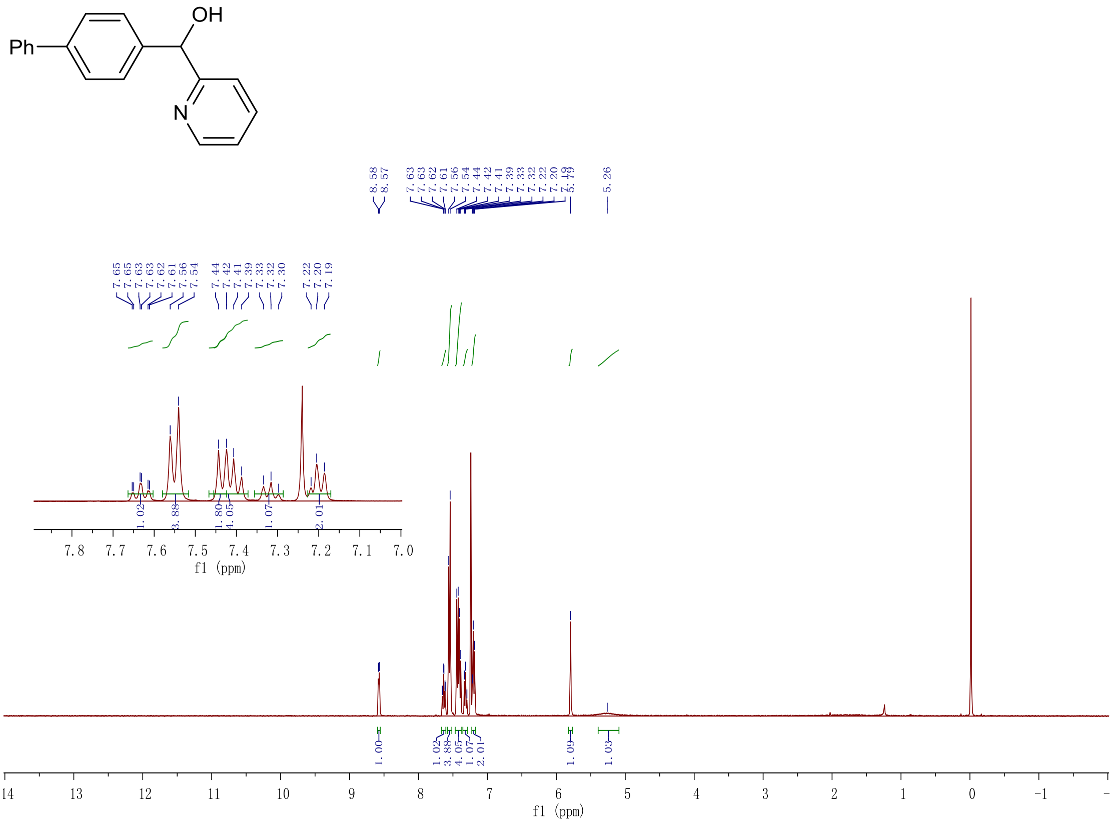

**Supplementary Figure 134. $^1$H NMR of $Ph_3P^{16}O/Ph_3P^{18}O$**

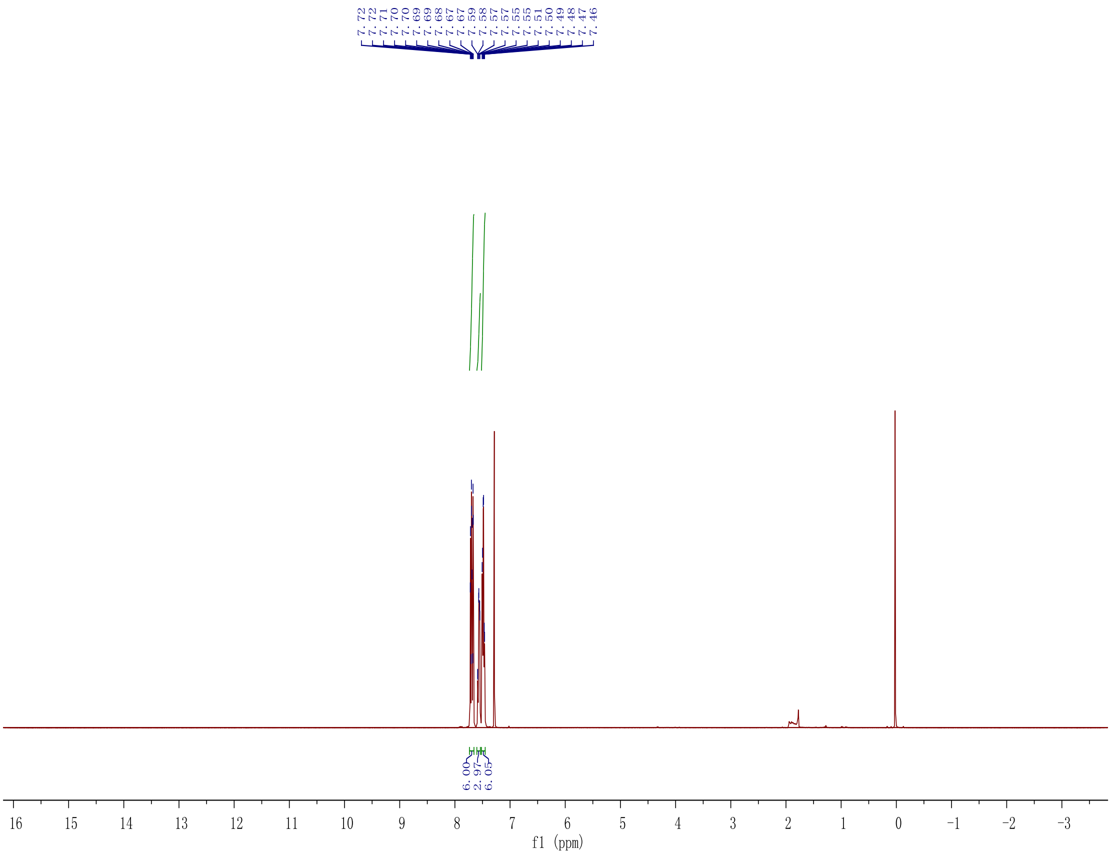

## Supplementary Figure 135. $^{31}$P NMR of $Ph_3P^{16}O/Ph_3P^{18}O$

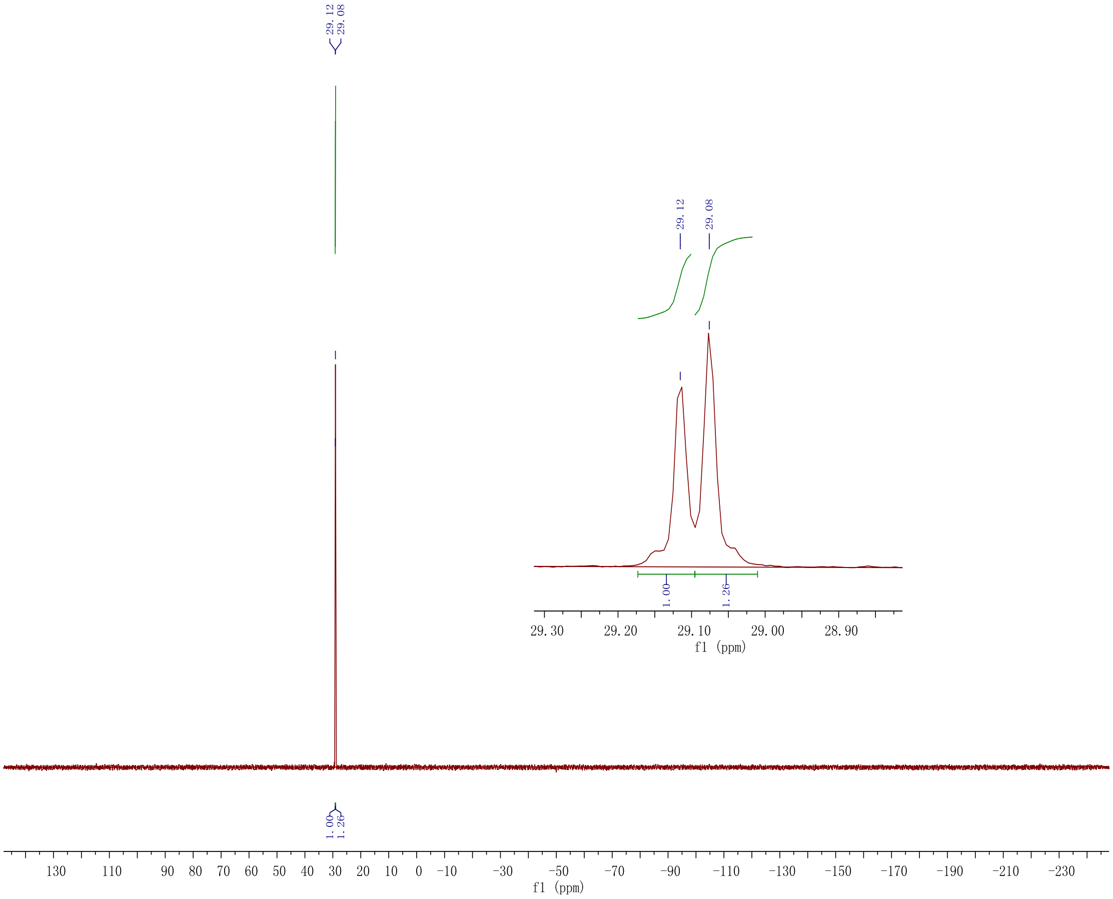


## Supplementary Figure 136. $^{13}$C NMR of $Ph_3P^{16}O/Ph_3P^{18}O$

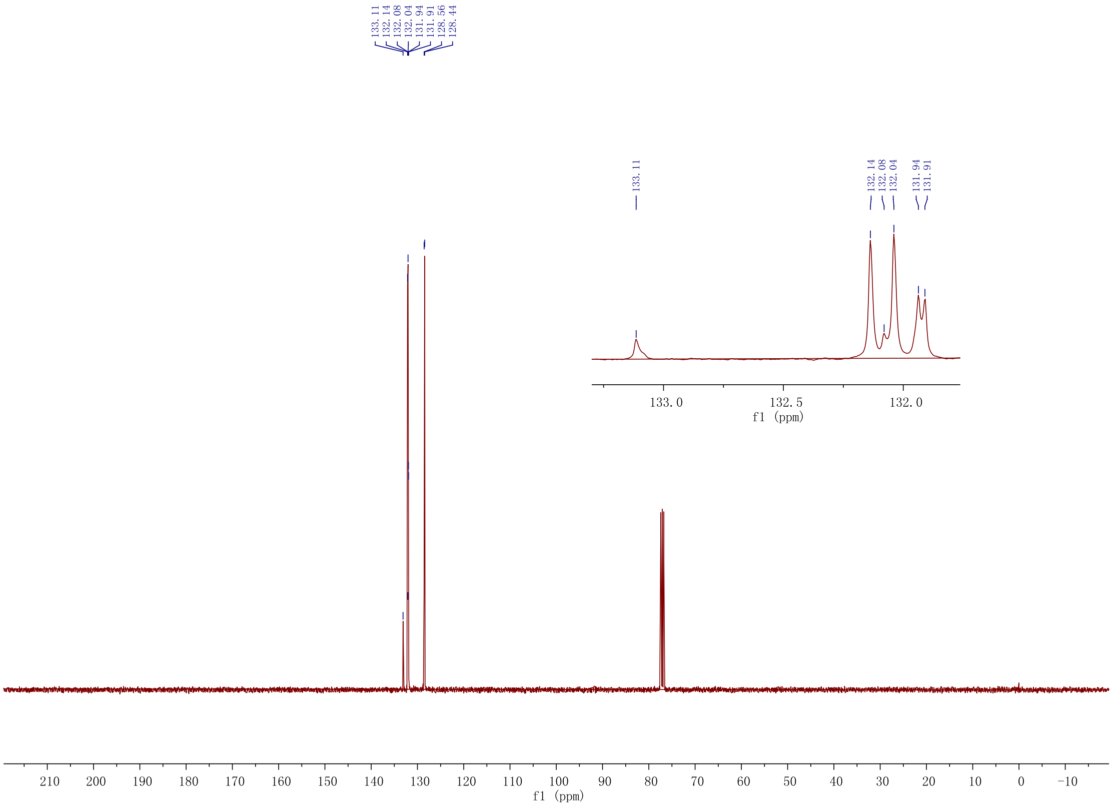

**Supplementary Figure 137.** $^{1}$**H NMR of** $Ph_5P$

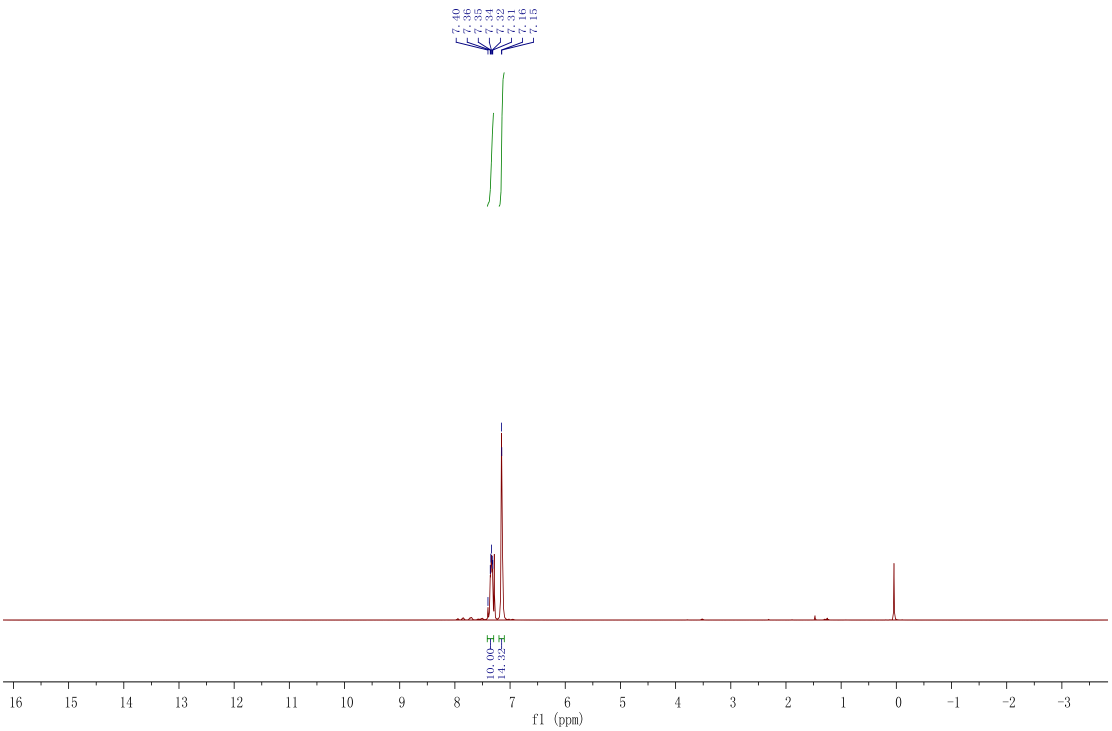


**Supplementary Figure 138.** $^{31}$**H NMR of** $Ph_5P$

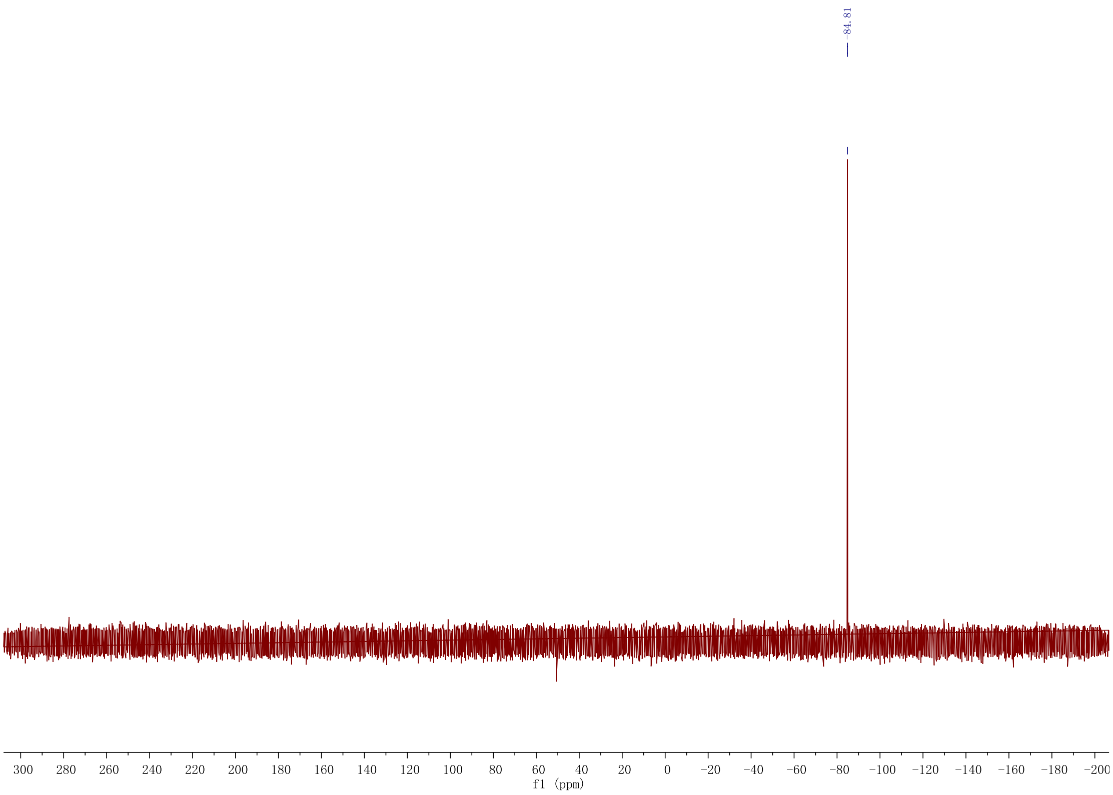

## Supplementary Figure 139. $^{13}$C NMR of $Ph_5P$

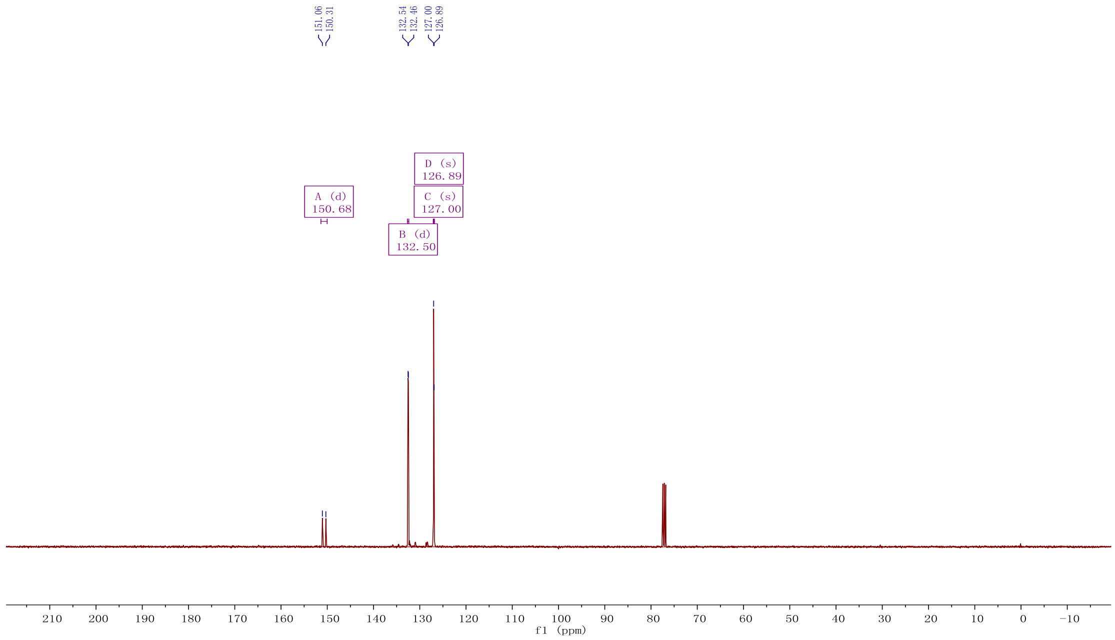


## Supplementary Figure 140. GC spectra

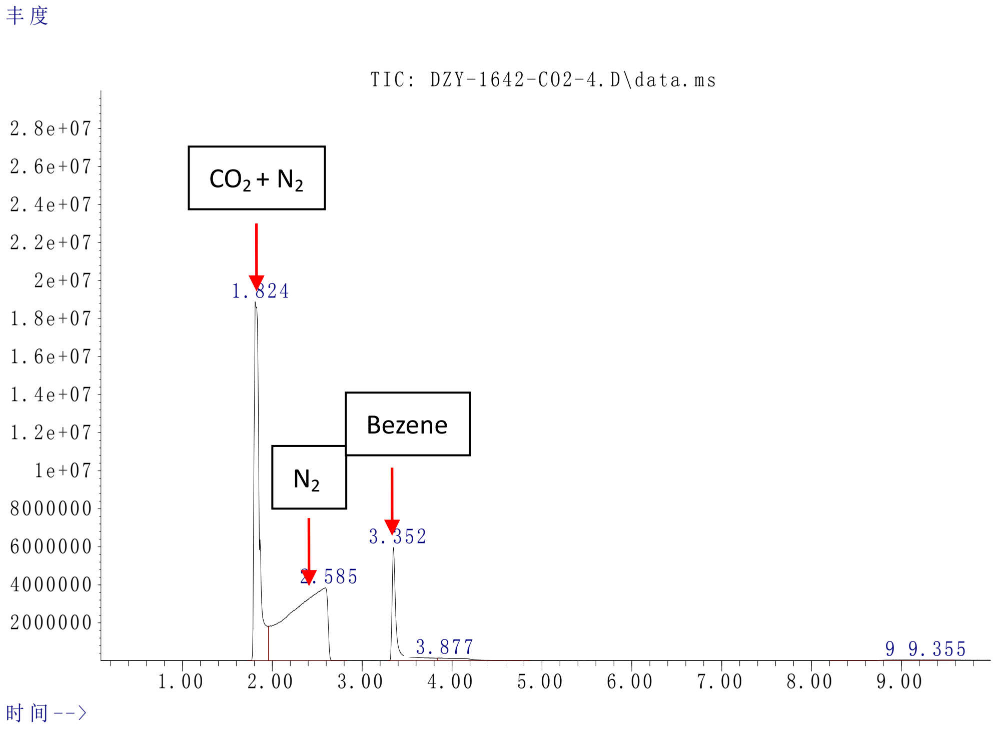

**Supplementary Figure 141. GC-MS spectra of $CO_2$ and $N_2$**

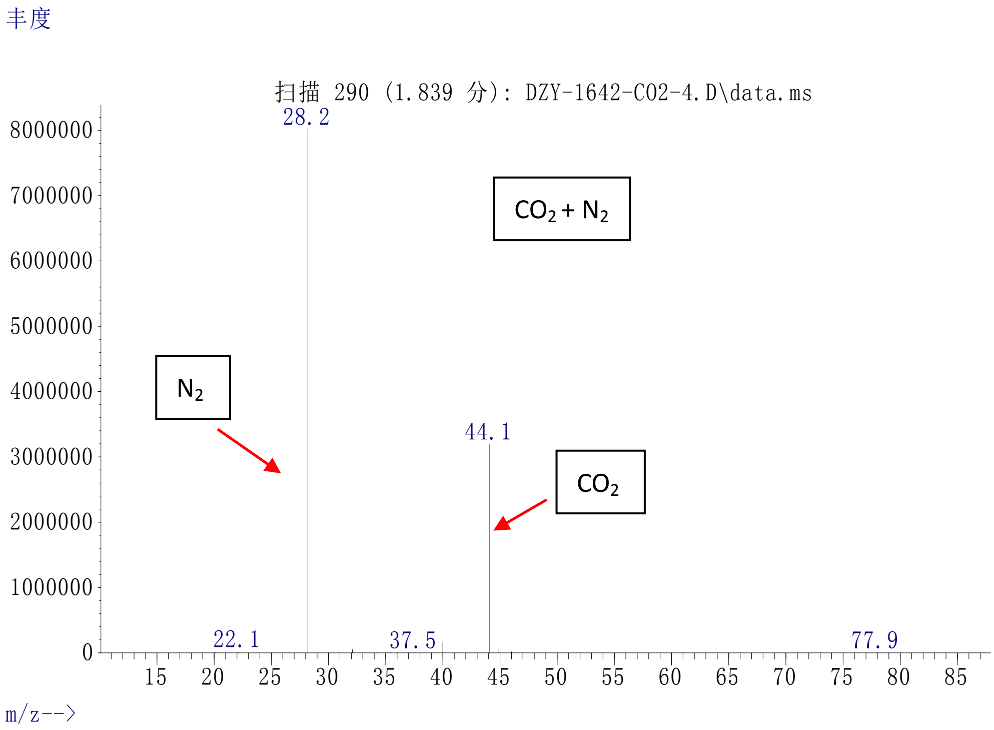


**Supplementary Figure 142. GC-MS spectra of $N_2$**

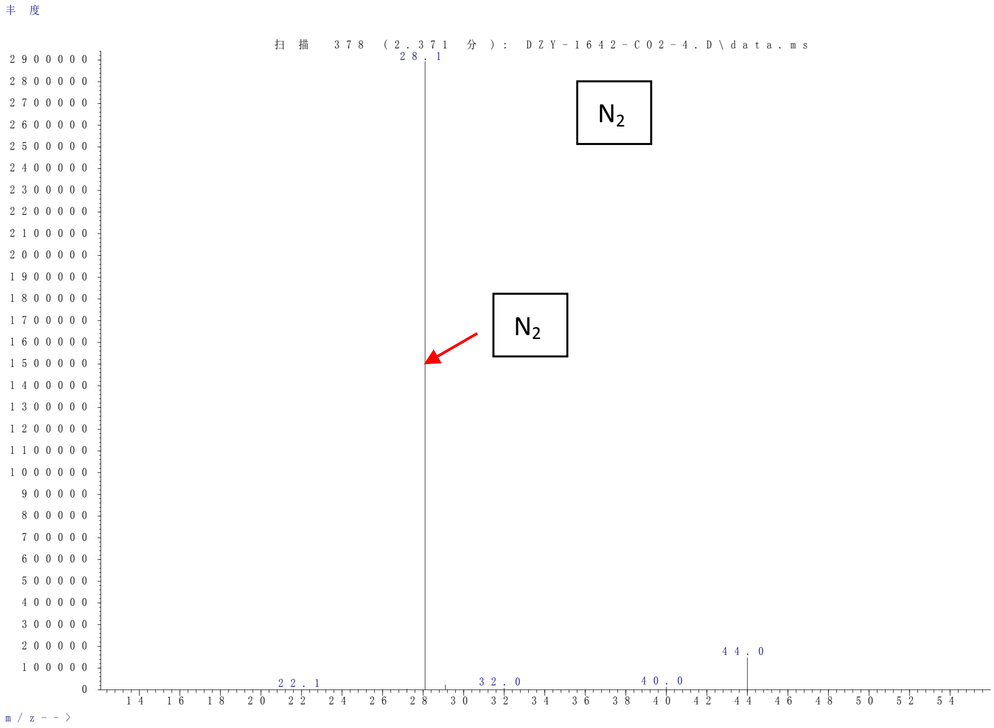

**Supplementary Figure 143. GC-MS spectra of Bezene**

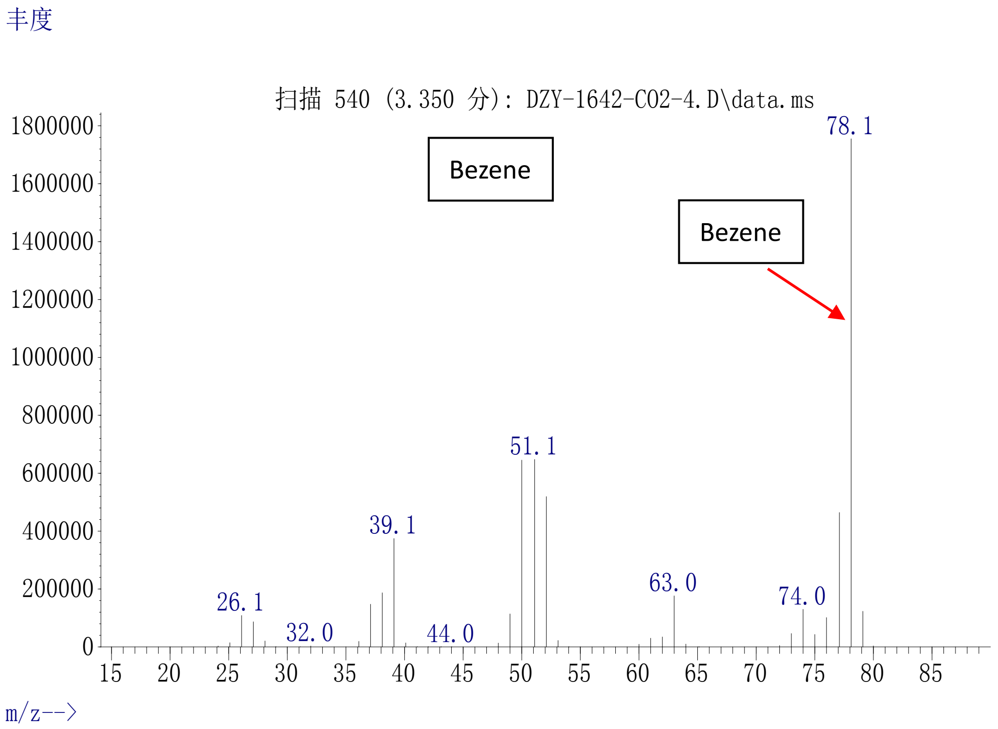


**Supplementary Figure 144(1). ICP-OES spectra of Pd in tetraphenylphosphonium iodide 2a.**

**Pd**

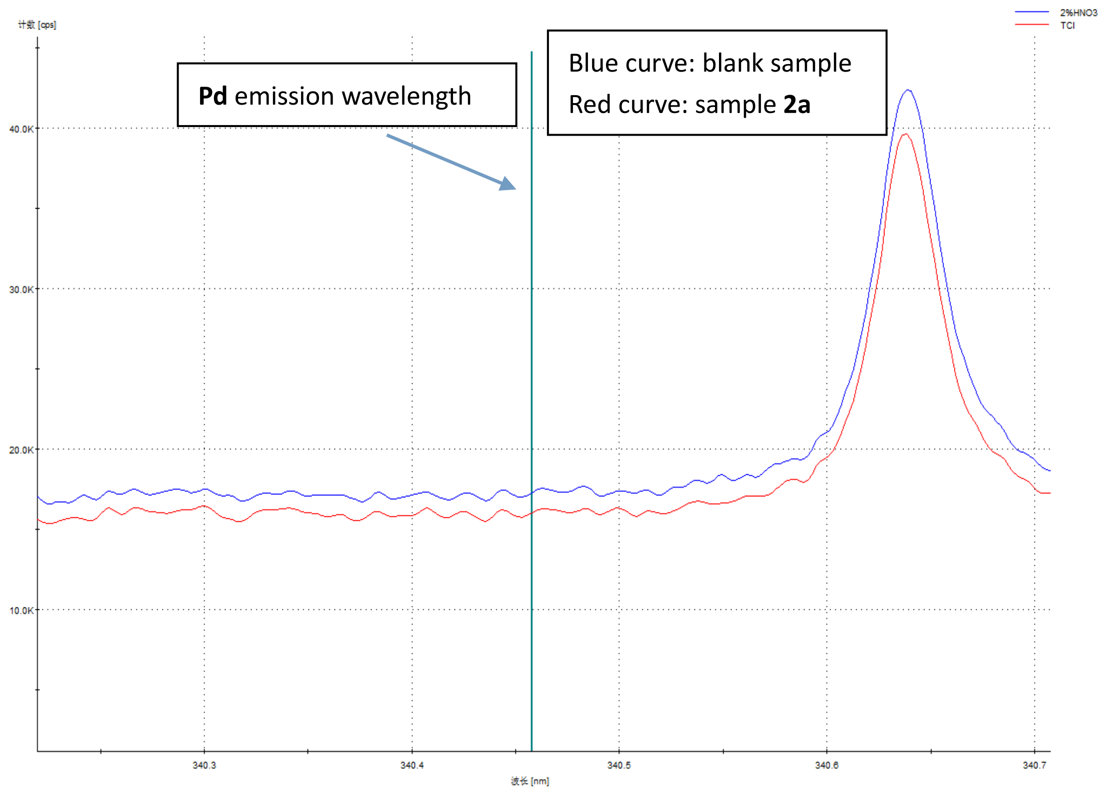

**Supplementary Figure 144(2). ICP-OES spectra of Pd in tetraphenylphosphonium iodide 2a**

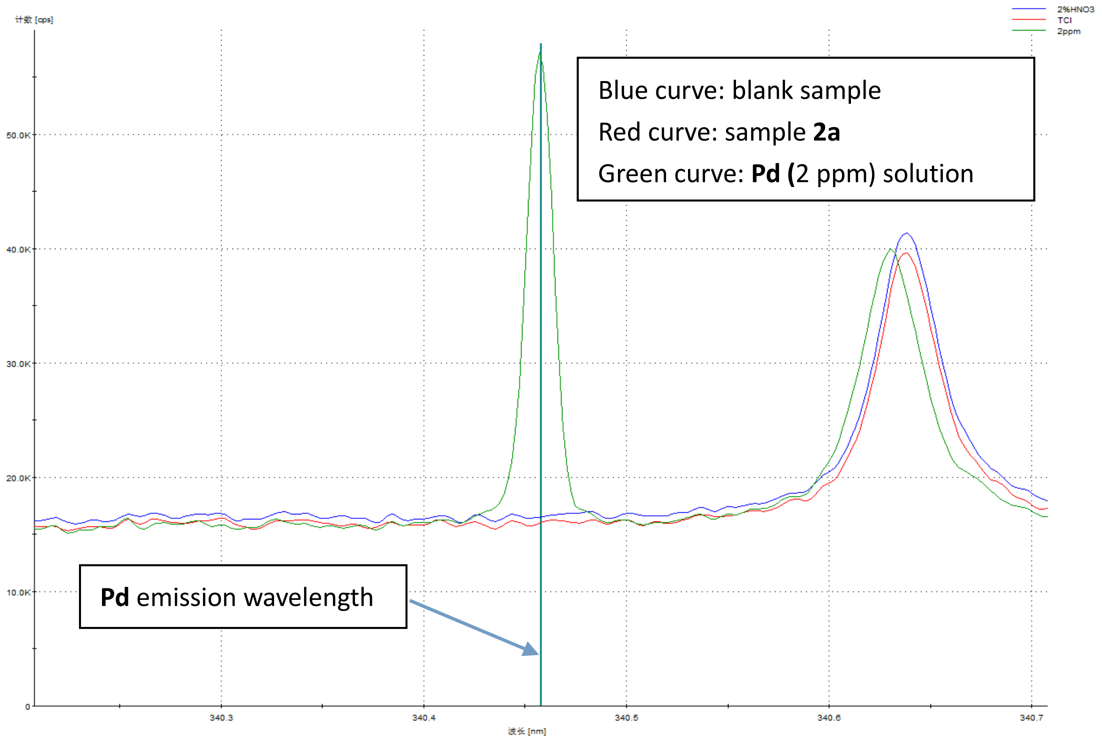


**Supplementary Figure 145(1). ICP-OES spectra of Fe in tetraphenylphosphonium iodide 2a**

**Fe**

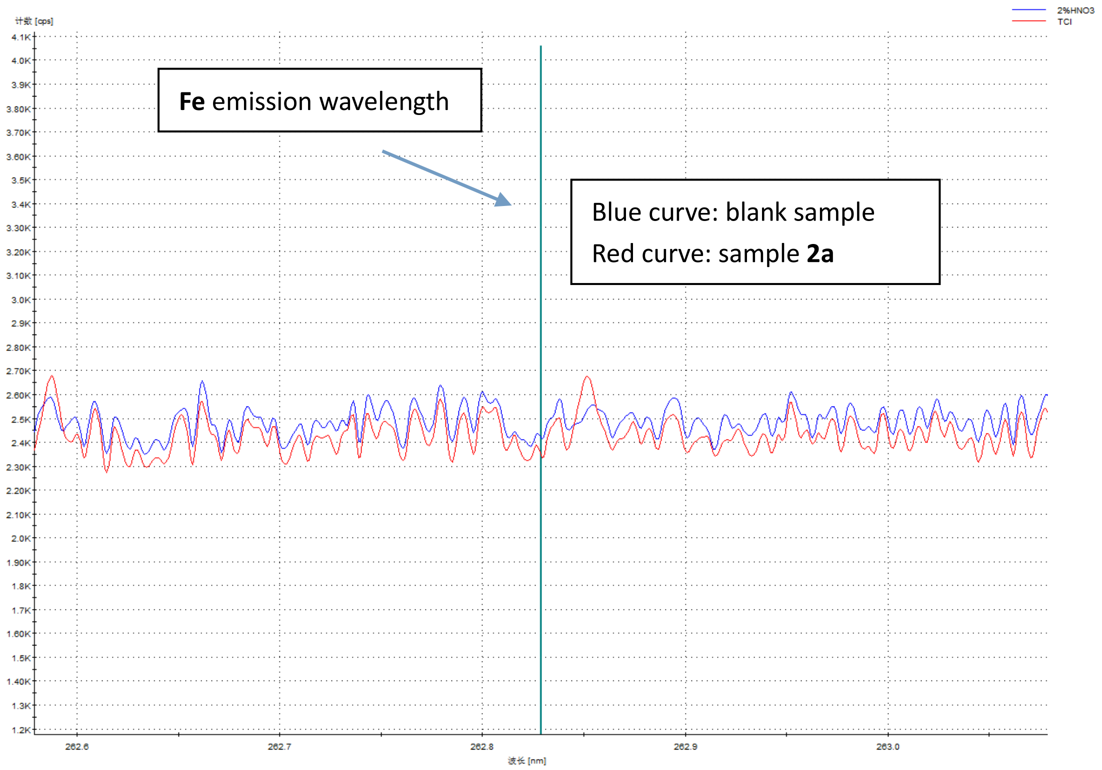

**Supplementary Figure 145(2). ICP-OES spectra of Fe in tetraphenylphosphonium iodide 2a**

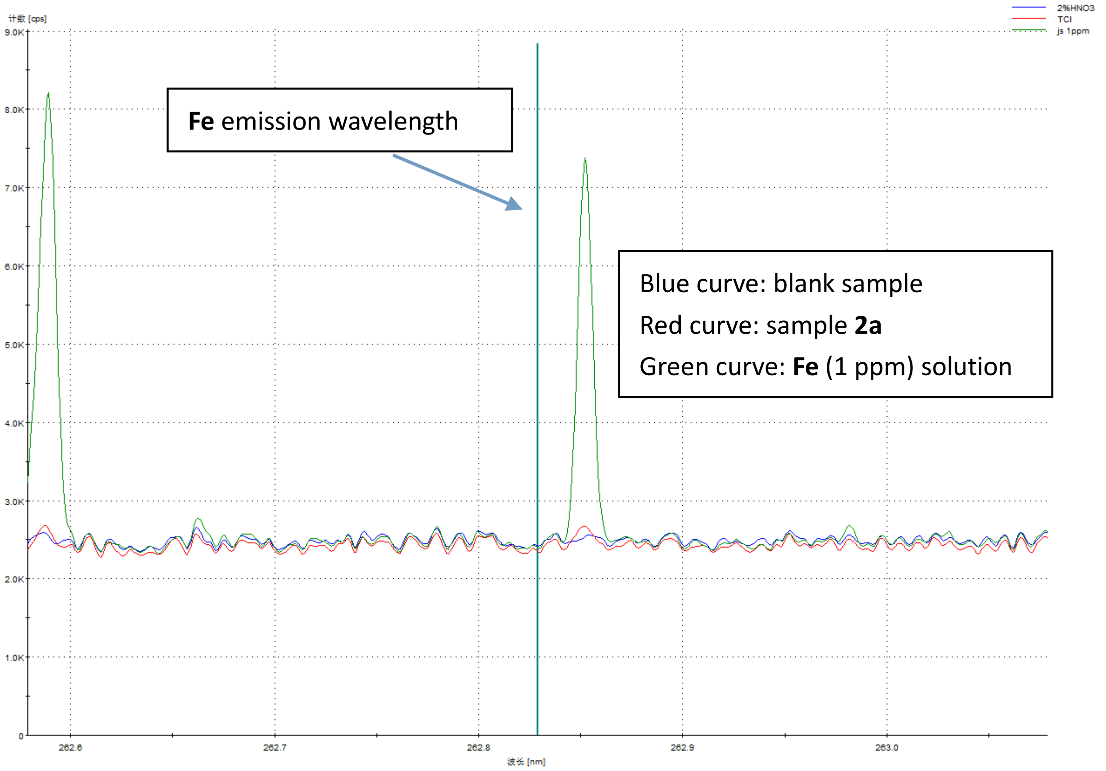


**Supplementary Figure 146(1). ICP-OES spectra of Co in tetraphenylphosphonium iodide 2a**

**Co**

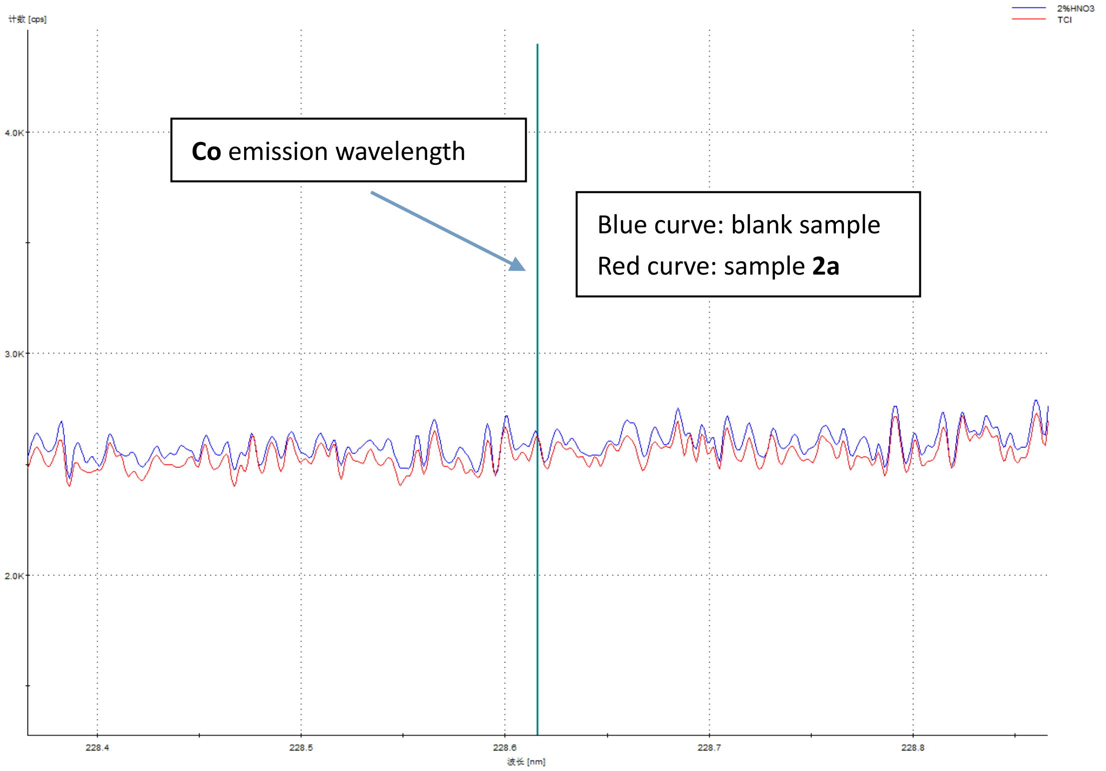

**Supplementary Figure 146(2). ICP-OES spectra of Co in tetraphenylphosphonium iodide 2a**

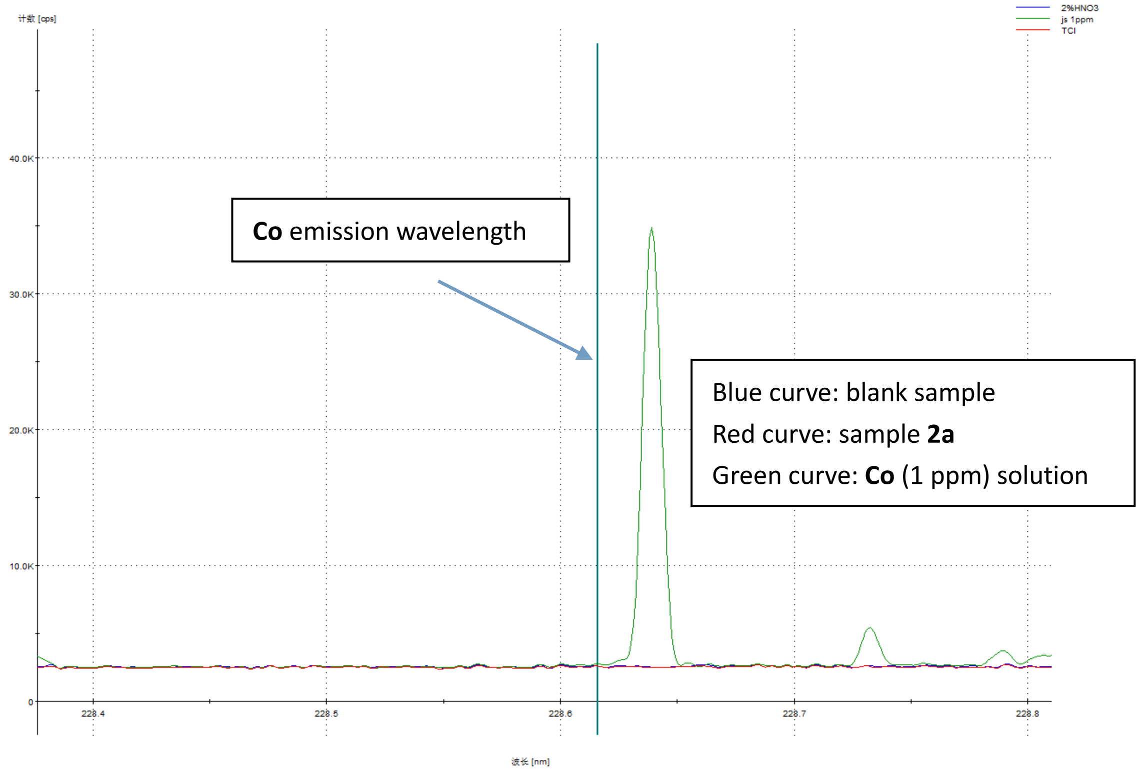


**Supplementary Figure 147(1). ICP-OES spectra of Ni in tetraphenylphosphonium iodide 2a**

**Ni**

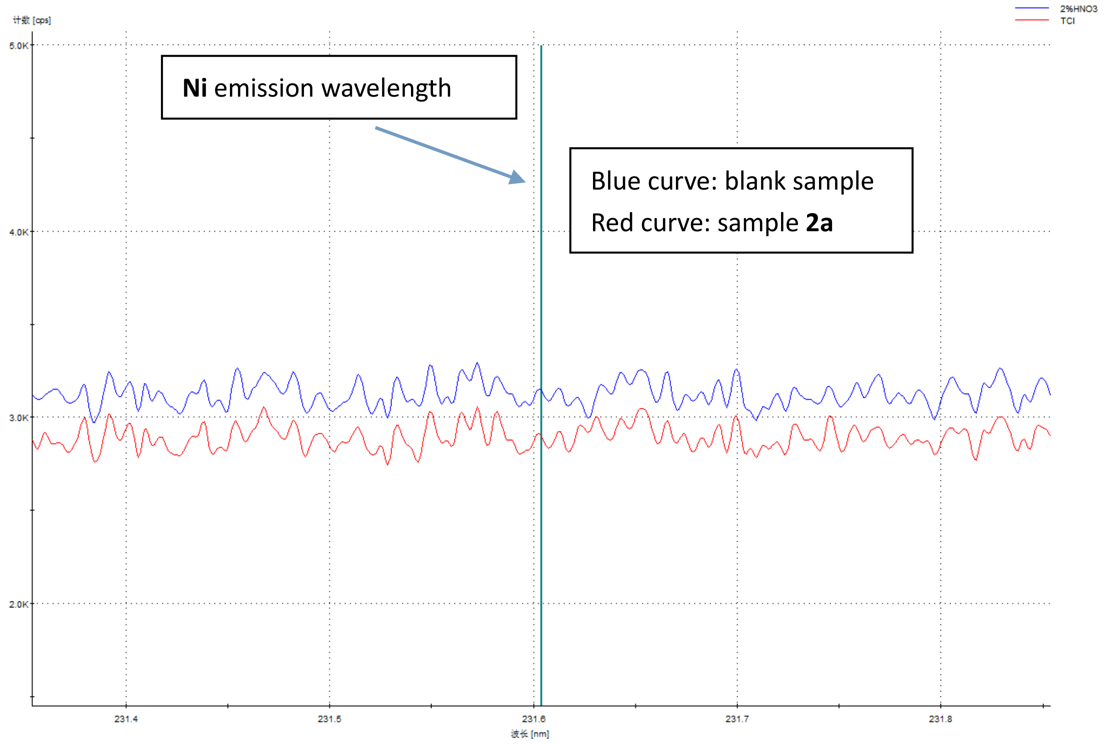

**Supplementary Figure 147(2). ICP-OES spectra of Ni in tetraphenylphosphonium iodide 2a**

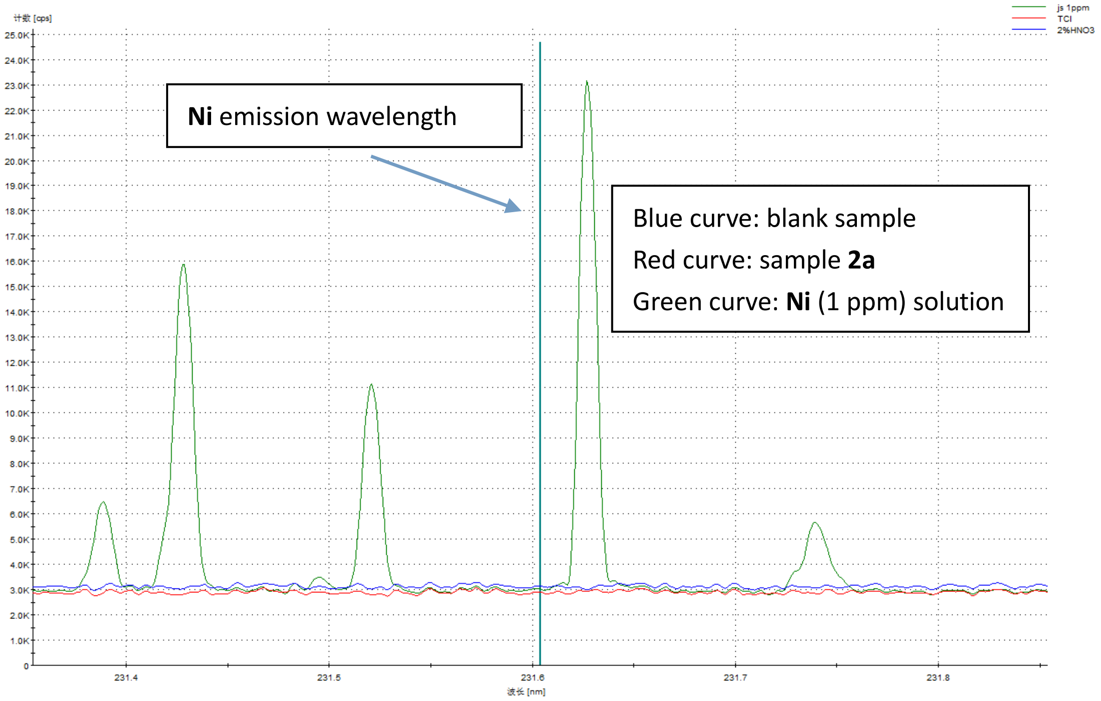


**Supplementary Figure 148(1). ICP-OES spectra of Cu in tetraphenylphosphonium iodide 2a**

**Cu**

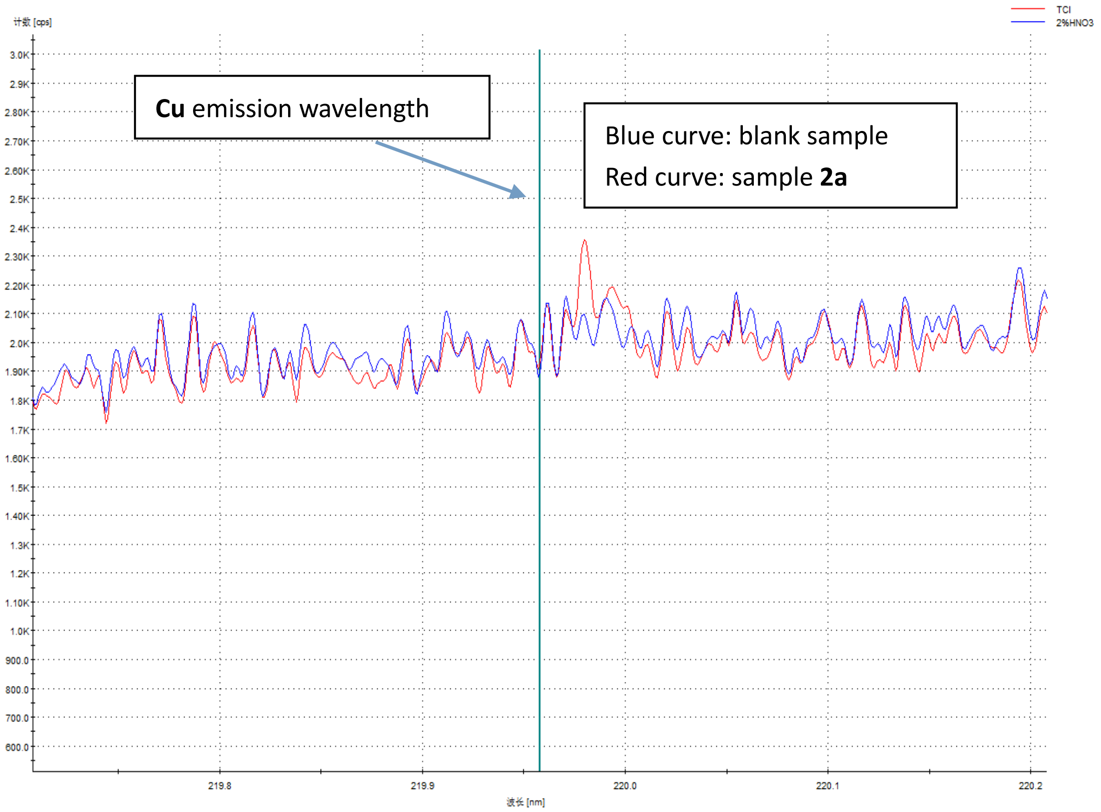

**Supplementary Figure 148(2). ICP-OES spectra of Cu in tetraphenylphosphonium iodide 2a**

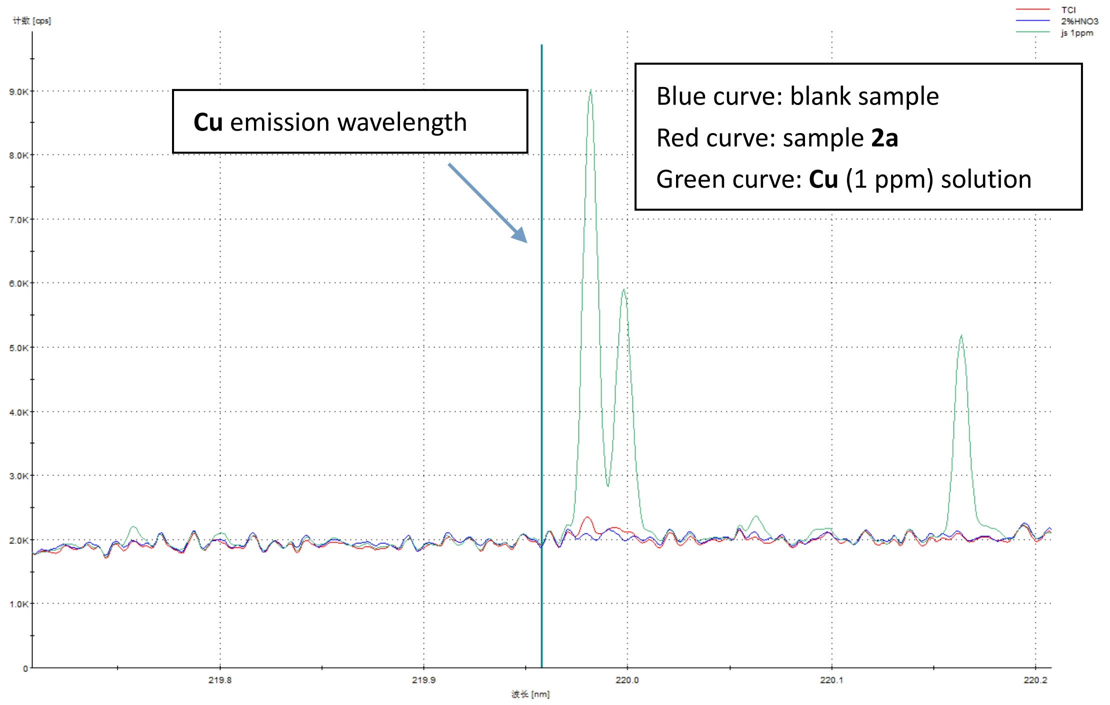


**Supplementary Figure 149(1). ICP-OES spectra of Zn in tetraphenylphosphonium iodide 2a**

**Zn**

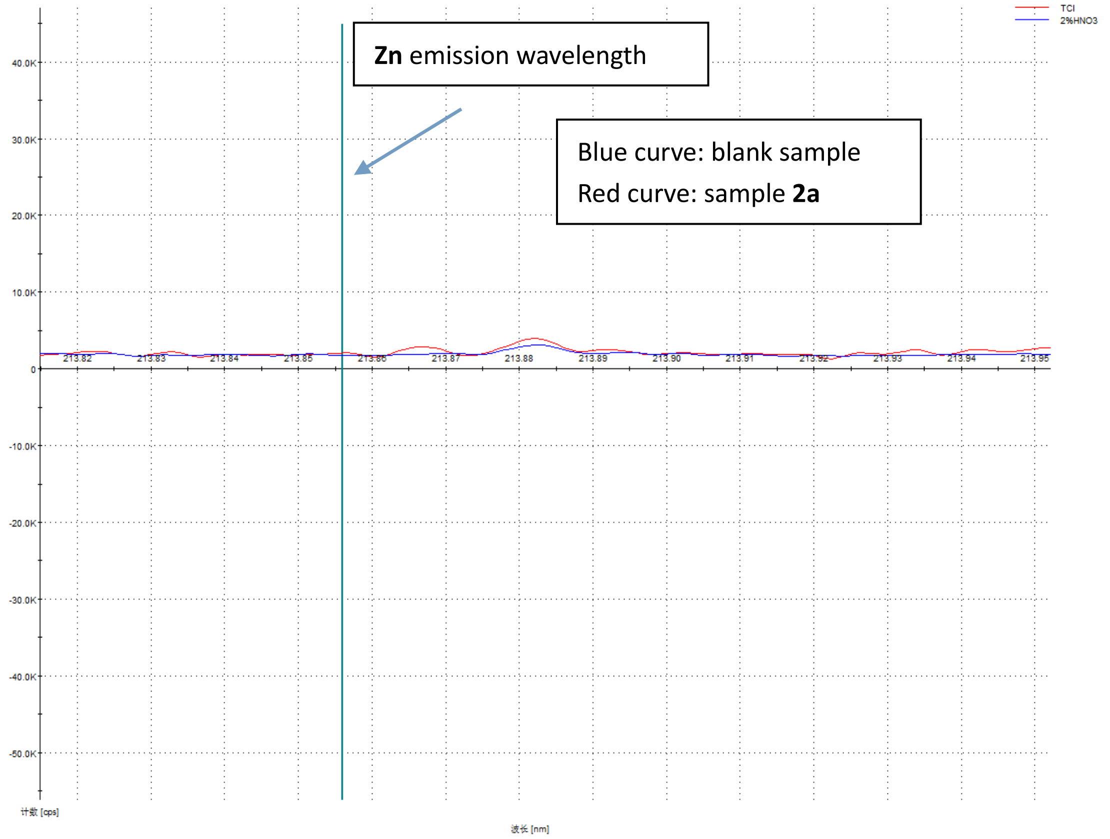

**Supplementary Figure 149(2). ICP-OES spectra of Zn in tetraphenylphosphonium iodide 2a**

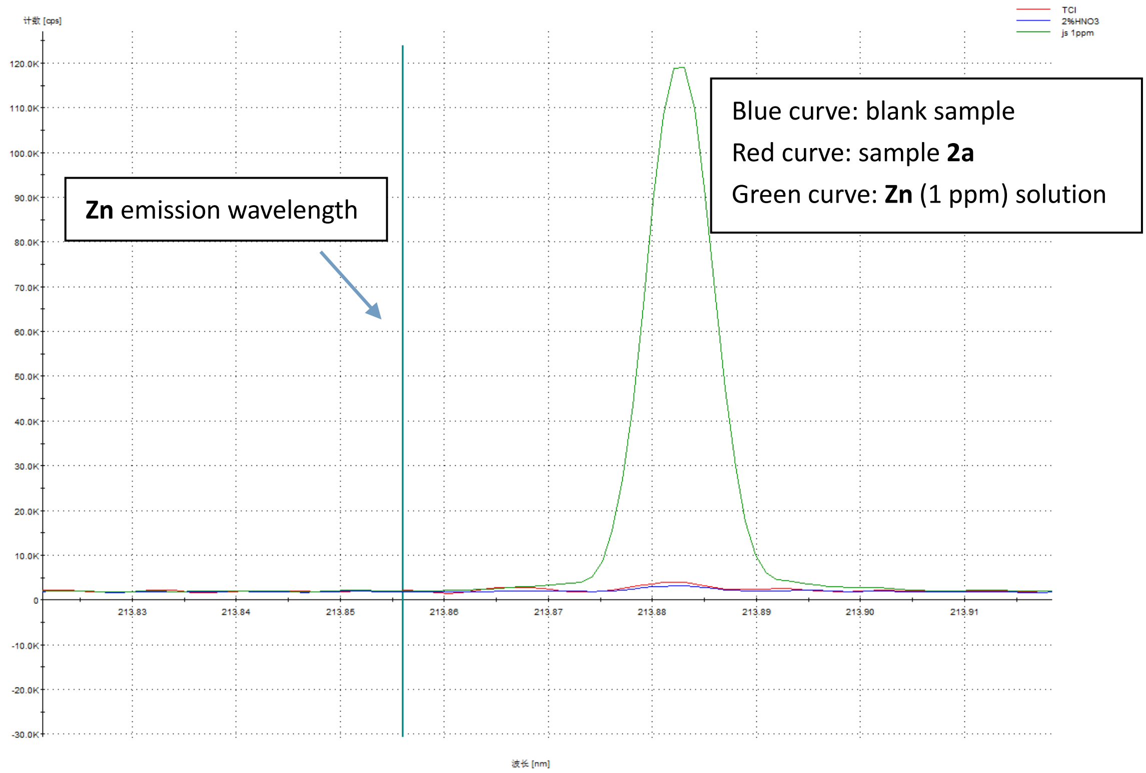


**Supplementary Figure 150. ICP-OES spectra of Ru in tetraphenylphosphonium iodide 2a**

**Ru**

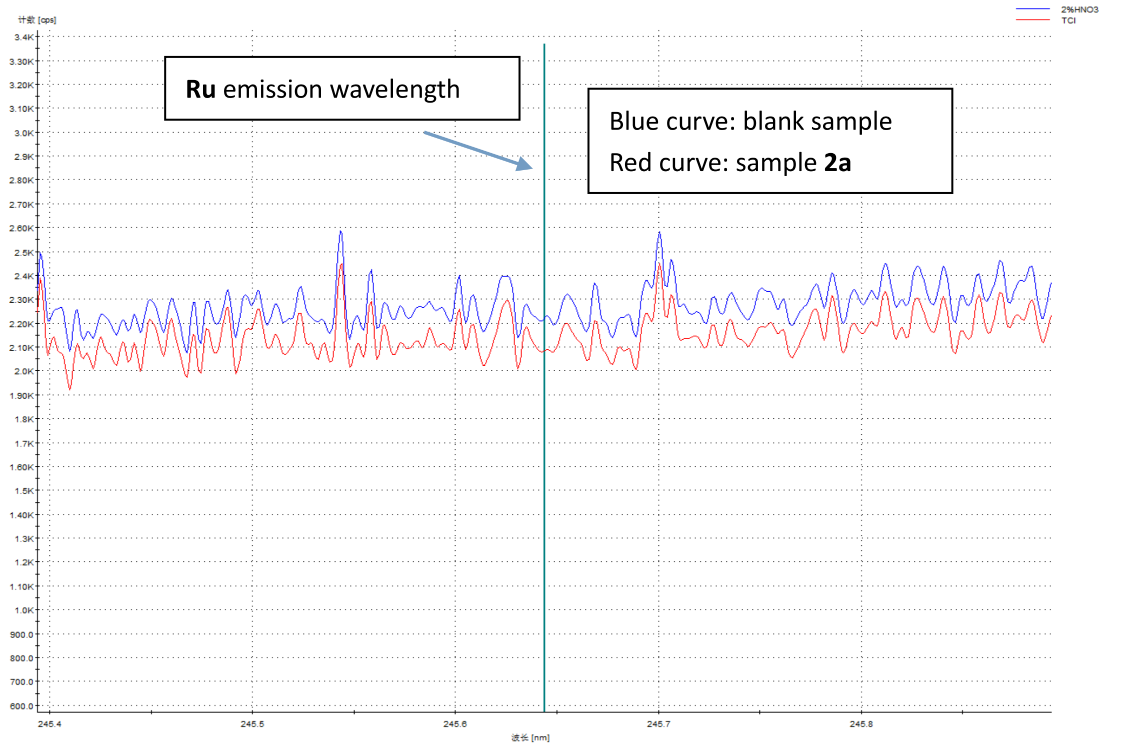

**Supplementary Figure 151. ICP-OES spectra of Rh in tetraphenylphosphonium iodide 2a**

**Rh**

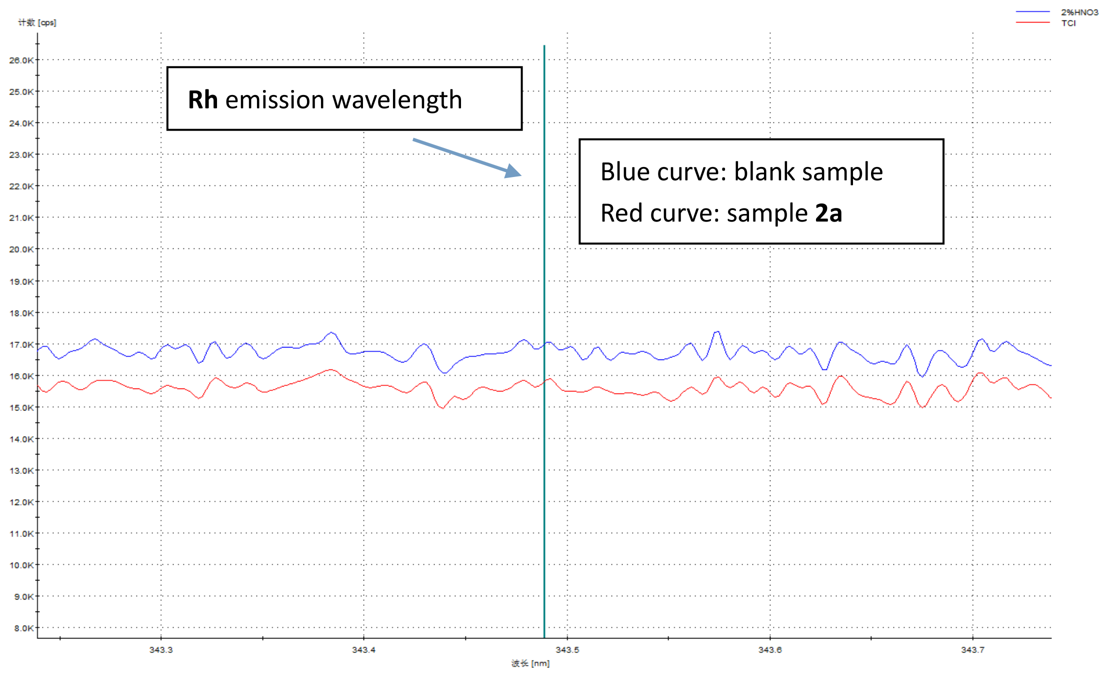


**Supplementary Figure 152. ICP-OES spectra of Ag in tetraphenylphosphonium iodide 2a**

**Ag**

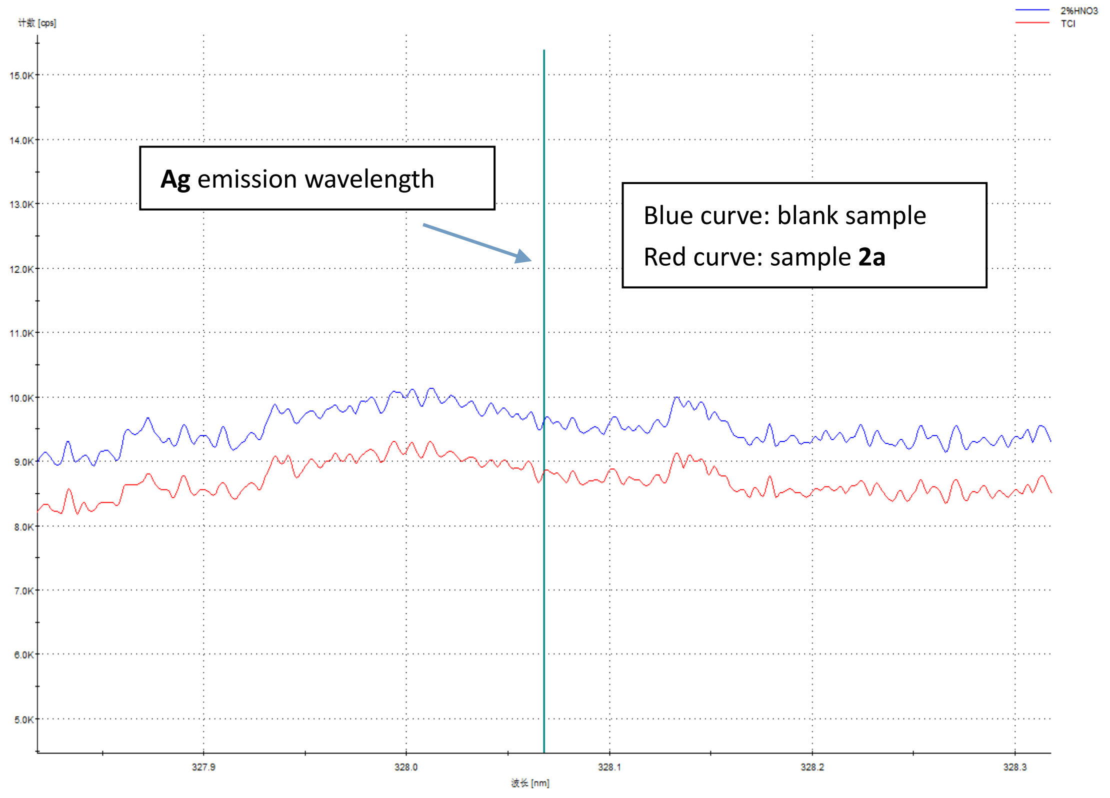

**Supplementary Figure 153(1). ICP-OES spectra of Cd in tetraphenylphosphonium iodide 2a**

**Cd**

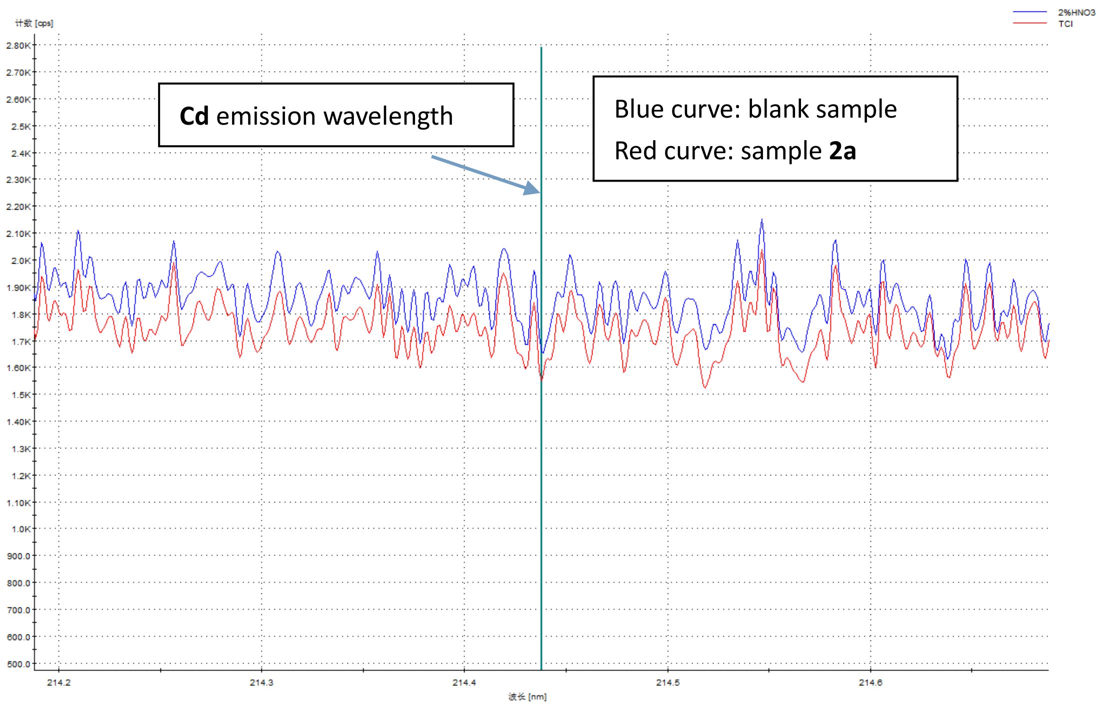


**Supplementary Figure 153(2). ICP-OES spectra of Cd in tetraphenylphosphonium iodide 2a**

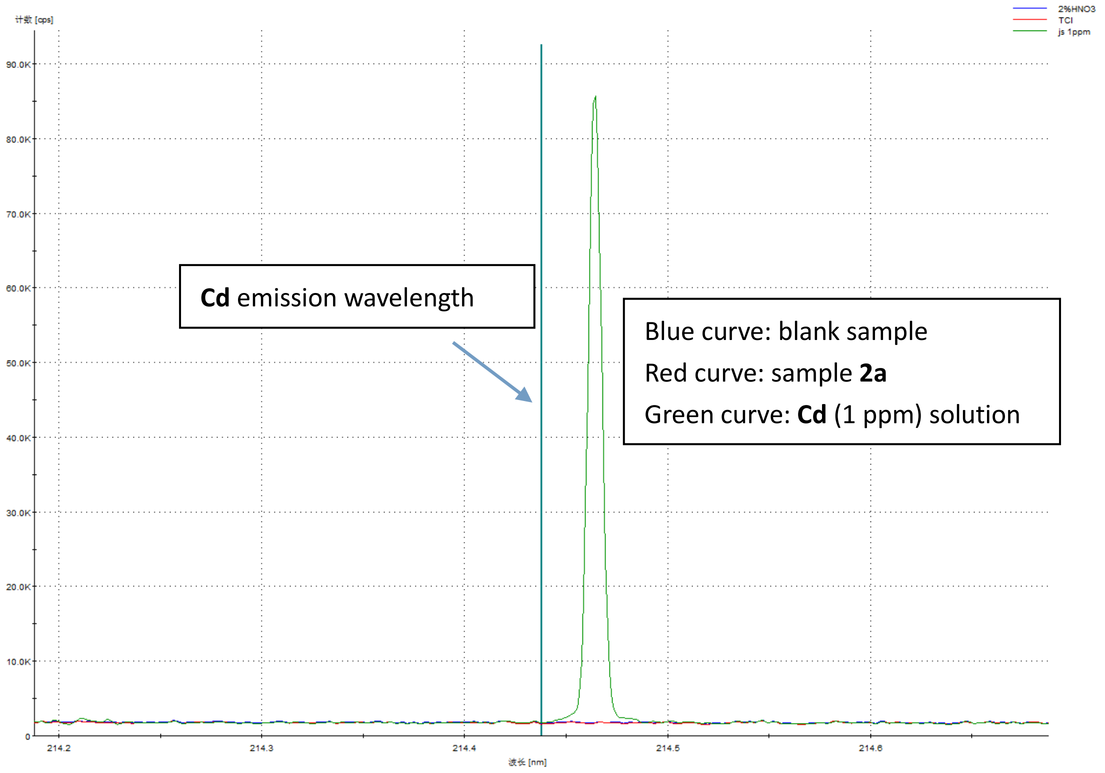

**Supplementary Figure 154. ICP-OES spectra of Os in tetraphenylphosphonium iodide 2a**

**Os**

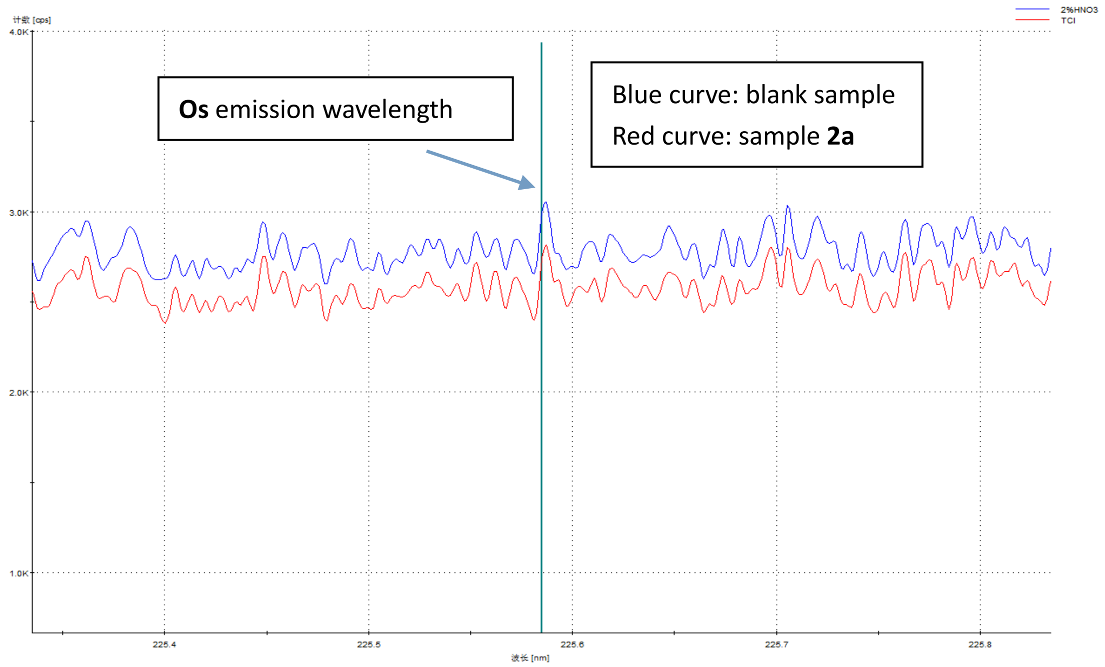


**Supplementary Figure 155. ICP-OES spectra of Ir in tetraphenylphosphonium iodide 2a**

**Ir**

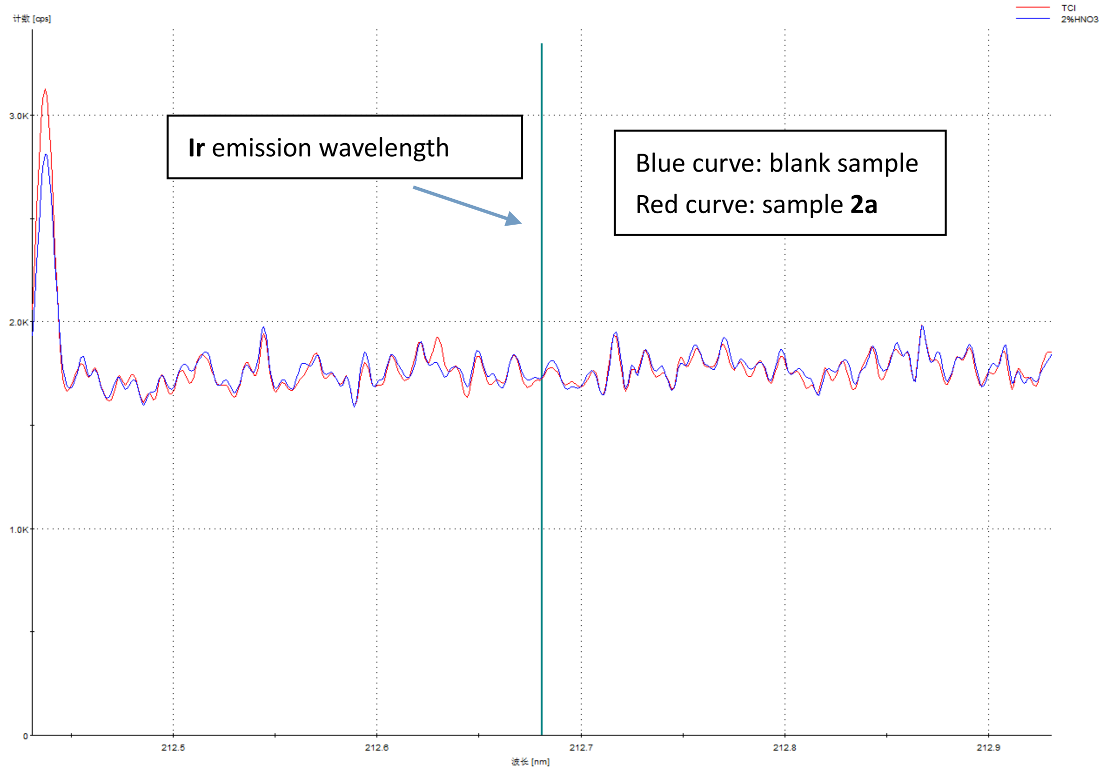

**Supplementary Figure 156. ICP-OES spectra of Pt in tetraphenylphosphonium iodide 2a**

**Pt**

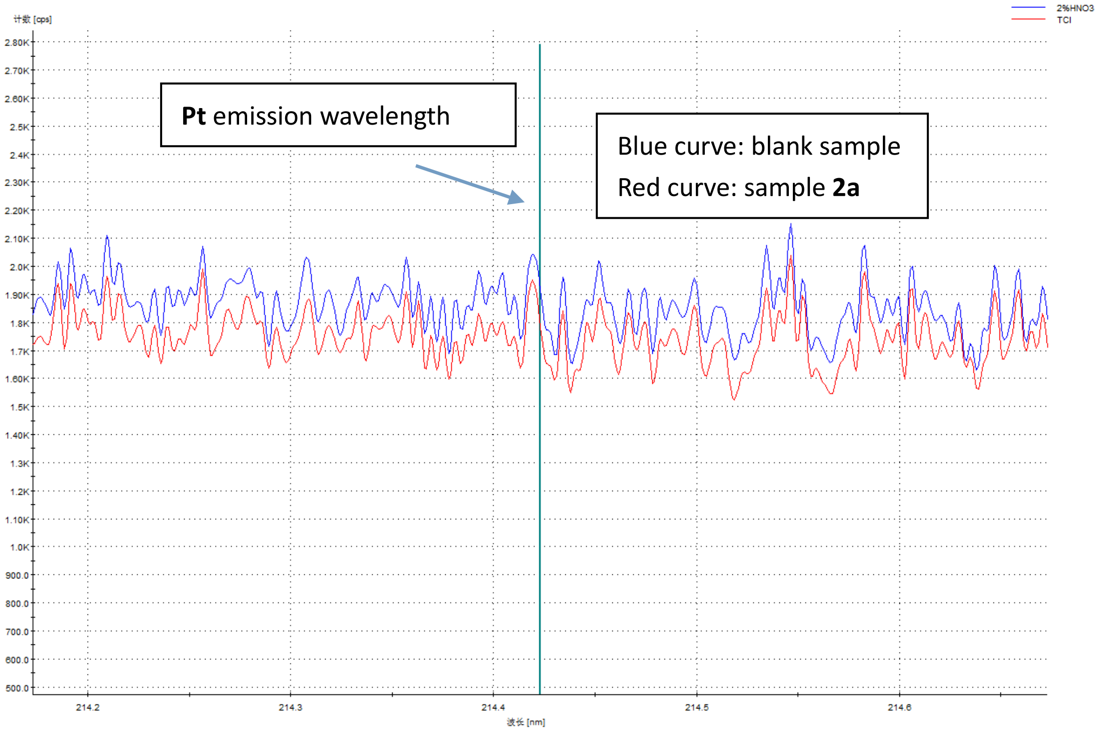


**Supplementary Figure 157. ICP-OES spectra of Au in tetraphenylphosphonium iodide 2a**

**Au**

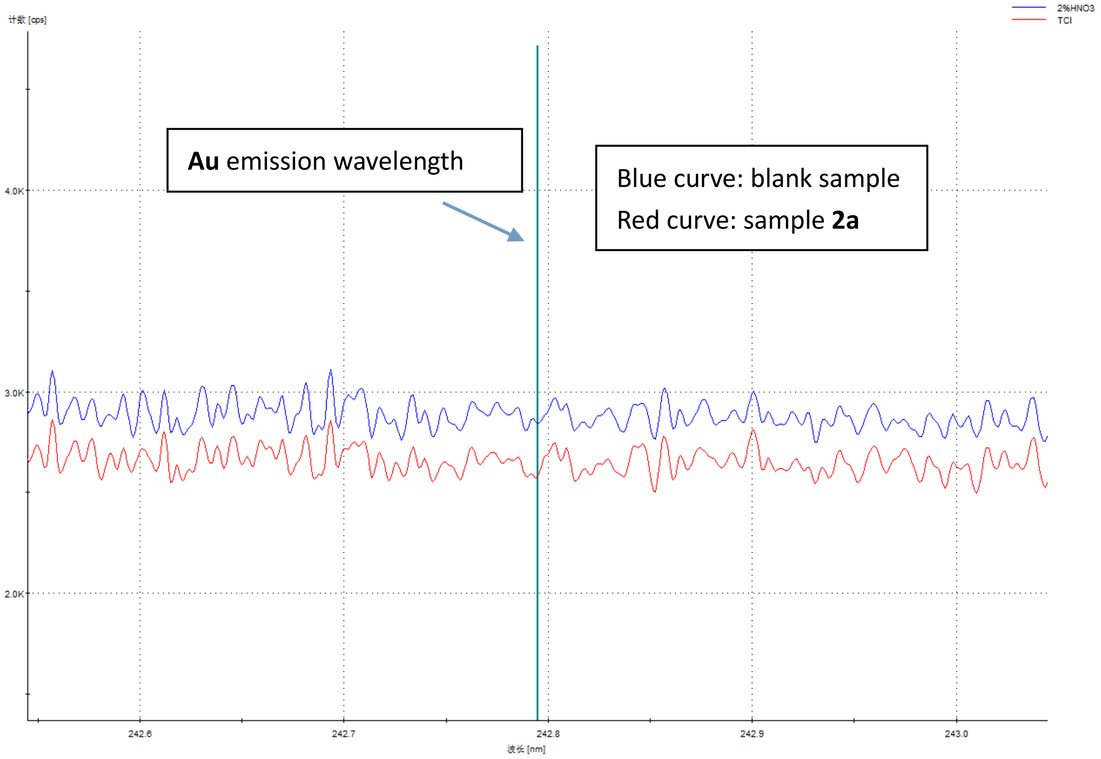

**Supplementary Figure 158. ICP-OES spectra of Hg in tetraphenylphosphonium iodide 2a**

**Hg**

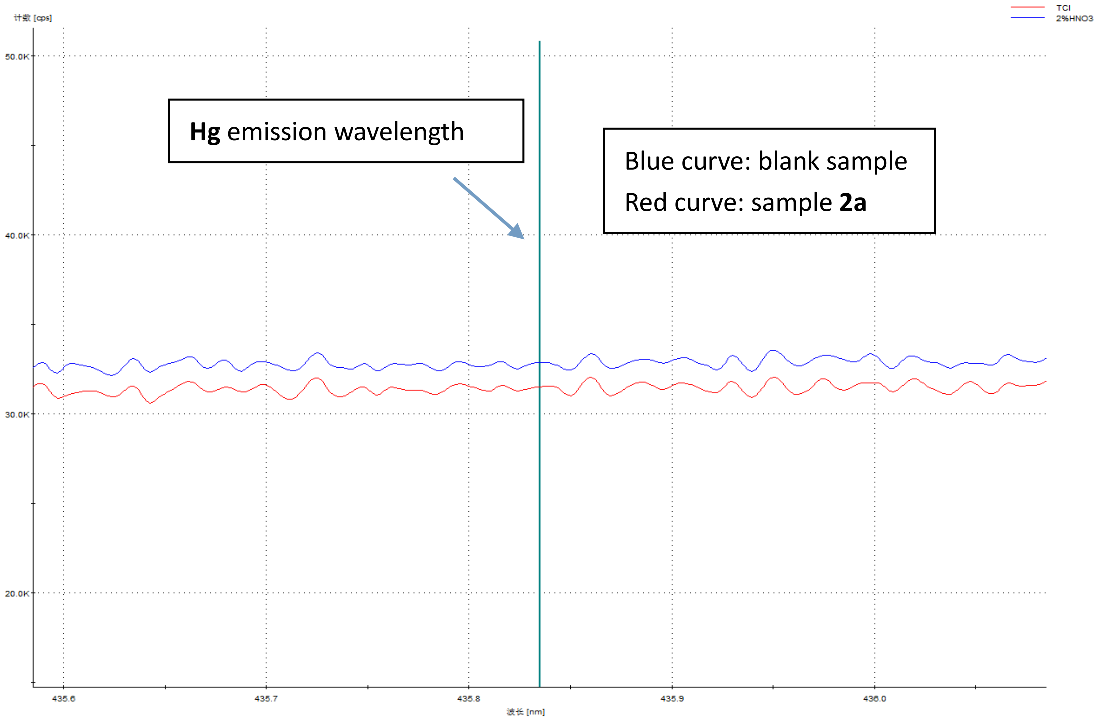


**Supplementary Figure 159(1). ICP-OES spectra of Pd in tetrakis(4-methoxyphenyl)phosphonium iodide 2b**

**Pd**

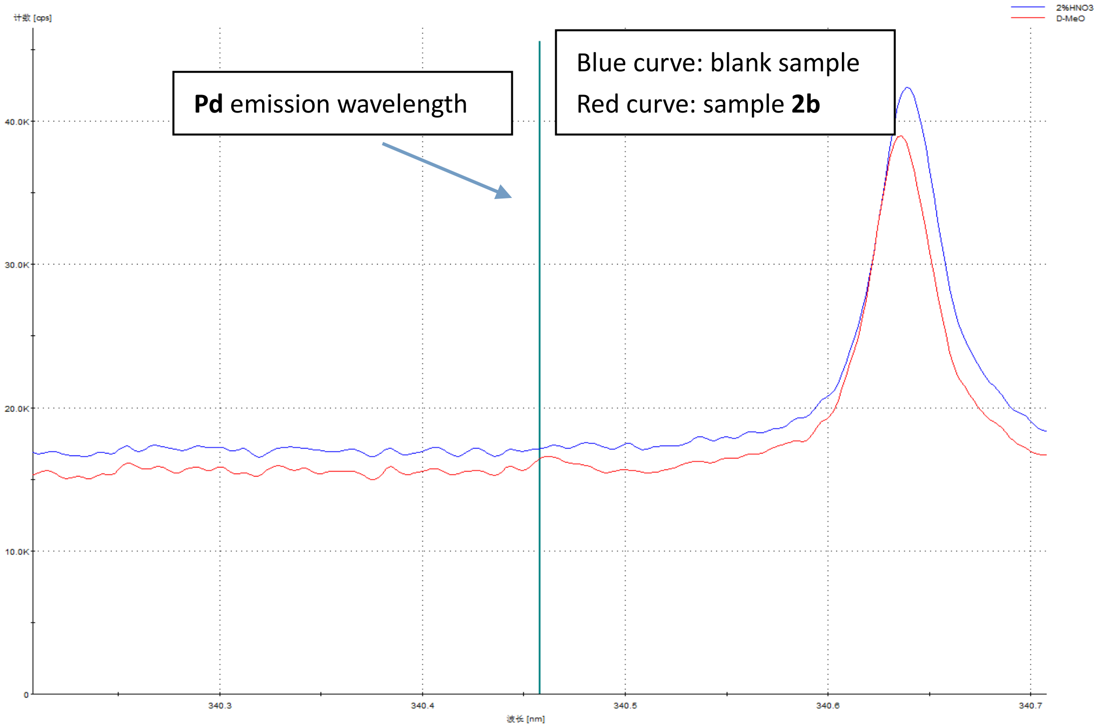

**Supplementary Figure 159(2). ICP-OES spectra of Pd in tetrakis(4-methoxyphenyl)phosphonium iodide 2b**

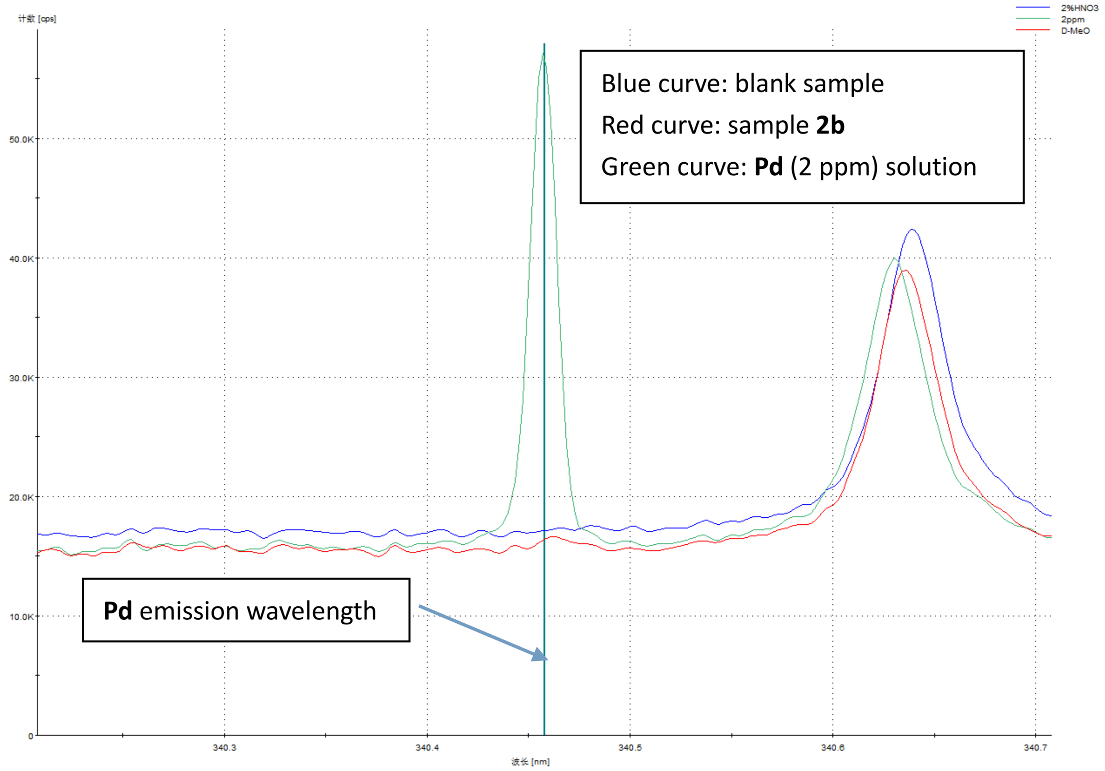


**Supplementary Figure 160(1). ICP-OES spectra of Pd in triphenyl(pyridin-2-yl)phosphonium iodide iodide 2j**

Pd

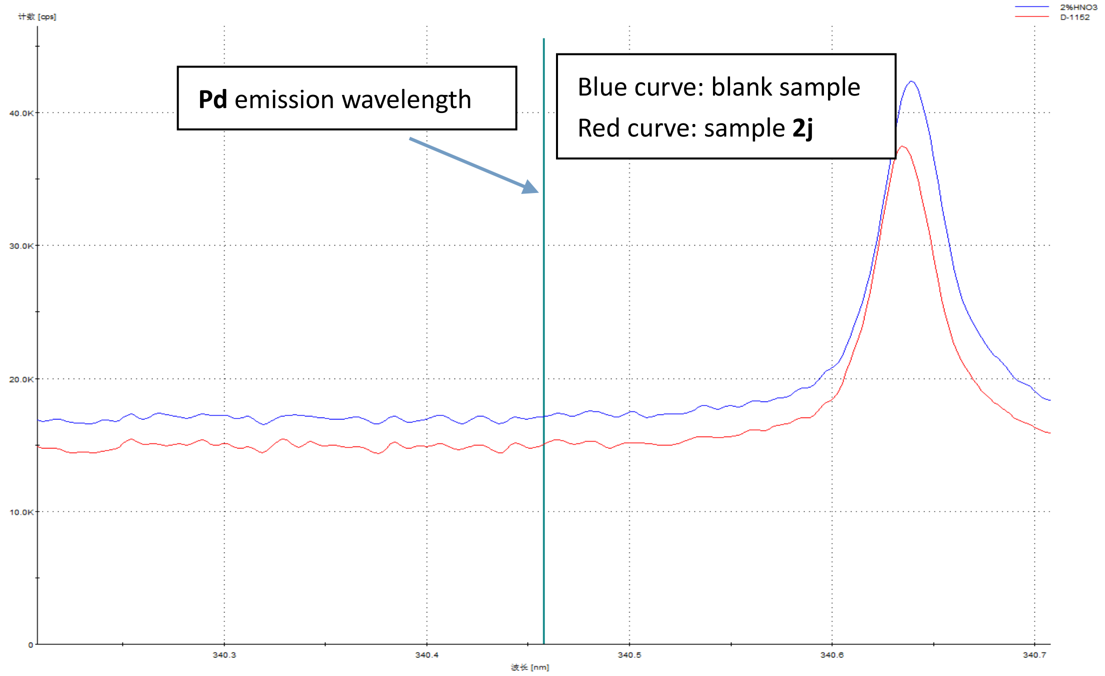

**Supplementary Figure 160(2). ICP-OES spectra of Pd in triphenyl(pyridin-2-yl)phosphonium iodide iodide 2j**

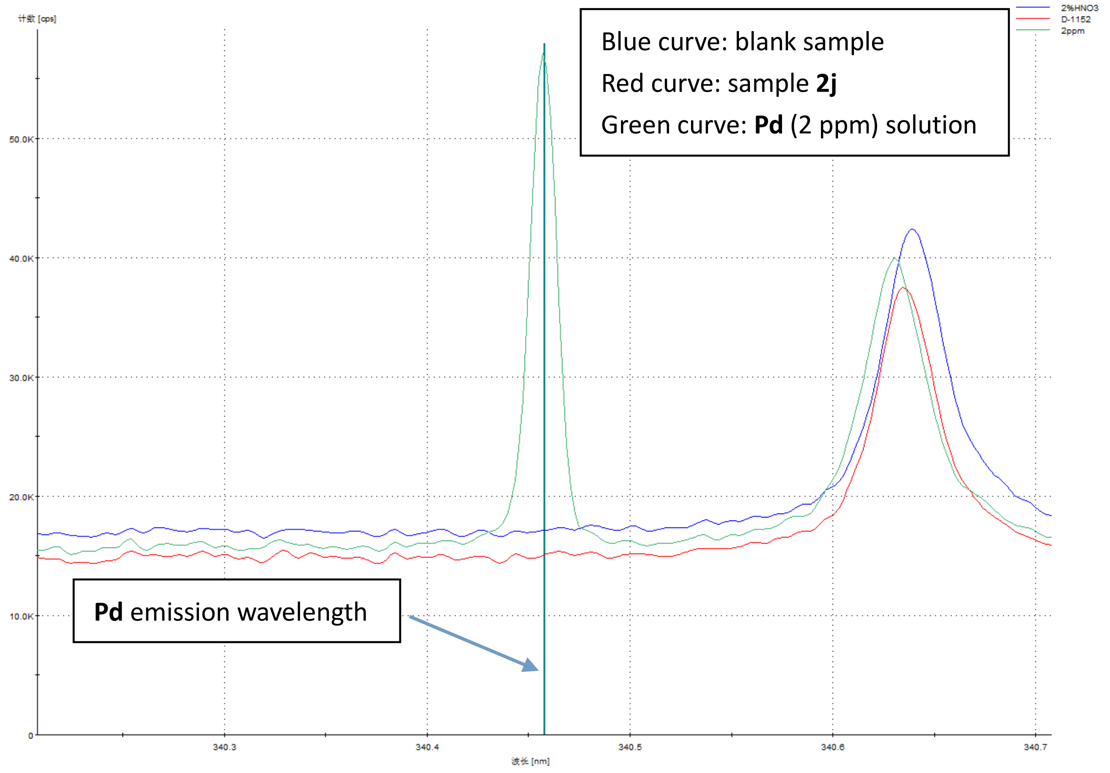


**Supplementary Figure 161(1). ICP-OES spectra of Pd in $Cs_2CO_3$**

**Pd**

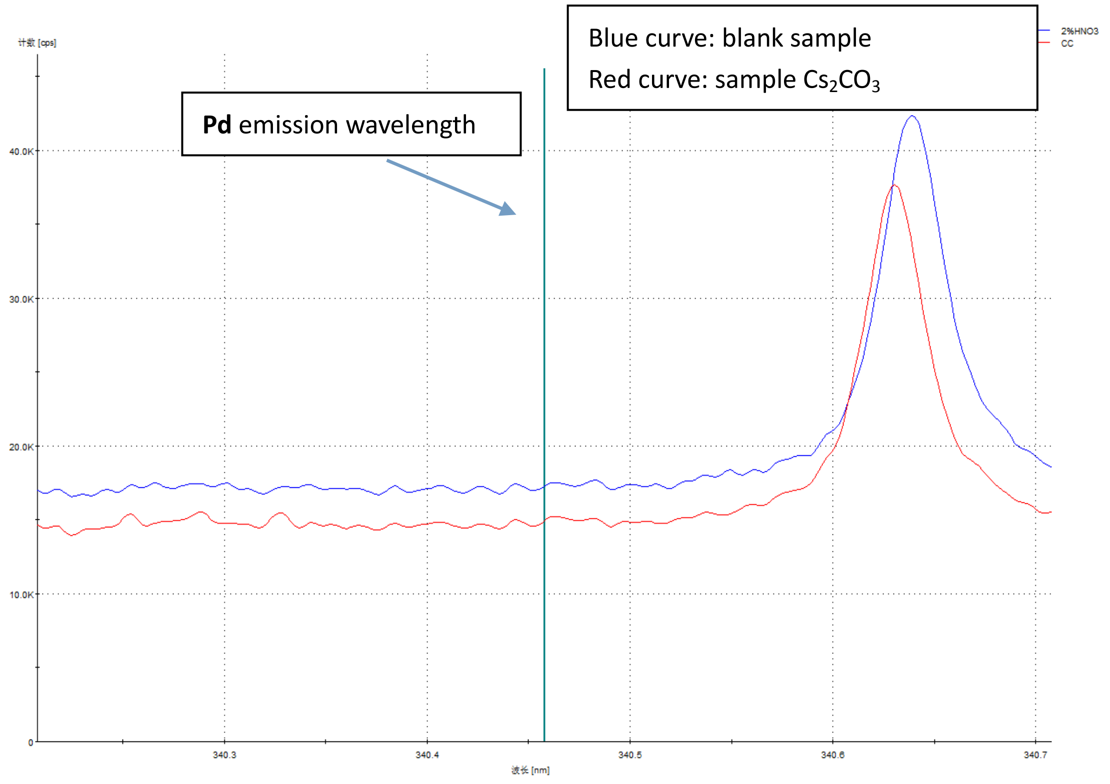

## Supplementary Figure 161(2). ICP-OES spectra of Pd in $Cs_2CO_3$

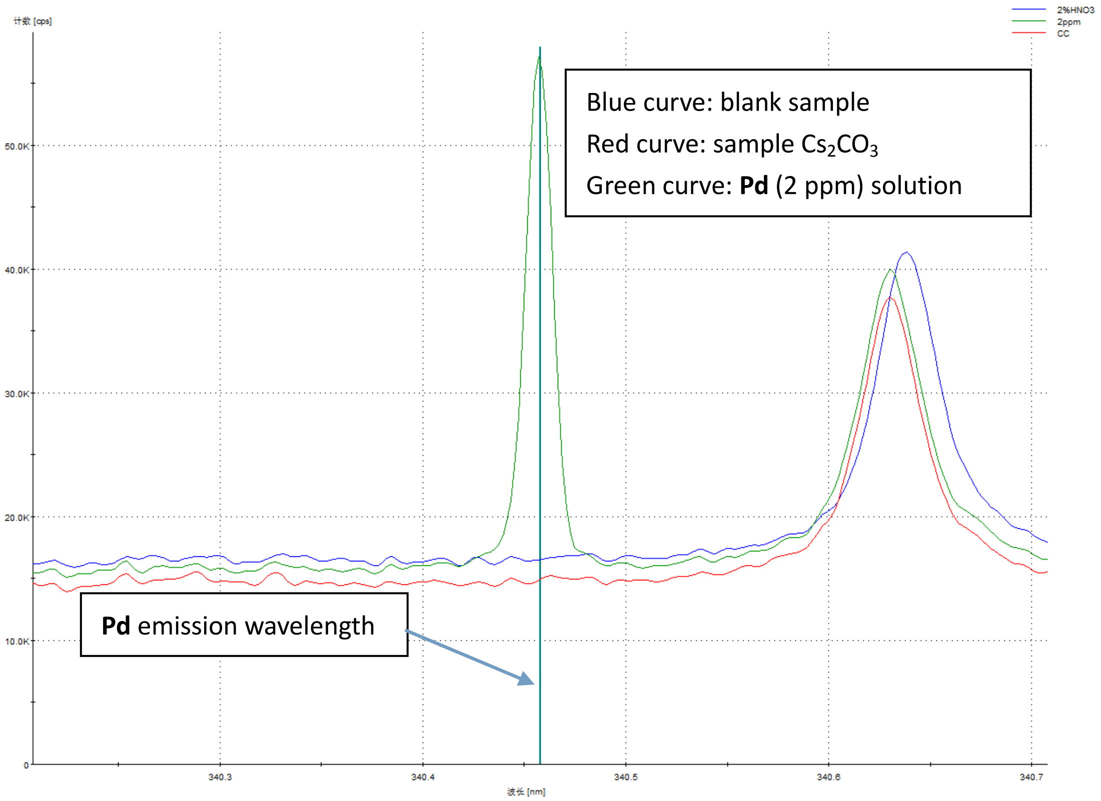


## Supplementary Figure 162(1). ICP-OES spectra of Fe in $Cs_2CO_3$

**Fe**

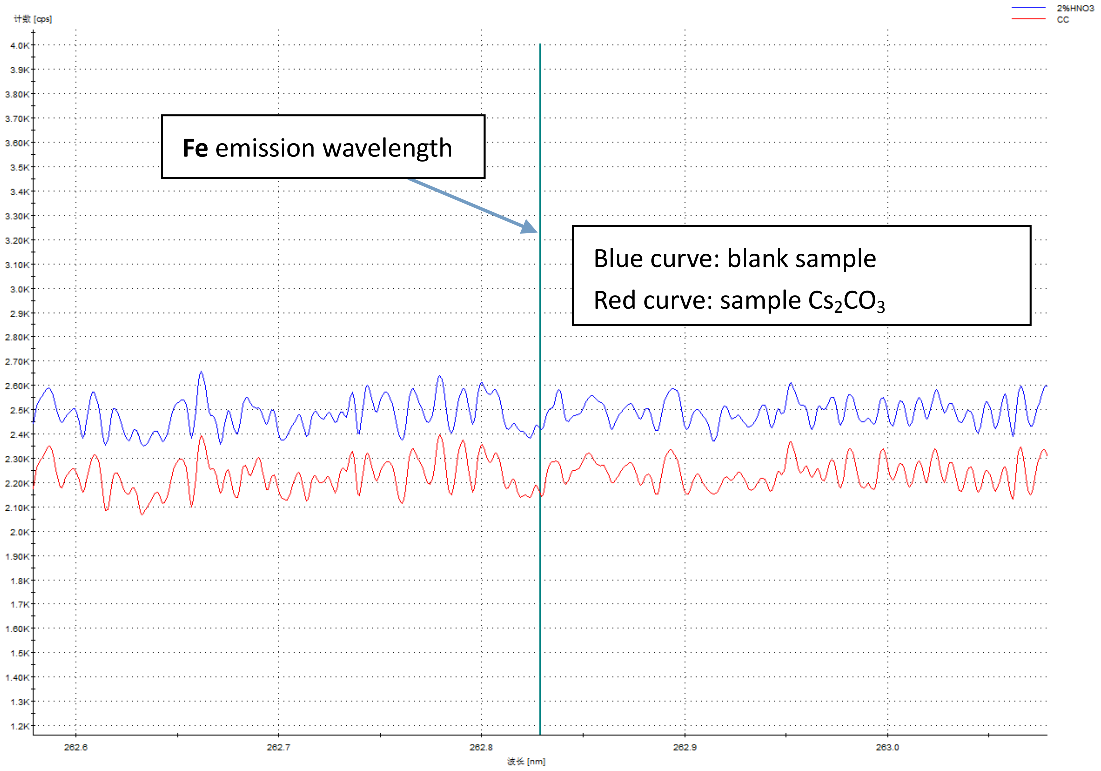

## Supplementary Figure 162(2). ICP-OES spectra of Fe in $Cs_2CO_3$

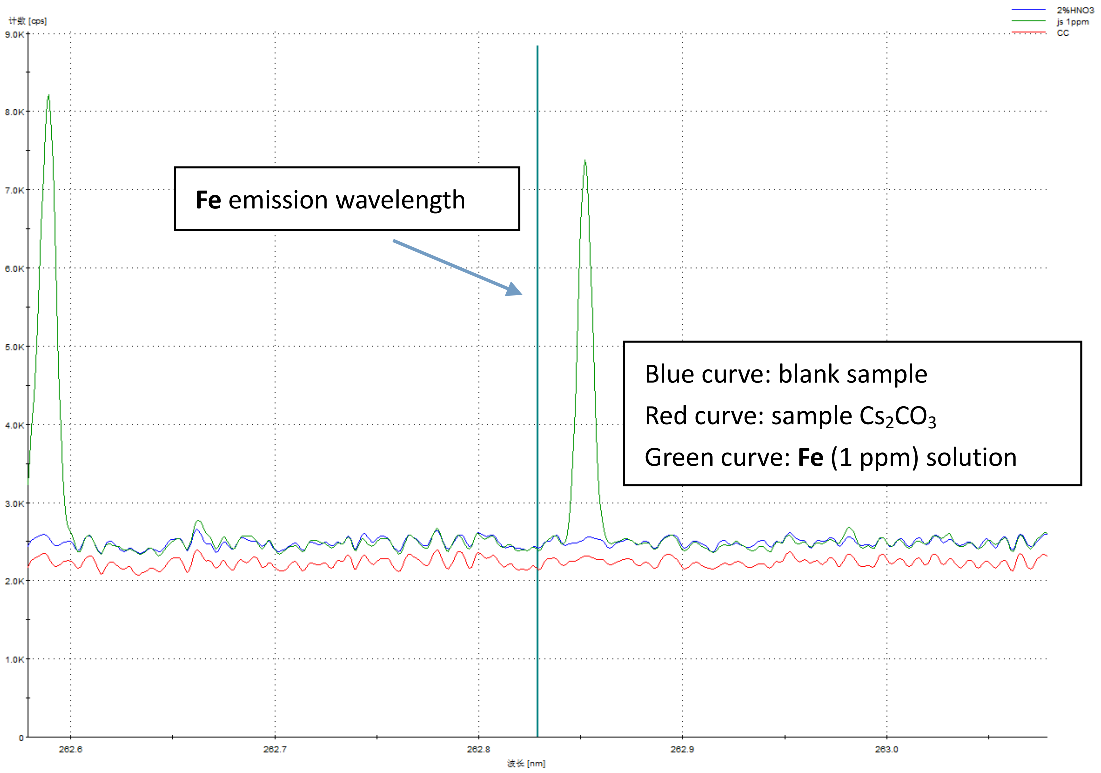


## Supplementary Figure 163(1). ICP-OES spectra of Co in $Cs_2CO_3$

**Co**

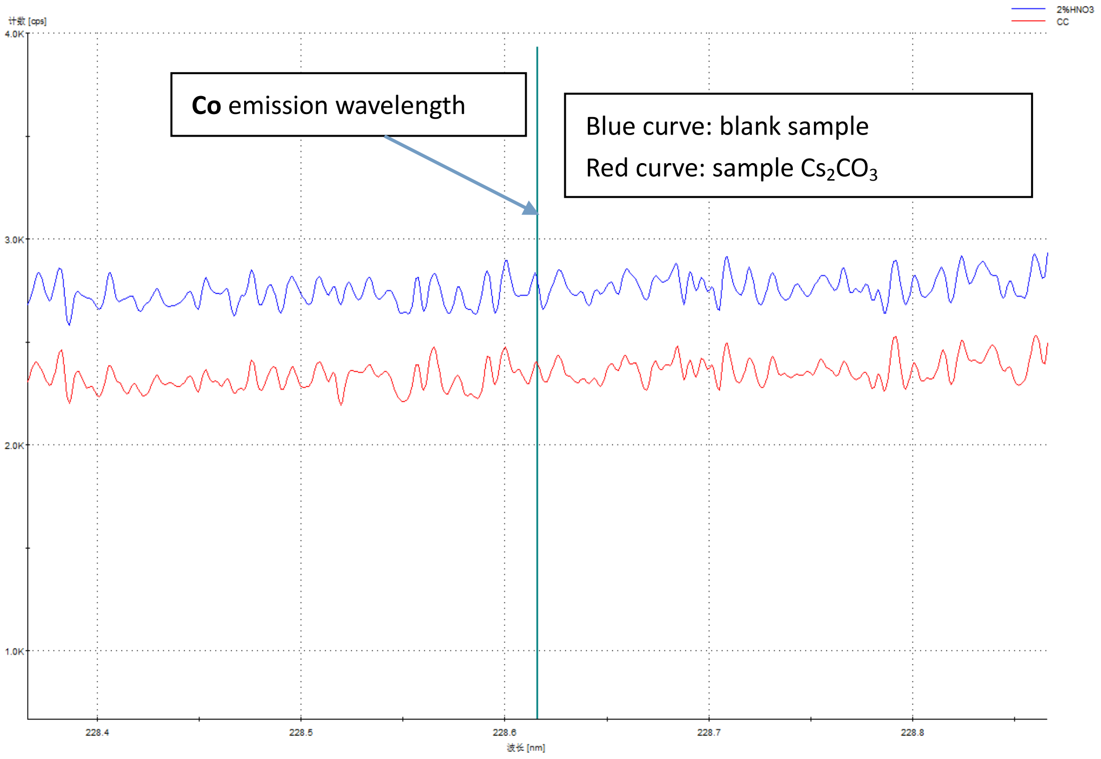

**Supplementary Figure 163(2). ICP-OES spectra of Co in $Cs_2CO_3$**

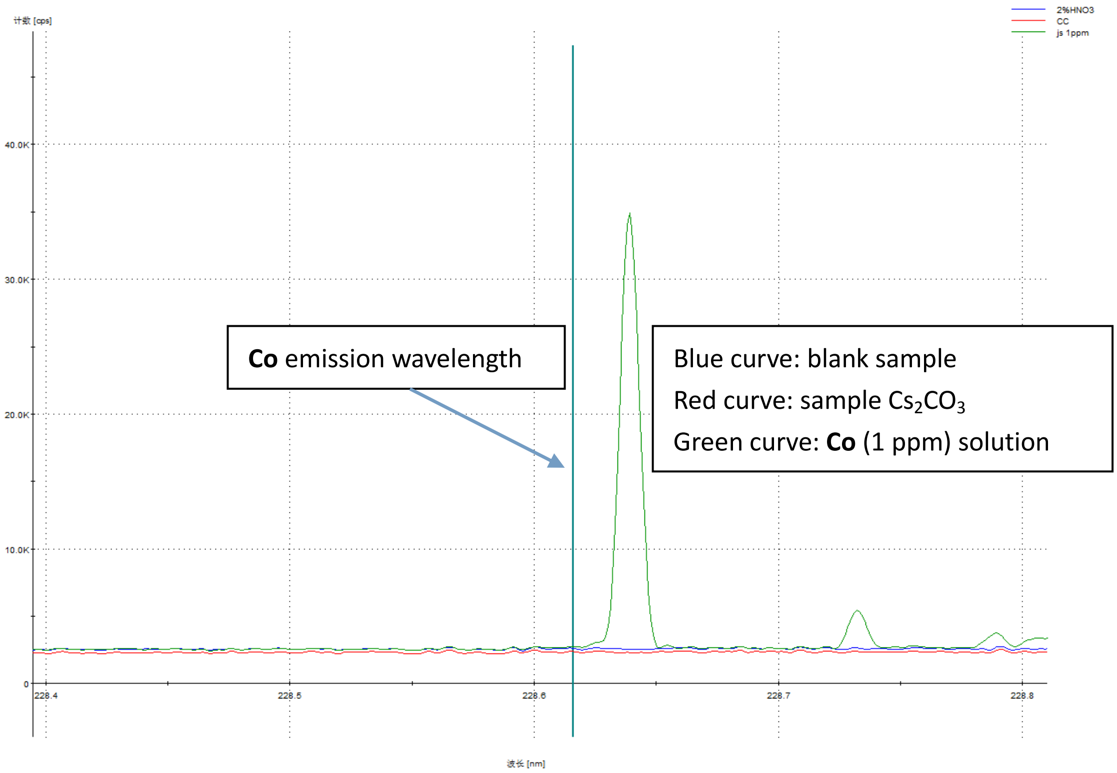


**Supplementary Figure 164(1). ICP-OES spectra of Ni in $Cs_2CO_3$**

**Ni**

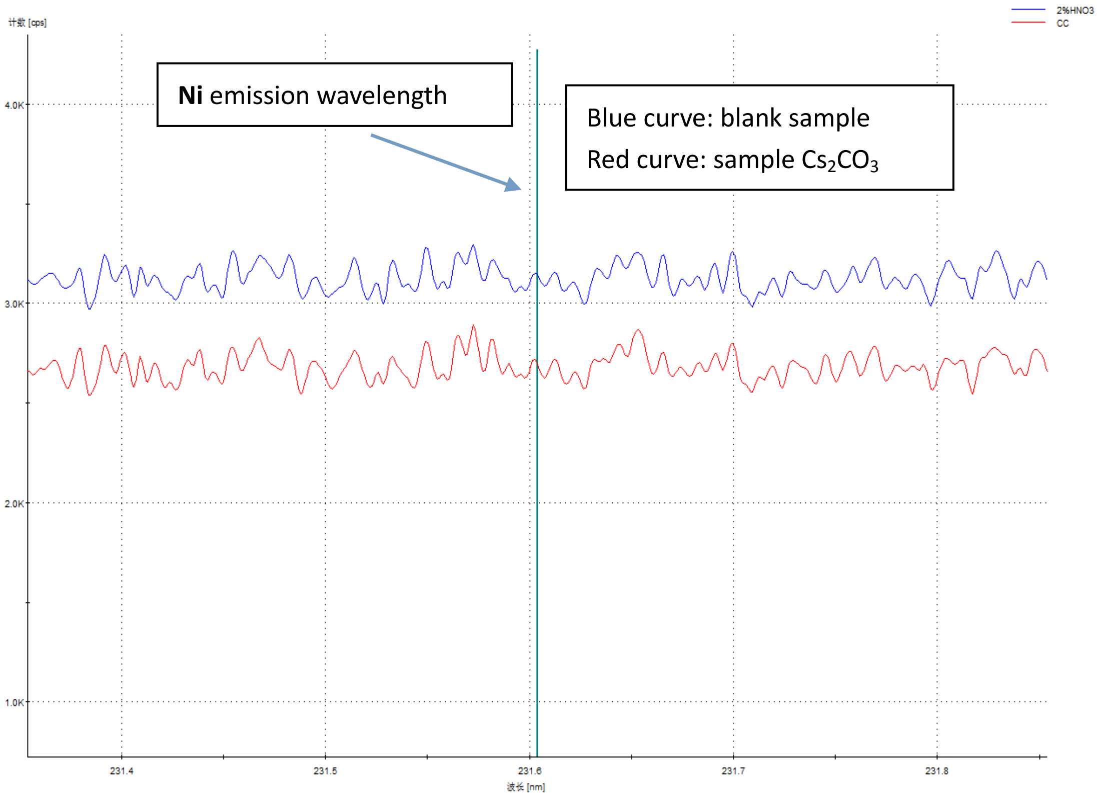

**Supplementary Figure 164(2). ICP-OES spectra of Ni in $Cs_2CO_3$**

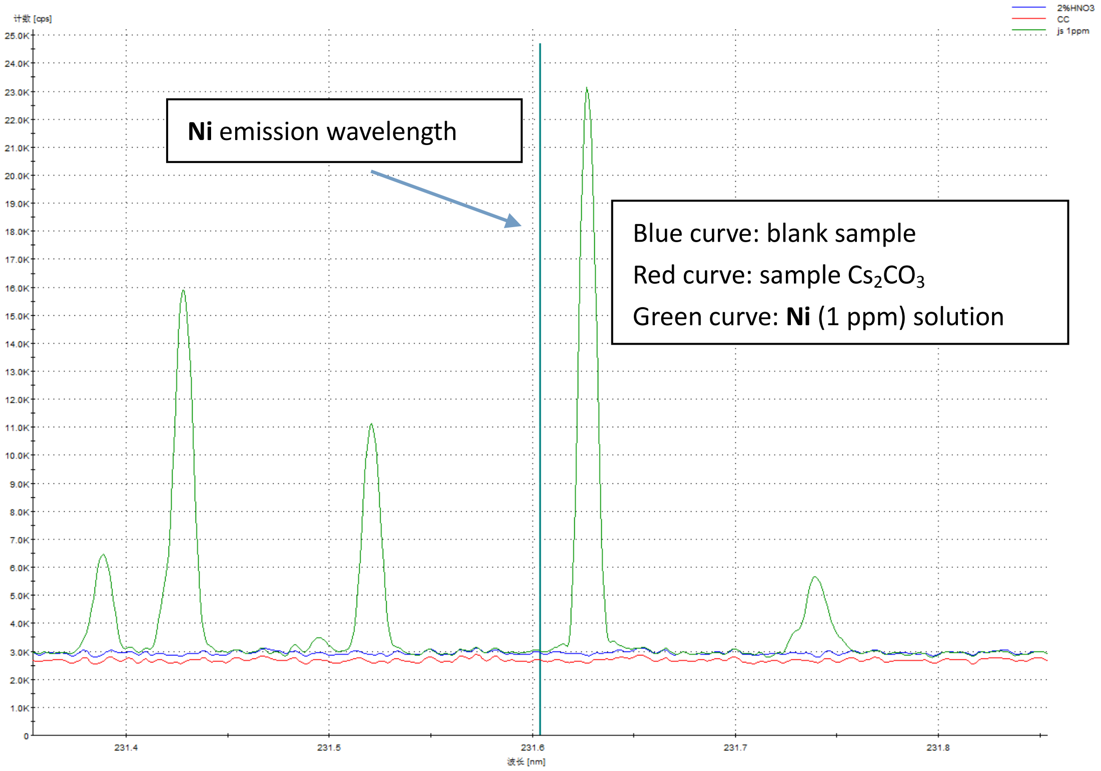


**Supplementary Figure 165(1). ICP-OES spectra of Cu in $Cs_2CO_3$**

**Cu**

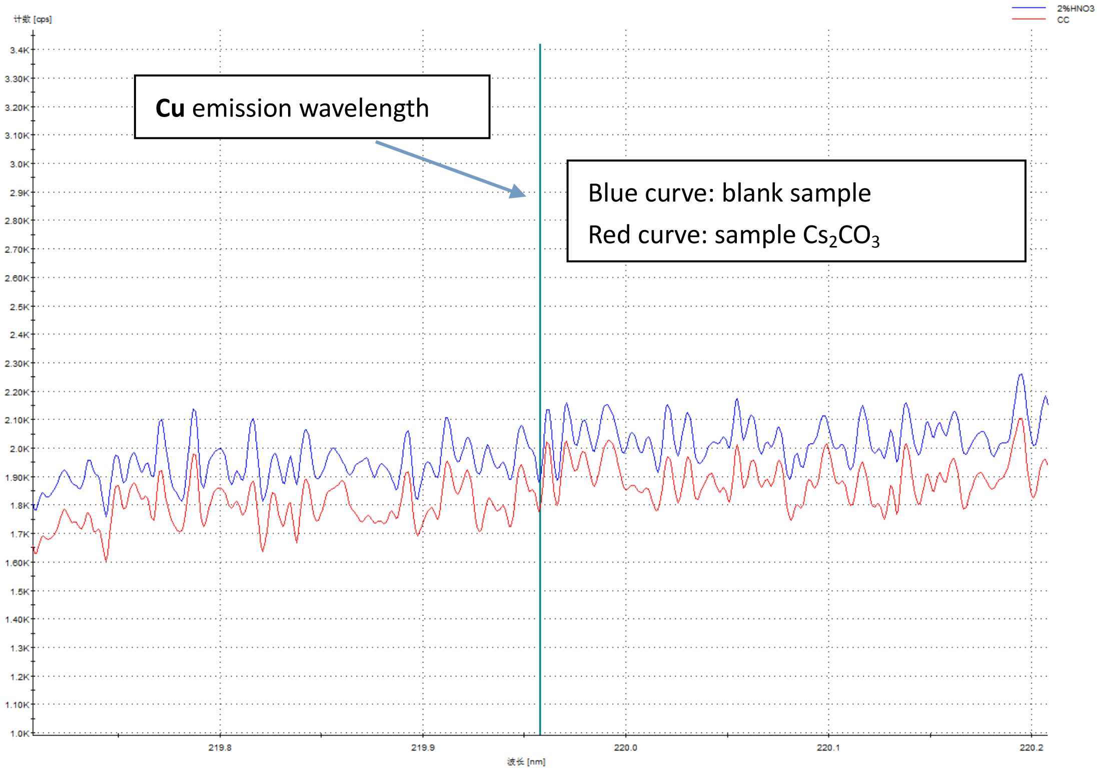

## Supplementary Figure 165(2). ICP-OES spectra of Cu in $Cs_2CO_3$

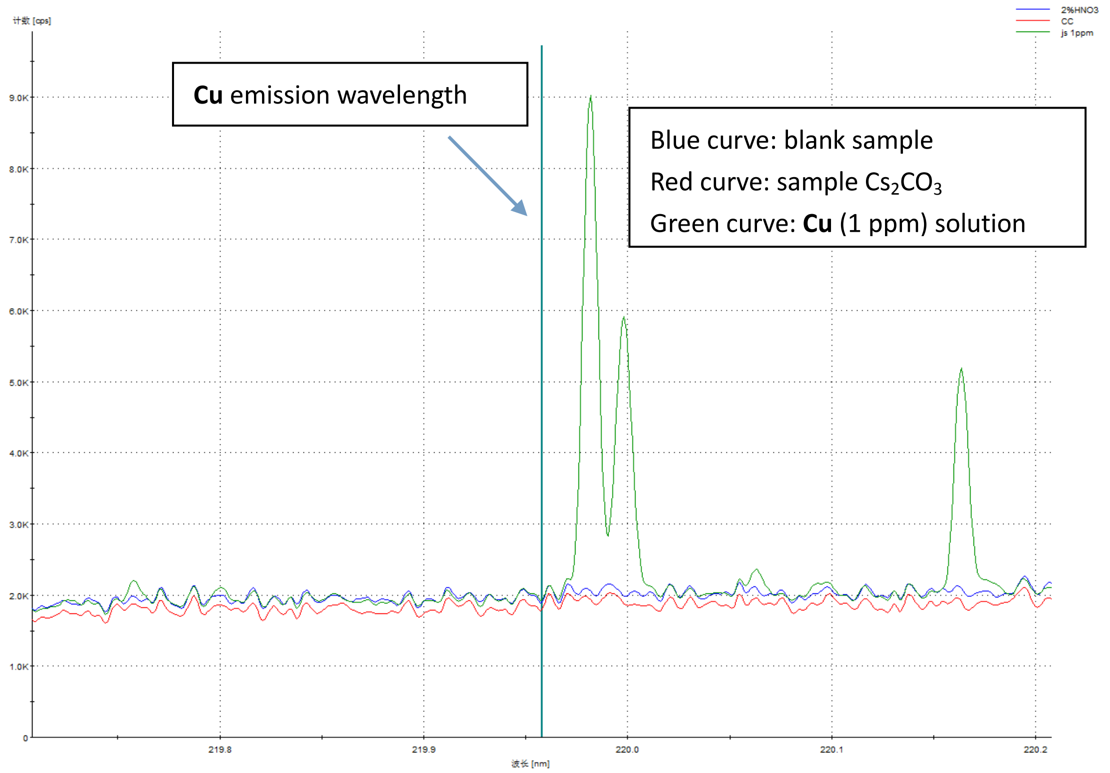


## Supplementary Figure 166(1). ICP-OES spectra of Zn in $Cs_2CO_3$

**Zn**

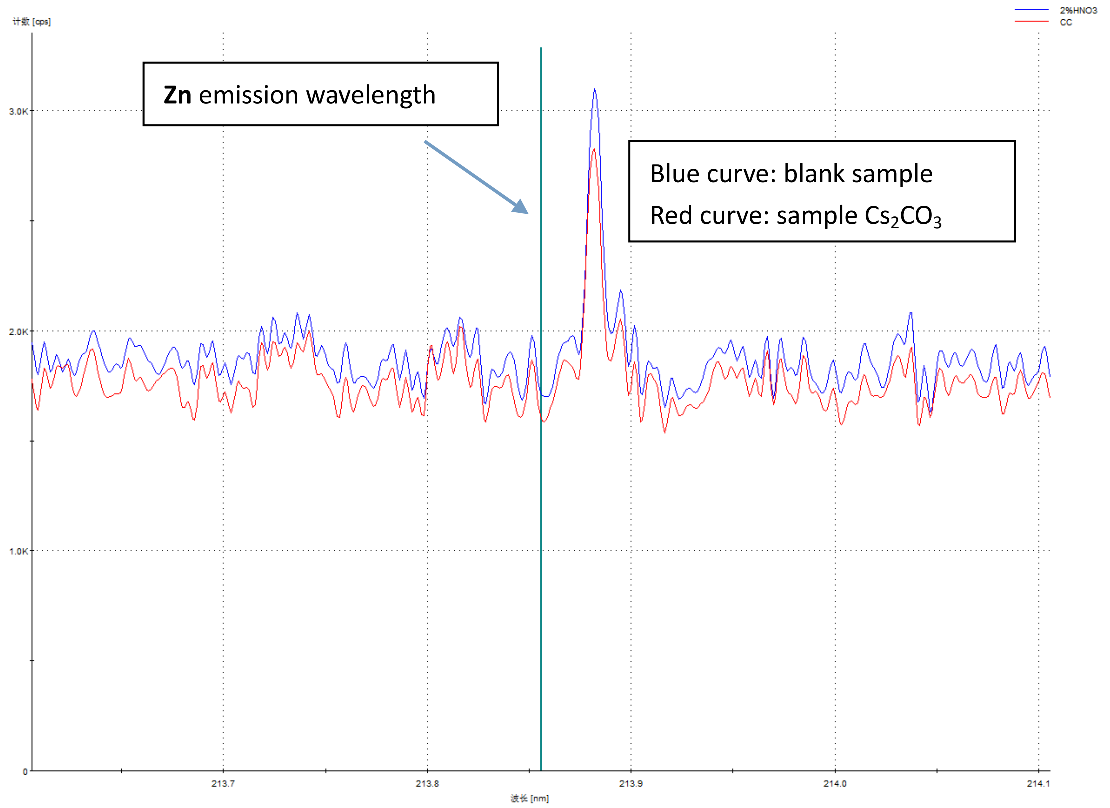

**Supplementary Figure 166(2). ICP-OES spectra of Zn in $Cs_2CO_3$**

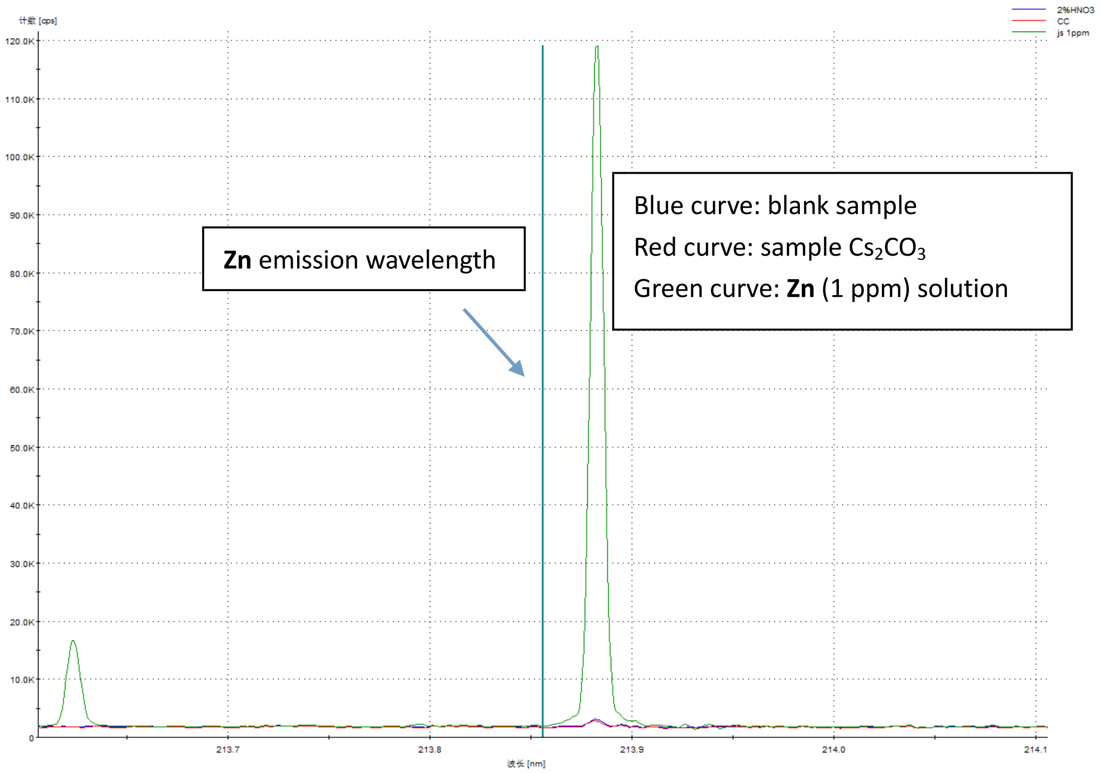


**Supplementary Figure 167. ICP-OES spectra of Ru in $Cs_2CO_3$**

**Ru**

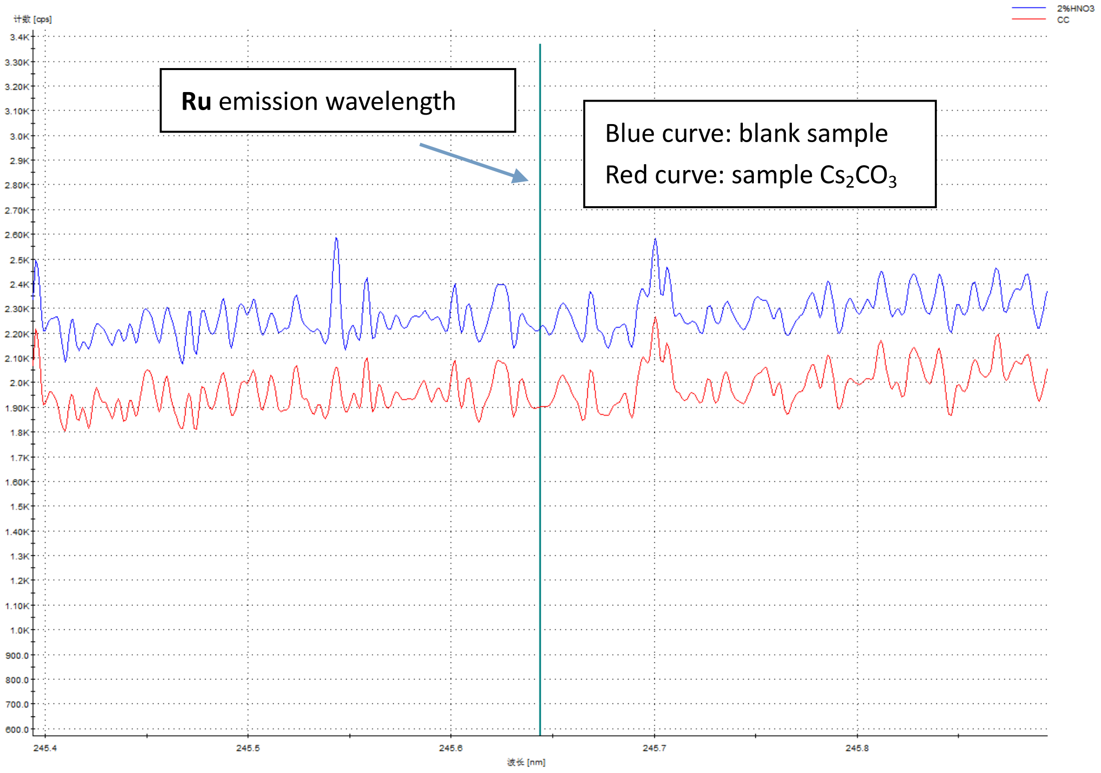

## Supplementary Figure 168. ICP-OES spectra of Rh in $Cs_2CO_3$

**Rh**

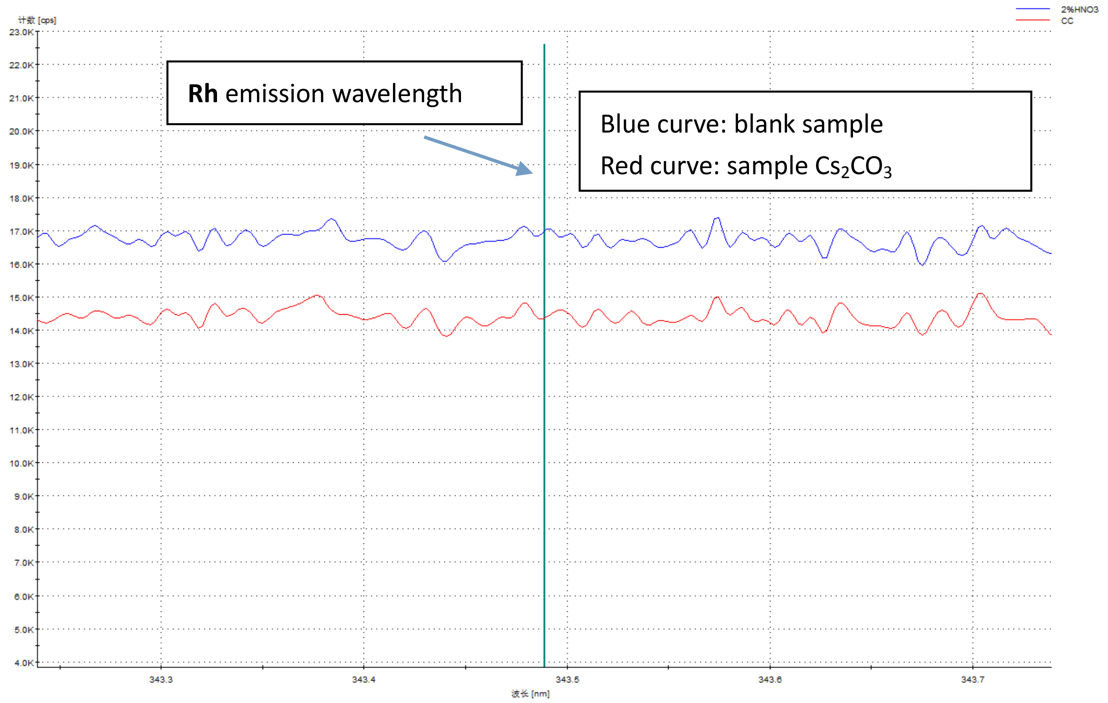


## Supplementary Figure 169. ICP-OES spectra of Ag in $Cs_2CO_3$

**Ag**

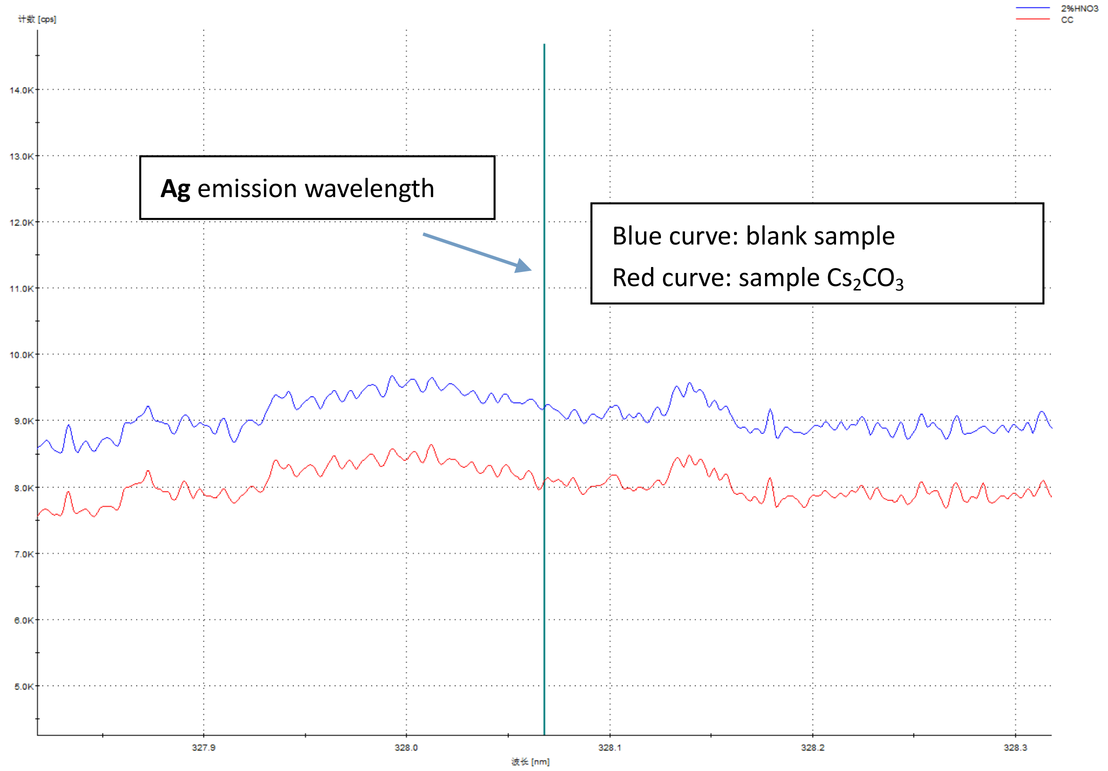

**Supplementary Figure 170 (1). ICP-OES spectra of Cd in $Cs_2CO_3$**

**Cd**

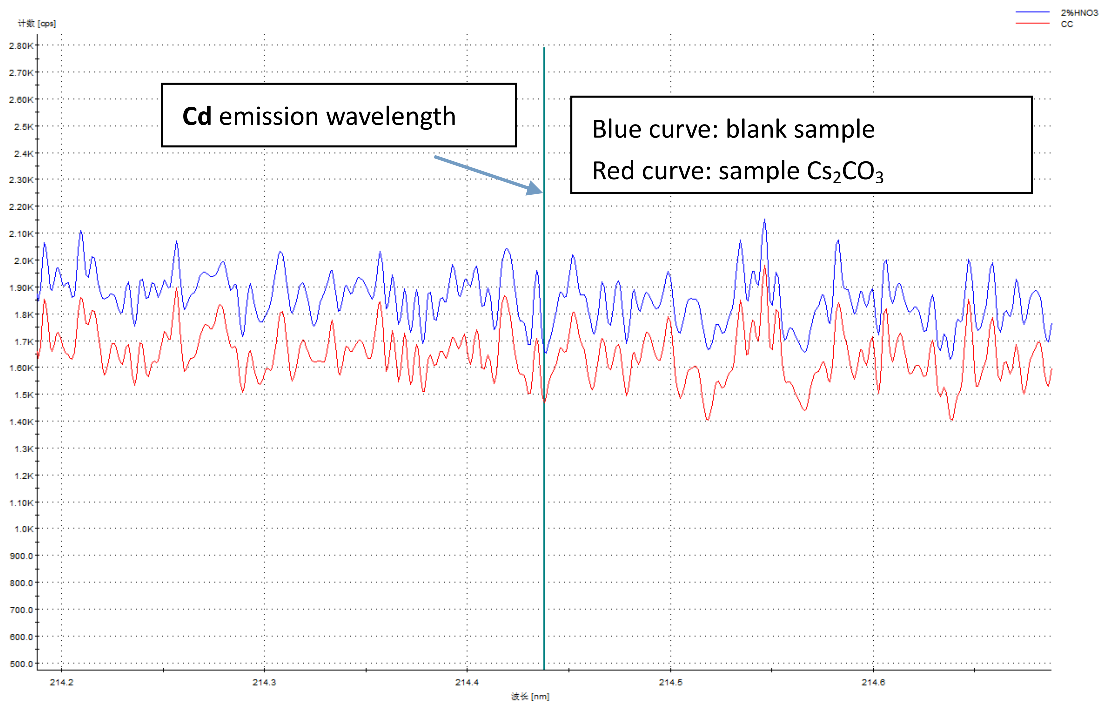


**Supplementary Figure 170(2). ICP-OES spectra of Cd in $Cs_2CO_3$**

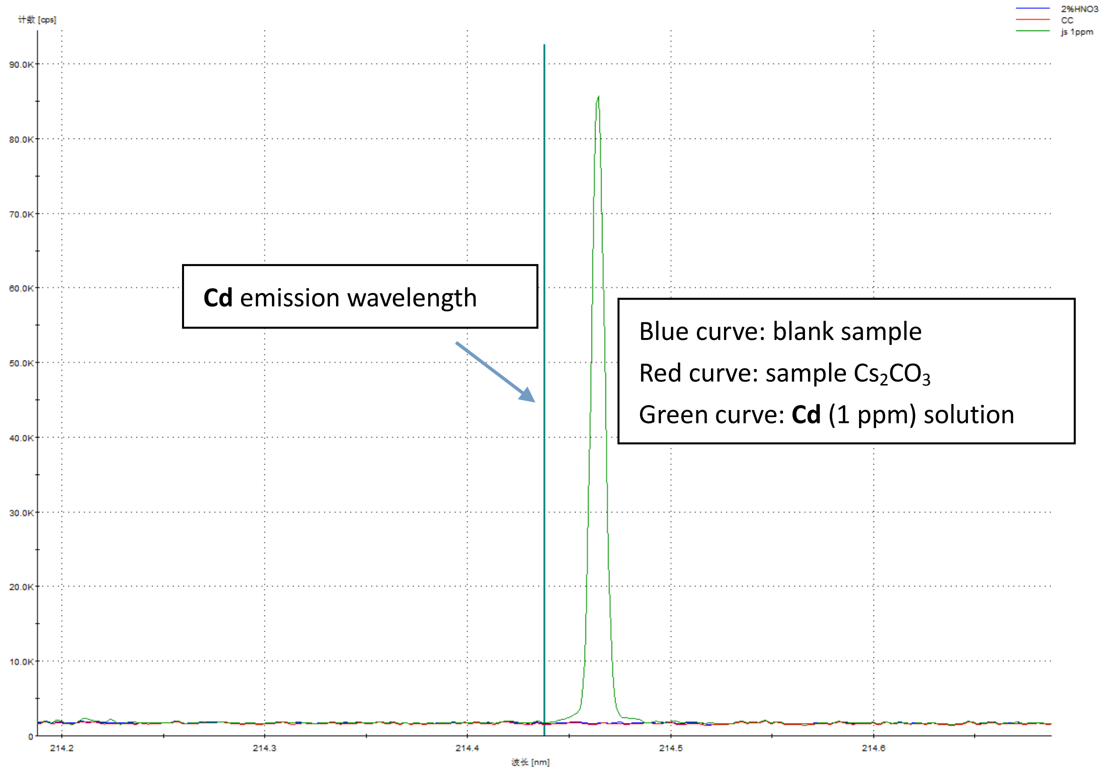

## Supplementary Figure 171. ICP-OES spectra of Os in $Cs_2CO_3$

**Os**

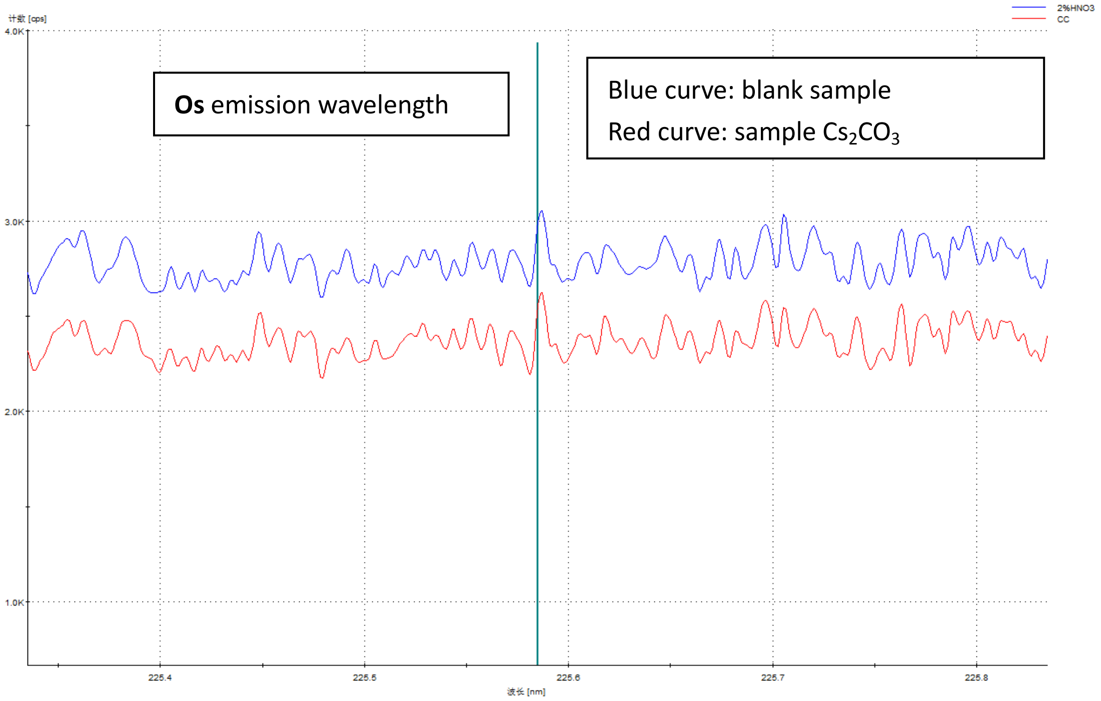


## Supplementary Figure 172. ICP-OES spectra of Ir in $Cs_2CO_3$

**Ir**

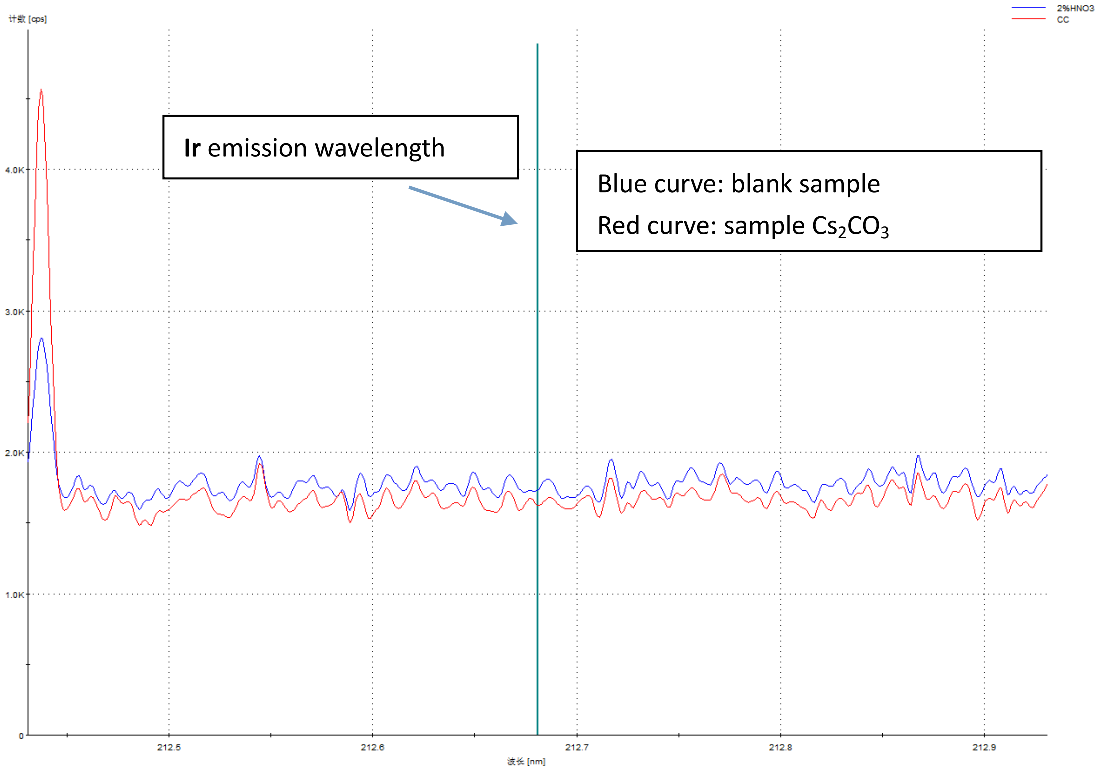

## Supplementary Figure 173. ICP-OES spectra of Pt in $Cs_2CO_3$

**Pt**

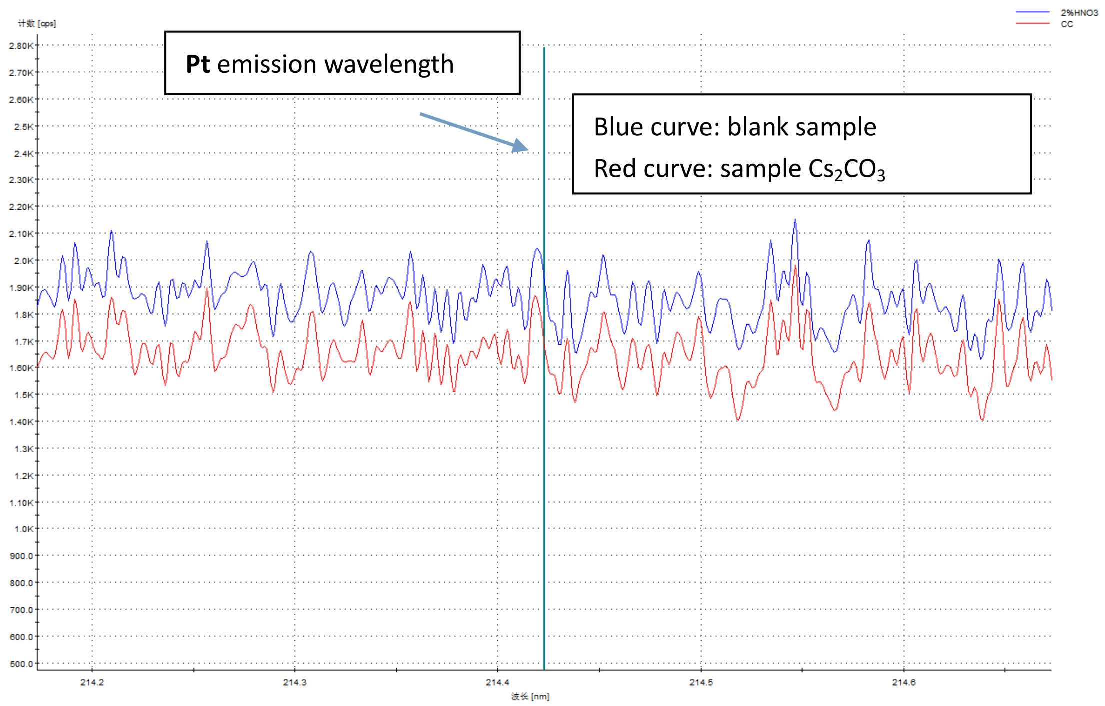


## Supplementary Figure 174. ICP-OES spectra of Au in $Cs_2CO_3$

**Au**

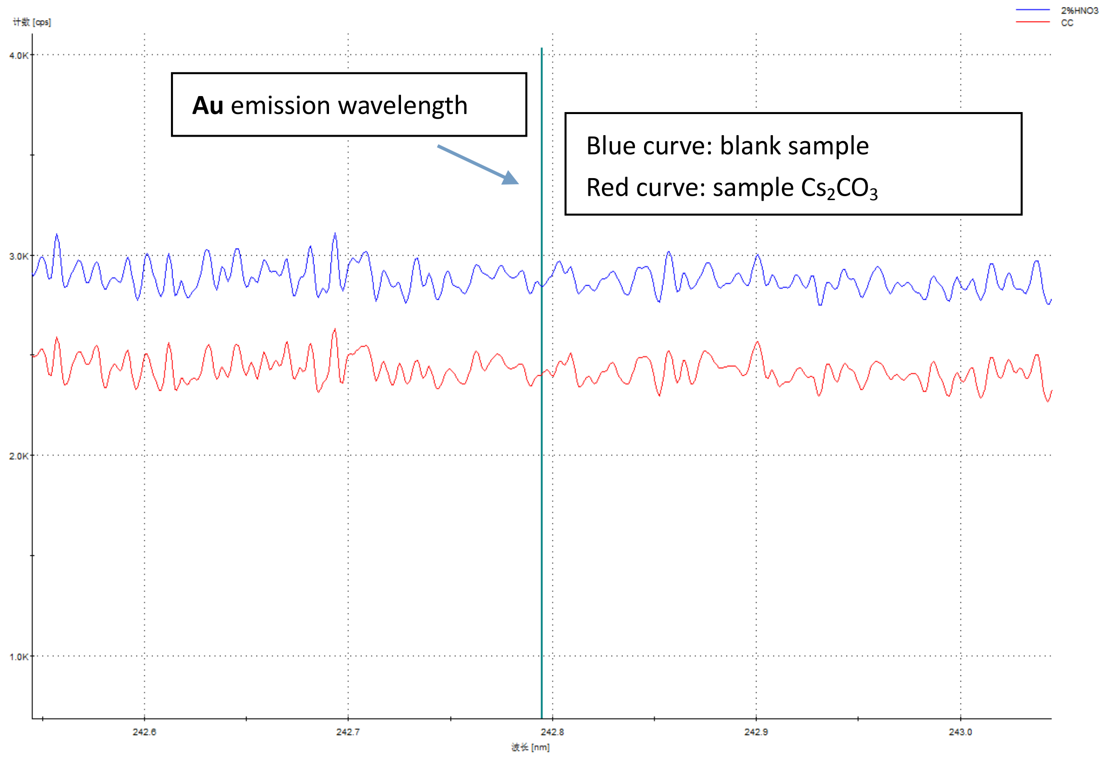

## Supplementary Figure 175. ICP-OES spectra of Hg in $Cs_2CO_3$

**Hg**

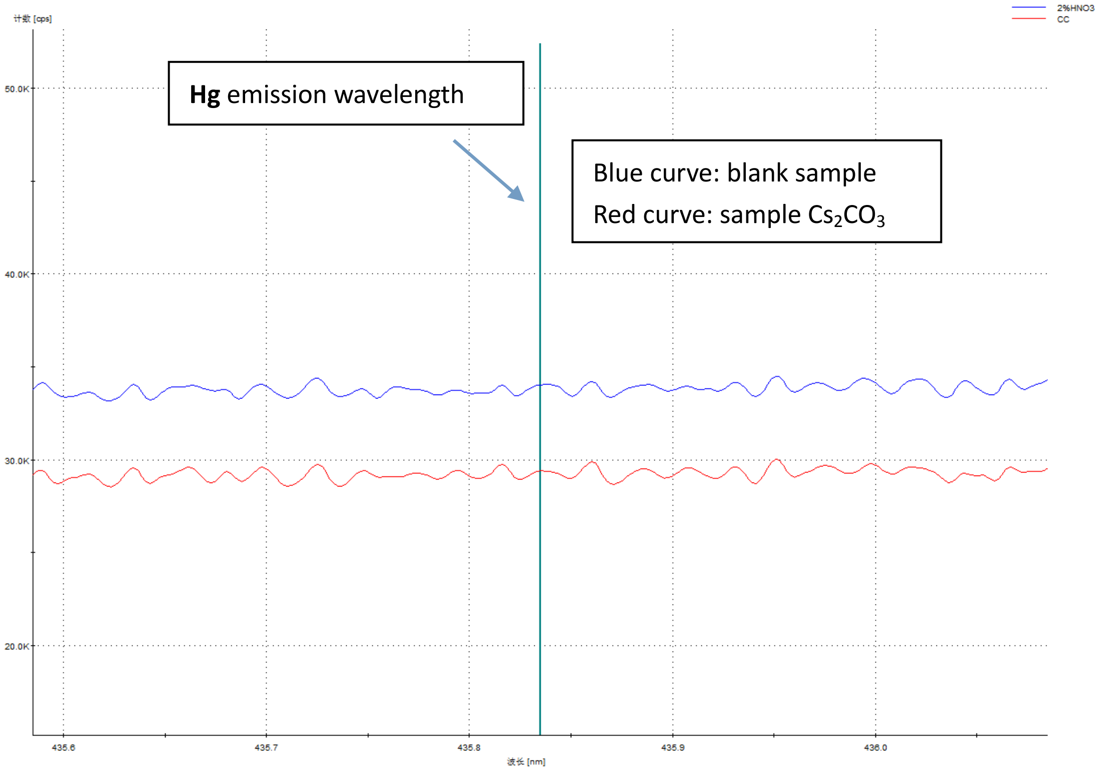

## Supplementary Table 1. Trace elemental analysis of reagents

Analyze samples for metals via ICP-OES. Samples reported as parts per million (ppm) ND = element was not detectable in sample.

| Metal | $Cs_2CO_3$ | Salt **2a** | Salt **2b** | Salt **2j** |
|---|---|---|---|---|
| Fe | ND | ND | | |
| Co | ND | ND | | |
| Ni | ND | ND | | |
| Cu | ND | ND | | |
| Zn | ND | ND | | |
| Ru | ND | ND | | |
| Rh | ND | ND | | |
| Pd | ND | ND | ND | ND |
| Ag | ND | ND | | |
| Cd | ND | ND | | |
| Os | ND | ND | | |
| Ir | ND | ND | | |
| Pt | ND | ND | | |
| Au | ND | ND | | |
| Hg | ND | ND | | |

## Supplementary Methods

## General Information

All solvents were obtained from commercial available and were extra dry grade. All glassware used was dried in a 120 ℃ oven and cooled in a desiccator before use. $^{1}H$, $^{19}F$, $^{31}P$ and $^{13}C$ NMR spectra were recorded on a 400 MHz NMR or 500 MHz NMR spectrometer. $^{1}H$ NMR chemical shifts were determined relative to internal $(CH_3)_4Si$ (TMS) at δ 0.0 or to the signal of a residual protonated solvent: $CDCl_3$ δ 7.26. $^{13}C$ NMR chemical shifts were determined relative to internal TMS at δ 0.0. $^{19}F$ spectra were referenced to $CFCl_3$.The following abbreviations were used to explain the multiplicities: s = singlet; d = doublet; t = triplet; q = quartet; m = multiplet; br = broad.

**Materials.** All reagents were used as received from commercial sources, unless specified otherwise, or prepared as described in the literature.

## Synthesis of the teraarylphosphonium salts[1]

## General procedure for the synthesis of teraarylphosphonium salts (2b-2i)[1]

Under argon atmosphere, the mixture of $Ar_3P$ (45 mmol, 1.5 equiv), Ar'I (30 mmol, 1.0 equiv) and $Pd_2(dba)_3$ (275 mg, 0.3 mmol, 1%) was stirred at 140 °C for 10 h. Then the mixture was cooled to room temperature. After filtration, the residue was washed with THF (20 mL × 3) and then dried under reduced pressure to afford the pure product as a solid.

## General procedure for the synthesis of teraarylphosphonium salts (2e'-2i')

Into the solution of (**2e-2i)** (10 mmol) in DCM (20 mL) was added the aqueous solution of lithium bis((trifluoromethyl)sulfonyl)amide (1 M, 20 mL). The resulting mixture was stirred for 1 min, and then extracted with DCM (20 mL × 3). The combined organic phase was concentrated to 20 mL. This anion metathesis procedure was further repeated twice. The final organic phase was dried over anhydrous $MgSO_4$. After filtration, the solvent was removed under vacumm to give the pure product (**2e'-2i')** as solid.

## General procedure for the synthesis of triphenyl(pyridin-2-yl)phosphonium iodide (2j)

Under argon atmosphere, the mixture of $Ph_3P$ (11.8g, 45 mmol, 1.5 equiv), 2-Iodopyridine (6.15g, 30 mmol, 1.0 equiv) was stirred at 140 °C for 10 h. Then the mixture was cooled to room temperature. After filtration, the residue was washed with THF (20 mL × 3) and then dried under reduced pressure to afford the pure product (**2j)** as white solid.

**Tetrakis(4-methoxyphenyl)phosphonium iodide (2b)**

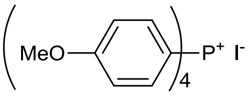


95% yield, gray solid, M.P. 217.6 °C. $^1$H NMR (400 MHz, $CDCl_3$) δ 7.47 (dd, *J* = 12.2, 8.9 Hz, 8H), 7.21 (dd, *J* = 8.9, 2.5 Hz, 8H), 3.93 (s, 12H). $^{31}$P NMR (162 MHz, $CDCl_3$) δ 20.46 (s, 1P). $^{13}$C NMR (101 MHz, $CDCl_3$) δ 164.64 (d, *J* = 2.9 Hz), 135.88 (d, *J* = 11.9 Hz), 116.26 (d, *J* = 14.0 Hz), 108.83 (d, *J* = 98.4 Hz), 56.37 (s). IR(KBr): 3010, 2972, 2938, 2836, 2565, 1910, 1589, 1567, 1500, 1462, 1436, 1411, 1291, 1263, 1183, 1108, 1014, 835, 820, 803, 717, 669, 620, 539, 518 $cm^{-1}$. HRMS (ESI): calcd. for $[C_{28}H_{28}O_4P]^+$ $[M - I]^+$ 459.1720, found 459.1707.

**Tetra-p-tolylphosphonium iodide (2c)**

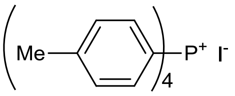


95% yield, gray solid, M.P. 272.3 °C. $^1$H NMR (400 MHz, $CDCl_3$) δ 7.52 (dd, *J* = 8.1, 3.0 Hz, 8H), 7.45 (dd, *J* = 12.6, 8.1 Hz, 8H), 2.50 (s, 12H). $^{31}$P NMR (162 MHz, $CDCl_3$) δ 22.07 (s, 1P). $^{13}$C NMR (126 MHz, $CDCl_3$) δ 146.73 (d, *J* = 5.0 Hz), 134.01 (d, *J* = 10.7 Hz), 131.26 (d, *J* = 13.0 Hz), 114.50 (d, *J* = 92.9 Hz), 21.84 (s). IR(KBr): 3014, 2913, 1594, 1560, 1496, 1443, 1398, 1312,

1188, 1106, 1039, 1011, 851, 803, 708, 662, 631, 518, 451 $cm^{-1}$. HRMS (ESI): calcd. for $[C_{28}H_{28}OP]^+$ $[M - I]^+$ 395.1923, found 395.1915.

**[1,1'-Biphenyl]-4-yltriphenylphosphonium iodide (2d)**[1]

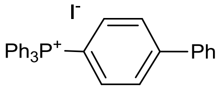


90% yield, white solid, M.P. $^1H$ NMR (400 MHz, $CDCl_3$) δ 7.96 (dd, *J* = 8.2, 3.1 Hz, 2H), 7.90 (tt, *J* = 7.6, 1.3 Hz, 3H), 7.80 (td, *J* = 7.6, 3.6 Hz, 6H), 7.76 – 7.61 (m, 10H), 7.49 – 7.37 (m, 3H). $^{31}P$ NMR (162 MHz, $CDCl_3$) δ 22.90 (s, 1P).

**(4-Cyanophenyl)triphenylphosphonium iodide (2e)**

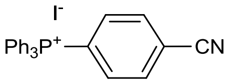


92% yield, yellow solid, M.P. 194.8 °C. $^1H$ NMR (400 MHz, $DCCl_3$) δ 8.08 (dd, *J* = 8.3, 2.8 Hz, 2H), 8.00 (dd, *J* = 12.5, 8.3 Hz, 2H), 7.90 (tt, *J* = 7.6, 1.3 Hz, 3H), 7.80 (td, *J* = 7.8, 3.7 Hz, 6H), 7.67 (dd, *J* = 13.1, 7.8 Hz, 6H). $^{31}P$ NMR (162 MHz, $DCCl_3$) δ 23.15 (s, 1P). $^{13}C$ NMR (126 MHz, $DCCl_3$) δ 136.09 (d, *J* = 3.0 Hz), 135.46 (d, *J* = 10.8 Hz), 134.50 (d, *J* = 10.5 Hz), 134.10 (d, *J* = 12.9 Hz), 131.10 (d, *J* = 13.1 Hz) 123.18 (d, *J* = 87.6 Hz), 118.52 (d, *J* = 3.4 Hz), 116.70 (d, *J* = 1.9 Hz), 116.04 (d, *J* = 89.6 Hz). IR(KBr): 3014, 2237, 1827, 1693, 1583, 1552, 1504, 1481, 1435, 1393, 1306, 1183, 1162, 1105, 1013, 996, 935, 849, 817, 753, 725, 691, 598, 618, 560, 528, 515, 497, 452 $cm^{-1}$. HRMS (ESI): calcd. for $[C_{25}H_{19}NP]^+$ $[M - I]^+$ 364.1250, found 364.1246.

**(3-Cyanophenyl)triphenylphosphonium iodide (2f)**

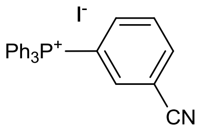


90% yield. white solid, M.P. 238.3 °C. $^1H$ NMR (400 MHz, $CDCl_3$) δ 8.09 (d, *J* = 7.4 Hz, 1H), 8.00 – 7.86 (m, 5H), 7.81 – 7.68 (m, 7H), 7.59 (dd, *J* = 13.2, 8.0 Hz, 6H). $^{31}P$ NMR (162 MHz, $CDCl_3$) δ 22.96 (s, 1P). $^{13}C$ NMR (101 MHz, $CDCl_3$) δ 138.82 (d, *J* = 9.6 Hz), 138.71 (d, *J* = 3.0 Hz), 136.66 (d, *J* = 11.7 Hz), 136.25 (d, *J* = 3.0 Hz), 134.47 (d, *J* = 10.6 Hz), 133.04 (d, *J* = 12.4 Hz), 131.19 (d, *J* = 13.0 Hz), 120.33 (d, *J* = 89.8 Hz), 116.54 (d, *J* = 1.0 Hz), 115.92 (d, *J* = 89.9 Hz), 114.83 (d, *J* = 14.9 Hz). IR(KBr): 3051, 2230, 1585, 1480, 1473, 1438, 1404, 1312, 1177, 1160, 1110, 1027, 993, 946, 852, 802, 755, 747, 727, 700, 688, 614, 562, 530, 523, 514 $cm^{-1}$. HRMS (ESI): calcd. for $[C_{25}H_{19}NP]^+$ $[M - NTf_2]^+$ 364.1250, found 364.1245.

**Triphenyl(4-(trifluoromethyl)phenyl)phosphonium iodide (2g)**

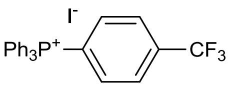


85% yield, white solid, M.P. 239.6 °C. $^1H$ NMR (400 MHz, $CDCl_3$) δ 8.06 – 7.88 (m, 7H), 7.81 (td, *J* = 8.0, 3.7 Hz, 6H), 7.68 (dd, *J* = 12.9, 8.0 Hz, 6H). $^{19}F$ NMR (376 MHz, $CDCl_3$) δ -63.55 (s,

3F). $^{31}$P NMR (162 MHz, $CDCl_3$) δ 23.00 (s, 1P). $^{13}$C NMR (101 MHz, $CDCl_3$) δ 136.27 (qd, *J* = 33.6, 3.1 Hz), 136.10 (d, *J* = 3.1 Hz), 135.40 (d, *J* = 10.8 Hz), 134.46 (d, *J* = 10.5 Hz), 131.11 (d, *J* = 13.0 Hz), 127.56 (dq, *J* = 13.2, 3.6 Hz), 122.74 (q, *J* = 274.1 Hz), 122.47 (d, *J* = 87.4 Hz), 116.30 (d, *J* = 89.6 Hz). IR(KBr): 3035, 3009, 2360, 1832, 1608, 1583, 1482, 1434, 1395, 1322, 1173, 1148, 1127, 1107, 1062, 1011, 996, 845, 764, 750, 736, 716, 689, 630, 615, 603, 553, 530, 491 $cm^{-1}$. HRMS (ESI): calcd. for $[C_{25}H_{19}F_3P]^+$ $[M - NTf_2]^+$ 407.1171, found 407.1166.

**(4-(Ethoxycarbonyl)phenyl)triphenylphosphonium iodide (2h)**

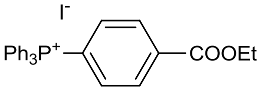


80% yield, white solid, M.P 207.0 $^{o}$C. $^{1}$H NMR (400 MHz, $CDCl_3$) δ 8.34 (dd, *J* = 8.5, 3.0 Hz, 2H), 7.90 (tt, *J* = 7.6, 1.3 Hz, 3H), 7.81 – 7.66 (m, 8H), 7.65 (dd, *J* = 13.1, 7.6 Hz, 6H), 4.40 (q, *J* = 7.1 Hz, 2H), 1.38 (t, *J* = 7.1 Hz, 3H). $^{31}$P NMR (162 MHz, $CDCl_3$) δ 23.05 (s, 1P). $^{13}$C NMR (101 MHz, $CDCl_3$) δ 164.30 (s), 136.19 (d, *J* = 2.7 Hz), 135.76 (d, *J* = 2.7 Hz), 134.47 (d, *J* = 10.7 Hz), 134.19 (d, *J* = 10.8 Hz), 130.86 (d, *J* = 13.2 Hz), 130.83 (d, *J* = 12.9 Hz), 122.30 (d, *J* = 87.7 Hz), 116.30 (d, *J* = 89.5 Hz), 61.86 (s), 14.11 (s). IR(KBr): 3016, 2980, 1733, 1584, 1480, 1435, 1393, 1375, 1308, 1266, 1178, 1161, 1108, 1089, 1008, 995, 761, 752, 723, 688, 629, 615, 560, 527, 448 $cm^{-1}$. HRMS (ESI): calcd. for $[C_{27}H_{24}O_2P]^+$ $[M - I]^+$ 411.1508, found 411.1507.

**(4-Acetylphenyl)triphenylphosphonium iodide (2i)[1]**

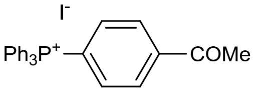


75% yield, white solid. $^{1}$H NMR (400 MHz, $CDCl_3$) δ 8.35 (dd, *J* = 8.4, 3.0 Hz, 2H), 7.93 – 7.82 (m, 5H), 7.78 (td, *J* = 7.8, 3.6 Hz, 6H), 7.65 (dd, *J* = 13.1, 7.8 Hz, 6H), 2.72 (s, 3H). $^{31}$P NMR (162 MHz, $CDCl_3$) δ 22.97 (s, 1P).

**(4-Cyanophenyl)triphenylphosphonium bis((trifluoromethyl)sulfonyl)amide (2e')**

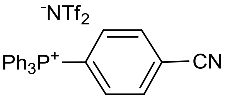


95% yield, white solid, M.P. 91.9 $^{o}$C. $^{1}$H NMR (400 MHz, $CDCl_3$) δ 7.98 (dd, *J* = 7.9, 2.5 Hz, 2H), 7.90 (t, *J* = 7.6 Hz, 3H), 7.83 – 7.73 (m, 8H), 7.61 (dd, *J* = 13.2, 8.1 Hz, 6H). $^{19}$F NMR (376 MHz, $CDCl_3$) δ -78.84 (s, 6F). $^{31}$P NMR (162 MHz, $CDCl_3$) δ 23.40 (s, 1P). $^{13}$C NMR (126 MHz, $CDCl_3$) δ 136.09 (d, *J* = 3.0 Hz), 135.13 (d, *J* = 10.7 Hz), 134.46 (d, *J* = 10.5 Hz), 133.76 (d, *J* = 13.0 Hz), 130.92 (d, *J* = 13.1 Hz), 123.65 (d, *J* = 87.6 Hz), 119.78 (q, *J* = 321.6 Hz), 118.87 (d, *J* = 3.4 Hz), 116.75 (d, *J* = 1.8 Hz), 116.13 (d, *J* = 89.7 Hz). IR(KBr): 3066, 2235,, 1504, 1485, 1440, 1394, 1352, 1227, 1183, 1136, 1108, 1057, 998, 833, 788, 751, 727, 690, 616, 598, 570, 559, 526, 516, 418 $cm^{-1}$. HRMS (ESI): calcd. for $[C_{25}H_{19}NP]^+$ $[M - NTf_2]^+$ 364.1250, found 364.1245.

**(3-Cyanophenyl)triphenylphosphonium bis((trifluoromethyl)sulfonyl)amide (2f')**

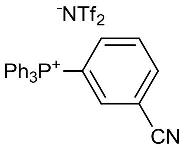


98% yield, white solid, M.P. 137.6 °C. $^1$H NMR (400 MHz, $CDCl_3$) δ 8.11 (d, *J* = 7.6 Hz, 1H), 8.05 – 7.97 (m, 2H), 7.91 (t, *J* = 7.6 Hz, 3H), 7.78 (td, *J* = 7.8, 3.7 Hz, 6H), 7.72 (d, *J* = 12.8 Hz, 1H), 7.61 (dd, *J* = 13.2, 7.8 Hz, 6H). $^{19}$F NMR (376 MHz, $CDCl_3$) δ -78.84 (s, 3F). $^{31}$P NMR (162 MHz, $CDCl_3$) δ 23.22 (s, 1P). $^{13}$C NMR (101 MHz, $CDCl_3$) δ 138.59 (d, *J* = 3.0 Hz), 138.43 (d, *J* = 9.7 Hz), 136.88 (d, *J* = 11.7 Hz), 136.20 (d, *J* = 3.1 Hz), 134.42 (d, *J* = 10.5 Hz), 132.10 (d, *J* = 12.6 Hz), 131.00 (d, *J* = 13.1 Hz), 119.75 (q, *J* = 321.7 Hz), 120.66 (d, *J* = 89.9 Hz), 116.54 (d, *J* = 1.4 Hz), 116.14 (d, *J* = 89.9 Hz), 115.04 (d, *J* = 15.0 Hz). IR(KBr): 3079, 2239, 1587, 1485, 1476, 1443, 1408, 1353, 1232, 1182, 1139, 1110, 1063, 996, 899, 850, 809, 793, 753, 727, 701, 687, 614, 570, 532, 515 cm$^{-1}$. HRMS (ESI): calcd. for $[C_{25}H_{19}NP]^+$ $[M - NTf_2]^+$ 364.1250, found 364.1246.

**Triphenyl(4-(trifluoromethyl)phenyl)phosphonium bis((trifluoromethyl)sulfonyl)amide (2g')**

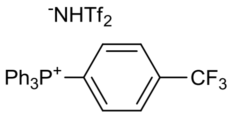


96% yield, white solid, M.P. 87.0 °C. $^1$H NMR (400 MHz, $CDCl_3$) δ 7.97 (dd, *J* = 8.2, 2.4 Hz, 2H), 7.90 (t, *J* = 7.4 Hz, 3H), 7.86 – 7.72 (m, 8H), 7.62 (dd, *J* = 13.2, 8.1 Hz, 6H). $^{19}$F NMR (376 MHz, $CDCl_3$) δ -63.76 (s, 3F), -78.85 (s, 6F). $^{31}$P NMR (162 MHz, $CDCl_3$) δ 23.23 (s, 1P). $^{13}$C NMR (101 MHz, $CDCl_3$) δ 136.29 (qd, *J* = 33.6, 3.2 Hz), 136.01 (d, *J* = 3.1 Hz), 135.18 (d, *J* = 10.8 Hz), 134.39 (d, *J* = 10.4 Hz), 130.87 (d, *J* = 13.1 Hz), 127.35 (dq, *J* = 13.2, 3.7 Hz), 122.83 (q, *J* = 274.8 Hz), 122.68 (d, *J* = 87.2 Hz), 119.78 (q, *J* = 322.6 Hz), 116.50 (d, *J* = 89.7 Hz).IR(KBr): 3101, 3058, 1610, 1589, 1486, 1439, 1401, 1355, 1324, 1185, 1135, 1110, 1062, 1015, 998, 837, 788, 762, 728, 715, 691, 613, 599, 570, 552, 527, 512, 464 cm$^{-1}$. HRMS (ESI): calcd. for $[C_{25}H_{19}F_3P]^+$ $[M - NTf_2]^+$ 407.1171, found 407.1167.

**(4-(Ethoxycarbonyl)phenyl)triphenylphosphonium bis((trifluoromethyl)sulfonyl)amide (2h')**

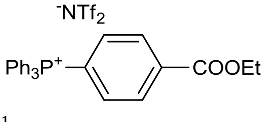


98% yield, white solid, M.P. 74.2 °C. $^1$H NMR (400 MHz, $CDCl_3$) δ 8.33 (dd, *J* = 8.5, 3.2 Hz, 2H), 7.89 (tt, *J* = 7.5, 1.3 Hz, 3H), 7.81 – 7.66 (m, 8H), 7.60 (dd, *J* = 13.2, 7.8 Hz, 6H), 4.42 (q, *J* = 7.1 Hz, 2H), 1.39 (t, *J* = 7.1 Hz, 3H). $^{19}$F NMR (376 MHz, $CDCl_3$) δ -78.81 (s, 6F). $^{31}$P NMR (162 MHz, $CDCl_3$) δ 23.24 (s, 1P). $^{13}$C NMR (101 MHz, $CDCl_3$) δ 164.54 (d, *J* = 1.2 Hz), 136.69 (d, *J* = 3.0 Hz), 135.95 (d, *J* = 2.8 Hz), 134.54 (d, *J* = 10.6 Hz), 134.36 (d, *J* = 10.4 Hz), 131.04 (d, *J* = 13.0 Hz), 130.83 (d, *J* = 13.0 Hz), 122.46 (d, *J* = 87.8 Hz), 119.81 (q, *J* = 321.9 Hz), 116.75 (d, *J* = 89.6 Hz), 62.08 (s), 14.07 (s). IR(KBr): 3016, 2979, 1718, 1588, 1486, 1439, 1398, 1354, 1287, 1228, 1200, 1137, 1109, 1056, 997, 788, 761, 725, 690, 653, 612, 570, 561, 528, 513 cm$^{-1}$. HRMS (ESI): calcd. for $[C_{27}H_{24}O_2P]^+$ $[M – NTf_2]^+$ 411.1508, found 411.1504.

**(4-Acetylphenyl)triphenylphosphonium Bis((trifluoromethyl)sulfonyl)amide (2i')**

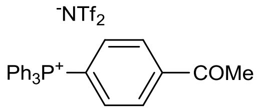


96% yield, white solid, M.P. 93.7 °C. $^1$H NMR (400 MHz, $CDCl_3$) δ 8.25 (dd, *J* = 8.3, 2.8 Hz, 2H), 7.88 (t, *J* = 7.8 Hz, 3H), 7.78 – 7.70 (m, 8H), 7.60 (dd, *J* = 13.1, 7.8 Hz, 6H), 2.66 (s, 3H). $^{31}$P NMR (162 MHz, $CDCl_3$) δ 23.13 (s, 1P). $^{19}$F NMR (376 MHz, $CDCl_3$) δ -78.82 (s, 6F). $^{13}$C NMR (126 MHz, $CDCl_3$) δ 196.98 (s), 141.91 (d, *J* = 3.0 Hz), 135.90 (d, *J* = 3.0 Hz), 134.92 (d, *J* = 10.6 Hz), 134.38 (d, *J* = 10.4 Hz), 130.81 (d, *J* = 13.0 Hz), 129.76 (d, *J* = 13.1 Hz), 122.33 (d, *J* = 87.9 Hz), 119.81 (q, *J* = 321.9 Hz), 116.86 (d, *J* = 89.6 Hz), 26.90 (s). IR(KBr): 3093, 3001, 1695, 1587, 1560, 1486, 1439, 1397, 1358, 1263, 1224, 1200, 1140, 1109, 1057, 997, 961, 821, 786, 759, 746, 739, 728, 694, 655, 626, 611, 891, 569, 552, 526, 512 $cm^{-1}$. HRMS (ESI): calcd. for $[C_{26}H_{22}OP]^+$ $[M - NTf_2]^+$ 381.1403, found 381.1401.

**Triphenyl(pyridin-2-yl)phosphonium iodide (2j)[2]**

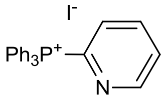


**2j**

90% yield, white solid, M.P. $^1$H NMR (400 MHz, $CDCl_3$) δ 8.99 (d, *J* = 4.4 Hz, 1H), 8.32 (dd, *J* = 13.3, 7.7 Hz, 1H), 8.02 (t, *J* = 7.0 Hz, 1H), 7.88 (t, *J* = 7.4 Hz, 3H), 7.84 – 7.58 (m, 13H). $^{31}$P NMR (162 MHz, $CDCl_3$) δ 14.92 (s, 1P).

## General procedure for phenylation of aldehydes

Under $N_2$ atmosphere, the mixture of aldehyde (0.50 mmol), tetraphenylphosphonium iodide (583.0 mg, 1.25 mmol) and $Cs_2CO_3$ (488.7mg, 1.50 mmol) in THF (4 mL) and stirred at 65 $^oC$ for 12 h. The reaction was quenched by 3 N HCl (0.5 mL). The resulting mixture was extracted with DCM (3 × 30 mL). The combined organic phase was dried over $Na_2SO_4$. After filtration, the solvent was removed by concentration, and the residue was subjected to column chromatography to afford the pure product.

**[1,1'-Biphenyl]-4-yl(phenyl)methanol (3aa)[3]**

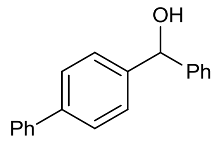


90% yield. white solid. $^1$H NMR (400 MHz, $CDCl_3$) δ 7.59 – 7.46 (m, 4H), 7.47 – 7.38 (m, 6H), 7.37 – 7.26 (m, 4H), 5.89 (d, *J* = 3.3 Hz, 1H), 2.23 (d, *J* = 3.3 Hz, 1H).

**(4-(Dimethylamino)phenyl)(phenyl)methanol (3ba)[4]**

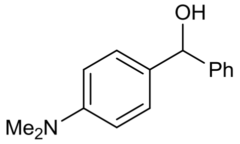


69% yield, colorless oil. $^1$H NMR (400 MHz, $CDCl_3$) δ 7.38 (d, *J* = 7.5 Hz, 2H), 7.32 (t, *J* = 7.5 Hz, 2H), 7.26 – 7.18 (m, 3H), 6.69 (d, *J* = 8.0 Hz, 2H), 5.76 (s, 1H), 2.92 (s, 6H).

**(4-Methoxyphenyl)(phenyl)methanol (3ca)[3]**

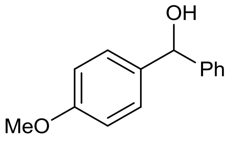


89% yield, colorless oil. $^1$H NMR (400 MHz, $CDCl_3$) δ 7.43 – 7.19 (m, 7H), 6.86 (d, *J* = 8.2 Hz, 2H), 5.81 (s, 1H), 3.78 (s, 3H), 2.16 (s, 1H).

**(2-Methoxyphenyl)(phenyl)methanol (3da)[3]**

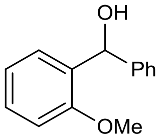


80% yield, colorless oil. $^1$H NMR (400 MHz, $CDCl_3$) δ 7.38 (d, *J* = 7.5 Hz, 2H), 7.32 (t, *J* = 7.5 Hz, 2H), 7.28 – 7.23 (m, 2H), 7.22 (d, *J* = 7.4 Hz, 1H), 6.94 (t, *J* = 7.5 Hz, 1H), 6.88 (d, *J* = 8.2 Hz, 1H), 6.05 (s, 1H), 3.81 (s, 3H), 3.03 (s, 1H).

**Mesityl(phenyl)methanol (3ea)**[5]

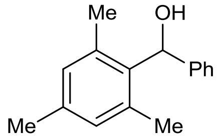


86% yield, white solid. $^1$H NMR (400 MHz, $CDCl_3$) δ 7.34 – 7.17 (m, 5H), 6.86 (s, 2H), 6.32 (d, *J* = 3.7 Hz, 1H), 2.26 (s, 3H), 2.23 (s, 6H), 2.10 (d, *J* = 3.7 Hz, 1H).

**(4-Fluorophenyl)(phenyl)methanol (3fa)**[3]

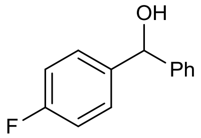


94% yield, colorless oil. $^1$H NMR (400 MHz, $CDCl_3$) δ 7.35 – 7.32 (m, 6H), 7.30 – 7.26 (m, 1H), 7.01 (t, *J* = 8.7 Hz, 2H), 5.83 (d, *J* = 3.4 Hz, 1H), 2.18 (d, *J* = 3.4 Hz, 1H). $^{19}$F NMR (376 MHz, colorless oil.) δ -114.01 – -116.53 (m, 1F).

**(4-Bromophenyl)(phenyl)methanol (3ga)**[3]

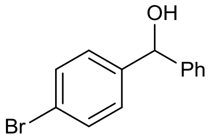


69% yield, white solid. $^1$H NMR (400 MHz, $CDCl_3$) δ 7.44 (d, *J* = 8.4 Hz, 2H), 7.34 – 7.31 (m, 4H), 7.30 – 7.22 (m, 3H), 5.78 (s, 1H), 2.21 (s, 1H).

**(3-Bromophenyl)(phenyl)methanol (3ha)**[6]

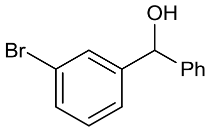


62% yield, colorless. $^1$H NMR (400 MHz, $CDCl_3$) δ 7.55 (s, 1H), 7.37 (d, *J* = 8.4 Hz, 1H), 7.35 – 7.31 (m, 4H), 7.31 – 7.25 (m, 2H), 7.18 (t, *J* = 7.8 Hz, 1H), 5.78 (s, 1H), 2.04 (s, 1H).

**Phenyl(4-(trifluoromethyl)phenyl)methanol (3ia)**[4]

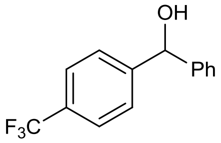


86% yield, white solid. $^1$H NMR (400 MHz, $CDCl_3$) δ 7.58 (d, *J* = 8.1 Hz, 2H), 7.50 (d, *J* = 8.1 Hz, 2H), 7.36 – 7.32 (m, 4H), 7.32 – 7.25 (m, 1H), 5.87 (s, 1H), 2.08 (s, 1H). $^{19}$F NMR (376 MHz, $CDCl_3$) δ -62.52 (s, 3F).

**Phenyl(3-(trifluoromethyl)phenyl)methanol (3ja)[7]**

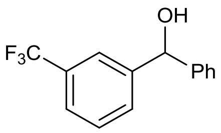


84% yield, colorless oil. $^{1}$H NMR (400 MHz, $CDCl_3$) δ 7.69 (s, 1H), 7.52 (t, *J* = 7.8 Hz, 2H), 7.43 (t, *J* = 7.7 Hz, 1H), 7.36 – 7.33 (m, 4H), 7.32 – 7.25 (m, 1H), 5.87 (s, 1H), 2.00 (s, 1H). $^{19}$F NMR (376 MHz, $CDCl_3$) δ -62.58 (s, 3F).

**Phenyl(2-(trifluoromethyl)phenyl)methanol (3ka)[7]**

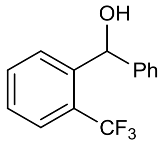


88% yield, colorless oil. $^{1}$H NMR (400 MHz, $CDCl_3$) δ 7.68 – 7.60 (m, 2H), 7.53 (t, *J* = 7.6 Hz, 1H), 7.42 – 7.29 (m, 5H), 7.26 (d, *J* = 6.9 Hz, 1H), 6.30 (s, 1H), 2.18 (s, 1H).

**3-(Hydroxy(phenyl)methyl)benzonitrile (3la)[8]**

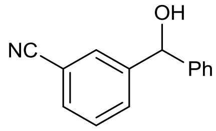


73% yield, yellow solid. $^{1}$H NMR (400 MHz, $CDCl_3$) δ 7.69 (s, 1H), 7.60 (d, *J* = 7.9 Hz, 1H), 7.52 (d, *J* = 7.7 Hz, 1H), 7.41 (t, *J* = 7.8 Hz, 1H), 7.38 – 7.26 (m, 5H), 5.83 (s, 1H), 2.08 (s, 1H).

**4-(Hydroxy(phenyl)methyl)benzonitrile (3ma)[9]**

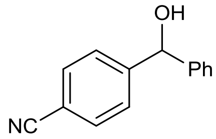


68% yield, yellow solid. $^{1}$H NMR (400 MHz, $CDCl_3$) δ 7.60 (d, *J* = 8.2 Hz, 2H), 7.49 (d, *J* = 8.2 Hz, 2H), 7.39 – 7.25 (m, 5H), 5.85 (s, 1H), 2.32 (br, 1H).

**Methyl 4-(hydroxy(phenyl)methyl)benzoate (3na)[3]**

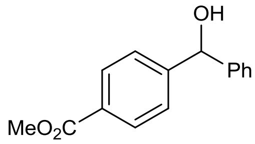


75% yield, colorless oil. $^{1}$H NMR (400 MHz, $CDCl_3$) δ 7.98 (d, *J* = 8.2 Hz, 2H), 7.45 (d, *J* = 8.2 Hz, 2H), 7.41 – 7.25 (m, 5H), 5.87 (s, 1H), 3.88 (s, 3H), 1.94 (br, 1H).

**[1,1'-Biphenyl]-2-yl(phenyl)methanol (3oa)[10]**

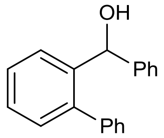


90% yield, white solid. $^{1}H$ NMR (400 MHz, $CDCl_3$) δ 7.55 (d, *J* = 7.7 Hz, 1H), 7.41 – 7.29 (m, 5H), 7.29 – 7.12 (m, 8H), 5.93 (s, 1H), 1.92 (br, 1H).

**Naphthalen-2-yl(phenyl)methanol (3pa)[3]**

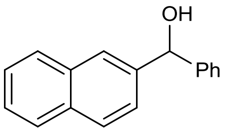


88% yield, white solid. $^{1}H$ NMR (400 MHz, $CDCl_3$) δ 7.88 (s, 1H), 7.86 – 7.74 (m, 3H), 7.51 – 7.38 (m, 5H), 7.34 (t, *J* = 7.4 Hz, 2H), 7.27 (t, *J* = 6.7 Hz, 1H), 5.99 (s, 1H), 2.33 (s, 1H).

**Phenyl(quinolin-3-yl)methanol (3qa)[9]**

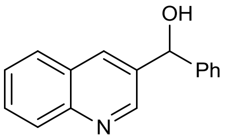


91% yield, white solid. $^{1}H$ NMR (400 MHz, $CDCl_3$) δ 8.82 (d, *J* = 1.9 Hz, 1H), 8.16 (s, 1H), 8.05 (d, *J* = 7.9 Hz, 1H), 7.78 (d, *J* = 7.9 Hz, 1H), 7.67 (t, *J* = 7.7 Hz, 1H), 7.52 (t, *J* = 7.9 Hz, 1H), 7.40 (d, *J* = 7.3 Hz, 2H), 7.35 (t, *J* = 7.3 Hz, 2H), 7.29 (t, *J* = 7.3 Hz, 1H), 6.04 (s, 1H), 2.92 (s, 1H).

**Benzo[b]thiophen-2-yl(phenyl)methanol (3ra)[11]**

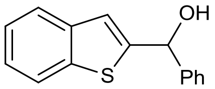


93% yield, yellow solid. $^{1}H$ NMR (400 MHz, $CDCl_3$) δ 7.76 (d, *J* = 7.2 Hz, 1H), 7.67 (d, *J* = 7.2 Hz, 1H), 7.48 (d, *J* = 7.2 Hz, 2H), 7.38 (t, *J* = 7.2 Hz, 2H), 7.35 – 7.25 (m, 3H), 7.11 (s, 1H), 6.10 (s, 1H), 2.53 (s, 1H).

**Benzofuran-2-yl(phenyl)methanol (3sa)[9]**

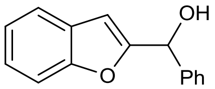


95% yield, white solid. $^{1}H$ NMR (400 MHz, $CDCl_3$) δ 7.53 – 7.31 (m, 7H), 7.27 – 7.22 (m, 1H), 7.19 (t, *J* = 7.4 Hz), 6.51 (s, 1H), 5.94 (s, 1H), 2.51 (s, 1H).

**Cyclohexyl(phenyl)methanol (3ta)**[9]

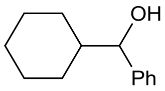


85% yield, colorless oil. $^1$H NMR (400 MHz, $CDCl_3$) δ 7.36 – 7.25 (m, 5H), 4.35 (d, *J* = 7.2 Hz, 1H), 2.01 - 1.95 (m, 1H), 1.79 – 1.71 (m, 1H), 1.67 – 1.55 (m, 3H), 1.30 – 1.32 (m, 1H), 1.29 – 0.85 (m, 5H).

## 1,3-Diphenylpropan-1-ol (3ua)[9]

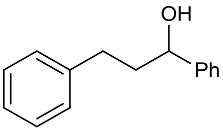


60% yield, colorless oil. $^1$H NMR (400 MHz, $CDCl_3$) δ 7.37 – 7.32 (m, 4H), 7.31 – 7.23 (m, 3H), 7.21 – 7.14 (m, 3H), 4.68 (dd, *J* = 7.8, 5.4 Hz, 1H), 2.81 – 2.60 (m, 2H), 2.21 – 1.94 (m, 2H), 1.79 (s, 1H).

## General procedure for phenylation of ketones

Under $N_2$ atmosphere, the mixture of ketone (0.40 mmol), tetraphenylphosphonium iodide (745.8 mg, 1.60 mmol) and $Cs_2CO_3$ (586.4 mg, 1.80 mmol) in THF (4 mL) was stirred at 65 $^{o}$C for 24 h. The reaction was quenched by 4.5 N HCl (1.5 mL). The resulting mixture was extracted with DCM (3 × 30 mL). The combined organic phase was dried over $Na_2SO_4$. After filtration, the solvent was removed by concentration, and the residue was subjected to column chromatography to afford the pure product.

**1-([1,1'-Biphenyl]-4-yl)-1-phenylethanol (5a)[12]**

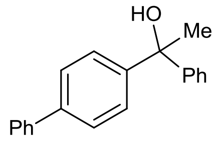


75% yield, white solid. $^{1}$H NMR (400 MHz, $CDCl_3$) δ 7.69 – 7.52 (m, 4H), 7.50 – 7.38 (m, 6H), 7.37 – 7.29 (m, 3H), 7.28 – 7.22 (m, 1H), 2.19 (s, 1H), 1.98 (s, 3H).

**1-(4-Fluorophenyl)-1-phenylethanol (5b)[13]**

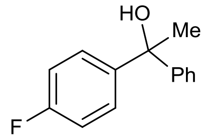


74% yield, coloreless oil. $^{1}$H NMR (400 MHz, $CDCl_3$) δ 7.41 – 7.34 (m, 4H), 7.31 (t, *J* = 7.4 Hz, 2H), 7.27 – 7.19 (m, 1H), 6.97 (t, *J* = 8.8 Hz, 2H), 2.11 (br, 1H), 1.92 (s, 3H). $^{19}$F NMR (376 MHz, $CDCl_3$) δ -116.15 - -116.24 (m, 1F).

**4-(1-Hydroxy-1-phenylethyl)benzonitrile (5c)[14]**

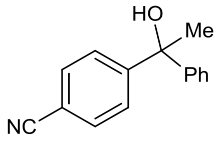


65% yield, white solid. $^{1}$H NMR (400 MHz, $CDCl_3$) δ 7.59 (d, *J* = 8.2 Hz, 2H), 7.53 (d, *J* = 8.2 Hz, 2H), 7.39 (d, *J* = 7.3 Hz, 2H), 7.34 (t, *J* = 7.3 Hz, 2H), 7.27 (t, *J* = 7.3 Hz, 1H), 2.14 (s, 1H), 1.95 (s, 3H).

**Phenyl-1-(4-(trifluoromethyl)phenyl)ethanol (5d)[15]**

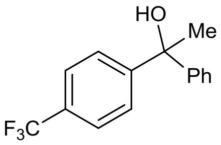


86% yield, coloreless oil. $^{1}$H NMR (400 MHz, $CDCl_3$) δ 7.55 (d, *J* = 8.9 Hz, 2H), 7.52 (d, *J* = 8.9 Hz, 2H), 7.40 (d, *J* = 7.4 Hz, 2H), 7.32 (t, *J* = 7.4 Hz, 2H), 7.28 – 7.25 (m, 1H), 2.02 (br, 1H), 1.95 (s, 3H). $^{19}$F NMR (376 MHz, $CDCl_3$) δ -62.49 (s, 3F).

**Phenyl-1-(4-(trifluoromethyl)phenyl)propan-1-ol (5e)[16]**

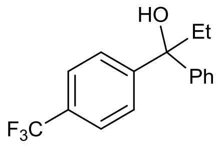


87% yield, coloreless oil. $^1$H NMR (400 MHz, $CDCl_3$) δ 7.58 – 7.47 (m, 4H), 7.40 (d, *J* = 7.5 Hz, 2H), 7.31 (t, *J* = 7.5 Hz, 2H), 7.26 – 7.20 (m, 1H), 2.59 – 2.26 (m, 2H), 2.02 (br, 1H), 0.87 (t, *J* = 7.3 Hz, 3H). $^{19}$F NMR (376 MHz, $CDCl_3$) δ -62.47 (s, 3F).

**2-Methyl-1,1-diphenylpropan-1-ol (5f)[16]**

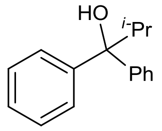


65% yield, colorless oil. $^1$H NMR (400 MHz, $CDCl_3$) δ 7.49 (d, *J* = 7.6 Hz, 4H), 7.27 (t, *J* = 7.6 Hz, 4H), 7.15 (t, *J* = 7.6 Hz, 2H), 2.96 – 2.81 (m, 1H), 1.98 (br, 1H), 0.88 (d, *J* = 6.7 Hz, 6H).

**Pyclohexyldiphenylmethanol (5g)[17]**

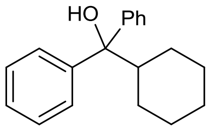


60% yield, white solid. $^1$H NMR (400 MHz, $CDCl_3$) δ 7.47 (d, *J* = 7.6 Hz, 4H), 7.28 (t, *J* = 7.6 Hz, 4H), 7.15 (t, *J* = 7.6 Hz, 2H), 2.47-2.37 (m, 1H), 1.96 (br, 1H), 1.80-1.53 (m, 5H), 1.38 – 1.22 (m, 2H), 1.19 – 1.00 (m, 3H).

**2,2-Dimethyl-1,1-diphenylpropan-1-ol (5h)[18]**

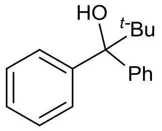


50% yield, colorless oil. $^1$H NMR (400 MHz, $CDCl_3$) δ 7.52 (d, *J* = 7.6 Hz, 4H), 7.36 (t, *J* = 7.6 Hz, 4H), 7.19 (t, *J* = 7.6 Hz, 2H), 2.26 (br, 1H), 1.17 (s, 9H).

**1,1,2-Triphenylethanol (5i)[19]**

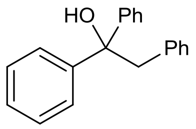


60% yield, white solid. $^1$H NMR (400 MHz, $CDCl_3$) δ 7.41 (d, *J* = 7.6 Hz, 4H), 7.29 (t, *J* = 7.6 Hz, 4H), 7.21 (t, *J* = 7.6 Hz, 2H), 7.17-7.11 (m, 3H), 6.88 (d, *J* = 7.8 Hz, 2H), 3.63 (s, 2H), 2.27 (br, 1H).

**Diphenyl(4-(trifluoromethyl)phenyl)methanol (5j)**

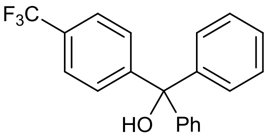


89% yield, white solid. M.P. 184.8 °C. $^1$H NMR (400 MHz, $CDCl_3$) δ 7.55 (d, *J* = 8.3 Hz, 2H), 7.44 (d, *J* = 8.3 Hz, 2H), 7.36 – 7.26 (m, 6H), 7.26 – 7.21 (m, 4H), 2.78 (s, 1H). $^{19}$F NMR (376 MHz, $CDCl_3$) δ -62.52 (s, 3F). $^{13}$C NMR (101 MHz, $CDCl_3$) δ 150.60 (s), 146.16 (s), 129.34 (q, *J* = 32.4 Hz), 128.24 (s), 124.17 (q, *J* = 272.9 Hz), 127.87 (s), 127.69 (s), 124.87 (q, *J* = 3.8 Hz), 122.82 (s), 81.83 (s). IR(KBr): 3420, 3062, 2926, 2838, 1328, 1115, 1069, 1019, 898, 836, 760, 701, 668, 639 $^{-1}$cm. HRMS (EI): calcd. for $[C_{20}H_{15}OF_3]^+$ $[M]^+$ 328.1075, found 328.1071.

**Ethyl 2-hydroxy-2,2-diphenylacetate (5k)[20]**

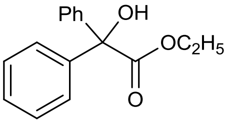


63% yield, colorless oil. $^1$H NMR (400 MHz, $CDCl_3$) δ 7.46 – 7.40 (m, 4H), 7.38 – 7.28 (m, 6H), 4.32 (q, *J* = 7.1 Hz, 2H), 4.17(br, 1H), 1.27 (t, *J* = 7.1 Hz, 3H).

**1-(Naphthalen-2-yl)-1-phenylethanol (5l)[16]**

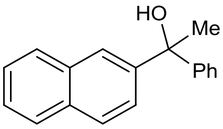


66% yield, white solid. $^1$H NMR (400 MHz, $CDCl_3$) δ 7.96 (s, 1H), 7.83 (d, *J* = 7.6 Hz, 1H), 7.79 (d, *J* = 7.6 Hz, 1H)., 7.75 (d, *J* = 8.7 Hz, 1H), 7.50 – 7.42 (m, 4H), 7.40 (dd, *J* = 7.0, 1.7 Hz, 1H), 7.31 (t, *J* = 7.5 Hz, 2H), 7.24 (t, *J* = 7.5 Hz, 1H), 2.27 (s, 1H), 2.04 (s, 3H).

**1-Phenylcyclopentanol (5m)[21]**

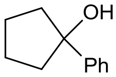


60% yield, colorless oil. $^1$H NMR (400 MHz, $CDCl_3$) δ 7.49 (d, *J* = 7.5 Hz, 2H), 7.34 (t, *J* = 7.5 Hz, 2H), 7.27 – 7.21 (m, 1H), 2.05 - 1.94 (m, 6H), 1.87 – 1.79 (m, 2H).

**2,4-Diphenylbutan-2-ol (5n)[22]**

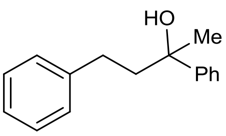


56% yield, colorless oil. $^1$H NMR (400 MHz, $CDCl_3$) δ 7.48 (d, *J* = 7.5 Hz, 2H), 7.37 (t, *J* = 7.5 Hz, 2H), 7.29 – 7.21 (m, 3H), 7.18 – 7.09 (m, 3H), 2.69 – 2.56 (m, 1H), 2.51 – 2.39 (m, 1H), 2.22 – 2.04 (m, 2H), 1.86 (br, 1H), 1.61 (s, 3H).

## General procedure for the synthesis of imines [23]

All imines were synthesized according to the procedure reported in literature

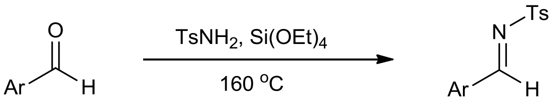


**6**

The mixture of aldehyde (31.5 mmol, 1.05 equiv), sulfonamide (30.0 mmol, 1.0 equiv) and tetraethyl orthosilicate (120 mmol, 25 g, 4.0 equiv) was stirred at 160 °C until no ethanol was produced. After the reaction system was cooled to room temperature, ethyl acetate/n-hexane (1:3) was added to precipitate the crude product. After filtration, the solid was washed by ethyl acetate/n-hexane(1:3) followed by ethanol to give the pure product.

### (*E*)-*N*-Benzylidene-4-methylbenzenesulfonamide (6a)[24]

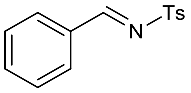


85% yield, white solid. $^{1}$H NMR (500 MHz, 400 MHz) δ 9.04 (s, 1H), 7.94 (d, *J* = 7.5 Hz, 2H), 7.90 (d, *J* = 8.2 Hz, 2H), 7.62 (t, *J* = 7.5 Hz, 1H), 7.50 (t, *J* = 7.57 Hz, 2H), 7.35 (d, *J* = 8.2 Hz, 2H), 2.45 (s, 3H).

### (*E*)-*N*-([1,1'-Biphenyl]-4-ylmethylene)-4-methylbenzenesulfonamide (6b)[24]

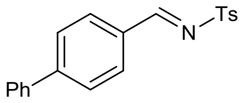


82% yield, white solid. $^{1}$H NMR (400 MHz, $CDCl_3$) δ 9.05 (s, 1H), 7.98 (d, *J* = 7.9 Hz, 2H), 7.89 (d, *J* = 8.0 Hz, 2H), 7.70 (d, *J* = 7.9 Hz, 2H), 7.61 (d, *J* = 7.2 Hz, 2H), 7.46 (t, *J* = 7.2 Hz, 2H), 7.43 – 7.37 (m, 1H), 7.34 (d, *J* = 8.0 Hz, 2H), 2.43 (s, 3H).

### (*E*)-4-Methyl-*N*-(4-methylbenzylidene)benzenesulfonamide (6c)[24]

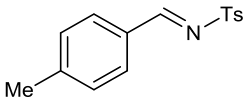


88% yield, white solid. $^{1}$H NMR (400 MHz, $CDCl_3$) δ 8.97 (s, 1H), 7.86 (d, *J* = 8.0 Hz, 2H), 7.79 (d, *J* = 8.0 Hz, 2H), 7.32 (d, *J* = 7.8 Hz, 2H), 7.26 (d, *J* = 7.8 Hz, 2H), 2.45 – 2.38 (m, 6H).

### (*E*)-4-Methyl-*N*-(2,4,6-trimethylbenzylidene)benzenesulfonamide (6d)

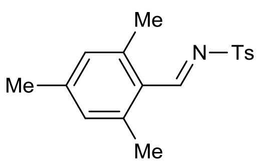


84% yield, white solid, M.P. 114.9 °C. $^{1}$H NMR (400 MHz, $CDCl_3$) δ 9.44 (s, 1H), 7.86 (d, *J* = 8.0 Hz, 2H), 7.31 (d, *J* = 8.0 Hz, 2H), 6.90 (s, 2H), 2.51 (s, 6H), 2.41 (s, 3H), 2.29 (s, 3H). $^{13}$C NMR (126 MHz, $CDCl_3$) δ 168.98 (s), 144.66 (s), 144.18 (s), 142.92 (s), 136.01 (s), 130.64 (s), 129.71

(s), 127.79 (s), 126.12 (s), 21.77 (s), 21.62 (s), 21.54 (s). IR(KBr): 2954, 2922, 1588, 1556, 1494, 1453, 1384, 1372, 1316, 1291, 1220, 1186, 1152, 1089, 1032, 1019, 998, 857, 833, 817, 774, 723, 704, 669, 592, 566, 540 $cm^{-1}$. HRMS (ESI): calcd. for $[C_{17}H_{20}O_2NS]^+$ $[M + H]^+$ 302.1209, found 302.1207.

**(*E*)-*N*-(4-Methoxybenzylidene)-4-methylbenzenesulfonamide (6e)**[24]

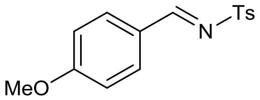


90% yield, white solid. $^1$H NMR (300 MHz, $CDCl_3$) δ 8.92 (s, 1H), 7.89 - 7.83 (m, 4H), 7.31 (d, *J* = 8.2 Hz, 2H), 6.94 (d, *J* = 8.8 Hz, 2H), 3.86 (s, 3H), 2.41 (s, 3H).

***N*-(2-Methoxybenzylidene)-4-methylbenzenesulfonamide (6f)**[24]

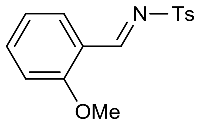


78% yield, white solid. $^1$H NMR (400 MHz, $CDCl_3$) δ 9.52 (s, 1H), 8.03 (d, *J* = 7.9 Hz, 1H), 7.86 (d, *J* = 7.7 Hz, 2H), 7.53 (t, *J* = 7.9 Hz, 1H), 7.30 (d, *J* = 7.7 Hz, 2H), 6.99 – 6.90 (m, 2H), 3.89 (s, 3H), 2.40 (s, 3H).

**(*E*)-4-Methyl-*N*-(4-(trifluoromethoxy)benzylidene)benzenesulfonamide (6g)**

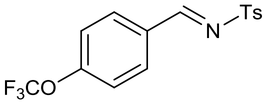


85% yield, white solid, M.P. 112.3 °C. $^1$H NMR (400 MHz, $CDCl_3$) δ 9.00 (s, 1H), 7.96 (d, *J* = 8.7 Hz, 2H), 7.86 (d, *J* = 8.3 Hz, 2H), 7.34 (d, *J* = 8.3 Hz, 2H), 7.29 (d, *J* = 8.7 Hz, 2H), 2.42 (s, 3H). $^{19}$F NMR (376 MHz, $CDCl_3$) δ -57.61 (s, 3F). $^{13}$C NMR (126 MHz, $CDCl_3$) δ 168.34 (s), 153.76 (s), 144.84 (s), 134.83 (s), 133.06 (s), 130.61 (s), 129.86 (s), 128.12 (s), 120.80 (s), 120.19 (q, *J* = 259.2 Hz), 21.59 (s). IR(KBr): 3096, 2475, 1926, 1607, 1582, 1506, 1421, 1390, 1320, 1305, 1255, 1157, 1088, 1016, 999, 975, 926, 873, 856, 807, 781, 729, 704, 675, 626, 566, 557, 532, 498 $cm^{-1}$. HRMS (ESI): calcd. for $[C_{15}H_{13}O_3NF_3S]^+$ $[M + H]^+$ 344.0563, found 344.0561.

**(*E*)-*N*-(4-(Benzyloxy)benzylidene)-4-methylbenzenesulfonamide (6h)**

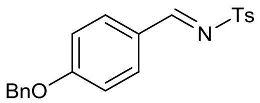


88% yield, white solid, M.P. 159.1 °C. $^1$H NMR (400 MHz, $CDCl_3$) δ 8.92 (s, 1H), 7.89 - 7.83 (m, 4H), 7.43 – 7.28 (m, 7H), 7.02 (d, *J* = 8.8 Hz, 2H), 5.12 (s, 2H), 2.41 (s, 3H). $^{13}$C NMR (126 MHz, $CDCl_3$) δ 169.18 (s), 164.39 (s), 144.29 (s), 135.77 (s), 135.74 (s), 133.72 (s), 129.76 (s), 128.74 (s), 128.38 (s), 127.89 (s), 127.53 (s), 125.38 (s), 115.53 (s), 70.35 (s), 21.64 (s). IR(KBr): 3033, 2874, 1586, 1565, 1509, 1467, 1455, 1424, 1390, 1372, 1313, 1303, 1287, 1258, 1152, 1162, 1085, 1009, 929, 881, 834, 820, 808, 778, 746, 701, 675, 634, 578, 558, 533 $cm^{-1}$. HRMS (ESI): calcd. for $[C_{21}H_{20}O_3NS]^+$ $[M + H]^+$ 366.1158, found 366.1156.

**(*E*)-*N*-(4-Fluorobenzylidene)-4-methylbenzenesulfonamide (6i)**[24]

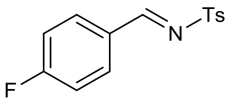


85% yield, white solid. $^1$H NMR (400 MHz, $CDCl_3$) δ 8.98 (s, 1H), 7.94 (dd, *J* = 8.5, 5.5 Hz, 2H), 7.87 (d, *J* = 8.2 Hz, 2H), 7.33 (d, *J* = 8.2 Hz, 2H), 7.16 (t, *J* = 8.5 Hz, 2H), 2.42 (s, 3H); $^{19}$F NMR (376 MHz, CDCl3) δ -101.03 - 101.13 (m, 1F).

**(*E*)-4-Methyl-*N*-(naphthalen-2-ylmethylene)benzenesulfonamide (6j)**[24]

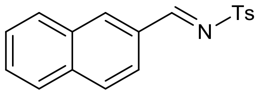


88% yield, white solid. $^1$H NMR (400 MHz, $CDCl_3$) δ 9.16 (s, 1H), 8.32 (s, 1H), 8.02 (d, *J* = 8.3 Hz, 1H), 7.96 - 7.84 (m, 5H), 7.62 (t, *J* = 7.4 Hz, 1H), 7.56 (t, *J* = 7.4 Hz, 1H), 7.34 (d, *J* = 7.6 Hz, 2H), 2.43 (s, 3H).

## General procedure for phenylation of imines

Under $N_2$ atmosphere, the mixture of imine (0.50 mmol), tetraphenylphosphonium iodide (583.0 mg, 1.25 mmol) and $Cs_2CO_3$ (488.7 mg, 1.50 mmol) in THF (4 mL) was stirred at 65 °C for 12 h. The reaction was quenched by 3 N HCl (0.5 mL). The resulting mixture was extracted with DCM (3 × 30 mL). The combined organic phase was dried over $Na_2SO_4$. After filtration, the solvent was removed by concentration. The residue was subjected to column chromatography to afford the pure product.

**_N_-Benzhydryl-4-methylbenzenesulfonamide (7a)**[25]

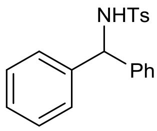


72% yield, white solid. $^1$H NMR (400 MHz, $CDCl_3$) δ 7.55 (d, *J* = 8.1 Hz, 2H), 7.23 – 7.17 (m, 6H), 7.13 (d, *J* = 8.1 Hz, 2H), 7.11 – 7.05 (m, 4H), 5.56 (d, *J* = 6.9 Hz, 1H), 4.99 (d, *J* = 6.9 Hz, 1H), 2.37 (s, 3H).

**_N_-([1,1'-Biphenyl]-4-yl(phenyl)methyl)-4-methylbenzenesulfonamide (7b)**[26]

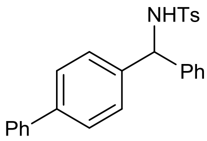


75% yield, white solid. $^1$H NMR (400 MHz, $CDCl_3$) δ 7.56 (d, *J* = 7.9 Hz, 2H), 7.50 (d, *J* = 8.2 Hz, 2H), 7.44 – 7.37 (m, 4H), 7.32 (t, *J* = 7.2 Hz, 1H), 7.23 – 7.18 (m, 3H), 7.18 – 7.08 (m, 6H), 5.60 (d, *J* = 7.0 Hz, 1H), 5.20 (d, *J* = 7.0 Hz, 1H), 2.34 (s, 3H).

**4-Methyl-_N_-(phenyl(p-tolyl)methyl)benzenesulfonamide (7c)**[26]

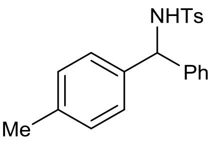


74% yield, white solid. $^1$H NMR (400 MHz, $CDCl_3$) δ 7.55 (d, *J* = 8.0 Hz, 2H), 7.23 – 7.16 (m, 3H), 7.13 (d, *J* = 8.0 Hz, 2H), 7.11 – 7.07 (m, 2H), 7.00 (d, *J* = 8.2 Hz, 2H), 6.95 (d, *J* = 8.2 Hz, 2H), 5.50 (d, *J* = 6.9 Hz, 1H), 5.01 (d, *J* = 6.9 Hz, 1H), 2.37 (s, 3H), 2.27 (s, 3H).

**_N_-(Mesityl(phenyl)methyl)-4-methylbenzenesulfonamide (7d)**

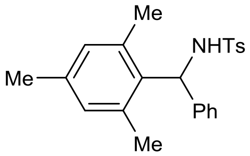


86% yield, white solid, M.P. 156.3 °C. $^1$H NMR (400 MHz, $CDCl_3$) δ 7.50 (d, *J* = 8.0 Hz, 2H), 7.30 – 7.11 (m, 5H), 7.07 (d, *J* = 8.2 Hz, 2H), 6.66 (s, 2H), 6.07 (d, *J* = 8.6 Hz, 1H), 5.15 (d, *J* = 8.6 Hz, 1H), 2.34 (s, 3H), 2.22 (s, 3H), 1.99 (s, 6H). $^{13}$C NMR (101 MHz, $CDCl_3$) δ 142.96 (s),

140.16 (s), 137.53 (s), 137.26 (s), 136.21 (s), 133.58 (s), 129.93 (s), 129.16 (s), 128.48 (s), 127.10 (s), 126.78 (s), 126.21 (s), 55.86 (s), 21.46 (s), 20.78 (s), 20.52 (s). IR(KBr): 3329, 3059, 3035, 2870, 2867, 1598, 1491, 1446, 1421, 1331, 1291, 1160, 1095, 1079, 1053, 1027, 905, 890, 848, 817, 136, 699, 670, 638, 589, 570, 552, 535, 520 cm$^{-1}$. HRMS (ESI): calcd. for $[C_{23}H_{26}NSO_2]^+$ [M + H]$^+$ 380.1679, found 380.1674.

**_N_-((4-Methoxyphenyl)(phenyl)methyl)-4-methylbenzenesulfonamide (7e)**[26]

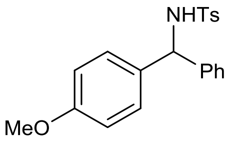


71% yield, white solid. $^1$H NMR (400 MHz, $CDCl_3$) δ 7.54 (d, *J* = 8.2 Hz, 2H), 7.22 – 7.15 (m, 3H), 7.12 (d, *J* = 8.2 Hz, 2H), 7.11 – 7.05 (m, 2H), 6.97 (d, *J* = 8.7 Hz, 2H), 6.71 (d, *J* = 8.7 Hz, 2H), 5.50 (d, *J* = 7.0 Hz, 1H), 5.10 (d, *J* = 7.0 Hz, 1H), 3.73 (s, 3H), 2.36 (s, 3H).

**_N_-((2-Methoxyphenyl)(phenyl)methyl)-4-methylbenzenesulfonamide (7f)**[25]

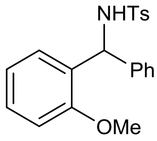


74% yield, white solid. $^1$H NMR (400 MHz, $CDCl_3$) δ 7.49 (d, *J* = 8.2 Hz, 2H), 7.23 – 7.09 (m, 6H), 7.03 (d, *J* = 8.2 Hz, 2H), 6.95 (d, *J* = 7.5 Hz, 1H), 6.76 (t, *J* = 7.5 Hz, 1H), 6.65 (d, *J* = 8.2 Hz, 1H), 5.73 (d, *J* = 8.6 Hz, 1H), 5.63 (d, *J* = 8.6 Hz, 1H), 3.57 (s, 3H), 2.30 (s, 3H).

**4-Methyl-_N_-(phenyl(4-(trifluoromethoxy)phenyl)methyl)benzenesulfonamide (7g)**

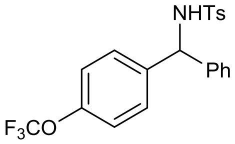


43% yield, white solid, M.P. 116.4 $^o$C. $^1$H NMR (400 MHz, $CDCl_3$) δ 7.53 (d, *J* = 8.0 Hz, 2H), 7.24 – 7.20 (m, 3H), 7.17 – 7.10 (m, 4H), 7.09 – 7.00 (m, 4H), 5.58 (d, *J* = 7.1 Hz, 1H), 5.12 (d, *J* = 7.1 Hz, 1H), 2.36 (s, 3H). $^{19}$F NMR (376 MHz, $CDCl_3$) δ -57.94 (s, 3F). $^{13}$C NMR (101 MHz, $CDCl_3$) δ 148.41 (s), 143.43 (s), 140.05 (s), 139.19 (s), 137.23 (s), 129.36 (s), 128.88 (s), 128.74 (s), 127.86 (s), 127.28 (s), 127.14 (s), 120.86 (s), 120.40 (q, *J* = 256.84), 60.73 (s), 21.37 (s). IR(KBr): 3240, 3062, 3040, 1598, 1507, 1497, 1452, 1316, 1259, 1220, 1173, 1158, 1094, 1063, 913, 876, 843, 812, 793, 743, 731, 697, 681, 668, 575, 556, 546 cm$^{-1}$. HRMS (ESI): calcd. for $[C_{21}H_{19}NF_3O_3S]^+$ [M + H]$^+$ 422.1032, found 422.1034.

***N*-((4-(Benzyloxy)phenyl)(phenyl)methyl)-4-methylbenzenesulfonamide (7h)**

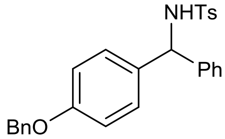


85% yield, white solid, M.P. 148.6 °C. $^1$H NMR (400 MHz, $CDCl_3$) δ 7.55 (d, *J* = 8.1 Hz, 2H), 7.45 – 7.28 (m, 5H), 7.23 – 7.17 (m, 3H), 7.16 – 7.06 (m, 4H), 6.99 (d, *J* = 8.6 Hz, 2H), 6.80 (d, *J* = 8.6 Hz, 2H), 5.51 (d, *J* = 6.9 Hz, 1H), 5.02 - 4.96 (m, 3H), 2.37 (s, 3H). $^{13}$C NMR (101 MHz, $CDCl_3$) δ 158.23 (s), 143.12 (s), 140.73 (s), 137.45 (s), 136.86 (s), 133.09 (s), 129.36 (s), 128.68 (s), 128.63 (s), 128.51 (s), 128.05 (s), 127.48 (s), 127.45 (s), 127.32 (s), 127.24 (s), 114.83 (s), 70.03 (s), 60.86 (s), 21.52 (s). IR(KBr): 3285, 3064, 3033, 3004, 2917, 2871, 1610, 1599, 1584, 1514, 1494, 1465, 1465, 1455, 1433, 1420, 1374, 1321, 1238, 1181, 1160, 1119, 1080, 1049, 1007, 990, 919, 910, 847, 809, 796, 741, 759, 700, 675, 570, 544 $cm^{-1}$. HRMS (ESI): calcd. for $[C_{27}H_{24}NO_3S]^+$ $[M + H]^+$ 442.1471, found 442.1469.

***N*-((4-Fluorophenyl)(phenyl)methyl)-4-methylbenzenesulfonamide (7i)[26]**

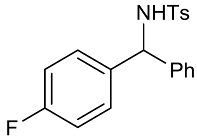


75% yield, white solid. $^1$H NMR (400 MHz, $CDCl_3$) δ 7.54 (d, *J* = 8.0 Hz, 2H), 7.23 – 7.18 (m, 3H), 7.14 (d, *J* = 8.0 Hz, 2H), 7.11 – 7.00 (m, 4H), 6.88 (t, *J* = 8.4 Hz, 2H), 5.54 (d, *J* = 6.9 Hz, 1H), 5.06 (d, *J* = 6.9 Hz, 1H), 2.38 (s, 3H). $^{19}$F NMR (376 MHz, $CDCl_3$) δ -114.68 - -114.80 (m, 1F).

**4-Methyl-*N*-(naphthalen-2-yl(phenyl)methyl)benzenesulfonamide (7j)[26]**

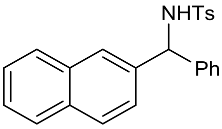


70% yield. white solid. $^1$H NMR (400 MHz, $CDCl_3$) δ 7.74 (dd, *J* = 6.0, 3.3 Hz, 1H), δ 7.66 (d, *J* = 8.9 Hz, 1H), 7.63 (dd, *J* = 6.0, 3.0 Hz, 1H), 7.53 (d, *J* = 8.2 Hz, 2H), 7.48 (s, 1H), 7.43 (dd, *J* = 6.2, 3.3 Hz, 1H), 7.23 – 7.18 (m, 3H), 7.17 – 7.11 (m, 3H), 7.02 (d, *J* = 8.0 Hz, 2H), 5.72 (d, *J* = 7.2 Hz, 1H), 5.14 (br, 1H), 2.26 (s, 3H).

## General procedure for arylation of 1a with teraarylphosphonium salt

Method A: Under $N_2$ atmosphere, the mixture of [1,1'-biphenyl]-4-carbaldehyde (**1a**) (91.2 mg, 0.50 mmol), tetraarylphosphonium iodide (1.25 mmol) and $Cs_2CO_3$ (488.7 mg, 1.50 mmol) in THF (4 mL) was stirred at 65 $^{o}$C until phosphonium salt was completely consumed as monitored by $^{31}$P NMR. The reaction was quenched by 3 N HCl (0.5 mL). The resulting mixture was extracted with DCM (3 × 30 mL). The combined organic phase was dried over $Na_2SO_4$, and the residue was subjected to column chromatography to afford the pure product.

Methmod B: Under $N_2$ atmosphere, into the mixture of [1,1'-biphenyl]-4-carbaldehyde (**1a**) (91.2 mg, 0.50 mmol) and $Cs_2CO_3$ (488.7 mg, 1.50 mmol) in THF (2 mL) at 65 $^{o}$C was added the solution of phosphonium bis(trifluoromethanesulfonyl)amide (1.25 mmol) in THF (2 mL) slowly for 6 h. On completion of addition, the reaction was stirred for another 10 minutes. The mixture was quenched by 3 N HCl (0.5 mL) and extracted with DCM (3 × 30 mL). The combined organic phase was dried over $Na_2SO_4$. After filtration, the solvent was removed by concentration, and the residue was subjected to column chromatography to afford the pure product.

**[1,1'-Biphenyl]-4-yl(4-methoxyphenyl)methanol (3ab)[27]**

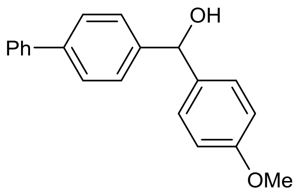


Method A: 55% yield, white solid. $^{1}$H NMR (400 MHz, $CDCl_3$) δ 7.58 – 7.53 (m, 4H), 7.45 – 7.38 (m, 4H), 7.35 – 7.29 (m, 3H), 6.88 (d, *J* = 8.7 Hz, 2H), 5.85 (s, 1H), 3.79 (s, 3H), 2.18 (s, 1H).

**[1,1'-Biphenyl]-4-yl(p-tolyl)methanol (3ac)[27]**

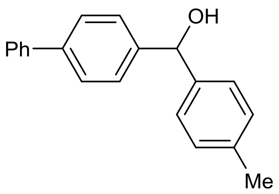


Method A: 49% yield, white solid. $^{1}$H NMR (400 MHz, $CDCl_3$) δ 7.57 – 7.52 (m, 4H), 7.47 – 7.38 (m, 4H), 7.35 – 7.27 (m, 3H), 7.15 (d, *J* = 7.9 Hz, 2H), 5.86 (s, 1H), 2.33 (s, 3H), 2.18 (s, 1H).

**di([1,1'-Biphenyl]-4-yl)methanol (3ad)[28]**

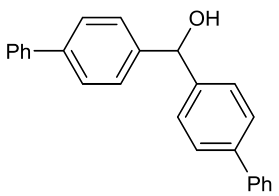


Method A: 35% yield, white solid. $^{1}$H NMR (400 MHz, $CDCl_3$) δ 7.61 – 7.54 (m, 8H), 7.49 (d, *J* = 8.2 Hz, 4H), 7.42 (t, *J* = 7.5 Hz, 4H), 7.33 (t, *J* = 7.5 Hz, 2H), 5.93 (s, 1H), 2.32 (s, 1H).

**4-([1,1'-Biphenyl]-4-yl(hydroxy)methyl)benzonitrile (3ae)**[27]

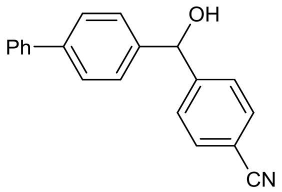

Method B: 73% yield, white solid. $^1$H NMR (400 MHz, $CDCl_3$) δ 7.63 (d, *J* = 8.5 Hz, 2H), 7.59 – 7.52 (m, 6H), 7.45 – 7.37 (m, 4H), 7.34 (t, *J* = 7.2 Hz, 1H), 5.91 (s, 1H), 2.32 (br, 1H).

**3-([1,1'-Biphenyl]-4-yl(hydroxy)methyl)benzonitrile (3af)**

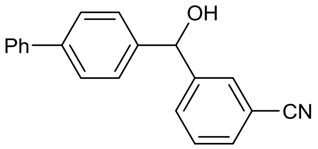

Method B: 75% yield, white solid, M.P. 92.1 $^o$C. $^1$H NMR (400 MHz, $CDCl_3$) δ 7.75 (s, 1H), 7.65 (d, *J* = 7.7 Hz, 1H), 7.61 – 7.53 (m, 5H), 7.49 – 7.38 (m, 5H), 7.35 (t, *J* = 7.1 Hz, 1H), 5.90 (s, 1H), 2.20 (br, 1H). $^{13}$C NMR (101 MHz, $CDCl_3$) δ 145.22 (s), 141.85 (s), 141.18 (s), 140.46 (s), 131.14 (s), 131.00 (s), 130.06 (s), 129.29 (s), 128.89 (s), 127.60 (s), 127.58 (s), 127.12 (s), 118.90 (s), 112.43 (s), 75.06 (s). IR(KBr): 3495, 3028, 2866, 2231, 1598, 1583, 1486, 1447, 1432, 1405, 1373, 1317, 1201, 1173, 1142, 1073, 1057, 1006, 907, 853, 830, 797, 767, 745, 696, 653, 599 cm$^{-1}$. HRMS (ESI): calcd. for $[C_{20}H_{16}ON]^+$ $[M + H]^+$ 286.1226, found 286.1225.

**[1,1'-Biphenyl]-4-yl(4-(trifluoromethyl)phenyl)methanol (3ag)**

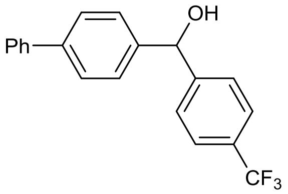

Method B: 78% yield, white solid, M.P. 97.0 $^o$C. $^1$H NMR (400 MHz, $CDCl_3$) δ 7.65 – 7.51 (m, 8H), 7.46 – 7.40 (m, 4H), 7.35 (d, *J* = 7.3 Hz, 1H), 5.94 (s, 1H), 2.07 (s, 1H). $^{19}$F NMR (376 MHz, $CDCl_3$) δ -62.50 (s, 3F). $^{13}$C NMR (101 MHz, $CDCl_3$) δ 147.50 (s), 142.14 (s), 141.06 (s), 140.56 (s), 129.76 (q, *J* = 32.5 Hz), 128.89 (s), 127.55 (s), 127.53 (s), 127.14 (s), 126.75 (s), 125.51 (q, *J* = 3.8 Hz), 124.01 (q, *J* = 272.7 Hz), 75.54 (s). IR(KBr): 33315, 3027, 2901, 1618, 1486, 1448, 1417, 1326, 1238, 1174, 1116, 1067, 1026, 1014, 867, 826, 810, 759, 723, 697, 633 cm$^{-1}$. HRMS (EI) : calcd. for $[C_{20}H_{16}OF_3]^+$ $[M]^+$ 328.1075, found 328.1078.

**ethyl 4-([1,1'-Biphenyl]-4-yl(hydroxy)methyl)benzoate (3ah)**

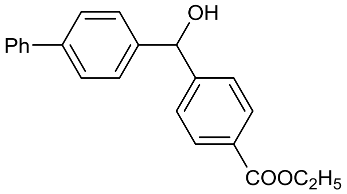

Method B: 66% yield, white solid, M.P. 109.5 $^o$C. $^1$H NMR (400 MHz, $CDCl_3$) δ 8.02 (d, *J* = 8.3 Hz, 2H), 7.58 – 7.52 (m, 4H), 7.50 (d, *J* = 8.2 Hz, 2H), 7.45 – 7.39 (m, 4H), 7.33 (t, *J* = 7.3 Hz,

1H), 5.93 (s, 1H), 4.36 (q, $J$ = 7.1 Hz, 2H), 1.90 (br, 1H), 1.37 (t, $J$ = 7.1 Hz, 3H). $^{13}$C NMR (101 MHz, $CDCl_3$) δ 166.56 (s), 148.69 (s), 142.39 (s), 140.81 (s), 140.65 (s), 129.83 (s), 129.62 (s), 128.83 (s), 127.44 (s), 127.40 (s), 127.15 (s), 127.11 (s), 126.36 (s), 75.67 (s), 61.03 (s), 14.35 (s). IR(KBr): 3476, 3052, 3034, 2984, 2906, 2863, 1689, 1608, 1576, 1566, 1488, 1776, 1441, 1423, 1402, 1367, 1313, 1283, 1183, 1160, 1129, 1107, 1031, 1017, 979, 878, 864, 855, 805, 779, 758, 739, 711, 687, 569 cm$^{-1}$. HRMS (ESI): calcd. for $[C_{22}H_{21}O_3]^+$ $[M + H]^+$ 333.1485, found 333.1483.

**1-(4-([1,1'-Biphenyl]-4-yl(hydroxy)methyl)phenyl)ethanone (3ai)**

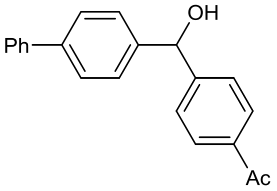


Method B: 35% yield, white solid, M.P. 136.9 $^{o}$C. $^{1}$H NMR (400 MHz, $CDCl_3$) δ 7.94 (d, $J$ = 8.3 Hz, 2H), 7.69 – 7.51 (m, 6H), 7.47 – 7.38 (m, 4H), 7.33 (t, $J$ = 7.3 Hz, 1H), 5.93 (s, 1H), 2.58 (s, 3H). $^{13}$C NMR (101 MHz, $CDCl_3$) δ 197.89 (s), 148.86 (s), 142.21 (s), 140.90 (s), 140.54 (s), 136.25 (s), 128.79 (s), 128.63 (s), 127.45 (s), 127.43 (s), 127.06 (s), 126.48 (s), 75.63 (s), 26.65 (s). IR(KBr): 3515, 3055, 3031, 2861, 1669, 1661, 1602, 1561, 1489, 1411, 1363, 1303, 1273, 1229, 1176, 1052, 1014, 1007, 962, 873, 827, 802, 766, 747, 732, 718, 698, 687, 603, 588 cm$^{-1}$. HRMS (ESI): calcd. for $[C_{21}H_{19}O_2]^+$ $[M + H]^+$ 303.1380, found 303.1378.

**[1,1'-Biphenyl]-4-yl(pyridin-2-yl)methanol (3aj)[7]**

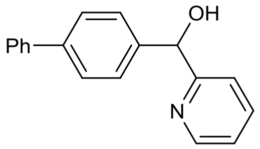


Method A: 73% yield, white solid. $^{1}$H NMR (400 MHz, $CDCl_3$) δ 8.57 (d, $J$ = 4.6 Hz, 1H), 7.63 (td, $J$ = 7.4, 1.8 Hz, 1H), 7.58 – 7.52 (m, 4H), 7.45 – 7.38 (m, 4H), 7.32 (t, $J$ = 7.5 Hz, 1H), 7.2 – 7.17 (m, 2H), 5.79 (s, 1H), 5.27 (br, 1H).

## The formation of $Ph_3P^{18}O$

Under $N_2$ atmosphere, the mixture of salt **2a** (583.0 mg, 1.25 mmol), $Cs_2CO_3$ (488.7 mg, 1.50 mmol) and $^{18}OH_2$ (30.0 mg, 1.5 mmol) in THF (4 mL) was stirred at 65 $^{o}$C for 12 h. The reaction was quenched by 3 N HCl (0.5 mL). The resulting mixture was extracted with DCM (3 × 30 mL). The combined organic phase was dried over $Na_2SO_4$. After filtration, the solvent was removed by concentration, and the residue was subjected to column chromatography to afford the $Ph_3P^{16}O/Ph_3P^{18}O$ mixture. The molar ratio was 1:1.26 determined by $^{31}$P NMR spectrometry.

$Ph_3P^{16}O/Ph_3P^{18}O$ (1:1.26)[29,30]: 99% yield. white solid. $^{1}$H NMR (400 MHz, $CDCl_3$) δ 7.74 – 7.65 (m, 12H), 7.57 (t, $J$ = 7.4Hz, 6H), 7.48 (td, $J$ = 7.4, 2.8 Hz, 12H). $^{31}$P NMR (162 MHz, $CDCl_3$) δ 29.12 (s, 1P), 29.08 (s, 1P). $^{13}$C NMR (101 MHz, $CDCl_3$) δ 132.60 (d, $J$ = 104.4 Hz), 132.09(d, $J$ = 10.0 Hz), 131.92 (d, $J$ = 2.7 Hz), 128.50 (d, $J$ = 12.1 Hz). Anal. Calcd for $C_{18}H_{15}{}^{16}O_{0.442}{}^{18}O_{0.558}$: C, 77.38; H, 5.41; P, 11.09; O, 6.12; Found: C, 77.22; H, 5.44; P, 11.09.

## The procedure for the synthesis of $Ph_5P$[31]

Under $N_2$ atmosphere, into a suspension of **2a** (4.66g, 10 mol) in dry diethyl ether (10 mml) was added the solution phenyl-lithium in diethyl ether (1 M, 11 mL). The mixture was stirred at room temperature for 8 days. The solvent was decanted and the solid was washed with dry diethyl ether (5 ml X 3) under nitrogen. The solid was then dissolved with dry THF (10 ml). After fast filtration, the solvent was removed under vaccum to give the product as a white solid.

Pentaphenylphosphorane: 60% yield, white solid. $^1H$ NMR (400 MHz, $CDCl_3$) δ 7.41 – 7.30 (m, 10H), 7.18-7.11 (m, 15H). $^{31}P$ NMR (162 MHz, $CDCl_3$) δ -84.81 (s, 1P). $^{13}C$ NMR (101 MHz, $CDCl_3$) δ 150.68 (d, $J$ = 75.9 Hz), 132.50 (d, $J$ = 8.3 Hz), 127.00 (s), 126.89 (s).

## The procedure for arylation of 1a with $Ph_5P$

Under $N_2$ atmosphere, the mixture of [1,1'-biphenyl]-4-carbaldehyde (**1a**) (91.2 mg, 0.50 mmol), $Ph_5P$ (520.2 mg, 1.25 mmol) and $Cs_2CO_3$ (488.8 mg, 1.50 mmol) in THF (4 mL) and stirred at 65 $^oC$ for 6 h. The reaction was quenched by 3 N HCl (0.5 mL). The resulting mixture was extracted with DCM (3 × 30 mL). The combined organic phase was dried over $Na_2SO_4$. After filtration, the solvent was removed by concentration, and the residue was subjected to column chromatography to afford the pure **3a** in 11% yield.

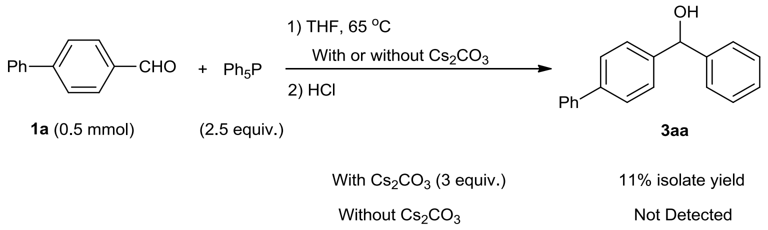


The use of $Ph_5P$ instead of salt **2a** for the phenylation of aldehyde **1a** was also examined. Although $Ph_5P$ can react with the aldehyde, the desired product was not detected at all without the presence of $Cs_2CO_3$. Even with $Cs_2CO_3$ present, the yield obtained was very low (11%), meaning that $Ph_5P$ should not be an important intermediate for this arylation reaction.

## Determination of $CO_2$ by GC-MS spectroscopy

In a glove box under $N_2$ atmosphere, [1,1'-biphenyl]-4-carbaldehyde (**1a**, 92.2 mg, 0.5 mmol), tetraphenylphosphonium iodide (583.2 mg, 1.25 mmol), $Cs_2CO_3$ (489.0 mg, 1.5 mmol) and DMF (4 ml) were added into a 25 mL Schlenk tube. The tube was sealed and taken out from the glove box. After being stirred at 65 $^oC$ for 12 h, the mixture was cooled to room temperature. In the gas phase, $CO_2$ was detected by GC-MS spectroscopy. In this gas phase, $N_2$ and benzene were also detected, which is reasonable because the reaction was performed under $N_2$ atmosphere, and the in situ generated phenyl anion equivalent might be readily trapped by proton to give benzene.

## The analysis of trace transition metals in reagents

Trace transition metals were analyzed by SPECTRO ARCOS ICP-OES (inductively coupled plasma optical emission spectrometry).

Sample **2a:**

The procedure for the preparation of sample **2a**: salt **2a** (150 mg) was nitrated by refluxing the mixture of salt **2a** and concentrated $HNO_3$ solution (5 mL, 60%~65%). The nitration was complete when slightly yellow transparent solution was obtained. The solution was then concentrated to 1 mL by distillation under atmospheric pressure. The 1-mL solution was transferred to a 50-mL volumetric flask and diluted by deionized water to 50 mL.

Samples **2b, 2j** and $Cs_2CO_3$ were made according to the procedure for the preparation of sample **2a**.

The procedure to make blank sample**:** Concentrated $HNO_3$ solution (5 mL, 60%~65%) was refluxed for the same period of time as that for the nitration of salt **2a**. The solution was then concentrated to 1 mL by distillation under atmospheric pressure. The 1-mL solution was transferred to a 50-mL volumetric flask and diluted by deionized water to 50 mL.

For $^{1}H$, $^{19}F$, $^{31}P$ and $^{13}C$ NMR spectra of the compounds in this article, see Supplementary Figures 1-139; for GC-MS spectra for the determination of $CO_2$, see Supplementary Figures 140-143; For ICP results, see Supplementary Table 1, and for ICP spectra, see Supplementary Figures 144-175.

## Supplementary References


1 Marcoux, D. & Charette, A. B. Palladium-Catalyzed Synthesis of Functionalized Tetraarylphosphonium Salts. *J. Org. Chem.* **73**, 590-593 (2008).

2 Sugimoto, O., Shimada, M., Sato, A. & Tanji, K.-i. Preparation and Reaction of Quinolinyl (or Pyridinyl) Phosphonium Salts with Base and Pivalaldehyde. *Heterocycles* **83**, 837-847 (2011).

3 Zhou, F. & Li, C.-J. the barbier-grignard type reaction arylatin of aldehydes using unactivated aryl iodides in water. *Nat. Commun.* **5**, 4254 - 4260 (2014).

4 Qin, C., Wu, H., Cheng, J., Chen, X. a., Liu, M., Zhang, W., Su, W. & Ding, J. The Palladium-Catalyzed Addition of Aryl- and Heteroarylboronic Acids to Aldehydes. *J. Org. Chem.* **72**, 4102-4107 (2007).

5 Denizaltı, S., Türkmen, H. & Çetinkaya, B. Chelating alkoxy NHC–Rh(I) complexes and their applications in the arylation of aldehydes. *Tetrahedron Lett.* **55**, 4129-4132 (2014).

6 Yu, A., Cheng, B., Wu, Y., Li, J. & Wei, K. Cyclopalladated complexes catalyzed addition of arylboronic acids to aldehydes in neat water. *Tetrahedron Lett.* **49**, 5405-5407 (2008).

7 Ching-Tien, L. & H., L. B. Nonracemic Diarylmethanols From CuH-Catalyzed Hydrosilylation of Diaryl Ketones. *Org. lett.* **10**, 4187-4190 (2008).

8 Tatsushi, I. & Yoshinori, K. A New Strategy for Deprotonative Functionalization of Aromatics: Transformations with Excellent Chemoselectivity and Unique Regioselectivities Using t-Bu-P4 Base. *J. Am. Chem. Soc.* **125**, 8082-8083 (2003).

9 Kuriyama, M., Shimazawa, R. & Shirai, R. Efficient 1,2-Addition of Aryl- and Alkenylboronic Acids to Aldehydes Catalyzed by the Palladium/Thioether−Imidazolinium Chloride System. *J. Org. Chem.* **73**, 1597-1600 (2008).

10 Krasovskiy, A. & Knochel, P. A LiCl-Mediated BrMg Exchange Reaction for. *Angew*

*Chem Int Ed Engl*, **43**, 3333-3336 (2004).
11 Kuriyama, M., Ishiyama, N., Shimazawa, R. & Onomura, O. Palladium-imidazolinium carbene-catalyzed arylation of aldehydes with arylboronic acids in water. *Tetrahedron* **66**, 6814-6819 (2010).
12 Brousmiche, D. W., Xu, M., Lukeman, M. & Wan, P. Photohydration and Photosolvolysis of Biphenyl Alkenes and Alcohols via Biphenyl Quinone Methide-type Intermediates and Diarylmethyl Carbocations. *J. Am. Chem. Soc.* **125**, 12961-12970 (2003).
13 Maekawa, T., Sekizawa, H. & Itami, K. Controlled Alcohol–Carbonyl Interconversion by Nickel Catalysis. *Angew. Chem. Int. Ed.* **50**, 7022-7026 (2011).
14 Qvortrup, K., Rankic, D. A. & MacMillan, D. W. C. A General Strategy for Organocatalytic Activation of C–H Bonds via Photoredox Catalysis: Direct Arylation of Benzylic Ethers. *J. Am. Chem. Soc.* **136**, 626-629 (2014).
15 Bouffard, J. & Itami, K. A Nickel Catalyst for the Addition of Organoboronate Esters to Ketones and Aldehydes. *Org. lett.* **11**, 4410 - 4413 (2009).
16 Liao, Y.-X., Xing, C.-H. & Hu, Q.-S. Rhodium(I)/Diene-Catalyzed Addition Reactions of Arylborons with Ketones. *Org. lett.* **14**, 1544-1547 (2012).
17 Korenaga, T., Ko, A., Uotani, K., Tanaka, Y. & Sakai, T. Synthesis and Application of 2,6-Bis(trifluoromethyl)-4-pyridyl. *Angew. Chem. Int. Ed.* **50**, 10703-10707, (2011).
18 Lomas, J. S., Briand, S. & Fain, D. Reactions of thermally generated tert-butyl and di(tert-alkyl) ketyl radicals in toluene: cage effects and hydrogen transfer. *J. Org. Chem.* **56**, 166-175, (1991).
19 Gao, F. et al. A simple and efficient copper oxide-catalyzed Barbier–Grignard reaction of unactivated aryl or alkyl bromides with ester. *Tetrahedron Lett.* **55**, 880-883, (2014).
20 Miyamura, S., Satoh, T. & Miura, M. Rhodium-Catalyzed Diarylation of Oxalates Using Arylboron Compounds. *J. Org. Chem.* **72**, 2255-2257 (2007).
21 Aureliano Antunes, C. S., Bietti, M., Lanzalunga, O. & Salamone, M. Photolysis of 1-Alkylcycloalkanols in the Presence of (Diacetoxyiodo)benzene and I2. Intramolecular Selectivity in the β-Scission Reactions of the Intermediate 1-Alkylcycloalkoxyl Radicals. *J. Org. Chem.* **69**, 5281-5289 (2004).
22 Pulis, A. P., Blair, D. J., Torres, E. & Aggarwal, V. K. Synthesis of Enantioenriched Tertiary Boronic Esters by the Lithiation/Borylation of Secondary Alkyl Benzoates. *J. Am. Chem. Soc.* **135**, 16054-16057 (2013).
23 Love, B. E., Raje, P. S. & II, T. C. W. Preparation of N-Tosylaldimines. *Synlett* **7**, 493 - 494 (1994).
24 Yoshida, K., Akashi, N. & Yanagisawa, A. Asymmetric addition of diethylzinc to aldehydes catalyzed by new zinc-amides prepared by a rhodium-catalyzed asymmetric addition. *Tetrahedron: Asymmetry* **22**, 1225 - 1230 (2011).
25 Oi, S., Moro, M., Fukuhara, H., Kawanishi, T. & Inoue, Y. Rhodium-catalyzed addition of arylstannanes to carbon–heteroatom double bond. *Tetrahedron* **59**, 4351-4361 (2003).
26 Chen, C.-C. *et al.* Enantioselective and Rapid Rh-Catalyzed Arylation of N Tosyl- and N-Nosylaldimines in Methanol. *J. Org. Chem.* **79**, 8077-8085 (2014).
27 Zhang, R., Xu, Q, Zhang, X. C., Zhang, T. & Shi, M. Axially chiral C2-symmetric N-heterocyclic carbene (NHC) palladium complexes-catalyzed asymmetric arylation of aldehydes with arylboronic acids. *Tetrahedron: Asymmetry* **21**, 1928 - 1935 (2010).

28 Park, B. S., Lee, S. W., Kim, I. T., Tae, J. S. & Lee, S. H. Synthesis and photoluminescent properties of new ceramidine derivatives. *Heteroat. Chem.* **23**, 66-73 (2012).

29 Ananthnag, G. S., Mague, J. T. & Balakrishna, M. S. A cyclodiphosphazane based pincer ligand, [2,6-{u-($^{t}$BuN)$_2$P($^{t}$BuHN)PO}$_2$C$_6$H3I]: Ni$^{II}$, Pd$^{II}$, Pt$^{II}$ and Cu$^{I}$ complexes and catalytic studies. *Dalton Transactions* **44**, 3785-3793, (2015).

30 Goto, A. *et al.* Effects of Phosphorus Substituents on Reactions of a-Alkoxyphosphonium Salts with Nucleophiles. *Chem. Eur. J.* **18**, 11423-11432 (2012).

31 Wittig, G. & Rieber, M. Darstellung und Eigenschaften des Pentaphenyl-phosphors. *Justus Liebigs Annalen der Chemie* **562**, 187-192 (1949).
